# Rhodium(i)-catalyzed C6-selective C–H alkenylation and polyenylation of 2-pyridones with alkenyl and conjugated polyenyl carboxylic acids†
†Electronic supplementary information (ESI) available. CCDC 1874166. For ESI and crystallographic data in CIF or other electronic format see DOI: 10.1039/c9sc03672e


**DOI:** 10.1039/c9sc03672e

**Published:** 2019-09-09

**Authors:** Haoqiang Zhao, Xin Xu, Zhenli Luo, Lei Cao, Bohan Li, Huanrong Li, Lijin Xu, Qinghua Fan, Patrick J. Walsh

**Affiliations:** a Department of Chemistry , Renmin University of China , Beijing 100872 , China . Email: 20050062@ruc.edu.cn; b Beijing National Laboratory for Molecular Sciences and Institute of Chemistry , Chinese Academy of Sciences , Beijing , 100190 , China . Email: fanqh@iccas.ac.cn; c Roy and Diana Vagelos Laboratories , Penn/Merck Laboratory for High-Throughput Experimentation , Department of Chemistry , University of Pennsylvania , 231 South 34th Street , Philadelphia , Pennsylvania 19104-6323 , USA . Email: pwalsh@sas.upenn.edu

## Abstract

A versatile Rh(i)-catalyzed C6-selective decarbonylative C–H alkenylation of 2-pyridones with readily available, and inexpensive alkenyl carboxylic acids has been developed. This directed dehydrogenative cross-coupling reaction affords 6-alkenylated 2-pyridones that would otherwise be difficult to access using conventional C–H functionalization protocols. The reaction occurs with high efficiency and is tolerant of a broad range of functional groups. A wide scope of alkenyl carboxylic acids, including challenging conjugated polyene carboxylic acids, are amenable to this transformation and no addition of external oxidant is required. Mechanistic studies revealed that (1) Boc_2_O acts as the activator for the *in situ* transformation of the carboxylic acids into anhydrides before oxidative addition by the Rh catalyst, (2) a decarbonylation step is involved in the catalytic cycle, and (3) the C–H bond cleavage is likely the turnover-limiting step.

## Abstract 2

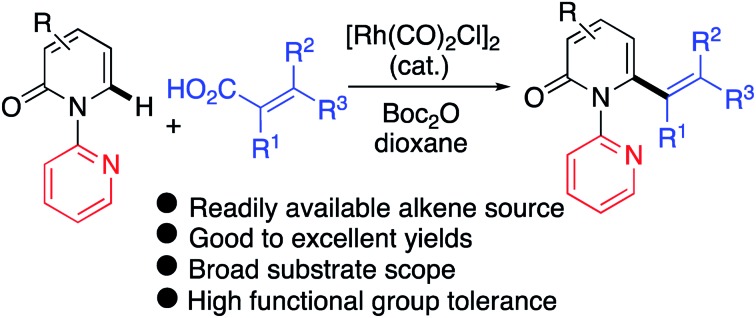
A versatile Rh(i)-catalyzed C6-selective decarbonylative C–H alkenylation of 2-pyridones with readily available alkenyl carboxylic acids has been developed.

## Introduction

**Fig. 1:**
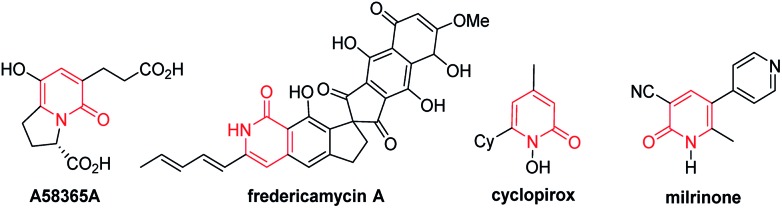
Biologically active 2(1*H*)-pyridone molecules.

The 2-pyridone motif is found in numerous naturally occurring molecules and synthetic organic compounds that possess a broad spectrum of bioactivities.^1^ For example, A58365A, isolated from the fermentation broth of a soil bacterium, serves as an angiotensin-converting enzyme inhibitor;1*f* fredericamycin A, isolated from *Streptomyces griseus*, is a potent antitumor antibiotic;1*g* ciclopirox is a widely used synthetic antifungal agent;1*h* and milrinone is a phosphodiesterase 3 inhibitor used to treat heart failure (Fig. 1).1*i* 2-Pyridones are also valued as building blocks, because they can be converted to pyridines, piperidines, quinolizidines and indolizidines.1*j* As a result of their widespread utility, the construction of 2-pyridones has been a vibrant research area in the synthetic community, and numerous methods for their synthesis are available.^2^,^3^

**Scheme 1:**
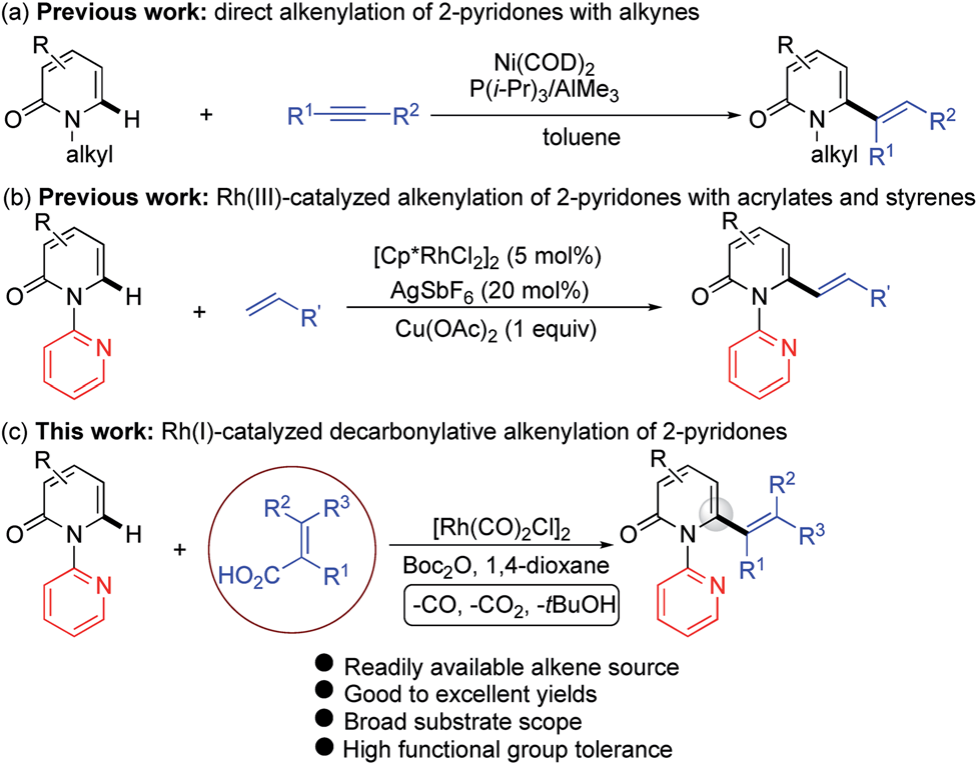
Catalytic direct C–H alkenylation of 2-pyridones at the C6 position: (a) alkenylation with alkynes, (b) alkenylation with acrylates and styrenes, and (c) decarbonylative alkenylation.

Several approaches for the functionalization of 2-pyridones have employed transition metals. Early studies focused on transition-metal catalyzed cross-coupling of functionalized 2-pyridones.^4^ More recent efforts to elaborate the 2-pyridone motif have been devoted to their direct catalytic C–H functionalization.2b,c In this context, rapid progress in site-selective C–H functionalization at C3, C5 and C6 positions of 2-pyridones has been advanced.^5^–^8^ Notably, Miura and co-workers found that the use of easily attachable and detachable 2-pyridyl directing groups at the nitrogen of the 2-pyridones could effectively facilitate the copper-mediated C6-selective dehydrogenative heteroarylation with 1,3-azoles.7*b* Following this seminal work, transition-metal catalyzed directed alkynylation,6*d* arylation,7h,j,o alkylation,7d,n,w,x borylation,7g,m thiolation,7*i* annulation,7e,f,p,r allylation,7l,q and amidation7t–v of 1-(2-pyridyl)-2-pyridones at the C6 positions have been successfully accomplished. In general, installation of vinyl groups has proven considerably more challenging than aryl or alkyl substituents, and this holds true for the vinylation of 2-pyridones at the C-6 position. Nakao and co-workers reported an impressive C6-alkenylation of 2-pyridones *via* C–H hydroarylation of *N*-alkylated 2-pyriodnes with alkynes at the C6 position under Ni/Al cooperative catalysis, albeit with limited substrate scope and low functional group tolerance (Scheme 1a).8*a* Very recently, the group of Hirano and Miura reported Rh(iii)-catalyzed (10 mol%) C6-selective alkenylation of 1-(2-pyridyl)-2-pyridones with acrylates and styrenes (Scheme 1b).8*b*

Recently, the use of readily available and inexpensive α,β-unsaturated carboxylic acids in transition metal catalyzed decarboxylative and decarbonylative alkenylation reactions has gained attention.^9^–^11^ We envisioned that 6-alkenylated 2-pyridones might be accessible from 1-(2-pyridyl)-2-pyridones and α,β-unsaturated acids under transition metal catalysis. In connection with our ongoing interests in direct alkenylation of C–H bonds,^12^ herein we report a Rh(i)-catalyzed C6-selective C–H alkenylation of 2-pyridones using alkenyl carboxylic acids as the vinyl source (Scheme 1c). This protocol features a simple and easy-to-handle catalytic system, high efficiency, very broad substrate scope and high functional group tolerance.

## Results and discussion

**Scheme 2:**
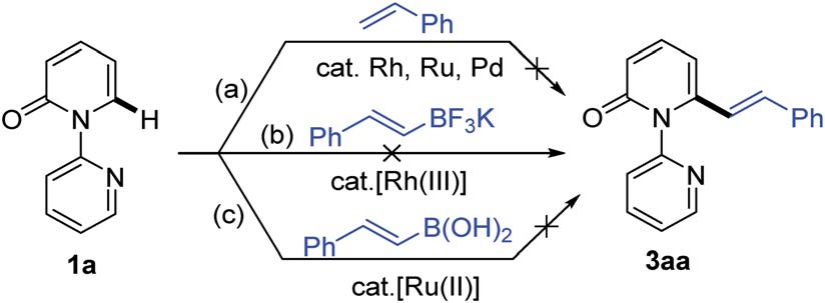
Unsuccessful catalytic direct alkenylations of 1-(2-pyridyl)-2-pyridone (**1a**).

Recent studies have revealed that catalytic systems based on Rh(iii), Ru(ii) and Pd(ii) complexes perform well in directed alkenylation of relatively inert (hetero)arene and alkene C–H bonds.^13^ Inspired by these reports, we first attempted the alkenylation of the model substrate 1-(2-pyridyl)-2-pyridone (**1a**) with styrene using Rh(iii), Ru(ii) and Pd(ii) complexes (ESI, Table S1†). Unfortunately, various catalytic systems, including those that have been shown to efficiently catalyze direct alkenylation of structurally similar 2-phenylpyrimidines, 1-(pyrimidin-2-yl)-1*H*-indoles and 2-(1*H*-pyrrol-1-yl)pyrimidines,^14^ did not furnish the desired products (Scheme 2a). Liu and co-workers recently described Rh(iii)-catalyzed site-selective C–H alkylation and arylation of 1-(2-pyridyl)-2-pyridones at the C6 position with potassium trifluoroborates.7*h* Expanding the substrate scope of this reaction to include potassium vinyl trifluoroborates, however, was unsuccessful in our hands using a similar Rh(iii) catalyst (Scheme 2b and ESI, Table S2†). Likewise, Ru(ii)-catalyzed alkenylation of **1a** with styrylboronic acids did not afford the desired alkenylation product (Scheme 2c and ESI, Table S2†).7*o*

We then turned our attention to the coupling reaction of vinyl carboxylic acids with 2-pyridones. We were pleased to discover that the reaction of **1a** and *trans*-cinnamic acid (**2a**) in the presence of [Rh(CO)_2_Cl]_2_ (1.0 mol%) and Boc_2_O (1.5 equiv.) at 130 °C in 1,4-dioxane, provided the desired product **3aa** in 92% yield after 6 h (Table 1, entry 1). A solvent screen revealed that 1,4-dioxane outperformed other frequently employed solvents, such as toluene, PhCl, *p*-xylene, THF, CH_3_CN, DCE, DMF and DME (Table 1, entries 2–9). Changing the rhodium source to [Rh(COD)Cl]_2_, [RhCl(PPh_3_)_3_], [Rh(COD)_2_BF_4_], or [Cp*RhCl_2_]_2_, did not lead to any improvement in the yield of **3aa** (Table 1, entries 10–13). Other transition metal complexes such as [Ru(*p*-cymene)Cl_2_]_2_, [Cp*IrCl_2_]_2_ and Pd(OAc)_2_ were also ineffective in this transformation (Table 1, entries 14–16).

**Table 1:** Optimization of the reaction conditions^*a*^

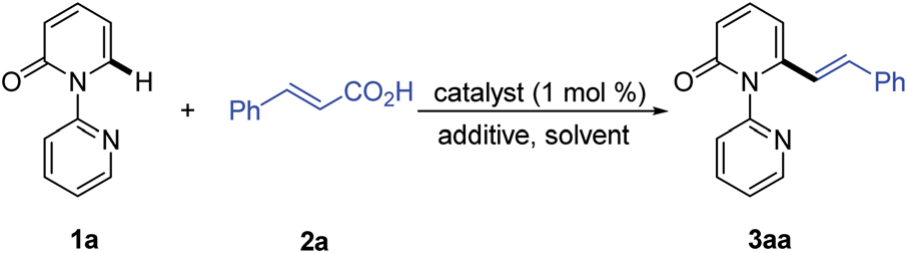				
Entry	Catalyst	Activator	Solvent	Yield (%)^*b*^
1	[Rh(CO)_2_Cl]_2_	Boc_2_O	1,4-Dioxane	93
2	[Rh(CO)_2_Cl]_2_	Boc_2_O	Toluene	15
3	[Rh(CO)_2_Cl]_2_	Boc_2_O	PhCl	11
4	[Rh(CO)_2_Cl]_2_	Boc_2_O	*p*-Xylene	15
5	[Rh(CO)_2_Cl]_2_	Boc_2_O	THF	NR
6	[Rh(CO)_2_Cl]_2_	Boc_2_O	CH_3_CN	NR
7	[Rh(CO)_2_Cl]_2_	Boc_2_O	DCE	10
8	[Rh(CO)_2_Cl]_2_	Boc_2_O	DMF	NR
9	[Rh(CO)_2_Cl]_2_	Boc_2_O	DME	NR
10	[Rh(COD)Cl]_2_	Boc_2_O	1,4-Dioxane	<5
11	[RhCl(PPh_3_)_3_]	Boc_2_O	1,4-Dioxane	NR
12	[Rh(COD)_2_BF_4_]	Boc_2_O	1,4-Dioxane	NR
13	[Cp*RhCl_2_]_2_	Boc_2_O	1,4-Dioxane	NR
14	[Ru(*p*-cymene)_2_Cl_2_]	Boc_2_O	1,4-Dioxane	NR
15	[Cp*IrCl_2_]_2_	Boc_2_O	1,4-Dioxane	NR
16	Pd(OAc)_2_	Boc_2_O	1,4-Dioxane	NR
17	[Rh(CO)_2_Cl]_2_	(MeOCO)_2_O	1,4-Dioxane	22
18	[Rh(CO)_2_Cl]_2_	Tf_2_O	1,4-Dioxane	NR
19	[Rh(CO)_2_Cl]_2_	(CF_3_CO)_2_O	1,4-Dioxane	NR
20	[Rh(CO)_2_Cl]_2_	PivCl	1,4-Dioxane	39
21	[Rh(CO)_2_Cl]_2_	Piv_2_O	1,4-Dioxane	92
22^*c*^	[Rh(CO)_2_Cl]_2_	Boc_2_O	1,4-Dioxane	55
23^*d*^	[Rh(CO)_2_Cl]_2_	Boc_2_O	1,4-Dioxane	43
24	None	Boc_2_O	1,4-Dioxane	NR
25	[Rh(CO)_2_Cl]_2_	None	1,4-Dioxane	NR
26^*e*^	[Rh(CO)_2_Cl]_2_	Boc_2_O	1,4-Dioxane	31

^*a*^Reaction conditions: **1a** (0.2 mmol), **2a** (0.22 mmol), catalyst (1.0 mol%), activator (1.5 equiv.), solvent (2.0 mL), 130 °C, 6 h, in air.

^*b*^Isolated yield.

^*c*^Reaction temperature 120 °C.

^*d*^[Rh(CO)_2_Cl]_2_ (0.5 mol%) was used.

^*e*^1-(Pyrimidin-2-yl)pyridin-2(1*H*)-one was employed.

We next screened different electrophiles to activate the unsaturated acid. Poor conversion was obtained with (MeOCO)_2_O (22%), Tf_2_O (NR), (CF_3_CO)_2_O (NR), or PivCl (39%) as the acid activators (Table 1, entries 17–20). In contrast, Piv_2_O was effective and gave **3aa** in 92% yield (Table 1, entry 21). Considering the price and compatibility, however, more economical and milder Boc_2_O was preferred.

Further optimization involving decreasing the reaction temperature or the catalyst loading led to dramatically lowered yields (Table 1, entries 22 and 23). Notably, the reaction did not proceed in the absence of either a rhodium catalyst or acid activator (Table 1, entries 24 and 25). Finally, the effect of the N-directing group in this reaction was examined. No reaction occurred when free 2-pyridone or 2-pyridone substrates bearing other substituents on the nitrogen, such as Me, Bn, Ph, or 3-pyridyl. The 2-pyrimidyl resulted in only 31% yield (Table 1, entry 26). These results clearly indicated that the judicious choice of the N-directing group is critical for catalysis in this transformation.

With the optimized conditions in hand, we investigated the scope of 1-(2-pyridyl)-2-pyridones with **2a** as the coupling partner (Table 2). It was found that a series of C3- and C4-substituted 1-(2-pyridyl)-2-pyridones (**1b–1l**) underwent smooth alkenylation with **2a** exclusively at the C6-position to deliver the corresponding products (**3ba–3la**) in good to excellent yields (77–91%) with high tolerance of functional groups, including halides at the 3- or 4-positions. Notably, the C5-substituted 2-pyridones (**1m–1q**) were compatible with our Rh-catalyzed system to afford the C6-alkenylated products (**3ma–3qa**) in 67–83% yield, despite the increased steric hindrance on C5. The 3,4-disubstituted 2-pyridones (**1r** and **1s**) were also readily engaged under the current conditions to give the corresponding products (**3ra** and **3sa**) in 75 and 63% yields, respectively. Substrates bearing electron donating or electron withdrawing substituents on the pyridyl rings (**1t–1v**) coupled smoothly with **2a** to generate the desired products (**3ta–3va**) in 83–89% yields. Moreover, this reaction could be readily extended to 4*H*-[1,2′-bipyridin]-4-one (**1w**) and 1-(pyridin-2-yl)quinolin-4(1*H*)-one (**1x**), thus producing **3wa** and **3xa** in 88 and 91% yields, respectively. It is notable that only the formation of the dialkenylated product was observed in the case of **1w**.

**Table 2:** Catalytic alkenylation of various 2-pyridones with **2a**^*a*^ ^,^^*b*^

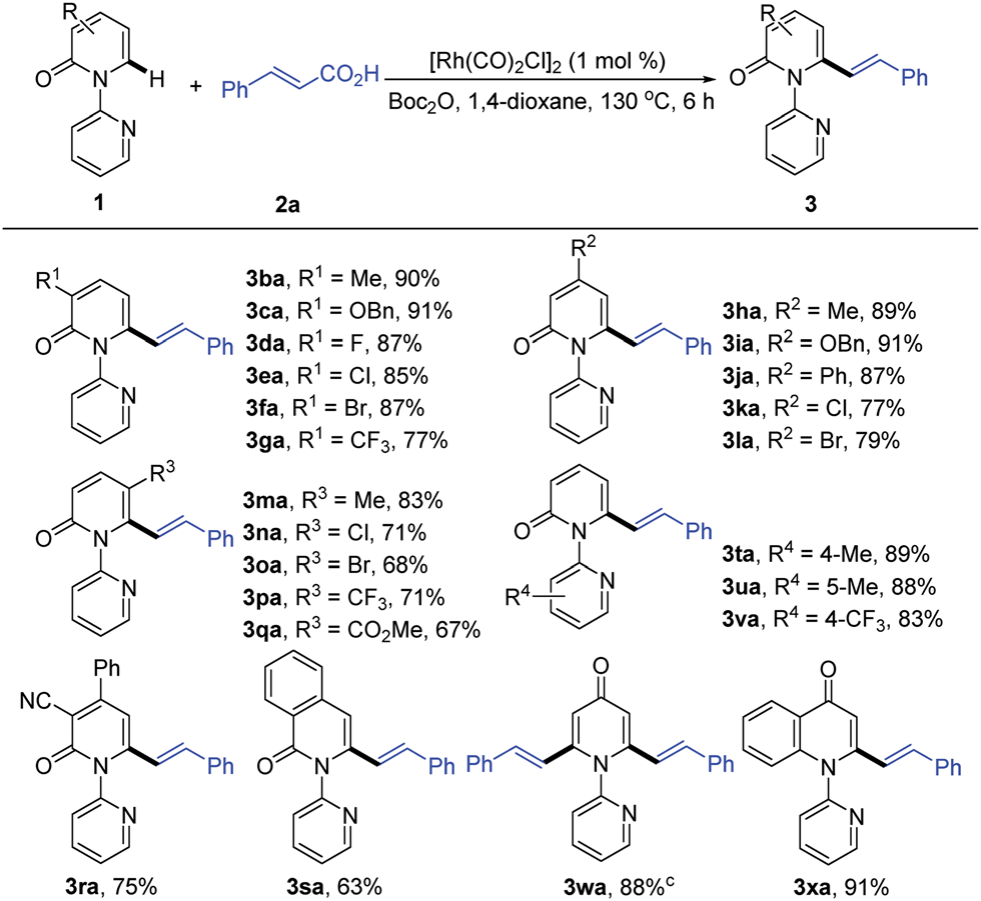

^*a*^Reaction conditions: **1a** (0.2 mmol), **2a** (0.22 mmol), [Rh(CO)_2_Cl]_2_ (1.0 mol%), Boc_2_O (1.5 equiv.), 1,4-dioxane (2.0 mL), 130 °C, 6 h, in air.

^*b*^Isolated yield.

^*c*^ **2a** (0.44 mmol) was employed.

Subsequently, we explored the reactivity of various cinnamic acids with **1a**. As shown in Table 3, a wide range of cinnamic acids (**2b–2p**) with mono-substituted aromatic rings efficiently participated in the alkenylation with **1a** to exclusively furnish the desired C6-alkenlayed 2-pyridone products (**3ab–3ap**) in good to excellent yields (77–92%). The alkenylation proved to be insensitive to the nature of the substituents on the aryl ring, with various electron-withdrawing (NO_2_, CO_2_Me and CN) and donating substituents (alkyl, OMe and NMe_2_) participating. Sensitive functional groups, including OH, B(OH)_2_, and halogens, were all well tolerated. The structure of **3ap** was confirmed by single-crystal X-ray diffraction (CCDC ; 1874166). Similarly, the more complex cinnamic acids (**2q–2v**), with polysubstituted aromatic rings, displayed good reactivity, affording the target products (**3aq–3av**) in 70–90% yields. Notably, a vinyl group bearing a pentafluoro phenyl provided the product (**3au**) in 70% yield. Heteroaryl groups are vital substructures in medicinal chemistry.^15^ We, therefore, examined the compatibility of heteroaryl cinnamic acids with **1a**. Heteroaryl cinnamic acids bearing 3-pyridyl, 2-furanyl, and 2-thiofuranyl (**2w–2y**) reacted smoothly with **1a** to give the desired products (**3aw–3ay**) in 82–90% yields. Importantly, the estrone-derived cinnamic acid **2z** proved to be equally effective in this transformation, indicating the robustness of the current catalytic system.

**Table 3:** Direct olefination of **1a** with cinnamic acids^*a*^ ^,^^*b*^

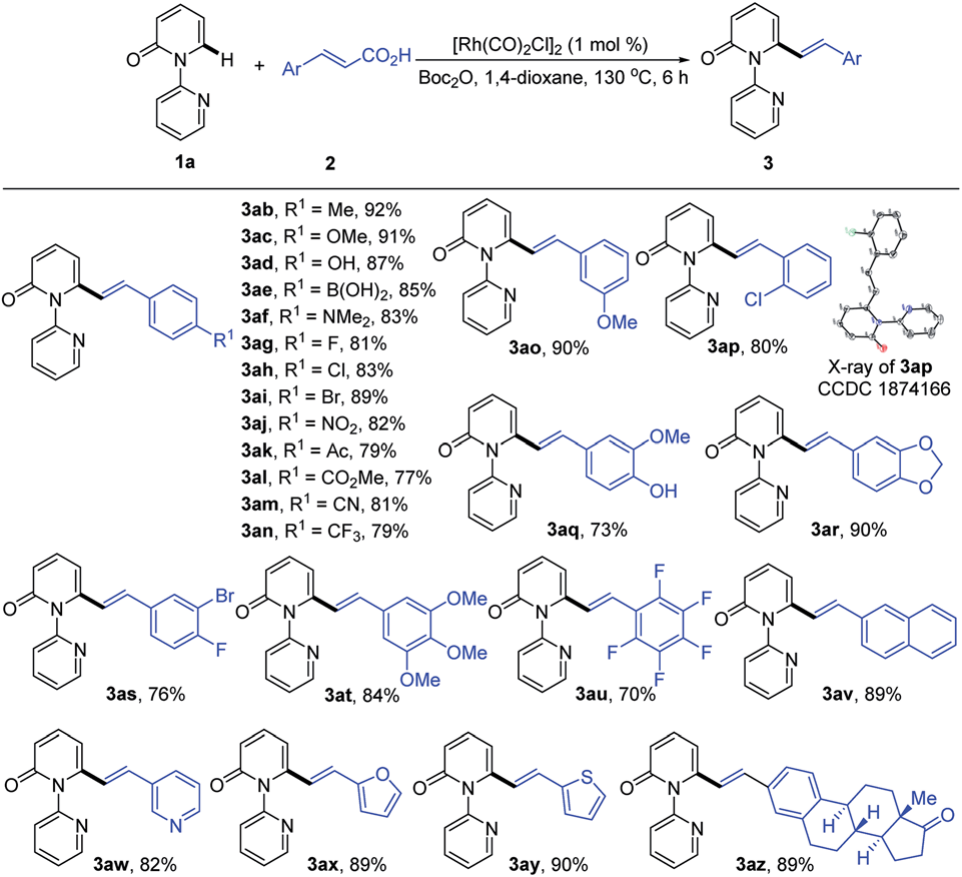

^*a*^Reaction conditions: **1a** (0.2 mmol), **2a** (0.22 mmol), [Rh(CO)_2_Cl]_2_ (1.0 mol%), Boc_2_O (1.5 equiv.), 1,4-dioxane (2.0 mL), 130 °C, 6 h, in air.

^*b*^Isolated yield.

To further demonstrate the potential of our catalytic system, the reaction was extended to other substituted alkenyl carboxylic acids, and the results are summarized in Table 4. It was found that treatment of various β-alkylated acrylic acids (**4a–4d**) with **1a** resulted in exclusive formation of C6-alkenylated 2-pyridone products (**5aa–5ad**) in 85–92% yields, irrespective of the nature of the β-alkyl groups. In the case of acid **4e** containing a sensitive Cl group, the reaction furnished the desired product **5ae** in 87% yield without dechlorination. Notably, the simple acrylic acid (**4f**) was also reactive, giving rise to the C6-vinylated 2-pyridone product **5af** in 75% yield. Likewise, the α-substituted acrylic acids **4g** and **4h** were competent substrates, delivering **5ag** and **5ah** both in 80% yield. Furthermore, trisubstituted acrylic acids (**4i–4n**), including the naturally occurring geranic acid (**4k**), shikimic acid (**4m**) and perillic acid (**4n**), were good substrates, producing **5ai–5an** in 63–91% yields. Potentially reactive groups, like OH and C=C, were not detrimental to the overall yields. Remarkably, a variety of conjugated polyene carboxylic acids were also efficient coupling partners in this transformation. More substituted and less sensitive conjugated dienyl carboxylic acids (**4o–4s**) formed the desired products (**5ao–5as**) in 65–86% yields. The formation of a mixture of *Z*/*E* isomers in the case of **5ar** was due to the low stereochemical purity of the starting trienoic acid **4r** (4*Z*/4*E* ratio 1 : 1). Surprisingly, both the bioactive retinoic acid (**4t**) and its derivative **4u** containing a conjugated hexaene unit, formed the corresponding products (**5at** and **5au**) in 72% and 65% yields, respectively. Application of 5-phenylpent-2-en-4-ynoic acid (**4v**) led to the formation of **5av** in 67% yield, with the alkyne having no obvious adverse effect on the reaction outcome.

**Table 4:** Direct olefination of **1a** with substituted alkenyl carboxylic acids^*a*^ ^,^^*b*^

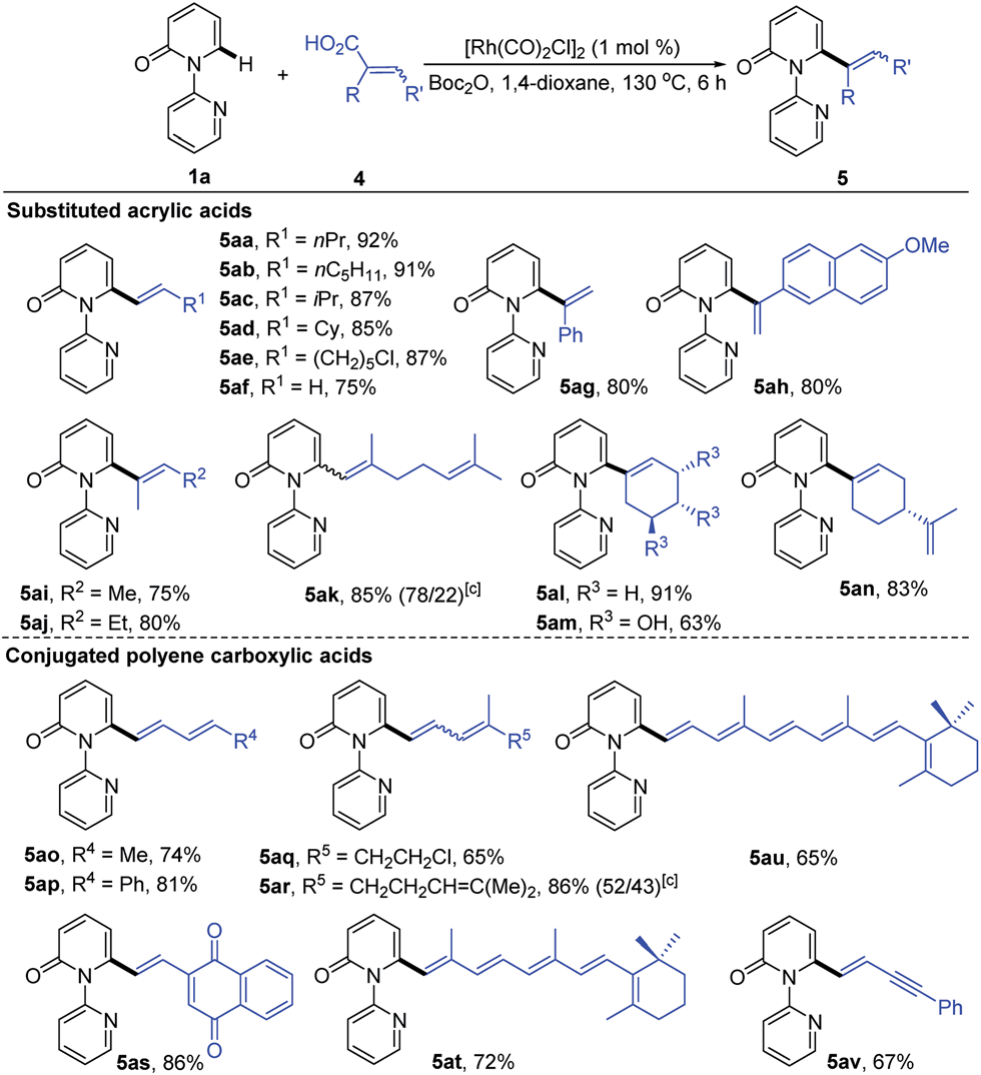

^*a*^Reaction conditions: **1a** (0.2 mmol), **2a** (0.22 mmol), [Rh(CO)_2_Cl]_2_ (1.0 mol%), Boc_2_O (1.5 equiv.), 1,4-dioxane (2.0 mL), 130 °C, 6 h, in air.

^*b*^Isolated yield.

^*c*^Ratio of isomers (*E*/*Z*).

**Scheme 3:**
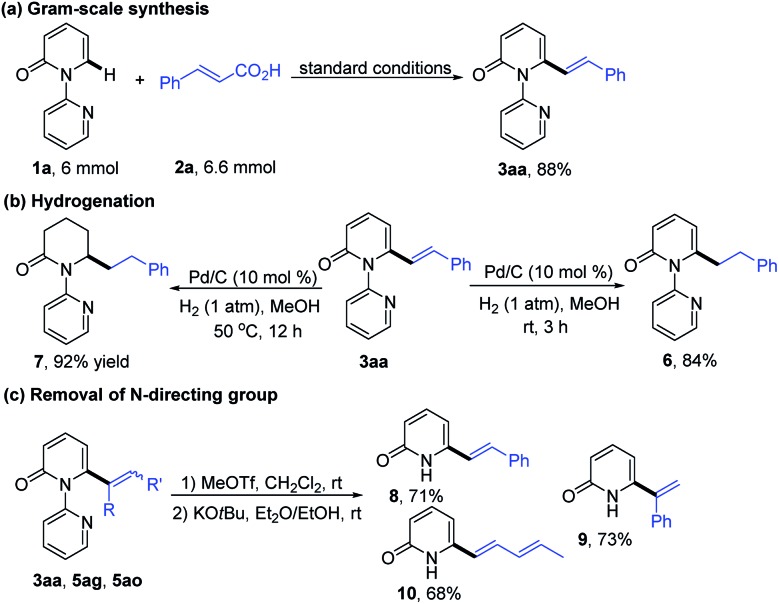
Synthetic applications: (a) gram-scale synthesis, (b) hydrogenation, and (c) deprotection.

In order to explore the synthetic practicality of this transformation, a gram scale reaction of **1a** and **2a** was performed to deliver **3aa** in 88% yield (Scheme 3a). Further transformations of the products were then explored. As depicted in Scheme 3b, hydrogenation of **3aa** at room temperature favored the reduction of the alkene moiety to generate the C6-alkylated 2-pyridone product **6** in 84% yield. Increasing the reaction temperature to 50 °C, however, enabled formation of piperidin-2-one product **7** (92% yield). The pyridine directing group could be conveniently removed by treatment with MeOTf and KO*t*Bu to give the C6-alkenylated 2-pyridone products in 68–73% yield (Scheme 3c).7*h*

**Scheme 4:**
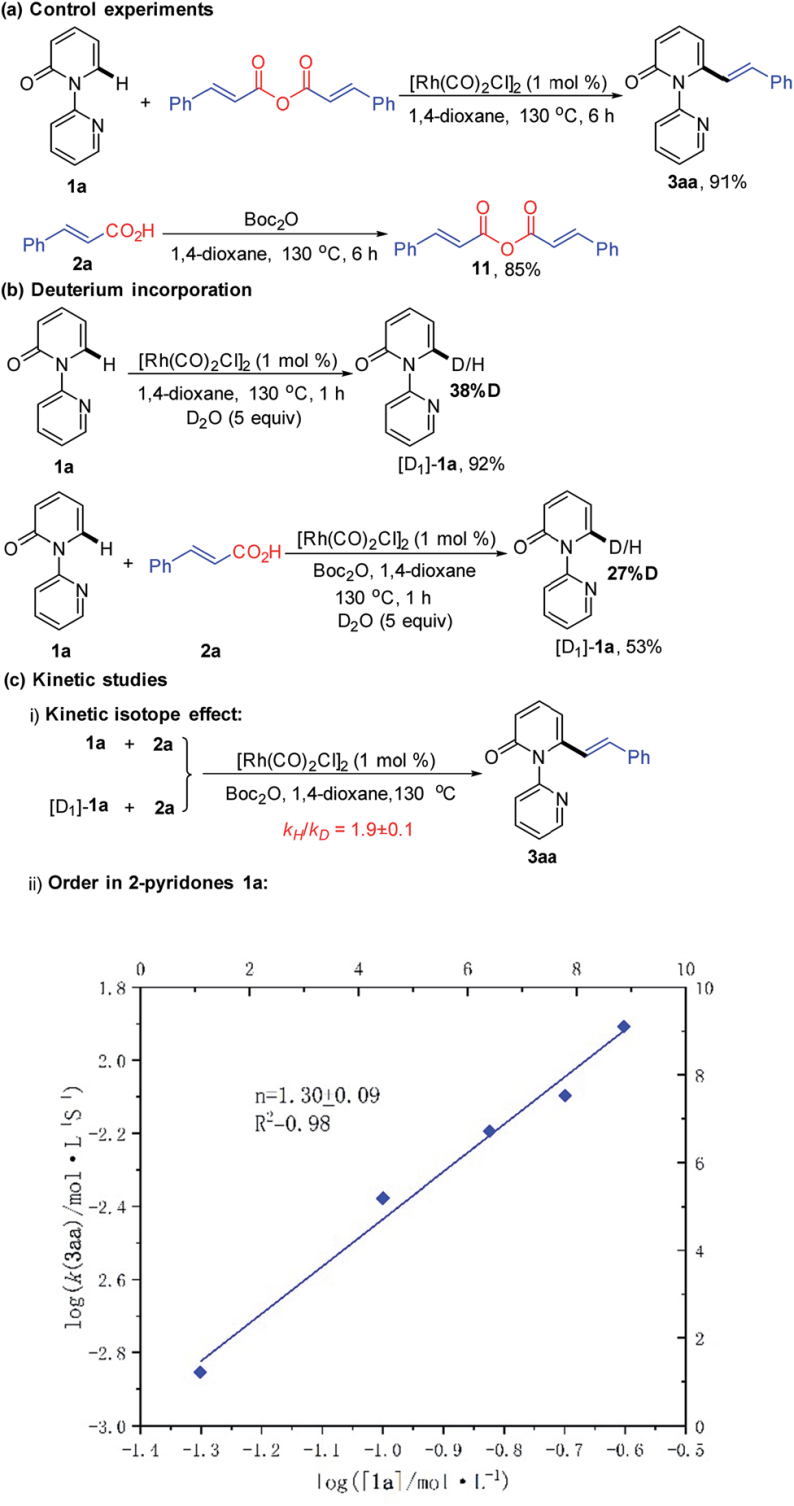
Mechanistic studies: (a) control experiments, (b) deuterium incorporation, and (c) kinetic studies.

We next desired to probe the basic steps of the reaction mechanism. Activation of the carboxylic acid was envisioned to proceed *via* an anhydride derivative.^16^ To test this hypothesis, a control experiment with cinnamic anhydride **11** and **1a** demonstrated that the coupling worked equally (91% yield) as well as acid **2a** with Boc_2_O (93% yield). Treatment of acid **2a** with an equimolar amount of Boc_2_O in 1,4-dioxane at 130 °C for 6 h led to the predominant formation of cinnamic anhydride **11** in 85% yield. This observation supports the involvement of *in situ* generation of the anhydride in the vinylation reaction.^16^ The generation of CO gas during the reaction was confirmed by analyzing the head gas of the reaction mixture with GC-TDC (ESI, Fig. S1†). Moreover, employing [Rh(COD)Cl]_2_ as the catalyst also generated CO gas albeit with a longer reaction time (18 h) and lower yield of **3aa** (50%) (ESI, Fig. S2†). These results rule out the possibility that CO gas might be derived from [Rh(CO)_2_Cl]_2_, thus indicating the presence of a decarbonylation step in the catalytic cycle. As shown in Scheme 4b, treatment of **1a** with D_2_O (5 equiv.) under the standard conditions for 1 h, in the absence or presence of **2a** resulted in approximately 38% and 27% deuteration at the C6-position, respectively, suggesting the reversibility of the C–H activation step under these conditions.

To gain insight into the turnover-limiting step, we conducted initial rate studies and a parallel kinetic isotope effect (KIE) on **1a**. The kinetic analyses highlighted a first-order (*n* = 1.30 ± 0.09) dependence on the concentration of **1a** for the reaction (Scheme 4c and ESI†). In separate reaction vessels, **1a** and [D_1_]-**1a** were subjected to identical reaction conditions (ESI†); it was observed that **1a** was alkenylated to **3aa** at a greater rate than the corresponding deuterium-labelled substrate. The KIE value determined from the average of five runs *via* the method of initial rates was 1.9 ± 0.1. This result implies that the C–H bond cleavage is likely involved in the turnover-limiting step.

**Scheme 5:**
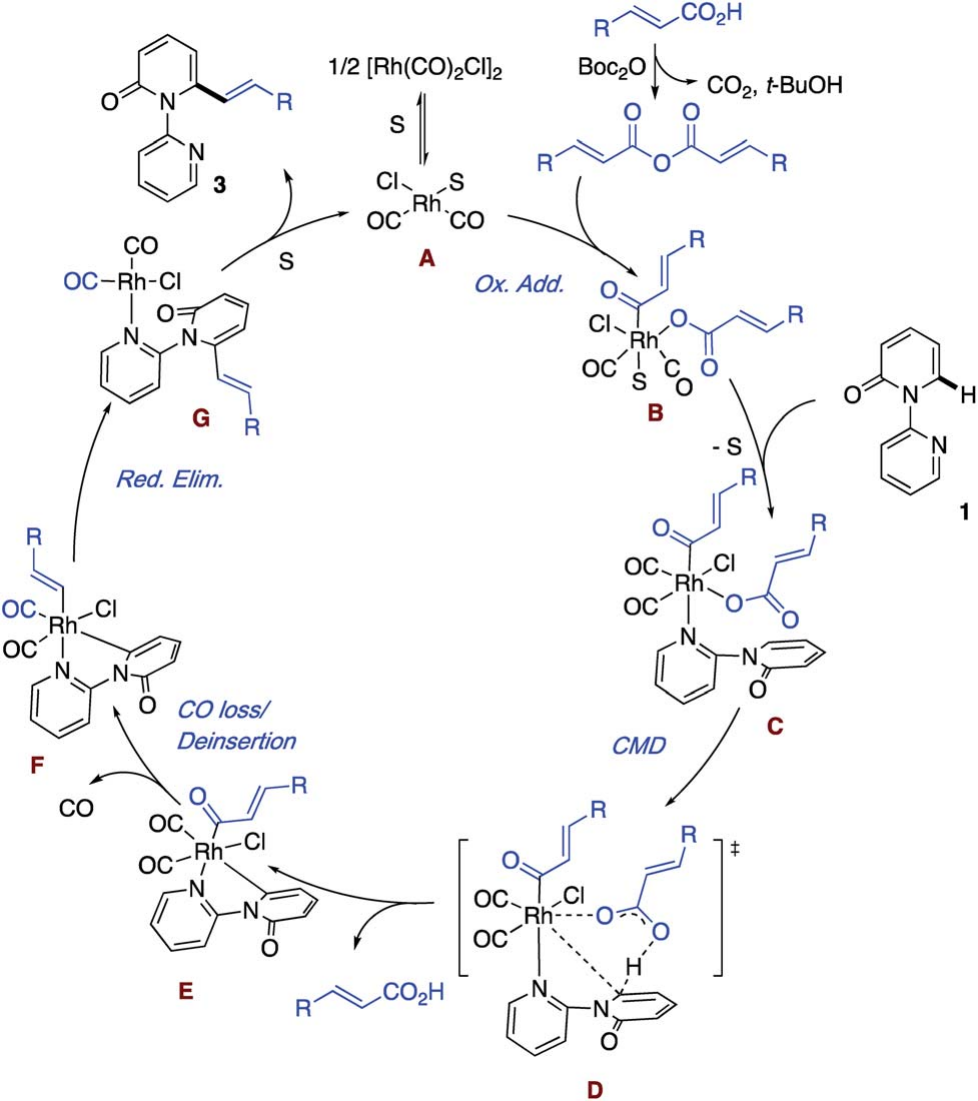
Plausible mechanism.

Based on the aforementioned results and literature precedence,^17^ a plausible mechanism highlighting the key steps is presented in Scheme 5. First, solvent (S) or the substrate pyridine breaks up the dimer [Rh(CO)_2_Cl]_2_ to give the monomer and enter the catalytic cycle. Meanwhile, the acid reacts with Boc_2_O to generate the anhydride, which undergoes oxidative addition to a Rh(i) species **A** and leads to the formation of the Rh(iii) intermediate **B**. In the event that S is solvent, ligand exchange for the substrate follows, giving intermediate **C**. Rather than a second oxidative addition, we prefer a concerted metalation deprotonation (CMD) by the carboxylate ligand *via* transition state **D** to generate the acid and the cyclometallated species with the key Rh–C bond. The liberated acid can react with the Boc_2_O to re-enter the cycle as the anhydride. **E** is envisioned to undergo loss of coordinated CO and then deinsertion of CO to afford the Rh–vinyl intermediate. Reductive elimination regenerates Rh(i) with the bound product **G**, which undergoes exchange with the solvent to liberate the product and close the catalytic cycle to form **A**. At this point, the exact ordering of the steps remains to be determined.

## Conclusions

We have developed the first Rh(i)-catalyzed decarbonylative alkenylation at C6 of 2-pyridones using readily available and inexpensive alkenyl carboxylic acids. This C6 alkenylation of 2-pyridones is applicable to the coupling of a wide range of substituted acyclic acids and conjugated polyene carboxylic acids. The reaction proceeds under oxidant-free conditions, enabling facile access to C6-alkenylated 2-pyridones in high yields with a broad functional group tolerance. Mechanistic studies support the following steps: initial activation of the carboxylic acid in the form of an anhydride, oxidative addition of the activated acid, coordination of the substrate followed by CMD to cleave the C–H bond. Dissociation of CO is followed by decarbonylation of the acyl group to generate the Rh-bound vinyl, and finally reductive elimination and liberation of product closes the cycle. A turnover limiting C–H bond cleavage is likely based on the observed KIE. Further investigation of the mechanism of this reaction and synthetic applications are underway in our laboratories.

## Conflicts of interest

There are no conflicts to declare.

## Supplementary Material

Supplementary information

Crystal structure data
