# Supplementary material for: Rhodium(i)-catalyzed C6-selective C–H alkenylation and polyenylation of 2-pyridones with alkenyl and conjugated polyenyl carboxylic acids
Source: Chem Sci. 2019 Sep 9;10(43):10089–96. doi: 10.1039/c9sc03672e (PMC6991184; doi:10.1039/c9sc03672e)
Supplement: Supplementary file 1 [file SC-010-C9SC03672E-s001.pdf]

Electronic Supplementary Material (ESI) for Chemical Science.
This journal is © The Royal Society of Chemistry 2019

Supporting Information for

Rhodium(I)-Catalyzed C6-Selective C–H Alkenylation and polyenylation of 2-Pyridones with Alkenyl and conjugated polyenyl Carboxylic Acids

Haoqiang Zhao,[a,c] Xin Xu,[a] Zhenli Luo,[a] Lei Cao[a], Bohan Li,[a] Huanrong Li,[a] Lijin Xu,[a,b]* Qinghua Fan,[b,]* and Patrick J. Walsh[c,]*

a. *Department of chemistry, Renmin University of China, Beijing 100872, China. E-mail: 20050062@ruc.edu.cn*

b. *Beijing National Laboratory for Molecular Sciences and Institute of Chemistry, Chinese Academy of Sciences, Beijing, 100190, China. E-mail: fanqh@iccas.ac.cn*

c. *Roy and Diana Vagelos Laboratories, Penn/Merck Laboratory for High-Throughput Experimentation, Department of Chemistry, University of Pennsylvania, 231 South 34th Street, Philadelphia, Pennsylvania 19104-6323, United States E-mail: pwalsh@sas.upenn.edu*

Contents:

1. General information S2
2. Optimization of the reaction conditions S2
3. Synthesis of substrates S6
4. The general procedure for direct alkenylation of 2-pyridones S7
5. Synthetic applications S8
6. The mechanistic studies S10
7. Characterization data for products S16
8. References: S34
9. Copies of ^{1}H and ^{13}C{^{1}H} NMR Spectra S35
10. X-ray crystal structure determination of 3ap S118

1. General information

Unless otherwise noted, all experiments were carried out in air and all commercially available chemicals including organic solvents were used as received from Aldrich, Acros or Strem without further purification. ^{1}H NMR and ^{13}C{^{1}H } NMR spectra were recorded on a Brüker Model Advance DMX 400 Spectrometer (^{1}H 400 MHz and ^{13}C 100.6 MHz, respectively). Chemical shifts (δ) are given in ppm and are referenced to residual solvent peaks. 2-Oxo-4-phenyl-1,2-dihydropyridine-3-carbonitrile,[1] methyl 6-oxo-1,6-dihydropyridine-3-carboxylate,[2] (*2E,4E*)-5-phenylpenta-2,4-dienoic acid,[3] (*2E,4E*)-5,9-dimethyldeca-2,4,8-trienoic acid,[4] (*S*)-4-(prop-1-en-2-yl)cyclohex-1-ene-1-carboxylic acid,[5] (*E*)-8-chlorooct-2-enoic acid[6] and 1-(2-pyridyl)-2-pyridones[7] were prepared according to the previous reports.

2. Optimization of the reaction conditions

Table S1 Unsuccessful attempts for the catalytic direct alkenylation of **1a** with styrene.

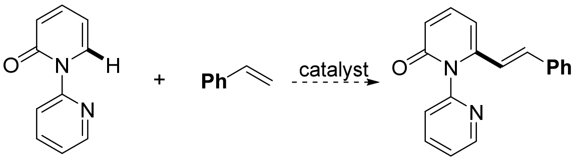

1a **3aa**

**Catalytic system**	**Yield** (%)
1. *Adv. Synth. Catal.* **2018**, *360*, 985-994.<br>**1a** (0.2 mmol), styrene (0.3 mmol), [Cp*RhCl$_2$]$_2$ (2.5 mol%), AgSbF$_6$ (10 mol%), Cu(OAc)$_2$ (80 mol%), O$_2$ (1 atm), DME, 130 °C, 2 h.	NR
2. *Chem. Eur. J.* **2015**, *21*, 9053-9056.<br>**1a** (0.1 mmol), styrene (1.0 mmol), [Cp^{E}RhCl$_2$]$_2$ (2.5 mol%), AgSbF$_6$ (10 mol%), Cu(OAc)$_2$·H$_2$O (20 mol%), acetone, under air, RT, 16 h.	NR
3. *Adv. Synth. Catal.* **2016**, *358*, 573-583.<br>**1a** (0.2 mmol), styrene (0.4 mmol), [Cp*RhCl$_2$]$_2$ (4.0 mol%), AgSbF$_6$ (20 mol%), Cu(OAc)$_2$·H$_2$O (80 mol%), MeOH, 140 °C, 24 h.	NR
4. *Chem. Commun.*, **2015**, *51*, 2532-2535.<br>**1a** (0.2 mmol), styrene (1.0 mmol), [Cp*RhCl$_2$]$_2$ (4.5 mol%), Cu(OAc)$_2$ (2.0 equiv.), DCE, 100 °C, 12 h.	NR
5. *Chem. Sci.*, **2015**, *6*, 1923-1927.<br>**1a** (0.2 mmol), styrene (0.5 mmol), [Cp*RhCl$_2$]$_2$ (5.0 mol%), NaOPiv (1.0 equiv.), MeCN, under air, 80 °C, 24 h.	NR
6. *Adv. Synth. Catal.* **2015**, *357*, 761-766.<br>**1a** (0.2 mmol), styrene (0.4 mmol), [Cp*RhCl$_2$]$_2$ (5.0 mol%), NaOAc (1.5 equiv.), AcOH (3.0 equiv.), DCE, 80 °C, 18 h.	NR
7. *Org. Lett.*, **2013**, *15*, 5662-5665.<br>**1a** (0.1 mmol), styrene (0.5 mmol), [Cp*RhCl$_2$]$_2$ (5.0 mol%), AgSbF$_6$ (20 mol%), Cu(OAc)$_2$ (2.5 equiv.), *t*-AmOH, 120 °C, 8 h.	NR

8. *J. Am. Chem. Soc.* **2013**, *135,* 468-473.<br>**1a** (0.2 mmol), styrene (0.3 mmol), [Cp*$RhCl_2$]$_2$ (2.0 mol%), $AgSbF_6$ (8.0 mol%), AgOAc (2.2 equiv.), MeOH, 70 °C, 36 h.	NR
9. *Chem. Commun.,* **2011**, *47,* 10458-10460.<br>**1a** (0.2 mmol), styrene (0.4 mmol), [Cp*$RhCl_2$]$_2$ (2.5 mol%), $AgSbF_6$ (10 mol%), Cu(OAc)$_2$ (2.0 equiv.), DME, 110 °C, 24 h.	NR
10. *Angew. Chem. Int. Ed.* **2012**, *51,* 7242-7245.<br>**1a** (0.2 mmol), styrene (0.5 mmol), [Cp*$RhCl_2$]$_2$ (5.0 mol%), AgOAc (30 mol%), Cu(OAc)$_2$·H_2O (2.0 equiv.), MeOH, under air, 90 °C, 24 h.	NR
11. *Org. Biomol. Chem.,* **2012**, *10,* 5521-5524.<br>**1a** (0.2 mmol), styrene (0.3 mmol), [Cp*$RhCl_2$]$_2$ (2.0 mol%), Cu(OAc)$_2$ (4.2 equiv.), MeCN, 110 °C, 12 h.	NR
12. *Angew. Chem. Int. Ed.* **2015**, *54,* 1657-1661.<br>**1a** (0.2 mmol), styrene (0.3 mmol), [Cp*$RhCl_2$]$_2$ (2.5 mol%), AgOAc (10 mol%), DCE/HOAc (3:1, v/v), RT, 24 h.	NR
13. *J. Am. Chem. Soc.* **2011**, *133,* 2350-2353.<br>**1a** (0.2 mmol), styrene (0.3 mmol), [Cp*$RhCl_2$]$_2$ (1.0 mol%), CsOAc (10 mol%), MeOH, 60 °C, 16 h.	NR
14. *Adv. Synth. Catal.,* **2014**, *356,* 137-143.<br>**1a** (0.2 mmol), styrene (0.24 mmol), [Cp*$RhCl_2$]$_2$ (5.0 mol%), Cu(OAc)$_2$·H_2O $_2$ (4.2 equiv.), DCE, under air, 60 °C, 5 h.	NR
15. *Angew. Chem. Int. Ed.* **2011**, *50,* 1064-1067.<br>**1a** (0.2 mmol), styrene (0.3 mmol), [Cp*$RhCl_2$]$_2$ (0.5 mol%), $AgSbF_6$ (2.0 mol%), Cu(OAc)$_2$ (2.1 equiv.), *t*-AmylOH, 120 °C, 16 h.	NR
16. *Angew. Chem. Int. Ed.* **2013**, *52,* 12430-12434.<br>**1a** (0.2 mmol), styrene (0.4 mmol), [Cp*Rh(MeCN)$_3$](SbF_6)$_2$ (5.0 mol%), PivOH (0.5 equiv.), DCM, 60 °C, 24 h.	NR
17. *Chem. Eur. J.* **2013**, *19,* 11863-11868.<br>**1a** (0.2 mmol), styrene (0.6 mmol), [Cp*Rh(MeCN)$_3$](SbF_6)$_2$ (2.0 mol%), Cu(OAc)$_2$·H_2O (2.0 equiv.), THF, 100 °C, 24 h.	NR
18. *Org. Lett.* **2015**, *17,* 3210-3213.<br>**1a** (0.2 mmol), styrene (0.3 mmol), [Cp*Rh (MeCN)$_3$](SbF_6)$_2$ (5.0 mol%), Cu(OAc)$_2$·H_2O (2.0 equiv.), TEMPO (1.0 equiv.), MeCN, 60 °C, 12 h.	NR
19. *J. Org. Chem.,* **2015**, *80,* 10457-10463.<br>**1a** (0.2 mmol), styrene (0.24 mmol), [Cp*(RhMeCN)$_3$](SbF_6)$_2$ (4.0 mol%), Cu(OAc)$_2$·H_2O (2.1 equiv.), AcOH (2.0 equiv.), DCE, 100 °C, 16 h.	NR
20. *ACS Catal.* **2018**, *8,* 6699-6706.<br>**1a** (0.2 mmol), styrene (0.6 mmol), [Rh(OAc)(cod)]$_2$ (5.0 mol%), 2,3-difluorobenzoic acid (1.0 equiv.), PhMe, 160 °C, 24.	NR
21. *J. Am. Chem. Soc.,* **2002**, *124,* 1586-1587.<br>**1a** (0.2 mmol), styrene (0.22 mmol), Pd(OAc)$_2$ (2.0 mol%), BQ (1.0 equiv.), TsOH (5.0 equiv.), AcOH/PhMe (1:2, v/v), 20 °C, 16 h.	NR

22. *J. Am. Chem. Soc.*, **2007**, *129*, 7666-7673.<br>**1a** (0.2 mmol), styrene (0.4 mmol), $PdCl_2$ (5.0 mol%), $Cu(OAc)_2$ (1.0 equiv.), TFE/AcOH (4:1, v/v), 80 °C, 48 h.	NR
23. *Angew. Chem., Int. Ed.*, **2009**, *48*, 6511-6515.<br>**1a** (0.2 mmol), styrene (0.4 mmol), $Pd(MeCN)_2Cl_2$ (10 mol%), $Cu(OAc)_2$ (1.0 equiv.), DMA, 110 °C, 18 h.	NR
24. *Org. Lett.*, **2012**, *14*, 728-731.<br>**1a** (0.2 mmol), styrene (0.3 mmol), $[RuCl_2(p\text{-cymene})]_2$ (5.0 mol%), KPF_6 (20 mol%), $Cu(OAc)_2{\cdot}H_2O$ (2.0 equiv.), H_2O, 100 °C, 20 h.	NR
25. *Org. Lett.*, **2012**, *14*, 736-739.<br>**1a** (0.2 mmol), styrene (0.36 mmol), $[RuCl_2(p\text{-cymene})]_2$ (5.0 mol%), NaOAc (30 mol%), MeOH, 60 °C, 24 h.	NR
26. *ACS Catal.*, **2016**, *6*, 230-234.<br>**1a** (0.2 mmol), styrene (0.4 mmol), $[RuCl_2(p\text{-cymene})]_2$ (5.0 mol%), $AgSbF_6$ (20 mol%), AcOH (2.0 equiv.), DCE, 25 °C, 24 h.	NR

NR: No Reaction.

Table S2 Unsuccessful attempts of catalytic direct alkenylation of **1a** with potassium styryltrifluoroborate, styrylboronic acid and cinnamic acid.

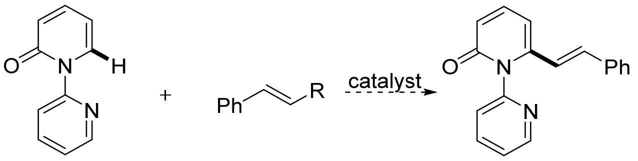

1a **3aa**

Catalytic system	R	Yield (%)
1. *Org. Lett.* **2016**, *18*, 5376-5379.<br>**1a** (0.2 mmol), potassium styryltrifluoroborate (0.6 mmol), $[Cp^*RhCl_2]_2$ (5.0 mol%), $AgSbF_6$ (20 mol%), Ag_2O (1.5 equiv.), DCE, 40 °C, 24 h, under argon.	BF_3K	NR
2. *Org. Biomol. Chem.* **2017**, *15*, 5457-5461.<br>**1a** (0.2 mmol), styrylboronic acid (0.6 mmol), $[RuCl_2(p\text{-cymene})]_2$ (5 mol%), Cu_2O (1 equiv.), AgOTf (1 equiv.), 1,4-Dioxane, 100 °C, 24 h, N_2.	$B(OH)_2$	NR
3. *Chem. Eur. J.* **2014**, *20*, 15000-15004.<br>**1a** (0.2 mmol), cinnamic acid (0.4 mmol), $[Pd(acac)_2]$ (10 mol%), dppe (20 mol%), $CuCO_3$ (3.5 equiv.), DMA/DMSO (8/3), MS 4Å, 140 °C, 12 h.	CO_2H	NR
4. *ACS Catal.* **2017**, *7*, 5363-5369.<br>**1a** (0.1 mmol), cinnamic acid (0.2 mmol), $[Pd(acac)_2]$ (10 mol%), dppe (20 mol%), $CuCO_3{\cdot}Cu(OH)_2$ (3.5 equiv.), DMAc/DMSO (8/3), MS 4Å, 140 °C, 12 h.	CO_2H	NR

NR: No Reaction.

Table S3 Optimization studies for catalytic direct alkenylation of **1a** with cinnamic acid **2a**.[a]

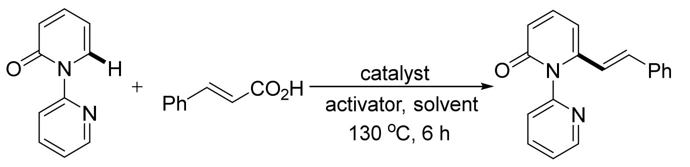

Entry	Catalyst	Activator	Solvent	Yield (%)[b]
**1**	**$[Rh(CO)_2Cl]_2$**	**Boc_2O**	**1,4-dioxane**	**93**
2	$[Rh(CO)_2Cl]_2$	Boc_2O	toluene	15
3	$[Rh(CO)_2Cl]_2$	Boc_2O	PhCl	11
4	$[Rh(CO)_2Cl]_2$	Boc_2O	*p*-xylene	15
5	$[Rh(CO)_2Cl]_2$	Boc_2O	THF	NR
6	$[Rh(CO)_2Cl]_2$	Boc_2O	CH_3CN	NR
7	$[Rh(CO)_2Cl]_2$	Boc_2O	DMSO	NR
8	$[Rh(CO)_2Cl]_2$	Boc_2O	DCE	10
9	$[Rh(CO)_2Cl]_2$	Boc_2O	acetone	NR
10	$[Rh(CO)_2Cl]_2$	Boc_2O	MeOH	NR
11	$[Rh(CO)_2Cl]_2$	Boc_2O	*i*PrOH	NR
12	$[Rh(CO)_2Cl]_2$	Boc_2O	*t*AmOH	NR
13	$[Rh(CO)_2Cl]_2$	Boc_2O	DMF	NR
14	$[Rh(CO)_2Cl]_2$	Boc_2O	DMA	NR
15	$[Rh(CO)_2Cl]_2$	Boc_2O	DME	NR
16[c]	$[Rh(CO)_2Cl]_2$	Boc_2O	1,4-dioxane	52
17[d]	$[Rh(CO)_2Cl]_2$	Boc_2O	1,4-dioxane	81
18[e]	$[Rh(CO)_2Cl]_2$	Boc_2O	1,4-dioxane	91
19	$[Rh(COD)Cl]_2$	Boc_2O	1,4-dioxane	<5
20	$[RhCl(PPh_3)_3]$	Boc_2O	1,4-dioxane	NR
21	$[Rh(CO)_2acac]$	Boc_2O	1,4-dioxane	NR
22	$[Rh(C_2H_4)_2Cl]_2$	Boc_2O	1,4-dioxane	14
23	$[RhCl(NBD)]_2$	Boc_2O	1,4-dioxane	15
24	$[RhCl(1,5\text{-}HD)]_2$	Boc_2O	1,4-dioxane	11
25	$[Rh(COD)_2]BF_4$	Boc_2O	1,4-dioxane	NR
26	$RhCl_3.\ xH_2O$	Boc_2O	1,4-dioxane	NR
27	$[Ru(p\text{-}cymene)_2Cl_2]_2$	Boc_2O	1,4-dioxane	NR
28	$[RuCl_2(PPh_3)_3]$	Boc_2O	1,4-dioxane	NR
29	$[Cp^*IrCl_2]_2$	Boc_2O	1,4-dioxane	NR
30	$[IrCl(COD)]_2$	Boc_2O	1,4-dioxane	NR
31	$[Cp^*RhCl_2]_2$	Boc_2O	1,4-dioxane	NR
32	$[Cp^*Rh(MeCN)_3](SbF_6)_2$	Boc_2O	1,4-dioxane	NR
33	$Pd(OAc)_2$	Boc_2O	1,4-dioxane	NR
34	$PdCl_2$	Boc_2O	1,4-dioxane	NR
35	$[Rh(CO)_2Cl]_2$	$(MeOCO)_2O$	1,4-dioxane	22
36	$[Rh(CO)_2Cl]_2$	Tf_2O	1,4-dioxane	NR
37	$[Rh(CO)_2Cl]_2$	$(CF_3CO)_2O$	1,4-dioxane	NR
38	$[Rh(CO)_2Cl]_2$	PivCl	1,4-dioxane	39

39	$[Rh(CO)_2Cl]_2$	Piv_2O	1,4-dioxane	92
40[f]	$[Rh(CO)_2Cl]_2$	Boc_2O	1,4-dioxane	55
41[g]	$[Rh(CO)_2Cl]_2$	Boc_2O	1,4-dioxane	43
42	$[Rh(CO)_2Cl]_2$	none	1,4-dioxane	NR
43	none	Boc_2O	1,4-dioxane	NR

[a]Reaction Conditions: **1a** (0.2 mmol), **2a** (0.22 mmol), $[Rh(CO)_2Cl]_2$ (1.0 mol%), activator (1.5 equiv.), solvent (2.0 mL), 130 °C, 6 h, in air. NR: no reaction. [b]Isolated yield. [c]Boc_2O (1.0 equiv.). [d]Boc_2O (1.2 equiv.). [e]Boc_2O (2.0 equiv.). [f]Reaction temperature 120 °C. [g]$[Rh(CO)_2Cl]_2$ (0.5 mol %) was employed.

Table S4 Effect of the N-directing groups.[a]

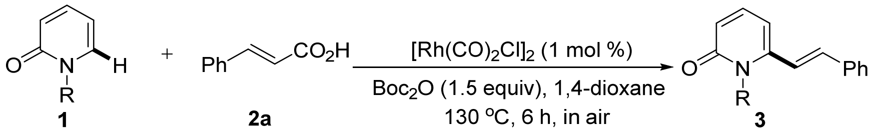

**Entry**	**R**	**Yield** (%)[b]
1	Me	NR
2	Et	NR
3	Bn	NR
4	Ph	NR
5	acetyl	NR
6	**2-pyridyl**	**93** (**3aa**)
**7**	3-pyridyl	NR
8	2-pyrimidyl	31 (**3a'a**)
9	H	NR

[a]Reaction Conditions: **1** (0.2 mmol), **2a** (0.22 mmol), $[Rh(CO)_2Cl]_2$ (1.0 mol%), Boc_2O (1.5 equiv.), 1,4-dioxane (2.0 mL), 130 °C, 6 h, in air. [b]Isolated yield.

(*E*)-1-(Pyrimidin-2-yl)-6-styrylpyridin-2(1H)-one (3a'a)

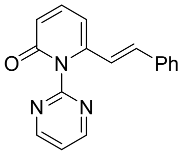

Yellow oil, 17.1 mg, 31%; ^1H NMR (400 MHz, $CDCl_3$) δ 8.97 (d, *J* = 4.9 Hz, 2H), 7.47 (dd, *J* = 10.4, 5.6 Hz, 2H), 7.34-7.15 (m, 5H), 7.07 (d, *J* = 15.9 Hz, 1H), 6.61 (dd, *J* = 18.6, 8.1 Hz, 2H), 6.15 (d, *J* = 15.9 Hz, 1H); $^{13}C\{^1H\}$ NMR (101 MHz, $CDCl_3$) δ 163.13, 159.76, 150.27, 145.51, 140.27, 135.24, 129.11, 128.79, 128.23, 127.07, 125.31, 121.13, 119.86, 103.84; HRMS (ESI) calcd. for $C_{17}H_{13}N_3O$ $[M+H]^+$: 276.1131, found: 276.1139.

3. Synthesis of substrates

(a) Synthesis of 1-(pyrimidin-2-yl)pyridin-2(1*H*)-one (1a')

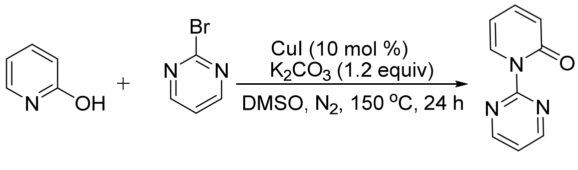

To a round-bottom flask were added sequentially 2-hydroxypyridine (475.5 mg, 5.0 mmol), CuI (96 mg, 10 mol%), K_2CO_3 (829.2 mg, 6.0 mmol), 2-bromopyrimidine

(953.9 mg, 6 mmol) and DMSO (20 mL). The mixture was stirred at 150 °C for 12 h under nitrogen atmosphere. The resulting mixture was allowed to cool to room temperature and then quenched with water (20 mL). Extraction with ethyl acetate (15 mL × 3), concentration under reduced pressure, and silica gel column purification with hexanes/ethyl acetate (1:1) afforded pure 1-(pyrimidin-2-yl)pyridin-2(1*H*)-one.

1-(Pyrimidin-2-yl)pyridin-2(1*H*)-one (1a'). Brown oil, 303.1 mg, 35%, ^{1}H NMR (400 MHz, $CDCl_3$) δ 8.91 (d, *J* = 4.9 Hz, 2H), 7.64 (dd, *J* = 7.1, 2.1 Hz, 1H), 7.46-7.35 (m, 2H), 6.69 (dt, *J* = 9.3, 1.0 Hz, 1H), 6.28 (td, *J* = 6.8, 1.3 Hz, 1H); $^{13}C\{^1H\}$ NMR (101 MHz, $CDCl_3$) δ 161.95, 159.34, 159.12, 140.31, 135.81, 122.71, 120.49, 105.99; HRMS (ESI) calcd. for $C_9H_7N_3O$ $[M+H]^+$: 174.0662, found: 174.0669.

(b) Synthesis of estrone alkenyl acid (2z)

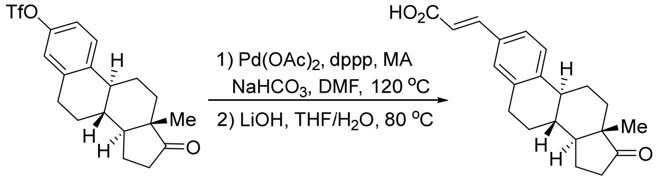

2Z, 56%

A mixture of estrone triflate[8] (1.21 g, 3.0 mmol), methyl acrylate (MA) (310 mg, 3.6 mmol), $Pd(OAc)_2$ (67 mg, 10% mmol), 1,3-bis(diphenylphosphino) propane (dppp) (99 mg, 8% mmol), and $NaHCO_3$ (504 mg, 6.0 mmol) in anhydrous DMF (20 mL) was stirred at 120 °C for 24 h under N_2. Then the mixture was cooled to room temperature, poured into brine (20 mL) and extracted with diethyl ether (10 mL × 3). The combined organic layer was dried over Na_2SO_4 and evaporated under vacuum, and the crude residual was purified by chromatography on silica gel column to afford the methyl ester in 78% yield.

A mixture of the above obtained methyl ester (791.9 mg, 2.34 mmol) and LiOH (280.2 mg, 5 equiv.) in THF/H_2O (1:1, 20mL) was stirred at 80 °C in an oil bath. The progress of reaction was monitored with TLC. After consumption of the starting material, the reaction mixture was diluted with HCl (3M, 15 mL), and the reaction mixture was extracted with ethyl acetate (15 mL × 3). The organic extracts were combined, dried over Na_2SO_4, filtered and the volatile materials removed under reduced pressure to afford the desired acid.

Estrone acrylic acid (2z). White solid, 408.1 mg, 56%, mp: 82.3~85.1 °C, ^{1}H NMR (400 MHz, $CDCl_3$) δ 7.77 (d, J = 15.9 Hz, 1H), 7.43-7.29 (m, 3H), 6.44 (d, J = 16.0 Hz, 1H), 2.97 (dd, J = 9.6, 4.7 Hz, 2H), 2.65-2.28 (m, 3H), 2.26-1.96 (m, 4H), 1.76-1.43 (m, 6H), 0.95 (s, 3H); $^{13}C\{^1H\}$ NMR (101 MHz, $CDCl_3$) δ 220.79, 172.27, 146.98, 143.08, 137.25, 131.68, 129.11, 126.04, 125.78, 116.49, 50.53, 47.98, 44.65, 37.98, 35.85, 31.57, 29.29, 26.34, 25.64, 21.61, 13.85; HRMS (ESI) calcd. for $C_{21}H_{24}O_3$ $[M+H]^+$: 325.1798, found: 325.1791.

4. The general procedure for directed alkenylation of 2-pyridones

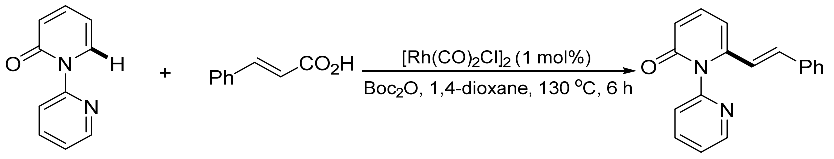

1a **2a** **3aa**

To an oven-dried pressure tube were sequentially added **1a** (34.6 mg, 0.2 mmol), $[Rh(CO)_2Cl]_2$ (0.8 mg, 1.0 mol%), **2a** (32.6 mg, 0.22 mmol), Boc_2O (65.5 mg, 0.3 mmol) and 1,4-dioxane (2.0 mL). The tube was sealed, and the reaction mixture was heated and stirred vigorously at 130 °C for 6 h in an oil bath under air atmosphere. The reaction tube was removed from the oil bath and cooled to room temperature. The reaction mixture was washed with saturated sodium bicarbonate solution (5 mL) and extracted with CH_2Cl_2 (5 mL × 3). The combined organic layer was dried over Na_2SO_4, filtered and evaporated under vacuum. The crude residue was purified by column chromatography on silica gel using a mixture of ethyl acetate and hexane to give the purified product.

5. Synthetic applications

(a) Gram-scale synthesis of **3aa**

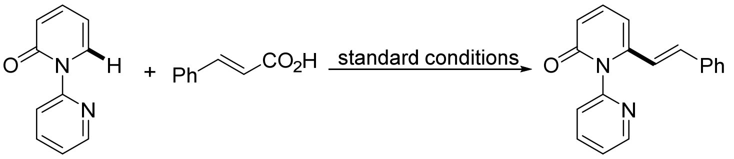

1a, 6 mmol **2a**, 6.6 mmol **3aa**, 88% yield

To a 100 mL round-bottom flask were added **1a** (1.03 g, 6.0 mmol), $[Rh(CO)_2Cl]_2$ (23.4 mg, 1.0 mol%), **2a** (978.0 mg, 6.6 mmol), Boc_2O (1.96 g, 9.0 mmol) and 1,4-dioxane (25 mL). The flask was sealed, and the reaction mixture was heated and stirred vigorously at 130 °C for 6 h in the oil bath under air atmosphere. The flask was next removed from the oil bath and cooled to room temperature. The mixture was washed with saturated sodium bicarbonate solution (50 mL) and extracted with CH_2Cl_2 (25 mL × 3). The combined organic layer was dried over Na_2SO_4, filtered and evaporated under vacuum. The crude residue was purified by column chromatography on silica gel using a mixture of ethyl acetate and hexanes (hexanes/ethyl acetate = 1/2) to give pure **3aa** (1.45g, 88% yield).

(b) Hydrogenation of **3aa**

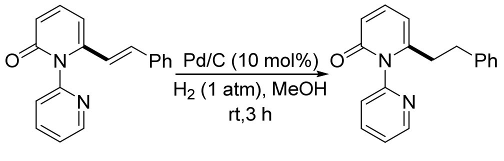

3aa **6**, 84%

To a 25 mL Schlenk flask charged with **3aa** (137.2 mg, 0.5 mmol) was added Pd/C (106.5 mg (5 wt % Pd on charcoal), 10 mol%) and absolute methanol (5 mL). The flask was evacuated and refilled with hydrogen three times. The reaction mixture was stirred at room temperature under H_2 (1 atm) for 3 h, filtered through a pad of celite and

concentrated by evaporation. The crude residue was purified by flash column chromatography on silica gel using a mixture of ethyl acetate and hexane (hexanes/ethyl acetate = 1/1) to give the target product as brown oil (116.0 mg, 84%).

6-phenethyl-2H-[1,2'-bipyridin]-2-one (6), brown oil, 116.0 mg, 84%; ^{1}H NMR (400 MHz, $CDCl_3$) δ 8.71 (dd, *J* = 4.9, 1.9 Hz, 1H), 7.91 (td, *J* = 7.7, 2.0 Hz, 1H), 7.49-7.30 (m, 3H), 7.27-7.11 (m, 3H), 6.98-6.91 (m, 2H), 6.57 (d, *J* = 9.3 Hz, 1H), 6.14 (d, *J* = 6.9 Hz, 1H), 2.78 (s, 2H), 2.55 (t, *J* = 8.1 Hz, 2H); ^{13}C{^{1}H} NMR (101 MHz, $CDCl_3$) δ 163.82, 151.72, 149.85, 148.86, 140.07, 140.03, 138.56, 128.50, 128.17, 126.35, 124.13, 124.12, 118.96, 105.44, 34.56, 29.70; HRMS (ESI) calcd. for $C_{18}H_{16}N_2O$ $[M+H]^+$: 277.1335, found: 277.1328.

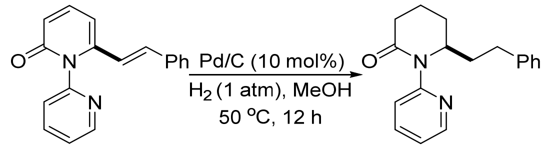

3aa **7**, 92%

To a 25 mL Schlenk flask charged with **3aa** (137.2 mg, 0.5 mmol) was added Pd/C (106.5 mg (5 wt % Pd on charcoal), 10 mol%) and absolute methanol (5 mL). The flask was evacuated and refilled with hydrogen three times. The reaction mixture was stirred at 50 °C under H_2 (1 atm) for 12 h, and then filtered through a pad of celite and concentrated by evaporation. The crude residue was purified by flash column chromatography on silica gel using a mixture of ethyl acetate and hexane (hexanes/ethyl acetate = 5/1) to give the target product as yellow oil (128.9 mg, 92%).

6-phenethyl-1-(pyridin-2-yl)piperidin-2-one (7), yellow oil, 128.9 mg, 92%; ^{1}H NMR (400 MHz, $CDCl_3$) δ 8.57-8.39 (m, 1H), 7.72 (td, *J* = 7.8, 2.0 Hz, 1H), 7.56 (d, *J* = 8.1 Hz, 1H), 7.36-7.09 (m, 4H), 7.03 (d, *J* = 7.4 Hz, 2H), 4.69 (dq, *J* = 9.8, 5.1 Hz, 1H), 2.76-2.42 (m, 4H), 2.22-1.64 (m, 6H); ^{13}C{^{1}H} NMR (101 MHz, $CDCl_3$) δ 170.74, 148.36, 141.19, 137.05, 128.36, 128.12, 125.95, 123.32, 121.28, 56.21, 35.23, 32.04, 29.69, 26.62, 17.68; HRMS (ESI) calcd. for $C_{18}H_{20}N_2O$ $[M+H]^+$: 281.1648, found: 281.1657.

(c) Deprotection of the alkenylated products

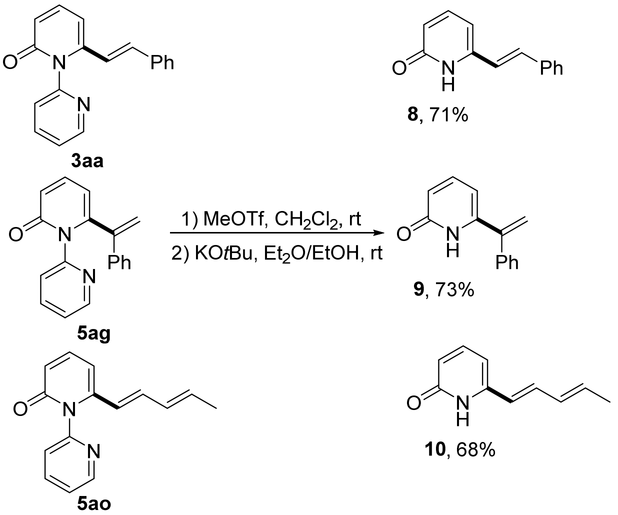

The 2-pyridone product (**3aa**, 82.3 mg; **5ag**, 82.3 mg; **5ao**, 71.5 mg; 0.3 mmol) and CH_2Cl_2 (3.0 mL) were placed in an oven-dried pressure tube under an N_2 atmosphere. The tube was capped with a rubber septum, cooled to 0 °C in an ice water bath, and methyl trifluoromethanesulfonate (54.2 mg, 0.33 mmol) was then added via syringe. The mixture was allowed to warm to room temperature and then stirred for 24 h. After evaporation of all volatile materials in vacuo, potassium *tert*-butoxide (101.0 mg, 0.9 mmol), EtOH (0.6 mL) and Et_2O (2.4 mL) were added sequentially under N_2, and the resulting suspension was stirred for 4 h at room temperature. The reaction mixture was then washed with water (10 mL) and extracted with CH_2Cl_2 (5 mL × 3). The combined organic layer was washed with brine (10 mL), dried over Na_2SO_4, filtered, and concentrated under reduced pressure. The crude residue was purified by flash chromatography to afford **8, 9,** and **10** as oils in 71%, 73%, 68% yields, respectively (**8**, 42.0 mg, 71%; **9**, 43.2 mg, 73%; **10**, 32.9 mg, 68%).

(*E*)-6-styrylpyridin-2(1H)-one (8),

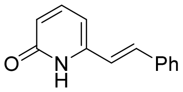

Brown oil, 42.0 mg, 71%; ^{1}H NMR (400 MHz, $CDCl_3$) δ 12.34 (s, 1H), 7.64-7.51 (m, 3H), 7.51-7.31 (m, 4H), 6.83 (d, *J* = 16.5 Hz, 1H), 6.55 (dd, *J* = 9.1, 0.9 Hz, 1H), 6.37 (d, *J* = 7.0 Hz, 1H); $^{13}C\{^1H\}$ NMR (101 MHz, $CDCl_3$) δ 164.07, 144.02, 141.33, 135.79, 133.55, 129.01, 128.82, 127.28, 120.56, 119.06, 106.49; HRMS (ESI) calcd. for $C_{13}H_{11}NO$ $[M+H]^+$: 198.0913, found: 198.0921.

6-(1-phenylvinyl)pyridin-2(1H)-one (9),

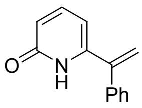

Brown oil, 43.2 mg, 73%; ^{1}H NMR (400 MHz, $CDCl_3$) δ 11.45 (s, 1H), 7.37 (td, *J* = 6.8, 3.6 Hz, 6H), 6.53 (dd, *J* = 9.2, 1.0 Hz, 1H), 6.11 (dd, *J* = 6.9, 0.9 Hz, 1H), 5.97 (s, 1H), 5.66 (s, 1H); $^{13}C\{^1H\}$ NMR (101 MHz, $CDCl_3$) δ 164.51, 146.05, 143.10, 140.96, 138.33, 128.65, 128.57, 128.54, 119.87, 118.59, 107.04; HRMS (ESI) calcd. for $C_{13}H_{11}NO$ $[M+H]^+$: 198.0913, found: 198.0921.

6-((1E,3E)-penta-1,3-dien-1-yl)pyridin-2(1H)-one (10),

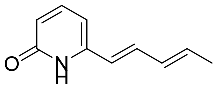

Yellow oil, 32.9 mg, 68%; ^{1}H NMR (400 MHz, $CDCl_3$) δ 12.35 (s, 1H), 7.39 (dd, *J* = 9.0, 7.0 Hz, 1H), 7.12 (dd, *J* = 15.9, 10.4 Hz, 1H), 6.45 (dd, *J* = 9.1, 0.9 Hz, 1H), 6.31-6.13 (m, 3H), 6.06 (dq, *J* = 13.9, 6.8 Hz, 1H), 1.95-1.83 (m, 3H); $^{13}C\{^1H\}$ NMR (101 MHz, $CDCl_3$) δ 165.08, 144.58, 141.27, 135.25, 134.39, 130.97, 121.47, 118.09, 105.23, 18.58; HRMS (ESI) calcd. for $C_{10}H_{11}NO$ $[M+H]^+$: 162.0913, found: 162.0921.

6. The mechanistic studies

a) <u>The H/D exchange experiments</u>

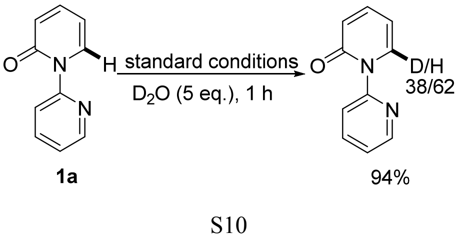

To an oven-dried pressure tube were sequentially added **1a** (51.7 mg, 0.3 mmol), $[Rh(CO)_2Cl]_2$ (1.2 mg, 1.0 mol%), D_2O (30.0 mg, 1.5 mmol), Boc_2O (98.2 mg, 0.45 mmol) and 1,4-dioxane (3.0 mL). The tube was sealed, and the reaction mixture was heated and stirred vigorously at 130 °C for 1 h in an oil bath under air atmosphere. Then the tube was removed from the oil bath and cooled to room temperature. After removal of the volatile materials under reduced pressure, the crude residue was purified by column chromatography on silica gel using a mixture of ethyl acetate and hexane to give an inseparable mixture of **1a** (62%) and [D]-**1a** (38%). The ratio of H/D was determined on the basis of ^1H NMR analysis.

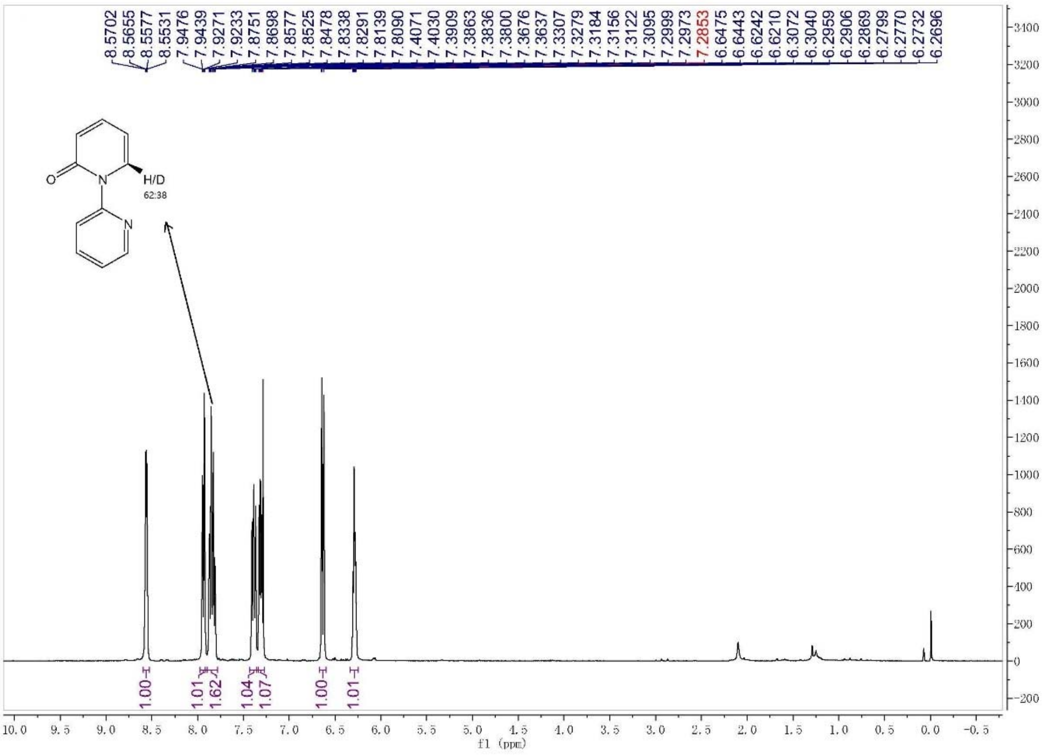

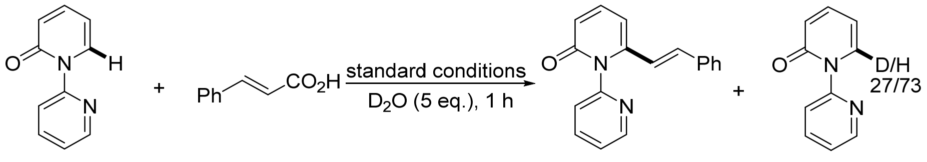

To an oven-dried pressure tube were sequentially added **1a** (51.7 mg, 0.3 mmol), $[Rh(CO)_2Cl]_2$ (1.2 mg, 1.0 mol%), **2a** (48.9 mg, 0.33 mmol), D_2O (30.0 mg, 1.5 mmol), Boc_2O (98.2 mg, 0.45 mmol) and 1,4-dioxane (3.0 mL). The tube was sealed, and the reaction mixture was heated and stirred vigorously at 130 °C for 1 h in the oil bath under air atmosphere. Then the tube was removed from the oil bath and cooled to room temperature. The mixture was washed with saturated sodium bicarbonate solution (15 mL) and extracted with CH_2Cl_2 (5 mL x 3). The combined organic layer was dried over Na_2SO_4 and filtered. The volatile materials were removed under reduced pressure and the crude residue was purified by column chromatography on silica gel using a mixture of ethyl acetate and hexane (hexanes/ethyl acetate = 1/2) to give **3aa** (32.1 mg, 39%) and an inseparable

mixture of **1a** and [D]-**1a** (27.7 mg, 53%). The ratio of H/D was determined on the basis of ^{1}H NMR analysis.

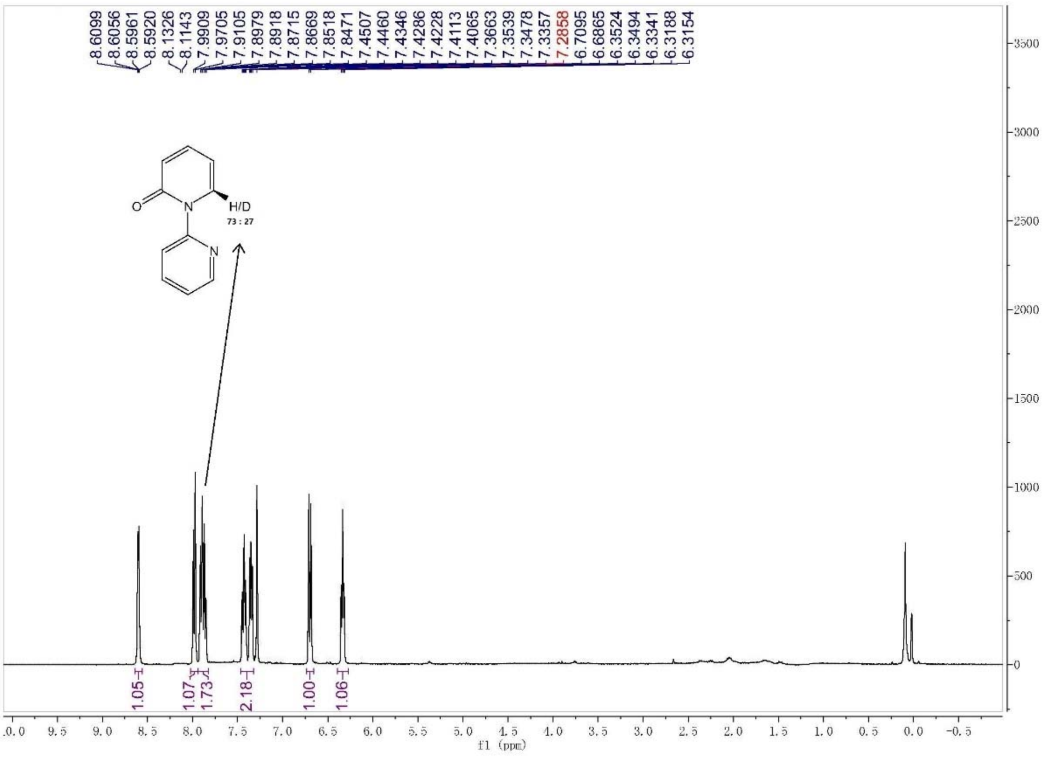

b) <u>The coupling reaction of anhydride **11** with **1a**</u>

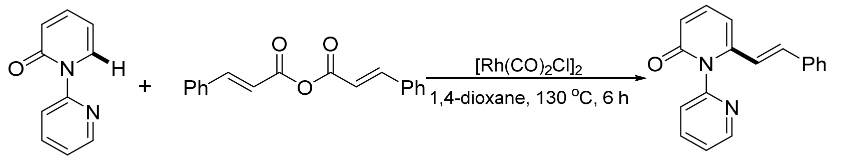

1a **11** **3aa,** 91%

To an oven-dried pressure tube were sequentially added **1a** (51.7 mg, 0.3 mmol), $[Rh(CO)_2Cl]_2$ (1.2 mg, 1.0 mol%), **11** (91.8 mg, 0.33 mmol) and 1,4-dioxane (3.0 mL). The tube was sealed, and the reaction mixture was heated and stirred vigorously at 130 °C in an oil bath. After 6 h, the tube was removed from the oil bath, and cooled to room temperature. The volatiles were removed, and the crude residual was purified by column chromatography on silica gel using a mixture of ethyl acetate and hexane (hexanes/ethyl acetate = 1/2) to give pure **3aa** (74.9 mg, 91%).

c) <u>The in situ generation of anhydride **11**</u>

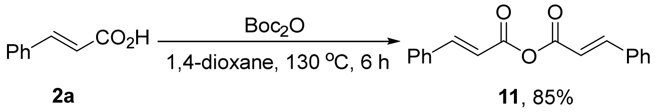

2a **11**, 85%

A mixture of equimolar amounts of **2a** (32.6 mg, 0.22 mmol) and Boc_2O (65.5 mg, 0.3 mmol) in 1,4-dioxane (2.0 mL) was stirred at 130 °C for 6 h. After cooling to room temperature, the volatile materials were removed under reduced pressure and the crude residual was purified by column chromatography on silica gel using a mixture of ethyl

acetate and hexane (hexanes/ethyl acetate = 2/1) to give cinnamic anhydride **11** (52.0 mg, 85%).

Cinnamic anhydride (11). White solid, 52.0 mg, 85%, mp: 135.4~137.8 °C; ^{1}H NMR (400 MHz, $CDCl_3$) δ 7.89 (d, *J* = 15.9 Hz, 2H), 7.60 (dd, *J* = 7.5, 2.1 Hz, 4H), 7.51-7.39 (m, 6H), 6.56 (d, *J* = 15.9 Hz, 2H); $^{13}C\{^{1}H\}$ NMR (126 MHz, $CDCl_3$) δ 162.50, 148.68, 133.74, 131.30, 129.10, 128.60, 116.76; HRMS (ESI) calcd. for $C_{18}H_{14}O_3$ $[M+H]^+$: 279.1016, found: 279.1009.

d) <u>Analysis of the gaseous products by GC-TCD</u>

To an oven-dried pressure tube were sequentially added **1a** (51.7 mg, 0.3 mmol), $[Rh(CO)_2Cl]_2$ (1.2 mg, 1.0 mol%), **2a** (48.9 mg, 0.33 mmol), Boc_2O (98.2 mg, 0.45 mmol) and 1,4-dioxane (3.0 mL). The reaction vessel was degassed and purged with nitrogen three times, then heated and stirred vigorously at 130 °C for 6 h in an oil bath under a nitrogen atmosphere. After cooling to room temperature, the reaction gas was withdrawn with the syringe and then analyzed by GC-TDC with argon as the carrier gas, and the results are shown in Figure S1.

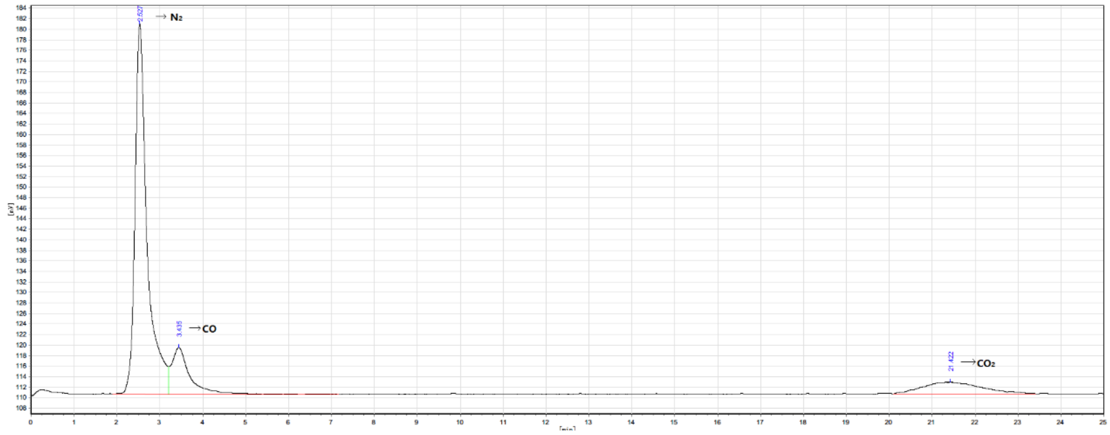

Figure S1. GC analysis indicated the formation of CO and CO_2 with $[Rh(CO)_2Cl]_2$ as the catalyst.

To an oven-dried pressure tube were added **1a** (51.7 mg, 0.3 mmol), $[Rh(COD)Cl]_2$ (1.5 mg, 1.0 mol%), **2a** (48.9 mg, 0.33 mmol), Boc_2O (98.2 mg, 0.45 mmol) and 1,4-dioxane (3.0 mL). The reaction vessel was degassed and purged with nitrogen three times, then heated and stirred vigorously at 130 °C for 18 h in an oil bath under a nitrogen atmosphere. After cooling to room temperature, the reaction gas was withdrawn by the syringe and then analyzed by GC-TDC with argon as the carrier gas. As shown in Figure S2, the formation of CO was detected, indicating that the CO is derived from the acid.

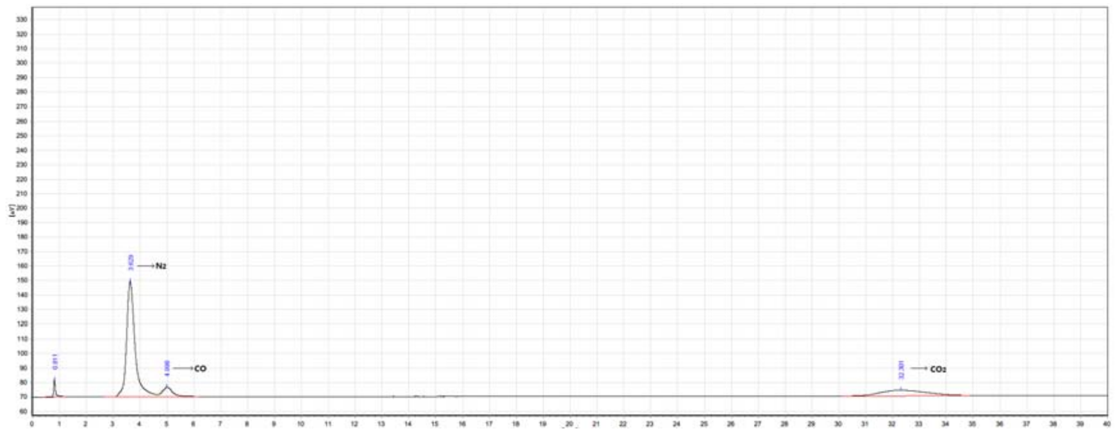

Figure S2. GC analysis indicated the generation of CO and CO_2 with $[Rh(COD)Cl]_2$ as the catalyst precursor

e) <u>Kinetic studies</u>

i)The parallel kinetic isotope effect (KIE) experiments

To an oven-dried pressure tube were sequentially added **1a** (34.6 mg, 0.2 mmol), $[Rh(CO)_2Cl]_2$ (0.8 mg, 1.0 mol%), **2a** (32.6 mg, 0.22 mmol), Boc_2O (65.5 mg, 0.3 mmol) and 1,4-dioxane (2.0 mL). In another pressure tube, deuterium-labeled compound [D_1]-**1a** (34.6 mg, 0.2 mmol) was used instead of **1a**. The two tubes were sealed, and the two reaction mixtures were heated and stirred vigorously at 130 °C under air atmosphere. An aliquot of each reaction mixture was taken at the time of 10 min, 20 min, 30 min, 40 min and 50 min. The conversions were determined by ^{1}H NMR analysis of the crude reaction mixtures. A value of $k_H/k_D = 1.9 \pm 0.1$ was obtained. The deuterium-labeled compound [D_1]-**1a** was prepared according to the reported procedure.[9]

**Time (min)**		10	20	30	40	50
**NMR**	**3aa**	3.6	10.6	15.1	27.5	33.8
**Yield** (%)	**[D]-3aa**	1.5	5.2	10.4	13.9	17.4

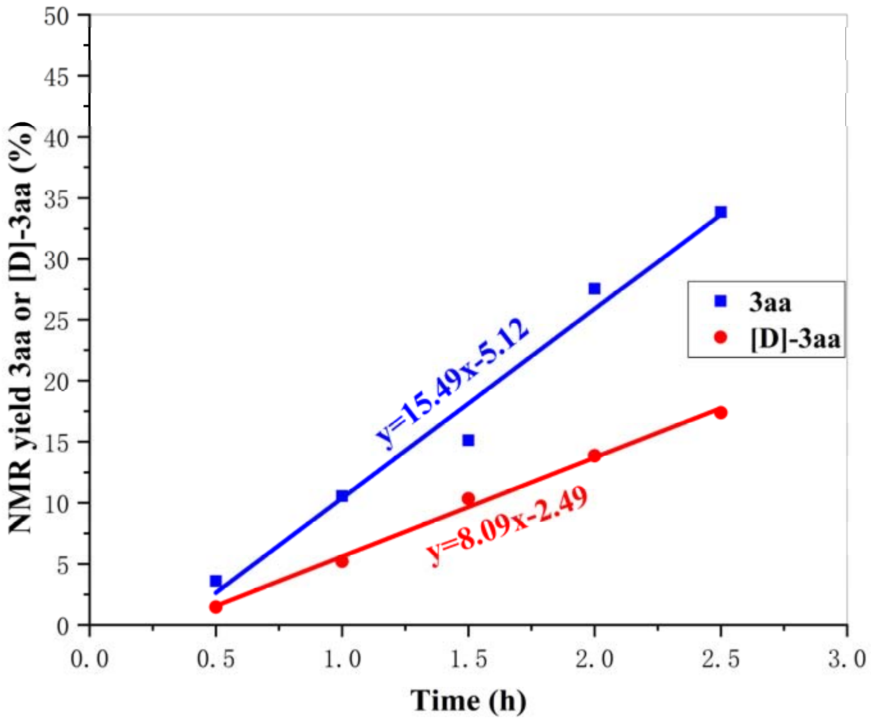

ii) Determination of the reaction order with respect to the concentration of **1a**

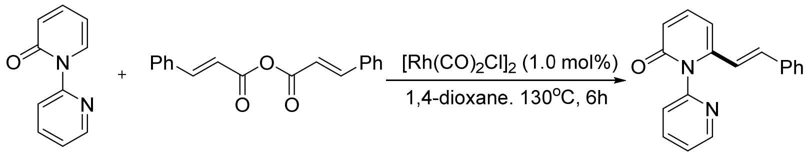

The reaction order was examined using the initial rate method. A suspension of pyridone **1a** (0.05, 0.1, 0.15, 0.2 and 0.25 mmol), cinnamic anhydride **11** (3.06g, 11 mmol, 20 equiv.) and $[Rh(CO)_2Cl]_2$ (0.8 mg, 1.0%) was heated at 130 °C in 1,4-dioxane (3 mL). Aliquots (25 μL) were removed at 10, 20, 30, 40 and 50 min by a syringe (up to ca. 10−20% conversion) and directly analyzed by NMR using *1,2-dibromoethane* as the internal standard.

Table S5. Reaction order in [**1a**].

Entry	c(1a)/mol·L^{-1}	k/mol·L^{-1}S^{-1}	log(c/mol·L^{-1})	log(k/mol·L^{-1}S^{-1})
1	0.05	0.0014±7.211E-4	-1.301	-2.854
2	0.10	0.0042±3.055E-4	-1.000	-2.377
3	0.15	0.0064±0.0021	-0.824	-2.194
4	0.20	0.0080±0.0034	-0.699	-2.097
5	0.25	0.0124±0.0010	-0.602	-1.907

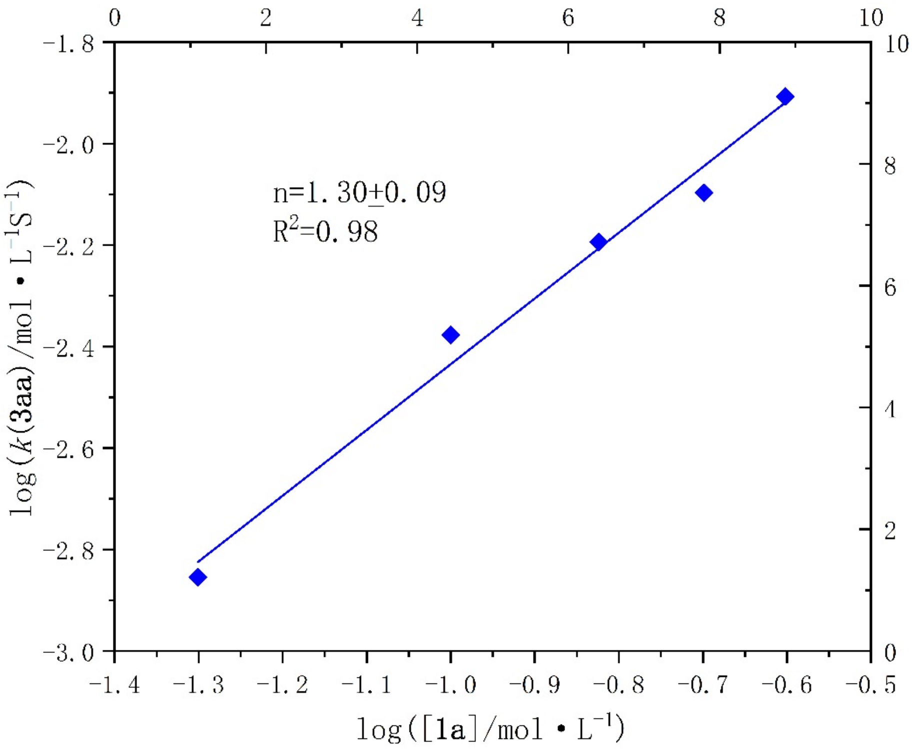

Figure S3. Reaction order in [**1a**].

7. Characterization data for products

(*E*)-6-styryl-2H-[1,2'-bipyridin]-2-one (3aa)

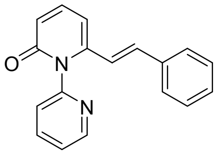

Yellow solid, 51.0 mg, 93%, mp: 102.3~133.7 °C; ^{1}H NMR (400 MHz, $CDCl_3$) δ 8.72 (ddd, *J* = 4.9, 2.0, 0.9 Hz, 1H), 7.94 (td, *J* = 7.7, 1.9 Hz, 1H), 7.50-7.38 (m, 3H), 7.29 (d, *J* = 7.5 Hz, 2H), 7.20 (dd, *J* = 7.4, 2.3 Hz, 2H), 7.04 (d, *J* = 16.0 Hz, 1H), 6.67-6.58 (m, 2H), 6.20 (d, *J* = 16.0 Hz, 1H); ^{13}C{^{1}H} NMR (101 MHz, $CDCl_3$) δ 163.24, 151.72, 150.03, 146.35, 139.96, 138.80, 135.66, 134.32, 128.97, 128.77, 127.01, 124.20, 124.13, 120.86, 119.80, 103.97; HRMS (ESI) calcd. for $C_{18}H_{14}N_2O$ [M+H]$^+$: 275.1179, found: 275.1186.

(*E*)-6-(4-methylstyryl)-2H-[1,2'-bipyridin]-2-one (3ab)

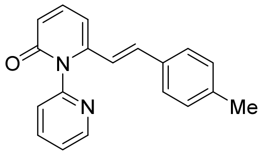

Yellow solid, 53.1 mg, 92%, mp: 108.4~109.8 °C; ^{1}H NMR (400 MHz, $CDCl_3$) δ 8.71 (ddd, *J* = 4.9, 1.9, 0.9 Hz, 1H), 7.93 (td, *J* = 7.7, 1.9 Hz, 1H), 7.49-7.35 (m, 3H), 7.10 (s, 4H), 7.02 (d, *J* = 15.9 Hz, 1H), 6.65-6.56 (m, 2H), 6.14 (d, *J* = 15.9 Hz, 1H), 2.33 (s, 3H); ^{13}C{^{1}H} NMR (101 MHz, $CDCl_3$) δ 163.27, 151.80, 150.00, 146.58, 139.98, 139.20, 138.77, 134.34, 132.94, 129.49, 126.97, 124.20, 124.09, 119.84, 119.48, 103.75, 21.31; HRMS (ESI) calcd. for $C_{19}H_{16}N_2O$ [M+H]$^+$: 289.1335, found: 289.1342.

(*E*)-6-(4-methoxystyryl)-2H-[1,2'-bipyridin]-2-one (3ac)

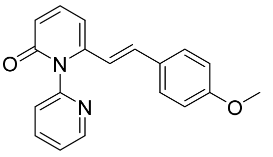

Yellow solid, 55.4 mg, 91%, mp: 111.4~112.6 °C; ^{1}H NMR (400 MHz, Chloroform-*d*) δ 8.67 (ddd, *J* = 4.9, 1.9, 0.8 Hz, 1H), 7.89 (td, *J* = 7.7, 1.9 Hz, 1H), 7.46-7.30 (m, 3H), 7.15-7.06 (m, 2H), 6.96 (d, *J* = 16.0 Hz, 1H), 6.84-6.72 (m, 2H), 6.60-6.51 (m, 2H), 6.00 (d, *J* = 15.9 Hz, 1H), 3.75 (s, 3H); ^{13}C{^{1}H} NMR (101 MHz, $CDCl_3$) δ 163.32, 160.31, 151.84, 150.00, 146.74, 140.04, 138.80, 133.99, 128.45, 124.21, 124.09, 119.15, 118.52, 114.22, 103.49, 55.33; HRMS (ESI) calcd. for $C_{19}H_{16}N_2O_2$ $[M+H]^+$: 305.1285, found: 305.1293.

(*E*)-6-(4-hydroxystyryl)-2H-[1,2'-bipyridin]-2-one (3ad)

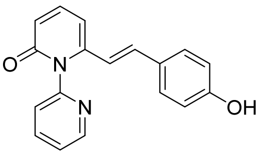

Yellow oil, 50.5 mg, 87%; ^{1}H NMR (400 MHz, $CDCl_3$) δ 8.93 (s, 1H), 8.66 (ddd, *J* = 5.0, 2.0, 0.9 Hz, 1H), 7.90 (td, *J* = 7.8, 2.0 Hz, 1H), 7.51-7.32 (m, 3H), 7.03-6.90 (m, 3H), 6.68-6.53 (m, 4H), 5.93 (d, *J* = 15.9 Hz, 1H); ^{13}C{^{1}H} NMR (101 MHz, $CDCl_3$) δ 163.83, 158.59, 151.50, 149.96, 147.25, 140.89, 139.11, 135.31, 128.60, 126.87, 124.39, 124.11, 118.12, 116.86, 116.00, 104.13; HRMS (ESI) calcd. for $C_{18}H_{14}N_2O_2$ $[M+H]^+$: 291.1128, found: 291.1135.

(*E*)-(4-(2-(2-oxo-2H-[1,2'-bipyridin]-6-yl)vinyl)phenyl)boronic acid (3ae)

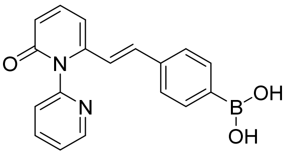

Yellow solid, 54.1 mg, 85%, mp: 119.5~121.6 °C; ^{1}H NMR (400 MHz, $CDCl_3$) δ 8.72 (dd, *J* = 5.2, 1.9 Hz, 1H), 7.94 (td, *J* = 7.7, 2.0 Hz, 1H), 7.50-7.38 (m, 3H), 7.33-7.25 (m, 2H), 7.21 (dd, *J* = 7.4, 2.3 Hz, 2H), 7.05 (d, *J* = 16.0 Hz, 1H), 6.71-6.56 (m, 2H), 6.20 (d, *J* = 16.0 Hz, 1H); ^{13}C{^{1}H} NMR (101 MHz, $CDCl_3$) δ 163.23, 151.72, 150.04, 146.35, 139.99, 138.83, 135.65, 134.34, 128.98, 128.78, 127.02, 124.20, 120.83, 119.78, 103.98; HRMS (ESI) calcd. for $C_{18}H_{15}BN_2O_3$ $[M+H]^+$: 319.1248, found: 319.1240.

(*E*)-6-(4-(dimethylamino)styryl)-2H-[1,2'-bipyridin]-2-one (3af)

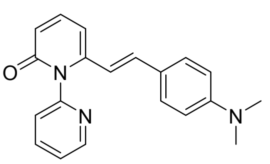

Yellow oil, 52.7 mg, 83%; ^{1}H NMR (400 MHz, $CDCl_3$) δ 8.73 (ddd, *J* = 4.9, 2.0, 0.8 Hz, 1H), 7.93 (td, *J* = 7.7, 1.9 Hz, 1H), 7.49-7.33 (m, 3H), 7.14-7.06 (m, 2H), 6.99 (d, *J* = 15.8 Hz, 1H), 6.65-6.52 (m, 4H), 5.94 (d, *J* = 15.9 Hz, 1H), 2.98 (s, 6H); ^{13}C{^{1}H} NMR (101 MHz, $CDCl_3$) δ 163.71, 152.10, 150.21, 149.69, 146.60, 140.11, 138.20, 134.24, 130.25, 128.40, 123.83, 123.46, 119.26, 118.36, 111.68, 106.90, 40.20; HRMS (ESI) calcd. for $C_{20}H_{19}N_3O$ $[M+H]^+$: 318.1601, found: 318.1610.

(*E*)-6-(4-fluorostyryl)-2H-[1,2'-bipyridin]-2-one (3ag)

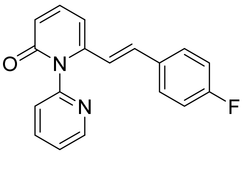

Yellow solid, 47.3 mg, 81%, mp: 112.4~113.9 °C; ^{1}H NMR (400 MHz, $CDCl_3$) δ 8.71 (ddd, *J* = 4.9, 1.9, 0.9 Hz, 1H), 7.94 (td, *J* = 7.7, 1.9 Hz, 1H), 7.50-7.36 (m, 3H), 7.21-7.14 (m, 2H), 7.04-6.92 (m, 3H), 6.69-6.55 (m, 2H), 6.11 (d, *J* = 15.9 Hz, 1H); ^{13}C{^{1}H} NMR (126 MHz, $CDCl_3$) δ 163.99 (d, $J^1_{C\text{-}F}$ = 253.26

Hz), 163.17, 151.71, 150.02, 146.16, 139.92, 138.80, 133.01, 131.92 (d, J^4_{C-F} = 3.78 Hz), 128.71, 128.65, 124.22 (d, J^3_{C-F} = 7.56 Hz), 120.67 (d, J^5_{C-F} = 2.52 Hz), 119.88, 115.93 (d, J^2_{C-F} = 21.42 Hz), 103.89; HRMS (ESI) calcd. for $C_{18}H_{13}FN_2O$ $[M+H]^+$: 293.1085, found: 293.1091.

(*E*)-6-(4-chlorostyryl)-2H-[1,2'-bipyridin]-2-one (3ah)

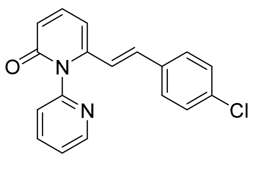

Yellow solid, 51.2 mg, 83%, mp: 189.7~190.4 °C; ^{1}H NMR (400 MHz, $CDCl_3$) δ 8.70 (ddd, *J* = 4.9, 1.9, 0.9 Hz, 1H), 7.93 (td, *J* = 7.7, 1.9 Hz, 1H), 7.50-7.37 (m, 3H), 7.27-7.21 (m, 2H), 7.15-7.07 (m, 2H), 6.97 (d, *J* = 16.0 Hz, 1H), 6.66-6.55 (m, 2H), 6.15 (d, *J* = 16.0 Hz, 1H); ^{13}C{^{1}H} NMR (101 MHz, $CDCl_3$) δ 163.16, 151.59, 150.00, 145.98, 139.95, 138.85, 134.68, 134.15, 132.92, 128.98, 128.15, 124.21, 121.39, 120.01, 104.14; HRMS (ESI) calcd. for $C_{18}H_{13}ClN_2O$ $[M+H]^+$: 309.0789, found: 309.0796.

(*E*)-6-(4-bromostyryl)-2H-[1,2'-bipyridin]-2-one (3ai)

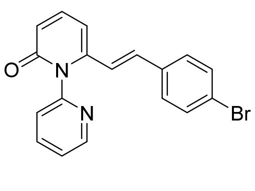

Yellow solid, 62.9 mg, 89%; mp: 203.7~204.4 °C;^{1}H NMR (400 MHz, $CDCl_3$) δ 8.70 (ddd, *J* = 4.9, 2.0, 0.9 Hz, 1H), 7.93 (td, *J* = 7.7, 1.9 Hz, 1H), 7.49-7.31 (m, 5H), 7.09-7.01 (m, 2H), 6.96 (d, *J* = 16.0 Hz, 1H), 6.67-6.56 (m, 2H), 6.17 (d, *J* = 16.0 Hz, 1H); ^{13}C{^{1}H} NMR (101 MHz, $CDCl_3$) δ 163.12, 151.61, 150.01, 145.95, 139.89, 138.82, 134.60, 132.93, 131.94, 128.40, 124.21, 122.93, 121.55, 120.11, 104.12; HRMS (ESI) calcd. for $C_{18}H_{13}BrN_2O$ $[M+H]^+$: 353.0284, found: 353.0292.

(*E*)-6-(4-nitrostyryl)-2H-[1,2'-bipyridin]-2-one (3aj)

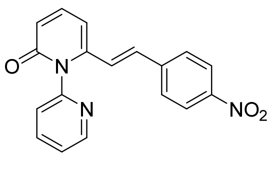

Brown oil, 52.3 mg, 82%; ^{1}H NMR (400 MHz, $CDCl_3$) δ 8.71 (ddd, *J* = 4.9, 1.9, 0.9 Hz, 1H), 8.20-8.08 (m, 2H), 7.96 (td, *J* = 7.7, 1.9 Hz, 1H), 7.53-7.42 (m, 3H), 7.37-7.30 (m, 2H), 7.06 (d, *J* = 16.0 Hz, 1H), 6.71-6.62 (m, 2H), 6.35 (d, *J* = 16.0 Hz, 1H); ^{13}C{^{1}H} NMR (101 MHz, $CDCl_3$) δ 162.94, 151.33, 150.03, 147.51, 145.10, 141.89, 139.77, 138.91, 131.44, 127.48, 125.13, 124.37, 124.26, 124.13, 121.09, 104.92; HRMS (ESI) calcd. for $C_{18}H_{13}N_3O_3$ $[M+H]^+$: 320.1030, found: 320.1038.

(*E*)-6-(4-acetylstyryl)-2H-[1,2'-bipyridin]-2-one (3ak)

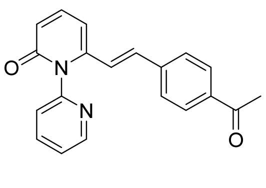

Yellow oil, 49.9 mg, 79%; ^{1}H NMR (400 MHz, $CDCl_3$) δ 8.72 (dd, *J* = 5.3, 1.7 Hz, 1H), 7.96 (td, *J* = 7.7, 2.0 Hz, 1H), 7.91-7.85 (m, 2H), 7.56-7.40 (m, 3H), 7.28 (d, *J* = 8.3 Hz, 2H), 7.06 (d, *J* = 16.0 Hz, 1H), 6.66 (dd, *J* = 10.4, 8.2 Hz, 2H), 6.31 (d, *J* = 16.0 Hz, 1H), 2.58 (s, 3H); ^{13}C{^{1}H} NMR ^{13}C NMR (101 MHz, $CDCl_3$) δ 197.18, 163.04, 151.52, 150.00, 145.66, 140.09, 139.83, 138.83, 136.91, 132.79, 128.81, 127.03, 126.63, 124.24, 123.37, 120.50, 104.47, 26.59; HRMS (ESI) calcd. for $C_{20}H_{16}N_2O_2$ $[M+H]^+$: 317.1285, found: 317.1292.

Methyl (*E*)-4-(2-(2-oxo-2H-[1,2'-bipyridin]-6-yl)vinyl)benzoate (3al)

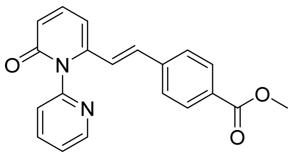

Yellow oil, 51.2 mg, 77%; ^{1}H NMR (400 MHz, $CDCl_3$) δ 8.72 (ddd, *J* = 4.9, 1.9, 0.9 Hz, 1H), 8.00-7.91 (m, 3H), 7.51-7.40 (m, 3H), 7.25 (d, *J* = 8.4 Hz, 2H), 7.05 (d, *J* = 16.0 Hz, 1H), 6.69-6.59 (m, 2H), 6.30 (d, *J* = 16.0 Hz, 1H), 3.91 (s, 3H); ^{13}C{^{1}H} NMR (101 MHz, $CDCl_3$) δ 166.47, 163.08, 151.52, 150.03, 145.71, 139.94, 139.83, 138.83, 132.93, 130.13, 130.02, 126.82, 124.23, 123.20, 120.47, 104.44, 52.19; HRMS (ESI) calcd. for $C_{20}H_{16}N_2O_3$ $[M+H]^+$: 333.1234, found: 333.1241.

(*E*)-4-(2-(2-oxo-2H-[1,2'-bipyridin]-6-yl)vinyl)benzonitrile (3am)

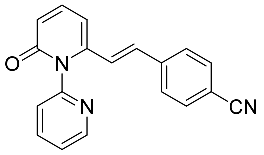

Brown oil, 48.5 mg, 81%; ^{1}H NMR (400 MHz, $CDCl_3$) δ 8.68 (ddd, *J* = 4.9, 2.0, 0.9 Hz, 1H), 7.93 (td, *J* = 7.7, 1.9 Hz, 1H), 7.56-7.51 (m, 2H), 7.48-7.39 (m, 3H), 7.25 (d, *J* = 8.3 Hz, 2H), 7.00 (d, *J* = 16.0 Hz, 1H), 6.68-6.58 (m, 2H), 6.28 (d, *J* = 16.0 Hz, 1H); ^{13}C{^{1}H} NMR (101 MHz, $CDCl_3$) δ 163.01, 151.32, 150.00, 145.24, 139.98, 139.89, 138.94, 132.52, 132.04, 127.37, 124.37, 124.22, 120.76, 118.54, 111.88, 104.84; HRMS (ESI) calcd. for $C_{19}H_{13}N_3O$ $[M+H]^+$: 300.1131, found: 300.1137.

(*E*)-6-(4-(trifluoromethyl)styryl)-2H-[1,2'-bipyridin]-2-one (3an)

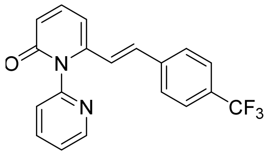

Yellow solid, 54.1 mg, 79%, mp: 120.6~121.5 °C; ^{1}H NMR (400 MHz, $CDCl_3$) δ 8.77-8.67 (m, 1H), 7.95 (td, *J* = 7.7, 1.9 Hz, 1H), 7.53 (d, *J* = 8.1 Hz, 2H), 7.49-7.41 (m, 3H), 7.29 (d, *J* = 8.3 Hz, 2H), 7.04 (d, *J* = 16.0 Hz, 1H), 6.74-6.59 (m, 2H), 6.28 (d, *J* = 16.0 Hz, 1H); ^{13}C{^{1}H} NMR (126 MHz, $CDCl_3$) δ 163.06, 151.51, 150.02, 145.57, 139.84, 139.07, 138.85, 132.47, 130.87 (q, $J^2_{C\text{-}F}$ = 32.76 Hz), 127.12 (q, $J^1_{C\text{-}F}$ = 272.16 Hz), 127.10, 125.77 (q, $J^3_{C\text{-}F}$ = 3.78 Hz), 124.25, 124.23, 123.36, 120.57, 104.49; HRMS (ESI) calcd. for $C_{19}H_{13}F_3N_2O$ $[M+H]^+$: 343.1053, found: 343.1060.

(E)-6-(3-methoxystyryl)-2H-[1,2'-bipyridin]-2-one (3ao)

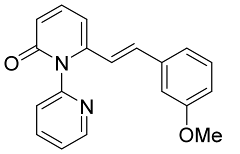

Yellow solid, 54.8 mg, 90%, mp: 111.6~112.5 °C; ^{1}H NMR (400 MHz, $CDCl_3$) δ 8.70 (ddd, *J* = 4.9, 1.9, 0.9 Hz, 1H), 7.92 (td, *J* = 7.7, 1.9 Hz, 1H), 7.52-7.34 (m, 3H), 7.20 (t, *J* = 8.0 Hz, 1H), 7.00 (d, *J* = 16.0 Hz, 1H), 6.81 (ddd, *J* = 8.7, 7.3, 1.9 Hz, 2H), 6.72 (t, *J* = 2.1 Hz, 1H), 6.68-6.51 (m, 2H), 6.18 (d, *J* = 15.9 Hz, 1H), 3.76 (s, 3H); ^{13}C{^{1}H} NMR (101 MHz, $CDCl_3$) δ 163.22, 159.77, 151.69, 150.00, 146.26, 139.98, 138.81, 137.08, 134.22, 129.77, 124.20, 124.14, 121.19, 119.81, 119.59, 114.26, 112.61, 104.07, 55.21; HRMS (ESI) calcd. for $C_{19}H_{16}N_2O_2$ $[M+H]^+$: 305.1285, found: 305.1294.

(*E*)-6-(2-chlorostyryl)-2H-[1,2'-bipyridin]-2-one (3ap)

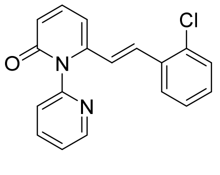

Yellow solid, 49.4 mg, 80%, mp: 162.3~164.1 °C; ^{1}H NMR (400 MHz, $CDCl_3$) δ 8.71 (ddd, J = 5.7, 1.9, 1.0 Hz, 1H), 7.93 (tt, J = 7.7, 1.5 Hz, 1H), 7.52-7.36 (m, 4H), 7.36-7.31 (m, 1H), 7.24-7.09 (m, 3H), 6.65 (dd, J = 8.1, 4.8 Hz, 2H), 6.20 (d, J = 16.0 Hz, 1H); ^{13}C{^{1}H} NMR (101 MHz, $CDCl_3$) δ 163.16, 151.60, 150.04, 145.87, 139.98, 138.90, 133.87, 133.77, 130.19, 129.97, 129.82, 127.01, 126.80, 124.23, 123.28, 120.26, 104.65; HRMS (ESI) calcd. for $C_{18}H_{13}ClN_2O$ [M+H]$^+$: 309.0789, found: 309.0796.

(*E*)-6-(4-hydroxy-3-methoxystyryl)-2H-[1,2'-bipyridin]-2-one (3aq)

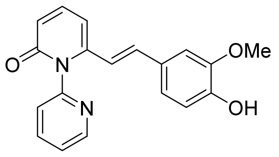

Brown oil, 46.8 mg, 73%; ^{1}H NMR (400 MHz, $CDCl_3$) δ 8.71 (ddd, J = 4.9, 1.9, 0.9 Hz, 1H), 7.93 (td, J = 7.7, 1.9 Hz, 1H), 7.50-7.37 (m, 3H), 6.96 (d, J = 15.9 Hz, 1H), 6.80 (d, J = 8.2 Hz, 1H), 6.74 (dd, J = 8.3, 1.9 Hz, 1H), 6.66 (d, J = 1.9 Hz, 1H), 6.64-6.55 (m, 2H), 6.28 (s, 1H), 6.00 (d, J = 15.9 Hz, 1H), 3.81 (s, 3H); ^{13}C{^{1}H} NMR (101 MHz, $CDCl_3$) δ 163.40, 151.83, 149.94, 146.99, 146.78, 146.73, 140.15, 138.85, 134.51, 128.16, 124.27, 124.06, 120.94, 119.12, 118.42, 114.84, 109.21, 103.65, 55.82; HRMS (ESI) calcd. for $C_{19}H_{16}N_2O_3$ [M+H]$^+$: 321.1234, found: 321.1239.

(*E*)-6-(2-(benzo[d][1,3]dioxol-5-yl)vinyl)-2H-[1,2'-bipyridin]-2-one (3ar)

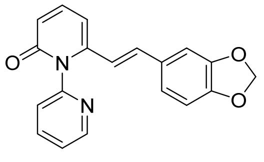

Brown oil, 57.3 mg, 90%; ^{1}H NMR (400 MHz, $CDCl_3$) δ 8.68 (ddd, J = 4.8, 2.0, 0.9 Hz, 1H), 7.90 (td, J = 7.7, 1.9 Hz, 1H), 7.47-7.31 (m, 3H), 6.92 (d, J = 15.9 Hz, 1H), 6.70 (d, J = 1.1 Hz, 2H), 6.65-6.51 (m, 3H), 5.97 (d, J = 15.8 Hz, 1H), 5.91 (s, 2H); ^{13}C{^{1}H} NMR (101 MHz, $CDCl_3$) δ 163.24, 151.75, 150.00, 148.45, 148.16, 146.47, 140.00, 138.81, 134.02, 130.11, 124.18, 124.15, 122.83, 119.29, 118.89, 108.45, 105.55, 103.60, 101.37; HRMS (ESI) calcd. for $C_{19}H_{14}N_2O_3$ [M+H]$^+$: 319.1077, found: 319.1085.

(*E*)-6-(3-bromo-4-fluorostyryl)-2H-[1,2'-bipyridin]-2-one (3as)

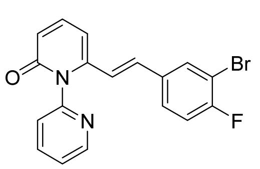

Yellow oil, 56.4 mg, 76%; ^{1}H NMR (400 MHz, $CDCl_3$) δ 8.71 (ddd, J = 4.9, 1.9, 0.8 Hz, 1H), 7.94 (td, J = 7.7, 1.9 Hz, 1H), 7.50-7.34 (m, 4H), 7.15-6.96 (m, 2H), 6.90 (d, J = 16.0 Hz, 1H), 6.63 (dd, J = 9.3, 1.1 Hz, 1H), 6.56 (dt, J = 7.1, 0.8 Hz, 1H), 6.11 (d, J = 16.0 Hz, 1H); ^{13}C{^{1}H} NMR (126 MHz, $CDCl_3$) δ 163.09, 160.13 (d, J^1_{C-F} = 252.00 Hz), 151.52, 150.03, 145.68, 139.88, 138.87, 133.47 (d, J^4_{C-F} = 3.78 Hz), 131.95, 131.60 (d, J^5_{C-F} = 1.26 Hz), 127.40 (d, J^3_{C-F} = 8.82 Hz), 124.27 (d, J^3_{C-F} = 7.56 Hz), 122.04 (d, J^2_{C-F} = 2.52 Hz), 120.31, 116.90, 116.72, 109.66 (d, J^2_{C-F} = 21.42 Hz), 104.30; HRMS (ESI) calcd. for $C_{18}H_{12}BrFN_2O$ [M+H]$^+$: 371.0190, found: 371.0197.

(*E*)-6-(3,4,5-trimethoxystyryl)-2H-[1,2'-bipyridin]-2-one (3at)

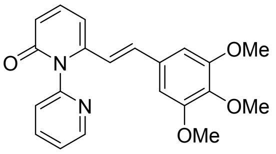

Yellow oil, 61.2 mg, 84%; ^{1}H NMR (400 MHz, $CDCl_3$) δ 8.74-8.67 (m, 1H), 7.93 (td, J = 7.7, 1.9 Hz, 1H), 7.48-7.35 (m, 3H), 6.95 (d, J = 15.9 Hz, 1H), 6.64-6.53 (m, 2H), 6.41 (s, 2H), 6.06 (d, J = 15.8 Hz, 1H), 3.82 (s, 3H), 3.78 (s, 6H); ^{13}C{^{1}H} NMR (101 MHz, $CDCl_3$) δ 163.18, 153.32, 151.83, 149.90, 146.25, 139.98, 139.02, 138.83, 134.22, 131.38, 124.31, 124.01, 120.44, 119.70, 104.19, 103.87, 60.94, 56.06; HRMS (ESI) calcd. for $C_{21}H_{20}N_2O_4$ $[M+H]^+$: 365.1496, found: 365.1489.

(*E*)-6-(2-(perfluorophenyl)vinyl)-2H-[1,2'-bipyridin]-2-one (3au)

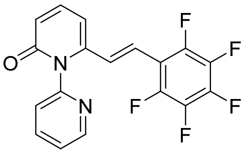

Yellow solid, 51.0 mg, 70%, mp: 208.8~210.5 °C; ^{1}H NMR (400 MHz, $CDCl_3$) δ 8.70 (ddd, J = 4.9, 1.9, 0.9 Hz, 1H), 7.95 (td, J = 7.7, 1.9 Hz, 1H), 7.52-7.41 (m, 3H), 6.93 (d, J = 16.5 Hz, 1H), 6.73-6.60 (m, 2H), 6.56 (d, J = 16.5 Hz, 1H); ^{13}C{^{1}H} NMR (101 MHz, $CDCl_3$) δ 162.82, 151.21, 150.02, 146.18, 146.12, 145.33, 143.71, 143.64, 139.65, 138.78, 136.30, 129.34, 129.25, 129.14, 124.29, 124.10, 121.39, 117.65, 110.95, 110.91, 104.72; ^{19}F NMR (376 MHz, $CDCl_3$) δ -141.92 (d, J = 7.5 Hz), -141.95 - -142.02 (m), -153.53 (t, J = 20.8 Hz), -161.88 - -162.11 (m); HRMS (ESI) calcd. for $C_{18}H_9F_5N_2O$ $[M+H]^+$: 365.0708, found: 365.0716.

(*E*)-6-(2-(naphthalen-2-yl)vinyl)-2H-[1,2'-bipyridin]-2-one (3av)

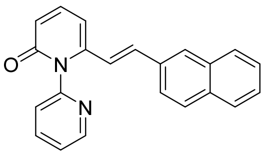

Yellow solid, 57.7 mg, 89%, mp: 133.8~134.2 °C;; ^{1}H NMR (400 MHz, $CDCl_3$) δ 8.76-8.69 (m, 1H), 7.92 (tt, J = 7.7, 1.7 Hz, 1H), 7.83-7.73 (m, 2H), 7.74-7.63 (m, 2H), 7.53-7.39 (m, 5H), 7.30-7.14 (m, 2H), 6.68-6.60 (m, 2H), 6.29 (d, J = 15.9 Hz, 1H); ^{13}C{^{1}H} NMR (101 MHz, $CDCl_3$) δ 163.28, 151.74, 150.07, 146.40, 140.07, 138.90, 134.45, 133.49, 133.32, 133.13, 128.56, 128.22, 127.73, 126.76, 126.68, 124.24, 122.96, 120.99, 119.71, 104.03; HRMS (ESI) calcd. for $C_{22}H_{16}N_2O$ $[M+H]^+$: 325.1335, found: 325.1342.

(*E*)-6-(2-(pyridin-3-yl)vinyl)-2H-[1,2'-bipyridin]-2-one (3aw)

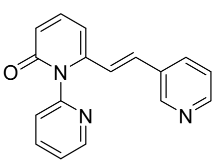

Yellow oil, 45.1 mg, 82%; ^{1}H NMR (400 MHz, $CDCl_3$) δ 8.70 (dd, J = 5.0, 1.8 Hz, 1H), 8.54-8.42 (m, 2H), 7.94 (td, J = 7.7, 1.9 Hz, 1H), 7.54-7.38 (m, 4H), 7.21 (dd, J = 8.0, 4.8 Hz, 1H), 7.00 (d, J = 16.0 Hz, 1H), 6.63 (dd, J = 10.4, 8.1 Hz, 2H), 6.26 (d, J = 16.1 Hz, 1H); ^{13}C{^{1}H} NMR (101 MHz, $CDCl_3$) δ 163.04, 151.47, 150.03, 149.70, 148.65, 145.54, 139.85, 138.86, 133.34, 131.42, 130.44, 124.28, 124.22, 123.59, 122.95, 120.51, 104.38; HRMS (ESI) calcd. for $C_{17}H_{13}N_3O$ $[M+H]^+$: 276.1131, found: 276.1139.

(*E*)-6-(2-(furan-2-yl)vinyl)-2H-[1,2'-bipyridin]-2-one (3ax)

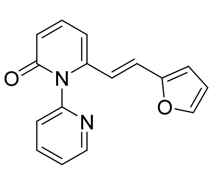

Brown oil, 47.0 mg, 89%; ^{1}H NMR (400 MHz, $CDCl_3$) δ 8.70 (ddd, J = 4.9, 2.0, 0.8 Hz, 1H), 7.93 (td, J = 7.7, 1.9 Hz, 1H), 7.48-7.36 (m, 3H), 7.29 (d, J = 1.6 Hz, 1H), 6.80 (d, J = 15.8 Hz, 1H), 6.63-6.51 (m, 2H), 6.42-6.30 (m, 2H), 6.06 (d, J = 15.8 Hz, 1H); ^{13}C{^{1}H} NMR (101 MHz, $CDCl_3$) δ 163.26, 151.63, 150.07, 146.10, 143.49, 139.87, 138.80, 124.14, 121.35, 119.52, 118.60, 111.97, 111.81, 103.48; HRMS (ESI) calcd. for $C_{16}H_{12}N_2O_2$ $[M+H]^+$: 265.0972, found: 265.0979.

(*E*)-6-(2-(thiophen-2-yl)vinyl)-2H-[1,2'-bipyridin]-2-one (3ay)

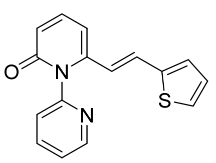

Brown oil, 50.5 mg, 90%; ^{1}H NMR (400 MHz, $CDCl_3$) δ 8.79-8.67 (m, 1H), 7.93 (td, J = 7.7, 1.9 Hz, 1H), 7.43 (ddd, J = 12.7, 8.2, 5.6 Hz, 3H), 7.22-7.08 (m, 2H), 7.05-6.86 (m, 2H), 6.66-6.52 (m, 2H), 5.97 (d, J = 15.8 Hz, 1H); ^{13}C{^{1}H} NMR (101 MHz, $CDCl_3$) δ 163.16, 151.64, 150.02, 145.92, 141.02, 139.89, 138.80, 128.26, 127.89, 127.03, 126.46, 124.19, 124.13, 119.95, 119.68, 103.68; HRMS (ESI) calcd. for $C_{16}H_{12}N_2OS$ $[M+H]^+$: 281.0743, found: 281.0751.

6-((*E*)-2-((8R,9S,13S,14S)-13-methyl-17-oxo-7,8,9,11,12,13,14,15,16,17-decahydro-6H-cyclopenta[a]phenanthren-3-yl)vinyl)-2H-[1,2'-bipyridin]-2-one (3az)

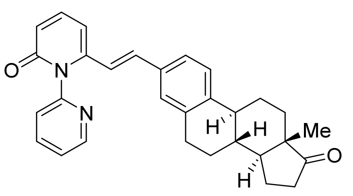

Yellow oil, 80.2 mg, 89%; ^{1}H NMR (400 MHz, $CDCl_3$) δ 8.70 (dd, J = 5.0, 1.8 Hz, 1H), 7.92 (td, J = 7.7, 1.9 Hz, 1H), 7.48-7.35 (m, 3H), 7.20 (d, J = 8.1 Hz, 1H), 7.03-6.91 (m, 3H), 6.63-6.54 (m, 2H), 6.14 (d, J = 15.9 Hz, 1H), 2.85 (dd, J = 9.1, 4.2 Hz, 2H), 2.50 (dd, J = 18.8, 8.7 Hz, 1H), 2.43-2.22 (m, 2H), 2.21-1.89 (m, 4H), 1.70-1.33 (m, 6H), 0.89 (s, 3H); ^{13}C{^{1}H} NMR (101 MHz, $CDCl_3$) δ 220.66, 171.15, 163.26, 151.76, 150.01, 146.57, 141.07, 140.03, 138.83, 136.97, 134.26, 133.26, 127.96, 125.82, 124.18, 124.11, 120.17, 119.50, 103.88, 50.41, 47.90, 44.46, 37.99, 35.82, 31.52, 29.26, 26.31, 25.62, 21.56, 13.81; HRMS (ESI) calcd. for $C_{30}H_{30}N_2O_2$ $[M+H]^+$: 451.2380, found: 451.2388.

(*E*)-3-methyl-6-styryl-2H-[1,2'-bipyridin]-2-one (3ba)

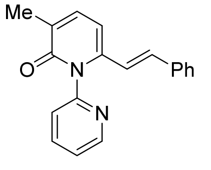

Yellow solid, 51.9 mg, 90%, mp: 109.3~110.7 °C; ^{1}H NMR (400 MHz, $CDCl_3$) δ 8.70 (dd, J = 5.1, 1.8 Hz, 1H), 7.91 (td, J = 7.7, 1.9 Hz, 1H), 7.46-7.36 (m, 2H), 7.35-7.21 (m, 4H), 7.19 (dd, J = 7.6, 2.0 Hz, 2H), 6.98 (d, J = 16.0 Hz, 1H), 6.55 (d, J = 7.1 Hz, 1H), 6.19 (d, J = 16.0 Hz, 1H), 2.20 (s, 3H); ^{13}C{^{1}H} NMR (101 MHz, $CDCl_3$) δ 163.48, 152.13, 149.96, 143.58, 138.68, 137.23, 135.90, 133.10, 129.01, 128.73, 128.70, 126.89, 124.21, 123.99, 121.03, 103.77, 17.14; HRMS (ESI) calcd. for $C_{19}H_{16}N_2O$ $[M+H]^+$: 289.1335, found: 289.1342.

(*E*)-3-(benzyloxy)-6-styryl-2H-[1,2'-bipyridin]-2-one (3ca)

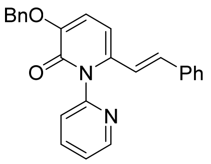

Yellow solid, 69.2 mg, 91%; mp:112.2~114.5 °C; ^{1}H NMR (400 MHz, $CDCl_3$) δ 8.72 (dd, *J* = 4.9, 1.8 Hz, 1H), 7.96 (td, *J* = 7.8, 1.9 Hz, 1H), 7.52-7.40 (m, 4H), 7.41-7.35 (m, 2H), 7.35-7.30 (m, 1H), 7.32-7.21 (m, 3H), 7.18 (dd, *J* = 7.8, 1.8 Hz, 2H), 6.98-6.79 (m, 2H), 6.50 (d, *J* = 7.8 Hz, 1H), 6.15 (d, *J* = 15.9 Hz, 1H), 5.21 (s, 2H); ^{13}C{^{1}H} NMR (101 MHz, $CDCl_3$) δ 158.66, 151.13, 149.52, 147.93, 139.35, 138.20, 136.37, 136.03, 132.29, 128.71, 128.57, 128.00, 127.42, 126.80, 124.49, 124.36, 120.69, 117.40, 103.47, 71.05; HRMS (ESI) calcd. for $C_{25}H_{20}N_2O_2$ $[M+H]^+$: 381.1598, found: 381.1608.

(*E*)-3-fluoro-6-styryl-2H-[1,2'-bipyridin]-2-one (3da)

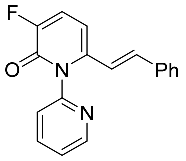

Yellow solid, 50.9 mg, 87%, mp: 133.4~135.3 °C; ^{1}H NMR (400 MHz, $CDCl_3$) δ 8.73 (ddd, *J* = 4.9, 1.9, 0.8 Hz, 1H), 7.96 (td, *J* = 7.7, 1.9 Hz, 1H), 7.48 (ddd, *J* = 7.6, 4.9, 1.1 Hz, 1H), 7.43 (dt, *J* = 8.0, 1.0 Hz, 1H), 7.32-7.23 (m, 3H), 7.26-7.17 (m, 3H), 7.00 (d, *J* = 16.0 Hz, 1H), 6.53 (dd, *J* = 7.9, 4.3 Hz, 1H), 6.17 (d, *J* = 15.9 Hz, 1H); ^{13}C{^{1}H} NMR (101 MHz, $CDCl_3$) δ 156.74 (d, $J^2_{C\text{-}F}$ = 26.26 Hz), 152.82 (d, $J^1_{C\text{-}F}$ = 252.50 Hz), 150.83, 150.10, 142.07 (d, $J^3_{C\text{-}F}$ = 6.06 Hz), 138.94, 135.62, 134.05 (d, $J^4_{C\text{-}F}$ = 3.03 Hz), 128.96, 128.79, 126.95, 124.50, 124.08, 120.79 (d, $J^2_{C\text{-}F}$ = 17.17 Hz), 120.29 (d, $J^5_{C\text{-}F}$ = 2.02 Hz), 101.87 (d, $J^4_{C\text{-}F}$ = 5.05 Hz); HRMS (ESI) calcd. for $C_{18}H_{13}FN_2O$ $[M+H]^+$: 293.1085, found: 293.1093.

(*E*)-3-chloro-6-styryl-2H-[1,2'-bipyridin]-2-one (3ea)

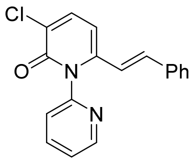

Yellow solid, 52.5 mg, 85%, mp: 111.7~112.3 °C; ^{1}H NMR (400 MHz, $CDCl_3$) δ 8.70 (ddd, *J* = 4.9, 1.9, 0.9 Hz, 1H), 7.94 (td, *J* = 7.7, 1.9 Hz, 1H), 7.63 (d, *J* = 7.7 Hz, 1H), 7.50-7.37 (m, 2H), 7.34-7.24 (m, 3H), 7.20 (dt, *J* = 6.7, 2.3 Hz, 2H), 7.05 (d, *J* = 16.0 Hz, 1H), 6.59 (d, *J* = 7.7 Hz, 1H), 6.17 (d, *J* = 16.0 Hz, 1H); ^{13}C{^{1}H} NMR (101 MHz, $CDCl_3$) δ 159.30, 151.32, 150.02, 145.19, 138.96, 137.99, 135.46, 134.81, 129.17, 128.82, 127.07, 124.88, 124.47, 124.02, 120.16, 103.29; HRMS (ESI) calcd. for $C_{18}H_{13}ClN_2O$ $[M+H]^+$: 309.0789, found: 309.0796.

(*E*)-3-bromo-6-styryl-2H-[1,2'-bipyridin]-2-one (3fa)

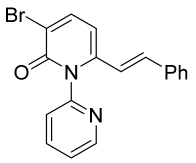

Yellow solid, 61.5 mg, 87%, mp: 121.9~123.6 °C; ^{1}H NMR (400 MHz, $CDCl_3$) δ 8.71 (ddd, *J* = 4.9, 1.9, 0.9 Hz, 1H), 7.95 (td, *J* = 7.7, 2.0 Hz, 1H), 7.85 (d, *J* = 7.7 Hz, 1H), 7.47 (ddd, *J* = 7.5, 4.9, 1.1 Hz, 1H), 7.46-7.38 (m, 1H), 7.34-7.24 (m, 3H), 7.21 (dd, *J* = 6.8, 3.0 Hz, 2H), 7.07 (d, *J* = 15.9 Hz, 1H), 6.54 (d, *J* = 7.7 Hz, 1H), 6.17 (d, *J* = 16.0 Hz, 1H); ^{13}C{^{1}H} NMR (101 MHz, $CDCl_3$) δ 159.33, 151.44, 149.99, 146.03, 141.83, 138.91, 135.47, 134.90, 129.19, 128.83, 127.08, 124.43, 124.02, 120.23, 114.98, 103.88; HRMS (ESI) calcd. for $C_{18}H_{13}BrN_2O$ $[M+H]^+$: 353.0284, found: 353.0292.

(*E*)-6-styryl-3-(trifluoromethyl)-2H-[1,2'-bipyridin]-2-one (3ga)

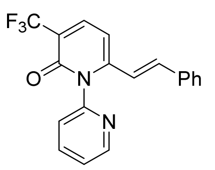

Yellow solid, 52.7 mg, 77%, mp: 114.9~116.5 °C; ^{1}H NMR (400 MHz, $CDCl_3$) δ 8.75-8.69 (m, 1H), 7.97 (qd, J = 7.5, 1.5 Hz, 1H), 7.84 (d, J = 7.5 Hz, 1H), 7.53-7.41 (m, 2H), 7.31 (dp, J = 4.0, 1.8 Hz, 3H), 7.28-7.13 (m, 3H), 6.68 (d, J = 7.6 Hz, 1H), 6.22 (d, J = 16.0 Hz, 1H); ^{13}C{^{1}H} NMR ^{13}C NMR (126 MHz, $CDCl_3$) δ 158.07, 149.57, 149.44, 149.11, 138.18 (q, $J^{3}_{C\text{-}F}$ = 5.04 Hz), 137.92, 135.95, 133.98, 128.66, 127.86, 126.30, 125.03 (q, $J^{1}_{C\text{-}F}$ = 272.16 Hz), 123.55, 123.14, 118.72, 117.94 (q, $J^{2}_{C\text{-}F}$ = 31.5 Hz), 100.77; HRMS (ESI) calcd. for $C_{19}H_{13}F_3N_2O$ $[M+H]^+$: 343.1053, found: 343.1060.

(*E*)-4-methyl-6-styryl-2H-[1,2'-bipyridin]-2-one (3ha)

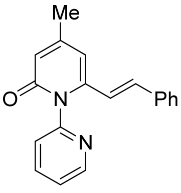

Yellow solid, 51.3 mg, 89%, mp: 104.2~105.8 °C; ^{1}H NMR (400 MHz, $CDCl_3$) δ 8.69 (ddd, J = 4.9, 1.9, 0.9 Hz, 1H), 7.90 (td, J = 7.7, 1.9 Hz, 1H), 7.46-7.34 (m, 2H), 7.33-7.21 (m, 3H), 7.23-7.17 (m, 2H), 7.03 (d, J = 16.0 Hz, 1H), 6.47 (dd, J = 6.9, 1.4 Hz, 2H), 6.20 (d, J = 16.0 Hz, 1H), 2.28 (d, J = 1.1 Hz, 3H); ^{13}C{^{1}H} NMR (101 MHz, $CDCl_3$) δ 163.29, 151.69, 151.56, 149.92, 145.02, 138.71, 135.72, 134.08, 128.89, 128.76, 126.98, 124.35, 124.04, 120.89, 118.20, 106.72, 21.63; HRMS (ESI) calcd. for $C_{19}H_{16}N_2O$ $[M+H]^+$: 289.1335, found: 289.1343.

(*E*)-4-(benzyloxy)-6-styryl-2H-[1,2'-bipyridin]-2-one (3ia)

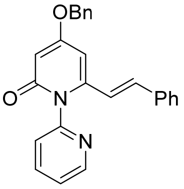

Yellow solid, 69.2 mg, 91%, mp:110.7~112.2 °C;^{1}H NMR (400 MHz, $CDCl_3$) δ 8.71 (dd, J = 5.0, 1.8 Hz, 1H), 7.92 (td, J = 7.7, 1.9 Hz, 1H), 7.54-7.37 (m, 7H), 7.34-7.24 (m, 3H), 7.24-7.16 (m, 2H), 7.04 (d, J = 15.9 Hz, 1H), 6.43 (d, J = 2.5 Hz, 1H), 6.20 (d, J = 15.9 Hz, 1H), 6.09 (d, J = 2.5 Hz, 1H), 5.10 (s, 2H); ^{13}C{^{1}H} NMR (101 MHz, $CDCl_3$) δ 167.28, 164.88, 151.54, 149.90, 146.11, 138.68, 135.57, 135.33, 134.57, 129.01, 128.77, 128.50, 127.77, 127.06, 124.57, 124.02, 120.66, 99.09, 97.26, 70.33; HRMS (ESI) calcd. for $C_{25}H_{20}N_2O_2$ $[M+H]^+$: 381.1598, found: 381.1607.

(*E*)-4-phenyl-6-styryl-2H-[1,2'-bipyridin]-2-one (3ja)

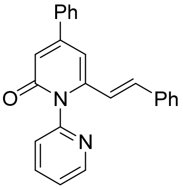

Yellow oil, 61.0 mg, 87%; ^{1}H NMR (400 MHz, $CDCl_3$) δ 8.74 (ddd, J = 4.9, 1.9, 0.9 Hz, 1H), 7.96 (td, J = 7.7, 1.9 Hz, 1H), 7.76-7.66 (m, 2H), 7.59-7.41 (m, 6H), 7.34-7.21 (m, 4H), 7.14 (d, J = 15.9 Hz, 1H), 6.88 (dd, J = 13.3, 1.8 Hz, 2H), 6.30 (d, J = 15.9 Hz, 1H); ^{13}C{^{1}H} NMR (101 MHz, $CDCl_3$) δ 163.46, 152.18, 151.65, 150.05, 146.03, 138.79, 137.84, 135.66, 134.51, 129.59, 129.05, 128.81, 127.06, 126.88, 124.32, 124.16, 121.12, 116.17, 103.97; HRMS (ESI) calcd. for $C_{24}H_{18}N_2O$ $[M+H]^+$: 351.1492, found: 351.1501.

(*E*)-4-chloro-6-styryl-2H-[1,2'-bipyridin]-2-one (3ka)

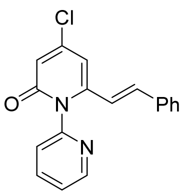

Yellow solid, 47.6 mg, 77%, mp: 115.7~116.4 °C; ^{1}H NMR (400 MHz, $CDCl_3$) δ 8.72 (ddd, *J* = 4.9, 1.9, 0.9 Hz, 1H), 7.95 (td, *J* = 7.7, 1.9 Hz, 1H), 7.51-7.37 (m, 2H), 7.37-7.26 (m, 3H), 7.27-7.17 (m, 2H), 7.09 (d, *J* = 15.9 Hz, 1H), 6.66 (dd, *J* = 17.9, 2.1 Hz, 2H), 6.18 (d, *J* = 16.0 Hz, 1H); $^{13}C\{^{1}H\}$ NMR (101 MHz, $CDCl_3$) δ 162.32, 150.96, 150.11, 147.47, 146.62, 138.92, 135.81, 135.20, 129.41, 128.85, 127.20, 124.40, 124.19, 119.74, 117.87, 105.61; HRMS (ESI) calcd. for $C_{18}H_{13}ClN_2O$ $[M+H]^+$: 309.0789, found: 309.0796.

(*E*)-4-bromo-6-styryl-2H-[1,2'-bipyridin]-2-one (3la)

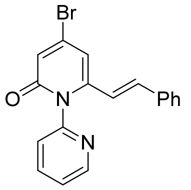

Yellow solid, 55.8 mg, 79%, mp: 125.6~126.5 °C; ^{1}H NMR (400 MHz, $CDCl_3$) δ 8.72 (ddd, *J* = 4.9, 1.9, 0.8 Hz, 1H), 7.94 (td, *J* = 7.7, 1.9 Hz, 1H), 7.51-7.36 (m, 2H), 7.35-7.27 (m, 3H), 7.21 (dd, *J* = 6.7, 3.0 Hz, 2H), 7.09 (d, *J* = 15.9 Hz, 1H), 6.89 (d, *J* = 1.9 Hz, 1H), 6.77 (d, *J* = 1.9 Hz, 1H), 6.17 (d, *J* = 15.9 Hz, 1H); $^{13}C\{^{1}H\}$ NMR (101 MHz, $CDCl_3$) δ 161.97, 150.99, 150.12, 146.33, 138.91, 136.47, 135.83, 135.22, 129.41, 128.86, 127.20, 124.40, 124.13, 121.44, 119.60, 108.07; HRMS (ESI) calcd. for $C_{18}H_{13}BrN_2O$ $[M+H]^+$: 353.0284, found: 353.0292.

(*E*)-5-methyl-6-styryl-2H-[1,2'-bipyridin]-2-one (3ma)

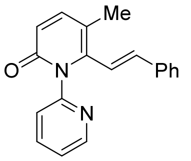

Yellow solid, 47.9 mg, 83%, mp: 108.4~109.7 °C; ^{1}H NMR (400 MHz, $CDCl_3$) δ 8.70-8.61 (m, 1H), 7.86 (td, *J* = 7.7, 1.9 Hz, 1H), 7.43-7.31 (m, 3H), 7.35-7.21 (m, 3H), 7.21-7.10 (m, 2H), 6.66-6.51 (m, 2H), 6.27 (d, *J* = 16.5 Hz, 1H), 2.26 (s, 3H); $^{13}C\{^{1}H\}$ NMR (101 MHz, $CDCl_3$) δ 162.68, 152.61, 149.82, 144.27, 142.13, 138.62, 137.46, 135.74, 128.76, 128.73, 126.52, 124.31, 123.82, 120.42, 119.82, 113.86, 18.50; HRMS (ESI) calcd. for $C_{19}H_{16}N_2O$ $[M+H]^+$: 289.1335, found: 289.1343.

(*E*)-5-chloro-6-styryl-2H-[1,2'-bipyridin]-2-one (3na)

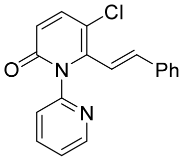

Yellow solid, 43.8 mg, 71%, mp: 122.4~125.8 °C; ^{1}H NMR (400 MHz, $CDCl_3$) δ 8.68 (dd, *J* = 5.0, 1.7 Hz, 1H), 7.90 (td, *J* = 7.7, 1.9 Hz, 1H), 7.49 (d, *J* = 9.8 Hz, 1H), 7.44-7.34 (m, 2H), 7.28 (s, 3H), 7.17 (dd, *J* = 6.8, 2.9 Hz, 2H), 6.79 (d, *J* = 16.7 Hz, 1H), 6.61 (d, *J* = 9.8 Hz, 1H), 6.36 (d, *J* = 16.6 Hz, 1H); $^{13}C\{^{1}H\}$ NMR (101 MHz, $CDCl_3$) δ 162.02, 151.99, 149.95, 142.25, 141.83, 139.39, 138.78, 135.43, 129.19, 128.76, 126.80, 124.22, 120.71, 118.44, 112.41; HRMS (ESI) calcd. for $C_{18}H_{13}ClN_2O$ $[M+H]^+$: 309.0789, found: 309.0797.

(*E*)-5-bromo-6-styryl-2H-[1,2'-bipyridin]-2-one (3oa)

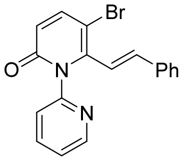

Yellow solid, 48.0 mg, 68%, mp: 131.9~133.5 °C; ^{1}H NMR (400 MHz, $CDCl_3$) δ 8.69-8.62 (m, 1H), 7.88 (td, *J* = 7.7, 1.9 Hz, 1H), 7.61 (d, *J* = 9.8 Hz, 1H), 7.45-7.23 (m, 5H), 7.22-7.13 (m, 2H), 6.67 (d, *J* = 16.6 Hz, 1H), 6.55 (d, *J* = 9.8 Hz, 1H), 6.40 (d, *J* = 16.6 Hz, 1H); $^{13}C\{^{1}H\}$ NMR (101 MHz, $CDCl_3$) δ 162.23, 152.18, 149.86,

144.51, 143.22, 139.44, 138.67, 135.30, 129.16, 128.75, 126.76, 124.19, 124.15, 120.96, 120.44, 100.10; HRMS (ESI) calcd. for $C_{18}H_{13}BrN_2O$ $[M+H]^+$: 353.0284, found: 353.0293.

(*E*)-6-styryl-5-(trifluoromethyl)-2H-[1,2'-bipyridin]-2-one (3pa)

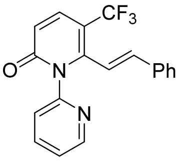

Yellow solid, 48.6 mg, 71%, mp: 133.1~134.8 °C; ^{1}H NMR (400 MHz, $CDCl_3$) δ 8.67 (dd, *J* = 5.2, 1.9 Hz, 1H), 7.87 (td, *J* = 7.7, 1.9 Hz, 1H), 7.69 (d, *J* = 9.8 Hz, 1H), 7.41-7.23 (m, 5H), 7.19-7.10 (m, 2H), 6.73-6.59 (m, 2H), 6.49 (dq, *J* = 16.7, 1.9 Hz, 1H); ^{13}C{^{1}H} NMR (126 MHz, $CDCl_3$) δ 162.41, 151.38, 149.95, 148.09 (q, J^3_{C-F} = 2.52 Hz), 139.59 (q, J^4_{C-F} = 2.52 Hz), 138.76, 136.95 (q, J^3_{C-F} = 5.04 Hz), 135.09, 129.31, 128.76, 126.98 (q, J^1_{C-F} = 272.16 Hz), 126.87, 124.32, 124.19, 119.77, 117.94, 109.39 (q, J^2_{C-F} = 32.76 Hz); HRMS (ESI) calcd. for $C_{19}H_{13}F_3N_2O$ $[M+H]^+$: 343.1053, found: 343.1061.

methyl (*E*)-2-oxo-6-styryl-2H-[1,2'-bipyridine]-5-carboxylate (3qa)

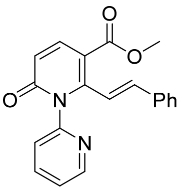

Yellow oil, 44.5 mg, 67%; ^{1}H NMR (400 MHz, $CDCl_3$) δ 8.67-8.60 (m, 1H), 8.00 (d, *J* = 9.8 Hz, 1H), 7.84 (td, *J* = 7.7, 1.9 Hz, 1H), 7.38-7.20 (m, 5H), 7.20-7.13 (m, 2H), 6.97 (d, *J* = 16.7 Hz, 1H), 6.63 (d, *J* = 9.7 Hz, 1H), 6.35 (d, *J* = 16.7 Hz, 1H), 3.82 (s, 3H); ^{13}C{^{1}H} NMR (101 MHz, $CDCl_3$) δ 165.71, 162.91, 152.06, 151.16, 149.71, 140.69, 138.50, 138.18, 135.62, 128.90, 128.68, 126.75, 124.27, 124.08, 121.08, 118.99, 109.68, 52.17; HRMS (ESI) calcd. for $C_{20}H_{16}N_2O_3$ $[M+H]^+$: 333.1234, found: 333.1242.

(*E*)-2-oxo-4-phenyl-6-styryl-2H-[1,2'-bipyridine]-3-carbonitrile (3ra)

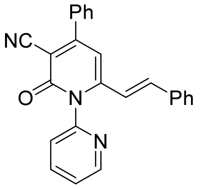

Brown oil, 56.3 mg, 75%; ^{1}H NMR (400 MHz, $CDCl_3$) δ 8.74 (ddd, *J* = 4.9, 1.9, 0.9 Hz, 1H), 8.00 (td, *J* = 7.7, 1.9 Hz, 1H), 7.80-7.67 (m, 2H), 7.63-7.45 (m, 5H), 7.45-7.19 (m, 6H), 6.80 (s, 1H), 6.28 (d, *J* = 15.9 Hz, 1H); ^{13}C{^{1}H} NMR (101 MHz, $CDCl_3$) δ 161.20, 159.65, 150.24, 149.85, 139.15, 138.17, 136.02, 134.83, 130.72, 130.05, 129.05, 128.98, 128.05, 127.51, 124.85, 124.10, 119.62, 115.70, 105.27, 100.56; HRMS (ESI) calcd. for $C_{25}H_{17}N_3O$ $[M+H]^+$: 376.1444, found: 376.1452.

(*E*)-2-(pyridin-2-yl)-3-styrylisoquinolin-1(2H)-one (3sa)

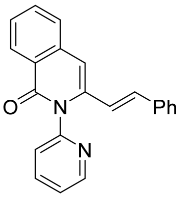

Yellow oil, 40.9 mg, 63%; ^{1}H NMR (400 MHz, $CDCl_3$) δ 8.74 (ddd, *J* = 4.9, 2.0, 0.9 Hz, 1H), 8.44 (dd, *J* = 8.1, 1.3 Hz, 1H), 7.93 (td, *J* = 7.7, 2.0 Hz, 1H), 7.69 (ddd, *J* = 8.2, 7.1, 1.4 Hz, 1H), 7.61 (dd, *J* = 8.0, 1.2 Hz, 1H), 7.53-7.41 (m, 3H), 7.35-7.20 (m, 5H), 7.09 (d, *J* = 16.0 Hz, 1H), 6.95 (s, 1H), 6.37-6.26 (m, 1H); ^{13}C{^{1}H} NMR (101 MHz, $CDCl_3$) δ 163.04, 152.17, 149.92, 140.29, 138.66, 137.07, 136.12, 132.93, 132.83, 128.74, 128.58, 128.18, 126.89, 126.25, 125.37, 124.64, 123.93, 121.80, 104.35; HRMS (ESI) calcd. for $C_{22}H_{16}N_2O$ $[M+H]^+$: 325.1335, found: 325.1342.

(*E*)-5'-methyl-6-styryl-2H-[1,2'-bipyridin]-2-one (3ta)

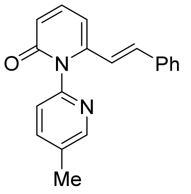

Yellow oil, 51.3 mg, 89%; ^{1}H NMR (400 MHz, $CDCl_3$) δ 8.52 (d, *J* = 2.3 Hz, 1H), 7.72 (dd, *J* = 8.1, 2.3 Hz, 1H), 7.43 (dd, *J* = 9.2, 7.1 Hz, 1H), 7.37-7.18 (m, 6H), 7.04 (d, *J* = 15.9 Hz, 1H), 6.65-6.56 (m, 2H), 6.24 (d, *J* = 16.0 Hz, 1H), 2.44 (s, 3H); ^{13}C{^{1}H} NMR (101 MHz, $CDCl_3$) δ 163.40, 150.29, 149.21, 146.52, 139.91, 139.40, 135.72, 134.26, 134.05, 128.94, 128.77, 127.08, 123.36, 120.97, 119.72, 103.94, 18.26; HRMS (ESI) calcd. for $C_{19}H_{16}N_2O$ $[M+H]^+$: 289.1335, found: 289.1343.

(*E*)-4'-methyl-6-styryl-2H-[1,2'-bipyridin]-2-one (3ua)

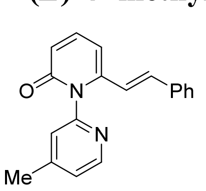

Yellow oil, 50.8 mg, 88%;^{1}H NMR (400 MHz, $CDCl_3$) δ 8.54 (d, *J* = 5.1 Hz, 1H), 7.42 (dd, *J* = 9.2, 7.1 Hz, 1H), 7.32-7.14 (m, 7H), 6.64-6.54 (m, 2H), 6.21 (d, *J* = 16.0 Hz, 1H), 2.43 (s, 3H); ^{13}C{^{1}H} NMR (101 MHz, $CDCl_3$) δ 163.27, 151.68, 150.53, 149.53, 146.40, 139.92, 135.72, 134.25, 128.93, 128.76, 127.04, 125.29, 124.75, 120.88, 119.66, 103.88, 21.11; HRMS (ESI) calcd. for $C_{19}H_{16}N_2O$ $[M+H]^+$: 289.1335, found: 289.1343.

(*E*)-6-styryl-5'-(trifluoromethyl)-2H-[1,2'-bipyridin]-2-one (3va)

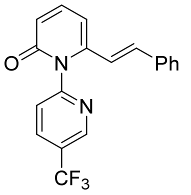

Yellow solid, 56.8 mg, 83%, mp: 143.8~145.2 °C; ^{1}H NMR (400 MHz, $CDCl_3$) δ 8.98 (d, *J* = 2.4 Hz, 1H), 8.18 (dd, *J* = 8.3, 2.4 Hz, 1H), 7.60 (d, *J* = 8.3 Hz, 1H), 7.49 (dd, *J* = 9.1, 7.3 Hz, 1H), 7.42-7.27 (m, 3H), 7.24 (dd, *J* = 7.4, 2.3 Hz, 2H), 7.08 (d, *J* = 15.9 Hz, 1H), 6.68-6.60 (m, 2H), 6.19 (d, *J* = 15.9 Hz, 1H); ^{13}C{^{1}H} NMR (101 MHz, $CDCl_3$) δ 162.97, 154.67, 147.17 (q, $J^3_{C\text{-}F}$ = 4.04 Hz), 146.00, 140.32, 136.17 (q, $J^3_{C\text{-}F}$ = 4.04 Hz), 135.40, 135.04, 129.25, 128.88, 127.52 (q, $J^2_{C\text{-}F}$ = 33.33 Hz), 127.10, 124.54, 124.41 (q, $J^1_{C\text{-}F}$ = 272.70 Hz), 120.31, 119.84, 104.43; HRMS (ESI) calcd. for $C_{19}H_{13}F_3N_2O$ $[M+H]^+$: 343.1053, found: 343.1060.

4-(pyridin-2-yl)-3,5-di((*E*)-styryl)cyclohexa-2,5-dien-1-one (3wa)

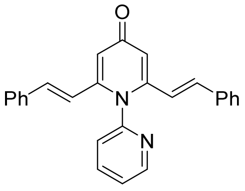

Yellow oil, 66.3 mg, 88%; ^{1}H NMR (400 MHz, Chloroform-*d*) δ 8.77 (dd, *J* = 4.9, 1.8 Hz, 1H), 7.93 (td, *J* = 7.7, 2.0 Hz, 1H), 7.57-7.49 (m, 1H), 7.34-7.24 (m, 7H), 7.24-7.17 (m, 4H), 7.09 (d, *J* = 15.8 Hz, 2H), 6.76 (s, 2H), 6.20 (d, *J* = 15.8 Hz, 2H); ^{13}C{^{1}H} NMR (101 MHz, $CDCl_3$) δ 179.62, 152.06, 150.24, 148.70, 139.37, 136.23, 135.41, 129.20, 128.80, 127.17, 124.97, 124.50, 120.43, 114.97; HRMS (ESI) calcd. for $C_{26}H_{20}N_2O$ $[M+H]^+$: 377.1648, found: 377.1657.

(*E*)-1-(pyridin-2-yl)-2-styrylquinolin-4(1H)-one (3xa)

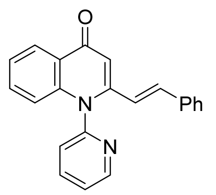

Brown oil, 59.0 mg, 91%; ^{1}H NMR (400 MHz, $CDCl_3$) δ 8.88-8.81 (m, 1H), 8.47 (dd, *J* = 8.0, 1.6 Hz, 1H), 8.04 (td, *J* = 7.7, 2.0 Hz, 1H), 7.61 (ddd, *J* = 7.6, 4.9, 1.0 Hz, 1H), 7.51-7.38 (m, 2H), 7.35 (ddd, *J* = 8.1, 7.0, 1.0 Hz, 1H), 7.31 (dd, *J* = 5.0, 1.9 Hz, 3H), 7.28-7.17 (m, 3H), 6.76 (s, 1H), 6.66 (d, *J* = 8.5 Hz, 1H), 6.30 (d, *J* = 15.9 Hz, 1H); $^{13}C\{^1H\}$ NMR (101 MHz, $CDCl_3$) δ 178.32, 152.06, 150.88, 149.82, 141.76, 139.79, 137.05, 135.37, 131.96, 129.36, 128.84, 127.28, 126.23, 126.02, 125.01, 124.89, 123.84, 120.95, 117.05, 107.93; HRMS (ESI) calcd. for $C_{22}H_{16}N_2O$ $[M+H]^+$: 325.1335, found: 325.1342.

(*E*)-6-(pent-1-en-1-yl)-2H-[1,2'-bipyridin]-2-one (5aa)

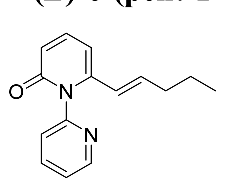

Yellow oil, 44.2 mg, 92%; ^{1}H NMR (400 MHz, $CDCl_3$) δ 8.69 (ddd, *J* = 4.9, 1.9, 0.9 Hz, 1H), 7.91 (td, *J* = 7.7, 1.9 Hz, 1H), 7.46-7.31 (m, 3H), 6.56 (dd, *J* = 9.2, 1.2 Hz, 1H), 6.40 (dt, *J* = 7.0, 0.9 Hz, 1H), 6.36 (s, 0H), 6.21 (dt, *J* = 15.6, 7.0 Hz, 1H), 5.51 (dt, *J* = 15.6, 1.6 Hz, 1H), 1.98 (qd, *J* = 7.2, 1.6 Hz, 2H), 1.33 (q, *J* = 7.3 Hz, 2H), 0.81 (t, *J* = 7.4 Hz, 3H); $^{13}C\{^1H\}$ NMR (101 MHz, $CDCl_3$) δ 163.27, 151.85, 149.90, 146.72, 140.07, 138.60, 137.89, 124.06, 123.96, 123.21, 119.03, 103.51, 34.81, 21.69, 13.46; HRMS (ESI) calcd. for $C_{15}H_{16}N_2O$ $[M+H]^+$: 241.1335, found: 241.1342.

(*E*)-6-(hept-1-en-1-yl)-2H-[1,2'-bipyridin]-2-one (5ab)

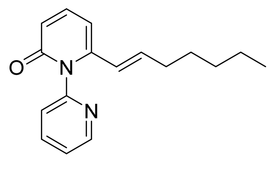

Yellow oil, 48.8 mg, 91%; ^{1}H NMR (400 MHz, $CDCl_3$) δ 8.67 (ddd, *J* = 5.0, 1.9, 0.9 Hz, 1H), 7.90 (td, *J* = 7.7, 1.9 Hz, 1H), 7.45-7.30 (m, 3H), 6.54 (dd, *J* = 9.2, 1.2 Hz, 1H), 6.38 (dd, *J* = 7.1, 1.2 Hz, 1H), 6.20 (dt, *J* = 15.5, 7.0 Hz, 1H), 5.50 (dt, *J* = 15.6, 1.6 Hz, 1H), 1.98 (qd, *J* = 7.1, 1.6 Hz, 2H), 1.38-1.04 (m, 6H), 0.84 (t, *J* = 7.1 Hz, 3H); $^{13}C\{^1H\}$ NMR (101 MHz, $CDCl_3$) δ 163.26, 151.85, 149.87, 146.74, 140.07, 138.59, 138.16, 124.06, 123.95, 123.03, 118.97, 103.50, 32.71, 31.05, 28.09, 22.35, 13.96; HRMS (ESI) calcd. for $C_{17}H_{20}N_2O$ $[M+H]^+$: 269.1648, found: 269.1654.

(*E*)-6-(3-methylbut-1-en-1-yl)-2H-[1,2'-bipyridin]-2-one (5ac)

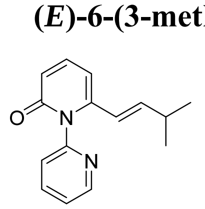

Yellow oil, 41.8 mg, 87%; ^{1}H NMR (400 MHz, $CDCl_3$) δ 8.69 (ddd, *J* = 4.9, 1.9, 0.8 Hz, 1H), 7.91 (td, *J* = 7.7, 1.9 Hz, 1H), 7.46-7.31 (m, 3H), 6.56 (dd, *J* = 9.2, 1.1 Hz, 1H), 6.39 (dd, *J* = 7.1, 1.2 Hz, 1H), 6.17 (dd, *J* = 15.7, 6.8 Hz, 1H), 5.47 (dd, *J* = 15.7, 1.4 Hz, 1H), 2.24 (dqd, *J* = 13.5, 6.8, 1.5 Hz, 1H), 0.89 (d, *J* = 6.8 Hz, 6H); $^{13}C\{^1H\}$ NMR (101 MHz, $CDCl_3$) δ 163.27, 151.88, 149.85, 146.81, 144.47, 140.04, 138.57, 124.09, 123.94, 120.43, 119.01, 103.43, 31.34, 21.64; HRMS (ESI) calcd. for $C_{15}H_{16}N_2O$ $[M+H]^+$: 241.1335, found: 241.1342.

(*E*)-6-(2-cyclohexylvinyl)-2H-[1,2'-bipyridin]-2-one (5ad)

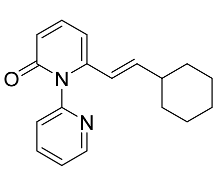

Yellow oil, 47.7 mg, 85%; ^{1}H NMR (400 MHz, $CDCl_3$) δ 8.73-8.62 (m, 1H), 7.90 (td, *J* = 7.7, 1.8 Hz, 1H), 7.47-7.25 (m, 3H), 6.54 (d, *J* = 9.2 Hz, 1H), 6.38 (d, *J* = 7.0 Hz, 1H), 6.13 (dd, *J* = 15.7, 6.8 Hz, 1H), 5.46 (d, *J* = 15.7 Hz, 1H), 1.90 (dtd, *J* = 11.2, 7.2, 3.3 Hz, 1H), 1.72-1.50 (m, 5H), 1.28-1.04 (m, 3H), 0.96 (td, *J* = 11.9, 3.0 Hz, 2H); $^{13}C\{^1H\}$ NMR (101 MHz, $CDCl_3$) δ 163.29, 151.88, 149.84, 146.96, 143.35, 140.05, 138.55, 124.07, 123.95, 120.77, 118.91, 103.41, 77.41, 77.09, 76.77, 40.77, 32.08, 25.86, 25.61; HRMS [ESI] calcd. for $C_{18}H_{20}N_2O$ $(M+H)^+$: 281.1648, found: 281.1656.

(*E*)-6-(7-chlorohept-1-en-1-yl)-2H-[1,2'-bipyridin]-2-one (5ae)

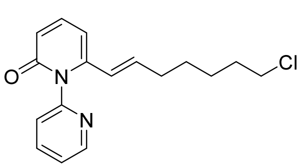

Yellow oil, 52.7 mg, 87%; ^{1}H NMR (400 MHz, $CDCl_3$) δ 8.68 (dd, *J* = 5.0, 1.8 Hz, 1H), 7.92 (td, *J* = 7.7, 1.9 Hz, 1H), 7.47-7.31 (m, 3H), 6.56 (dd, *J* = 9.3, 1.1 Hz, 1H), 6.39 (dd, *J* = 7.1, 1.1 Hz, 1H), 6.19 (dt, *J* = 15.6, 7.0 Hz, 1H), 5.52 (dt, *J* = 15.6, 1.6 Hz, 1H), 3.49 (t, *J* = 6.6 Hz, 2H), 2.02 (tdd, *J* = 7.1, 5.2, 2.5 Hz, 2H), 1.79-1.62 (m, 2H), 1.41-1.28 (m, 4H); $^{13}C\{^1H\}$ NMR (101 MHz, $CDCl_3$) δ 163.26, 151.79, 149.91, 146.53, 140.09, 138.68, 137.46, 124.08, 124.03, 123.41, 119.14, 103.63, 44.89, 32.58, 32.23, 27.72, 26.13; HRMS (ESI) calcd. for $C_{17}H_{19}ClN_2O$ $[M+H]^+$: 303.1259, found: 303.1266.

6-vinyl-2H-[1,2'-bipyridin]-2-one (5af)

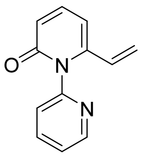

Yellow oil, 29.7 mg, 75%; ^{1}H NMR (400 MHz, $CDCl_3$) δ 8.69 (ddd, *J* = 4.9, 1.9, 0.9 Hz, 1H), 7.92 (td, *J* = 7.7, 1.9 Hz, 1H), 7.46-7.34 (m, 3H), 6.62 (dd, *J* = 9.3, 1.2 Hz, 1H), 6.48 (dd, *J* = 7.0, 1.1 Hz, 1H), 5.90 (dd, *J* = 17.2, 10.9 Hz, 1H), 5.73 (dd, *J* = 17.2, 1.0 Hz, 1H), 5.23 (dd, *J* = 10.9, 1.0 Hz, 1H); $^{13}C\{^1H\}$ NMR (101 MHz, $CDCl_3$) δ 163.05, 151.55, 149.94, 146.27, 139.89, 138.62, 130.06, 124.08, 124.05, 120.25, 119.89, 103.86; HRMS (ESI) calcd. for $C_{12}H_{10}N_2O$ $[M+H]^+$: 199.0866, found: 199.0874.

6-(1-phenylvinyl)-2H-[1,2'-bipyridin]-2-one (5ag)

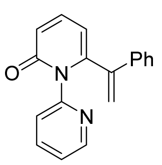

Yellow oil, 43.9 mg, 80%; ^{1}H NMR (400 MHz, $CDCl_3$) δ 8.36 (ddd, *J* = 4.8, 1.9, 0.9 Hz, 1H), 7.47 (dd, *J* = 9.3, 6.8 Hz, 1H), 7.33 (td, *J* = 7.7, 1.9 Hz, 1H), 7.23-7.02 (m, 4H), 6.89-6.81 (m, 2H), 6.79 (dt, *J* = 7.9, 1.0 Hz, 1H), 6.68 (dd, *J* = 9.4, 1.3 Hz, 1H), 6.43 (dd, *J* = 6.7, 1.3 Hz, 1H), 5.57 (d, *J* = 1.0 Hz, 1H), 5.41 (d, *J* = 1.0 Hz, 1H); $^{13}C\{^1H\}$ NMR (101 MHz, $CDCl_3$) δ 163.14, 151.19, 149.07, 148.55, 144.61, 139.97, 138.49, 136.59, 128.10, 128.04, 126.47, 124.92, 123.01, 121.03, 119.24, 108.38; HRMS (ESI) calcd. for $C_{18}H_{14}N_2O$ $[M+H]^+$: 275.1179, found: 275.1187.

6-(1-(7-methoxynaphthalen-2-yl)vinyl)-2H-[1,2'-bipyridin]-2-one (5ah)

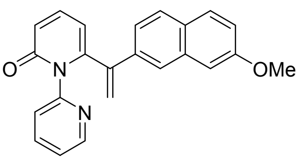

Brown oil, 56.7 mg, 80%; ^{1}H NMR (400 MHz, $CDCl_3$) δ 8.34 (ddd, *J* = 4.9, 1.9, 0.9 Hz, 1H), 7.56 (d, *J* = 9.0 Hz, 1H), 7.55-7.44 (m, 2H), 7.23 (d, *J* = 1.8 Hz, 1H), 7.19-7.08 (m, 2H), 7.04 (d, *J* = 2.5 Hz, 1H), 7.02-6.92 (m, 2H), 6.79-6.68 (m, 2H), 6.47 (dd, *J* = 6.7, 1.3 Hz, 1H), 5.59 (d, *J* = 1.0 Hz, 1H), 5.51 (d, *J* = 1.1 Hz, 1H), 3.91 (s, 3H); $^{13}C\{^1H\}$ NMR (101 MHz, $CDCl_3$) δ 163.20, 157.98, 151.25, 149.23, 148.58, 144.46, 140.00, 136.53, 134.09, 133.70, 129.54, 128.24, 126.65, 125.68, 124.76, 124.68, 123.02, 121.06, 119.19, 118.96, 108.37, 105.50, 55.29; HRMS (ESI) calcd. for $C_{23}H_{18}N_2O_2$ $[M+H]^+$: 355.1441, found: 355.1449.

(*E*)-6-(but-2-en-2-yl)-2H-[1,2'-bipyridin]-2-one (5ai)

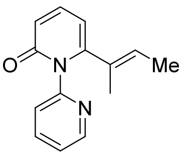

Yellow oil, 33.9 mg, 75%; ^{1}H NMR (400 MHz, $CDCl_3$) δ 8.60 (dd, *J* = 5.3, 1.9 Hz, 1H), 7.83 (td, *J* = 7.7, 1.9 Hz, 1H), 7.41-7.30 (m, 3H), 6.57 (dd, *J* = 9.3, 1.2 Hz, 1H), 6.09 (dd, *J* = 6.9, 1.2 Hz, 1H), 5.67 (qq, *J* = 6.9, 1.5 Hz, 1H), 1.51 (dq, *J* = 6.9, 1.2 Hz, 3H), 1.39 (t, *J* = 1.3 Hz, 3H); $^{13}C\{^1H\}$ NMR (101 MHz, $CDCl_3$) δ 163.36, 152.70, 152.18, 149.20, 140.26, 137.70, 131.43, 129.45, 124.48, 123.44, 119.42, 105.82, 16.08, 13.59; HRMS (ESI) calcd. for $C_{14}H_{14}N_2O$ $[M+H]^+$: 227.1179, found: 227.1187.

(*E*)-6-(pent-2-en-2-yl)-2H-[1,2'-bipyridin]-2-one (5aj)

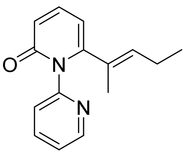

Yellow oil, 38.4 mg, 80%; ^{1}H NMR (400 MHz, $CDCl_3$) δ 8.56 (dd, *J* = 5.3, 2.0 Hz, 1H), 7.80 (dt, *J* = 7.8, 3.9 Hz, 1H), 7.42-7.26 (m, 3H), 6.54 (d, *J* = 9.3 Hz, 1H), 6.08 (d, *J* = 6.9 Hz, 1H), 5.54-5.44 (m, 1H), 1.86 (p, *J* = 7.5 Hz, 2H), 1.43 (s, 3H), 0.75 (t, *J* = 7.5 Hz, 3H); $^{13}C\{^1H\}$ NMR (101 MHz, $CDCl_3$) δ 163.33, 152.60, 152.25, 149.12, 140.22, 137.64, 136.77, 129.86, 124.49, 123.43, 119.33, 105.69, 21.21, 16.37, 13.09; HRMS (ESI) calcd. for $C_{15}H_{16}N_2O$ $[M+H]^+$: 241.1335, found: 241.1342.

(*E*)-6-(2,6-dimethylhepta-1,5-dien-1-yl)-2H-[1,2'-bipyridin]-2-one / (*Z*)-6-(2,6-dimethylhepta-1,5-dien-1-yl)-2H-[1,2'-bipyridin]-2-one = 78 / 22 (5ak)

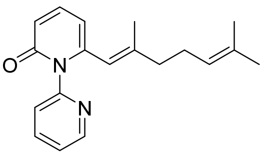

Yellow oil, 50.1 mg, 85%; ^{1}H NMR (400 MHz, $CDCl_3$) δ 8.63 (dd, *J* = 4.9, 2.0 Hz, 1H), 7.84 (td, *J* = 7.7, 2.0 Hz, 1H), 7.43-7.30 (m, 2H), 7.23 (dd, *J* = 14.2, 7.9 Hz, 1H), 6.54 (dd, *J* = 9.3, 1.2 Hz, 1H), 6.11 (dd, *J* = 6.9, 1.2 Hz, 1H), 5.44 (s, 0.2H), 5.40 (s, 0.8H), 5.05 (ddq, *J* = 8.4, 5.4, 1.5 Hz, 0.2H), 4.84 (tq, *J* = 5.4, 1.5 Hz, 0.8H), 2.25-1.99 (m, 1H), 1.94-1.74 (m, 5H), 1.69 (d, *J* = 1.4 Hz, 0.6H), 1.60 (dd, *J* = 9.9, 1.7 Hz, 4H), 1.50 (d, *J* = 1.3 Hz, 2.5H); $^{13}C\{^1H\}$ NMR (101 MHz, $CDCl_3$) δ 163.56, 152.24, 149.71, 145.73, 144.94, 139.82, 138.42, 132.02, 123.76, 123.65, 123.08, 118.88, 118.50, 107.29, 39.61, 26.12, 25.66, 18.52, 17.62; HRMS (ESI) calcd. for $C_{19}H_{22}N_2O$ $[M+H]^+$: 295.1805, found: 295.1813.

6-(cyclohex-1-en-1-yl)-2H-[1,2'-bipyridin]-2-one (5al)

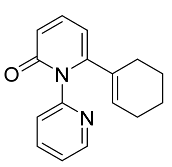

Yellow oil, 45.9 mg, 91%; ^{1}H NMR (400 MHz, $CDCl_3$) δ 8.62-8.55 (m, 1H), 7.82 (td, *J* = 7.7, 1.9 Hz, 1H), 7.40-7.28 (m, 3H), 6.55 (dd, *J* = 9.3, 1.2 Hz, 1H), 6.08 (dd, *J* = 6.8, 1.2 Hz, 1H), 5.71 (tt, *J* = 3.8, 1.7 Hz, 1H), 1.90 (dh, *J* = 8.5, 3.3 Hz, 2H), 1.80-1.67 (m, 2H), 1.40-1.27 (m, 4H); $^{13}C\{^{1}H\}$ NMR (101 MHz, $CDCl_3$) δ 163.36, 152.27, 151.68, 149.11, 140.26, 137.65, 133.53, 131.63, 124.43, 123.47, 119.36, 105.72, 28.71, 25.12, 22.25, 21.27; HRMS (ESI) calcd. for $C_{16}H_{16}N_2O$ $[M+H]^+$: 253.1335, found: 253.1342.

6-(3,4,5-trihydroxycyclohex-1-en-1-yl)-2H-[1,2'-bipyridin]-2-one (5am)

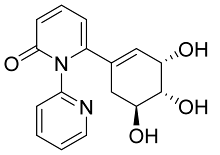

Brown oil, 37.8 mg, 63%; ^{1}H NMR (400 MHz, MeOD) δ 8.58 (dd, *J* = 5.0, 1.8 Hz, 1H), 8.01 (td, *J* = 7.7, 1.9 Hz, 1H), 7.65 (dd, *J* = 9.2, 6.9 Hz, 1H), 7.57-7.44 (m, 2H), 6.62 (dd, *J* = 9.3, 1.2 Hz, 1H), 6.43 (dd, *J* = 7.0, 1.2 Hz, 1H), 5.74 (dd, *J* = 4.1, 2.1 Hz, 1H), 4.07 (t, *J* = 4.3 Hz, 1H), 3.71-3.59 (m, 1H), 3.37 (s, 1H), 3.27 (dd, *J* = 8.5, 4.3 Hz, 1H), 2.32 (dd, *J* = 17.5, 5.1 Hz, 1H); $^{13}C\{^{1}H\}$ NMR (101 MHz, MeOD) δ 163.84, 151.16, 149.85, 148.82, 141.74, 138.93, 132.81, 131.85, 124.63, 124.37, 118.72, 107.20, 71.77, 66.21, 65.82, 35.87; HRMS (ESI) calcd. for $C_{16}H_{16}N_2O_4$ $[M+H]^+$: 301.1183, found: 301.1189.

(*S*)-6-(4-(prop-1-en-2-yl)cyclohex-1-en-1-yl)-2H-[1,2'-bipyridin]-2-one (5an)

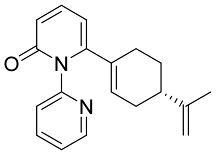

Yellow oil, 48.2 mg, 83%; ^{1}H NMR (400 MHz, $CDCl_3$) δ 8.64-8.57 (m, 1H), 7.84 (td, *J* = 7.7, 1.9 Hz, 1H), 7.42-7.31 (m, 3H), 6.57 (dd, *J* = 9.3, 1.2 Hz, 1H), 6.10 (dd, *J* = 6.8, 1.3 Hz, 1H), 5.77 (dt, *J* = 4.7, 2.1 Hz, 1H), 4.63 (d, *J* = 41.2 Hz, 2H), 2.09 (dd, *J* = 14.4, 9.5 Hz, 1H), 2.01-1.56 (m, 8H), 1.22-1.06 (m, 1H); $^{13}C\{^{1}H\}$ NMR (101 MHz, $CDCl_3$) δ 163.34, 152.26, 151.31, 149.18, 148.70, 140.18, 137.69, 133.27, 131.06, 124.43, 123.50, 119.52, 109.00, 105.79, 39.73, 30.54, 28.96, 27.06, 20.69; HRMS (ESI) calcd. for $C_{19}H_{20}N_2O$ $[M+H]^+$: 293.1648, found: 293.1654.

6-((1*E*,3*E*)-penta-1,3-dien-1-yl)-2H-[1,2'-bipyridin]-2-one (5ao)

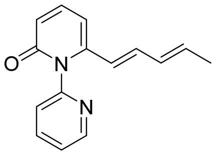

Yellow oil, 35.3 mg, 74%; ^{1}H NMR (400 MHz, $CDCl_3$) δ 8.70 (ddd, *J* = 4.9, 1.9, 0.8 Hz, 1H), 7.92 (td, *J* = 7.7, 1.9 Hz, 1H), 7.48-7.32 (m, 3H), 6.74-6.62 (m, 1H), 6.62-6.44 (m, 2H), 6.01-5.82 (m, 2H), 5.50 (d, *J* = 15.3 Hz, 1H), 1.81-1.72 (m, 3H); $^{13}C\{^{1}H\}$ NMR (101 MHz, $CDCl_3$) δ 163.38, 151.72, 149.99, 146.47, 139.96, 138.74, 135.10, 134.90, 130.88, 124.13, 124.05, 121.37, 119.02, 103.33, 18.43; HRMS (ESI) calcd. for $C_{15}H_{14}N_2O$ $[M+H]^+$: 239.1179, found: 239.1188.

6-((1*E*,3*E*)-4-phenylbuta-1,3-dien-1-yl)-2H-[1,2'-bipyridin]-2-one (5ap)

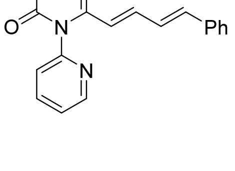

Brown oil, 48.7 mg, 81%; ^{1}H NMR (400 MHz, $CDCl_3$) δ 8.73 (dd, *J* = 5.0, 1.8 Hz, 1H), 7.94 (td, *J* = 7.7, 1.9 Hz, 1H), 7.51-7.16 (m, 9H), 6.88 (dd, *J* = 15.2, 10.2 Hz, 1H), 6.77-6.52 (m, 4H), 5.74 (d, *J* = 15.2 Hz, 1H); $^{13}C\{^{1}H\}$ NMR (101 MHz, $CDCl_3$) δ

163.25, 151.71, 150.08, 146.10, 139.83, 138.79, 136.57, 136.38, 134.77, 128.72, 128.46, 127.76, 126.76, 124.20, 124.12, 123.88, 119.48, 103.66; HRMS (ESI) calcd. for $C_{20}H_{16}N_2O$ $[M+H]^+$: 301.1335, found: 301.1342.

6-((1*E*,3*E*)-6-chloro-4-methylhexa-1,3-dien-1-yl)-2H-[1,2'-bipyridin]-2-one (5aq)

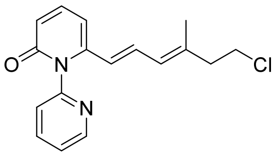

Yellow oil, 39.1 mg, 65%; ^{1}H NMR (400 MHz, $CDCl_3$) δ 8.66 (ddd, *J* = 4.9, 2.0, 0.8 Hz, 1H), 7.95-7.83 (m, 1H), 7.46-7.35 (m, 3H), 7.34-7.26 (m, 1H), 6.58 (dt, *J* = 9.2, 0.9 Hz, 1H), 6.21 (dd, *J* = 6.9, 1.1 Hz, 1H), 5.59 (s, 1H), 5.50 (s, 1H), 3.52 (t, *J* = 7.1 Hz, 2H), 2.40 (td, *J* = 7.1, 1.1 Hz, 2H), 1.93 (d, *J* = 1.4 Hz, 3H); $^{13}C\{^1H\}$ NMR (101 MHz, $CDCl_3$) δ 163.53, 152.20, 150.01, 145.19, 139.72, 138.68, 134.61, 130.35, 126.73, 123.88, 123.76, 121.82, 119.29, 107.51, 43.04, 42.58, 19.53; HRMS (ESI) calcd. for $C_{17}H_{17}ClN_2O$ $[M+H]^+$: 301.1102, found: 301.1109.

6-((1*E*,3*E*)-4,8-dimethylnona-1,3,7-trien-1-yl)-2H-[1,2'-bipyridin]-2-one / 6-((1*E*,3*Z*)-4,8-dimethylnona-1,3,7-trien-1-yl)-2H-[1,2'-bipyridin]-2-one = 52 / 43 (5ar)

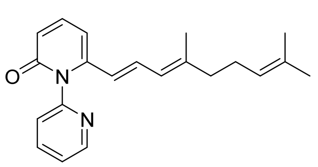

Yellow oil, 55.1 mg, 86%; ^{1}H NMR (400 MHz, $CDCl_3$) δ 8.69 (dd, *J* = 4.8, 2.3 Hz, 1H), 7.90 (tt, *J* = 7.7, 2.0 Hz, 1H), 7.46-7.30 (m, 3H), 6.92 (ddd, *J* = 15.1, 11.2, 5.4 Hz, 1H), 6.60-6.43 (m, 2H), 5.69 (d, *J* = 11.2 Hz, 1H), 5.49 (dd, *J* = 15.2, 7.1 Hz, 1H), 5.10 (tdd, *J* = 6.9, 3.0, 1.4 Hz, 0.52H), 5.02 (dddt, *J* = 6.9, 5.3, 3.2, 1.6 Hz, 0.43H), 2.22 (dd, *J* = 8.9, 6.6 Hz, 1H), 2.16-2.01 (m, 3H), 1.83-1.74 (m, 3H), 1.74-1.63 (m, 3H), 1.59 (dd, *J* = 18.9, 1.3 Hz, 3H); $^{13}C\{^1H\}$ NMR (101 MHz, $CDCl_3$) δ 163.32, 151.88, 149.99, 146.92, 145.21, 145.02, 139.85, 138.67, 132.42, 132.01, 131.26, 130.98, 125.23, 124.38, 124.15, 123.98, 123.44, 121.45, 121.25, 118.76, 103.26, 103.16, 40.11, 32.77, 26.80, 26.36, 25.73, 25.67, 24.15, 17.75, 17.68; HRMS (ESI) calcd. for $C_{21}H_{24}N_2O$ $[M+H]^+$: 321.1961, found: 321.1969.

(*E*)-2-(2-(2-oxo-2H-[1,2'-bipyridin]-6-yl)vinyl)naphthalene-1,4-dione (5as)

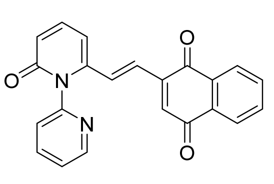

Brown oil, 60.9 mg, 86%; ^{1}H NMR (400 MHz, $CDCl_3$) δ 8.68 (ddd, *J* = 4.9, 1.9, 0.9 Hz, 1H), 8.14 (dd, *J* = 8.0, 1.7 Hz, 1H), 7.94 (td, *J* = 7.7, 1.9 Hz, 1H), 7.90 (s, 1H), 7.63 (ddd, *J* = 8.7, 7.1, 1.7 Hz, 1H), 7.48-7.32 (m, 5H), 7.00 (d, *J* = 15.8 Hz, 1H), 6.79 (dd, *J* = 15.8, 0.6 Hz, 1H), 6.62-6.52 (m, 2H); $^{13}C\{^1H\}$ NMR (126 MHz, $CDCl_3$) δ 181.84, 175.92, 163.24, 155.50, 155.10, 151.60, 150.01, 146.78, 139.96, 138.83, 133.84, 126.18, 125.54, 125.11, 124.44, 124.21, 124.11, 123.95, 120.27, 119.87, 118.05, 103.99; HRMS (ESI) calcd. for $C_{22}H_{14}N_2O_3$ $[M+H]^+$: 355.1077, found: 355.1084.

6-((1*E*,3*E*,5*E*,7*E*)-2,6-dimethyl-8-(2,6,6-trimethylcyclohex-1-en-1-yl)octa-

1,3,5,7-tetraen-1-yl)-2H-[1,2'-bipyridin]-2-one (5at)

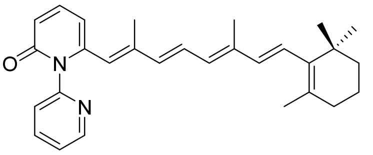

Yellow oil, 61.4 mg, 72%; ^{1}H NMR (400 MHz, $CDCl_3$) δ 8.74-8.54 (m, 1H), 7.87 (dtd, *J* = 7.7, 3.9, 2.0 Hz, 1H), 7.50-7.32 (m, 2H), 7.32-7.22 (m, 1H), 6.86-6.50 (m, 3H), 6.33-5.88 (m, 4H), 5.50 (d, *J* = 31.9 Hz, 1H), 2.10-1.87 (m, 7H), 1.85-1.78 (m, 1H), 1.77-1.56 (m, 5H), 1.53-1.37 (m, 2H), 1.03 (d, *J* = 8.6 Hz, 6H); $^{13}C\{^{1}H\}$ NMR (101 MHz, $CDCl_3$) δ 163.41, 152.04, 149.90, 149.83, 145.18, 139.50, 138.59, 137.30, 135.50, 129.89, 129.53, 129.17, 128.06, 127.84, 123.75, 122.87, 120.97, 119.35, 108.96, 108.07, 39.58, 34.24, 33.07, 28.97, 21.73, 20.81, 19.21, 14.69, 12.82; HRMS (ESI) calcd. for $C_{29}H_{34}N_2O$ $[M+H]^+$: 427.2744, found: 427.2751.

6-((1*E*,3*E*,5*E*,7*E*,9*E*)-4,8-dimethyl-10-(2,6,6-trimethylcyclohex-1-en-1-yl)deca-1,3,5,7,9-pentaen-1-yl)-2H-[1,2'-bipyridin]-2-one (5au)

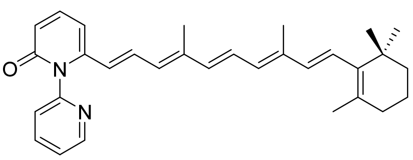

Yellow oil, 58.8 mg, 65%; ^{1}H NMR (400 MHz, $CDCl_3$) δ 8.77-8.63 (m, 1H), 7.98-7.86 (m, 1H), 7.49-7.32 (m, 4H), 6.88-6.64 (m, 1H), 6.57 (d, *J* = 9.6 Hz, 2H), 6.29-6.04 (m, 4H), 5.95 (d, *J* = 11.7 Hz, 1H), 5.61 (d, *J* = 15.0 Hz, 1H), 1.98 (t, *J* = 8.1 Hz, 7H), 1.79-1.68 (m, 3H), 1.62 (ddd, *J* = 13.2, 9.9, 6.2 Hz, 3H), 1.54-1.43 (m, 2H), 1.04 (d, *J* = 8.4 Hz, 6H); $^{13}C\{^{1}H\}$ NMR (101 MHz, $CDCl_3$) δ 163.29, 151.78, 150.06, 146.59, 140.52, 139.75, 138.75, 137.48, 136.08, 130.87, 130.24, 130.09, 129.70, 127.65, 127.23, 124.20, 124.05, 123.11, 119.07, 118.63, 106.47, 103.68, 42.21, 39.60, 34.27, 33.11, 28.98, 21.78, 19.24, 13.04, 12.84; HRMS (ESI) calcd. for $C_{31}H_{36}N_2O$ $[M+H]^+$: 453.2900, found: 453.2908.

(E)-6-(4-phenylbut-1-en-3-yn-1-yl)-2H-[1,2'-bipyridin]-2-one (5av)

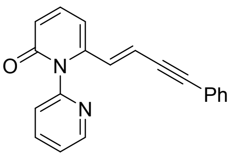

Brown oil, 40.0 mg, 67%; ^{1}H NMR (400 MHz, $CDCl_3$) δ 8.74 (dd, *J* = 5.2, 1.9 Hz, 1H), 7.96 (td, *J* = 7.7, 2.0 Hz, 1H), 7.51-7.34 (m, 6H), 7.35-7.32 (m, 2H), 6.66 (dd, *J* = 9.3, 1.1 Hz, 1H), 6.56 (d, *J* = 7.0 Hz, 1H), 6.35 (d, *J* = 15.9 Hz, 1H), 6.12 (d, *J* = 15.9 Hz, 1H); $^{13}C\{^{1}H\}$ NMR (101 MHz, $CDCl_3$) δ 163.12, 150.17, 145.01, 139.63, 138.84, 132.48, 131.64, 128.89, 128.54, 128.40, 124.28, 124.15, 120.86, 117.10, 114.00, 104.23, 94.97, 87.52; HRMS (ESI) calcd. for $C_{20}H_{14}N_2O$ $[M+H]^+$: 299.1179, found: 299.1187.

8. References:

1. R. Jain, F. Roschangar and M. A. Ciufolini, *Tetrahedron Lett.* **1995,** *36,* 3307-3310.
2. N. T. Tzvetkov, S. Hinz, P. Küppers, M. Gastreich and C. E. Müller, *J. Med. Chem.* **2014,** *57,* 6679-6703.
3. J. Zhang, X. Liu and R. Wang, *Chem. Eur. J.* **2014,** *20,* 4911-4915.
4. L. Coudray, R. M. De Figueiredo, S. Duez, S. Cortial and J. Dubois, *J. Enzyme. Inhib. Med. Chem.* **2009,** *24,* 972-985.
5. M. Gucma, W. M. Gołębiewski and A. K. Michalczyk, *J. Mol. Struct.* **2014,** *1060,* 223-232.
6. J. Van Gompel and G. B. Schuster, *J. Org. Chem.* **1987,** *52,* 1465-1468.
7. R. Odani, K. Hirano, T. Satoh and M. Miura, *Angew. Chem. Int. Ed.* **2014,** *53,* 10784-10788.
8. A. L. Crombie, J. L. Kane, K. M. Shea and R. L. Danheiser, *J. Org. Chem.* **2004,** *69,* 8652-8667.
9. W. Miura, K. Hirano and M. Miura, *J. Org. Chem.* **2017,** *82,* 5337-5344.

9. Copies of ^{1}H and ^{13}C{^{1}H} NMR Spectra

^{1}H and ^{13}C{^{1}H} NMR spectra of compound 1a' in $CDCl_3$

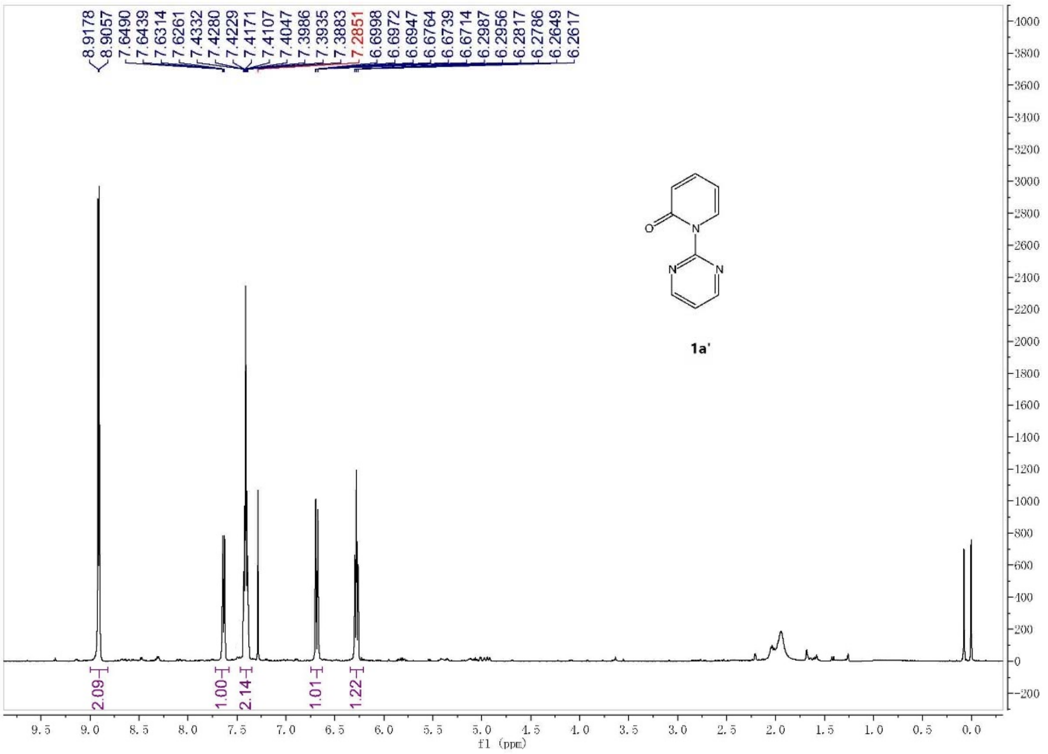

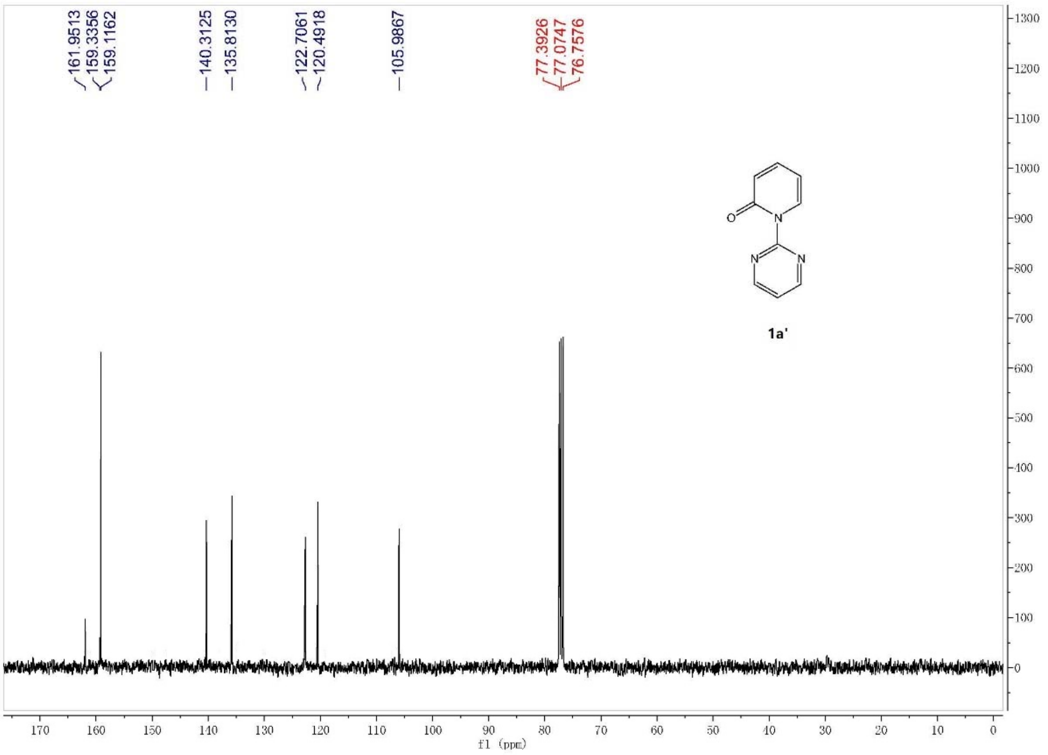

^{1}H and $^{13}C\{^{1}H\}$ NMR spectra of compound 1j in $CDCl_3$

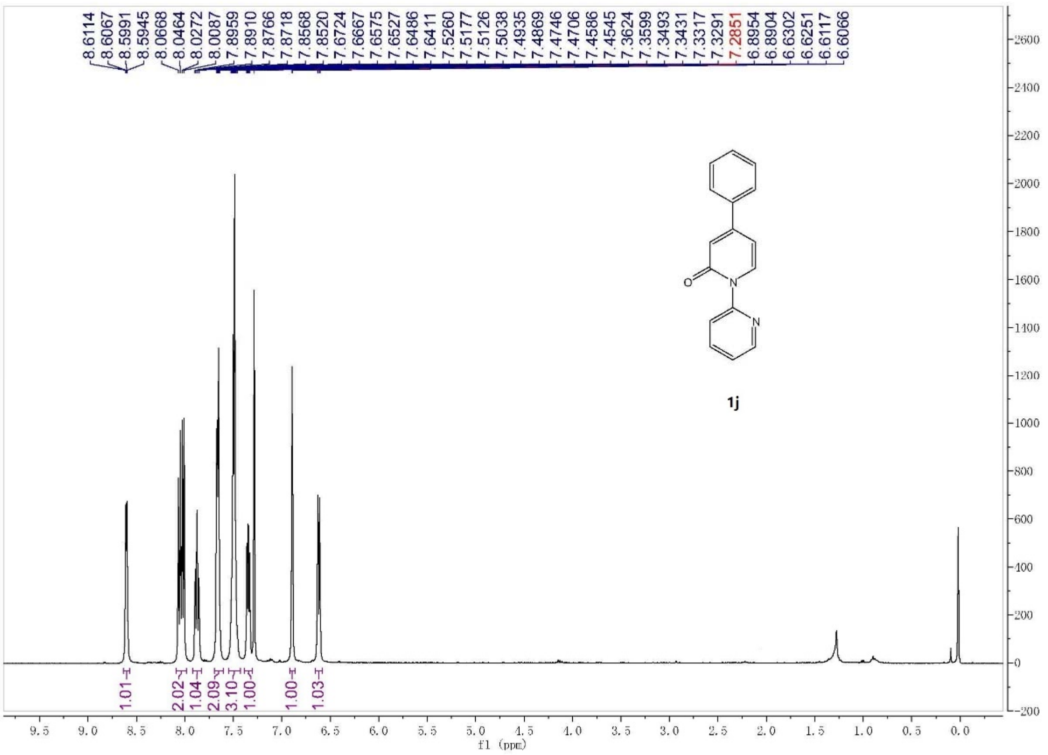

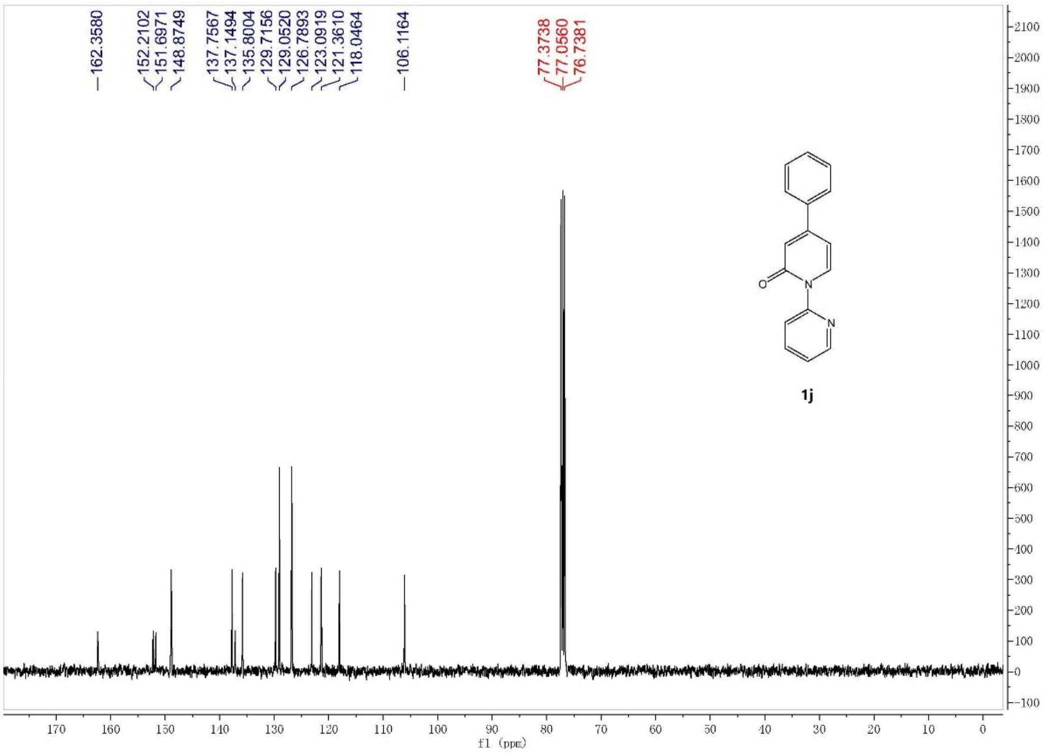

^{1}H and $^{13}C\{^{1}H\}$ NMR spectra of compound 1r in $CDCl_3$

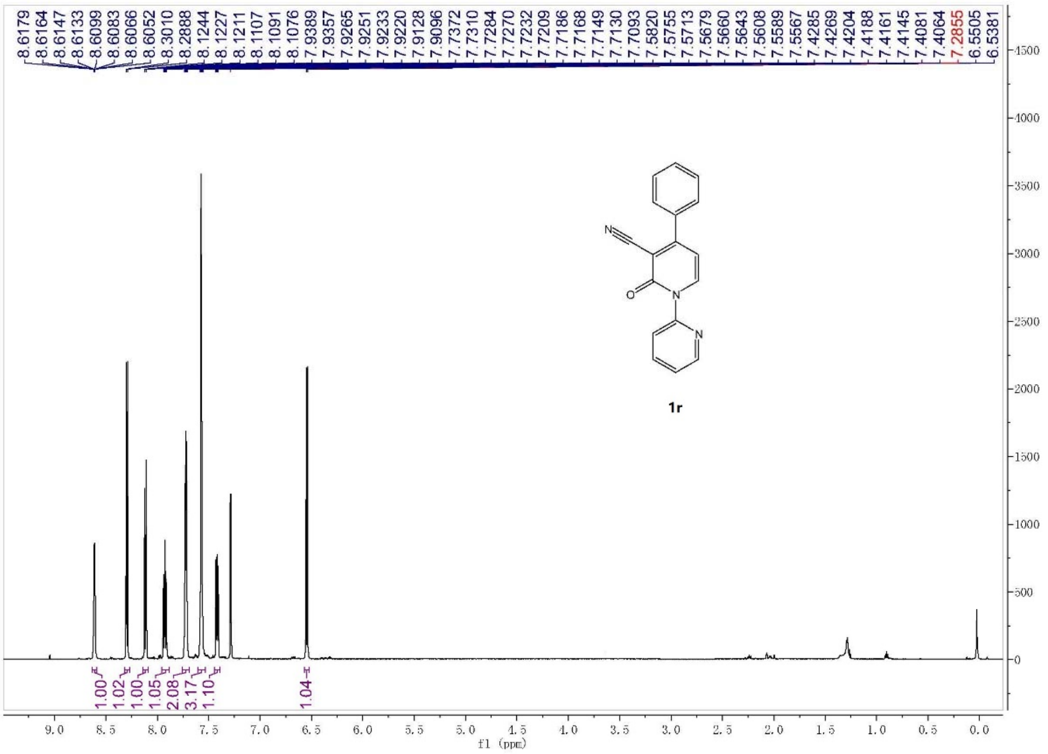

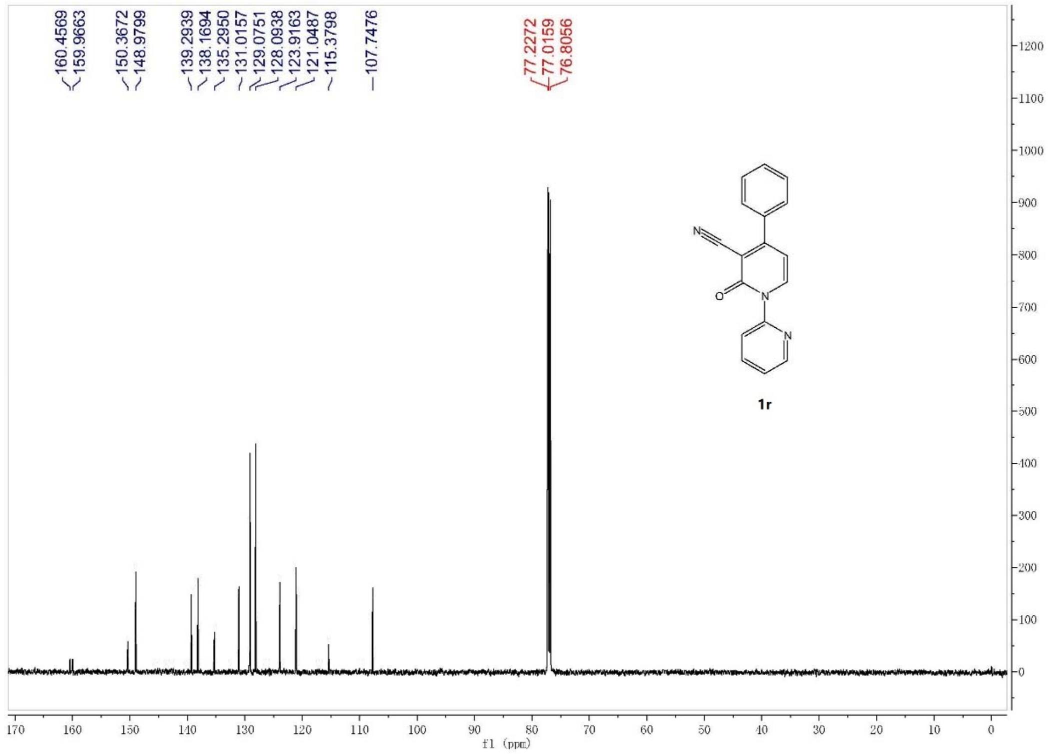

^{1}H and $^{13}C\{^{1}H\}$ NMR spectra of compound 2z in $CDCl_3$

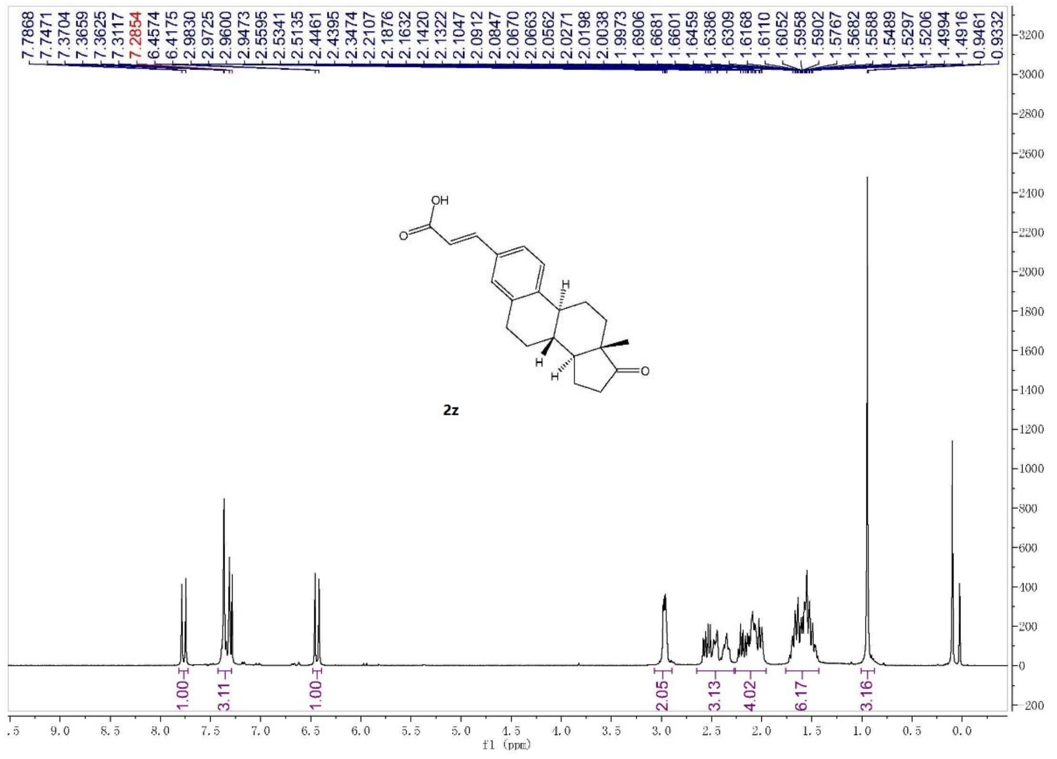

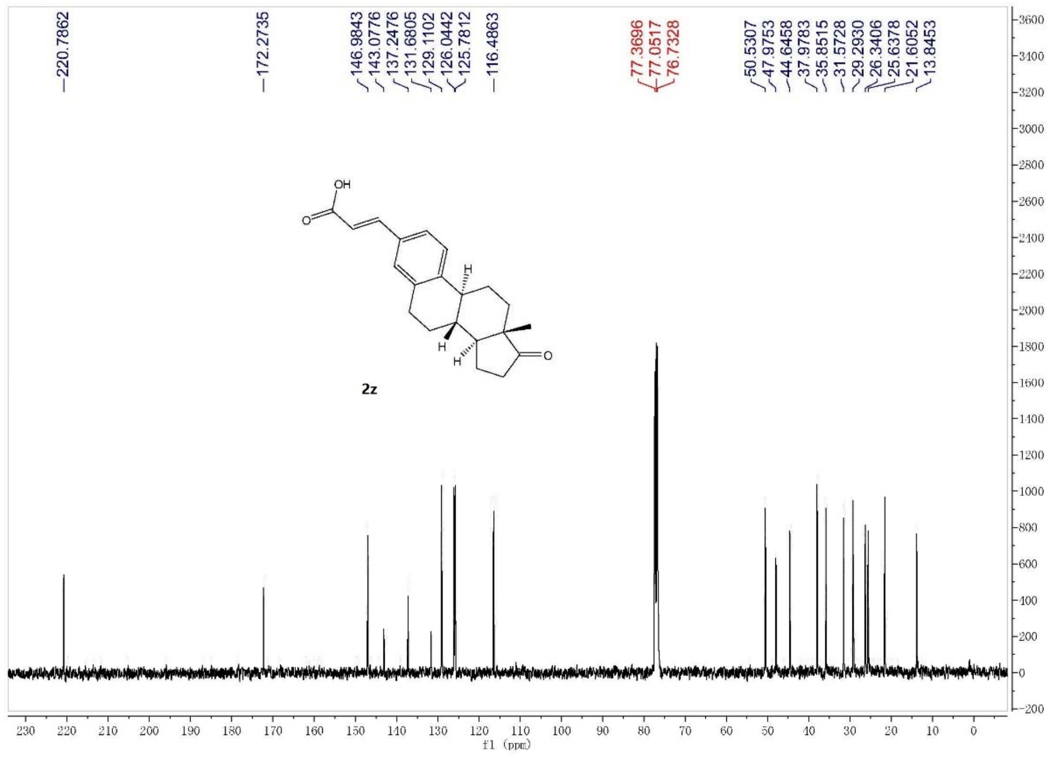

^{1}H and $^{13}C\{^{1}H\}$ NMR spectra of compound 3a'a in $CDCl_3$

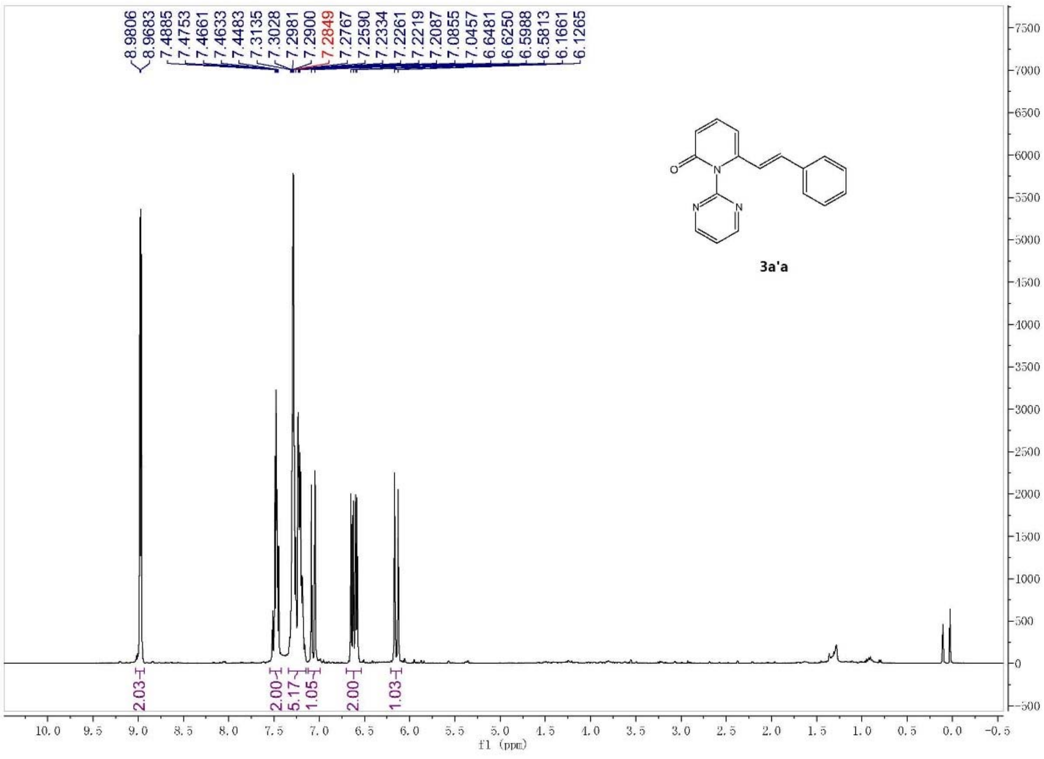

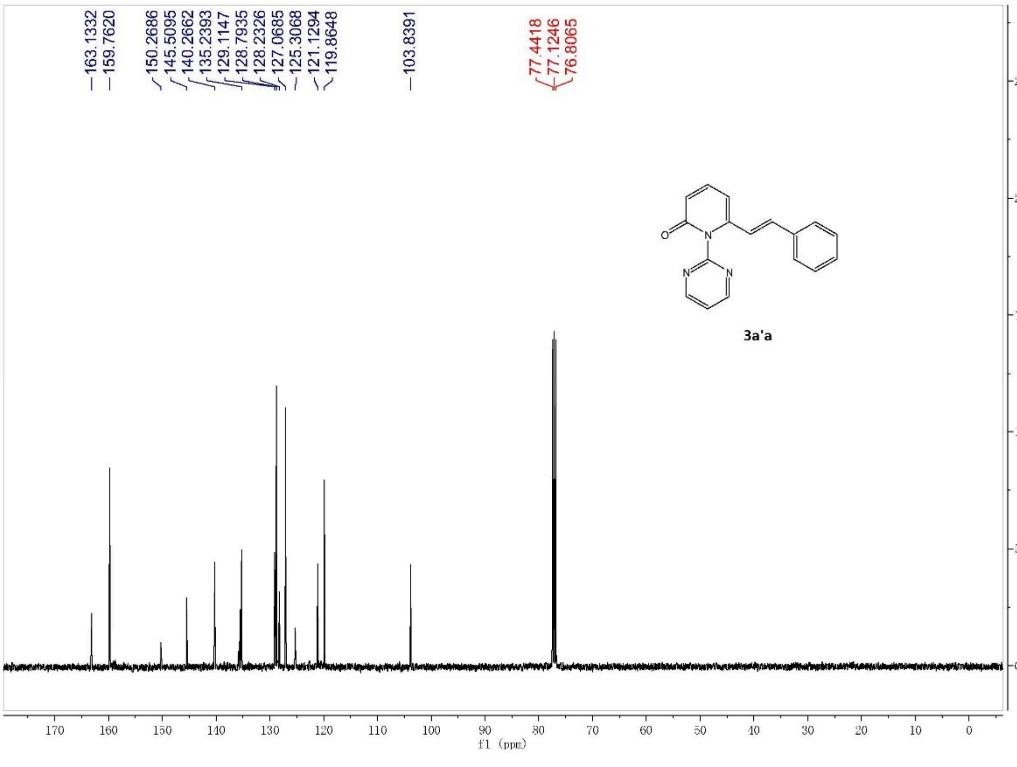

^{1}H and $^{13}C\{^{1}H\}$ NMR spectra of compound 3aa in $CDCl_3$

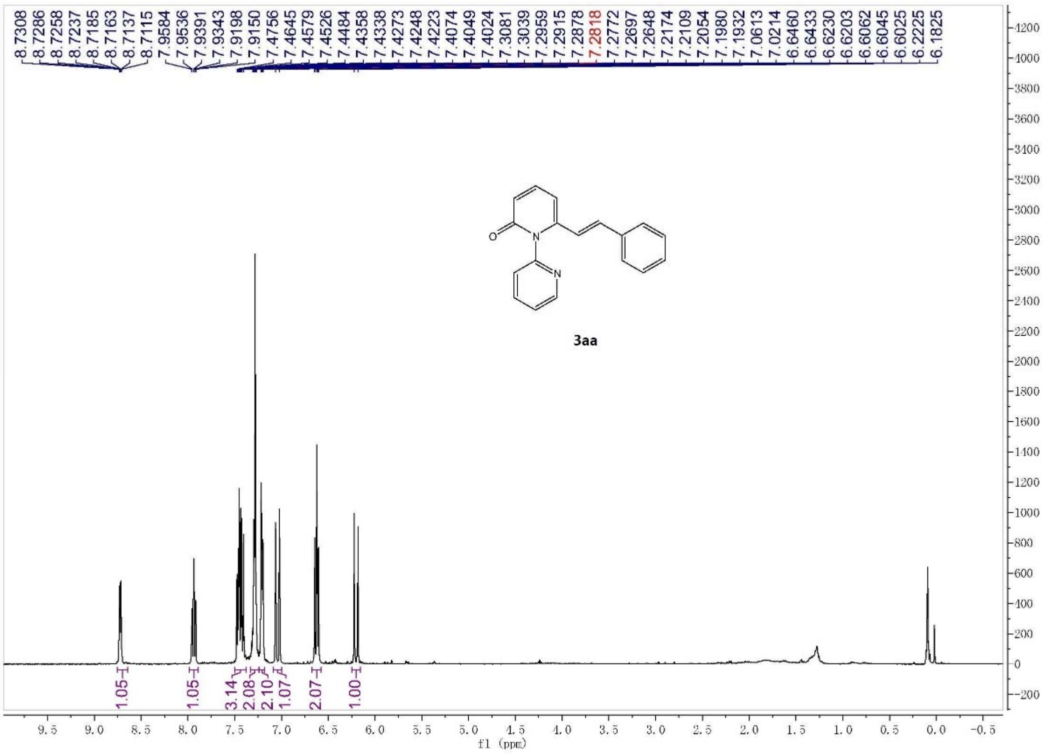

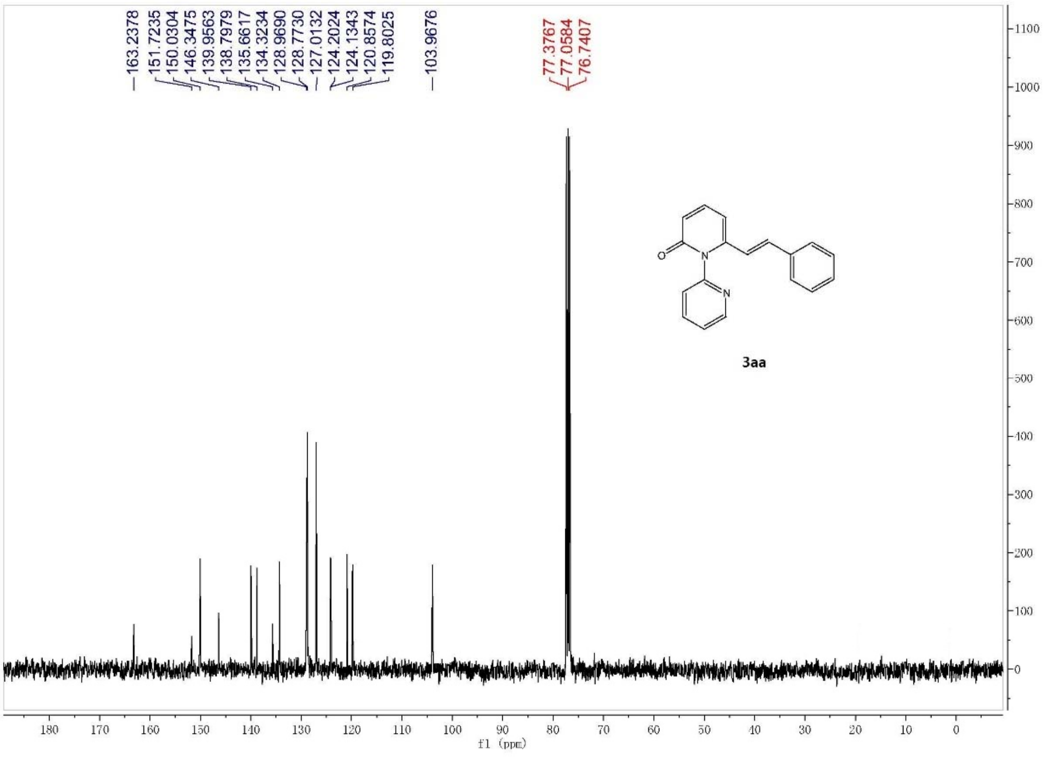

^1H and $^{13}C\{^1H\}$ NMR spectra of compound 3ab in $CDCl_3$

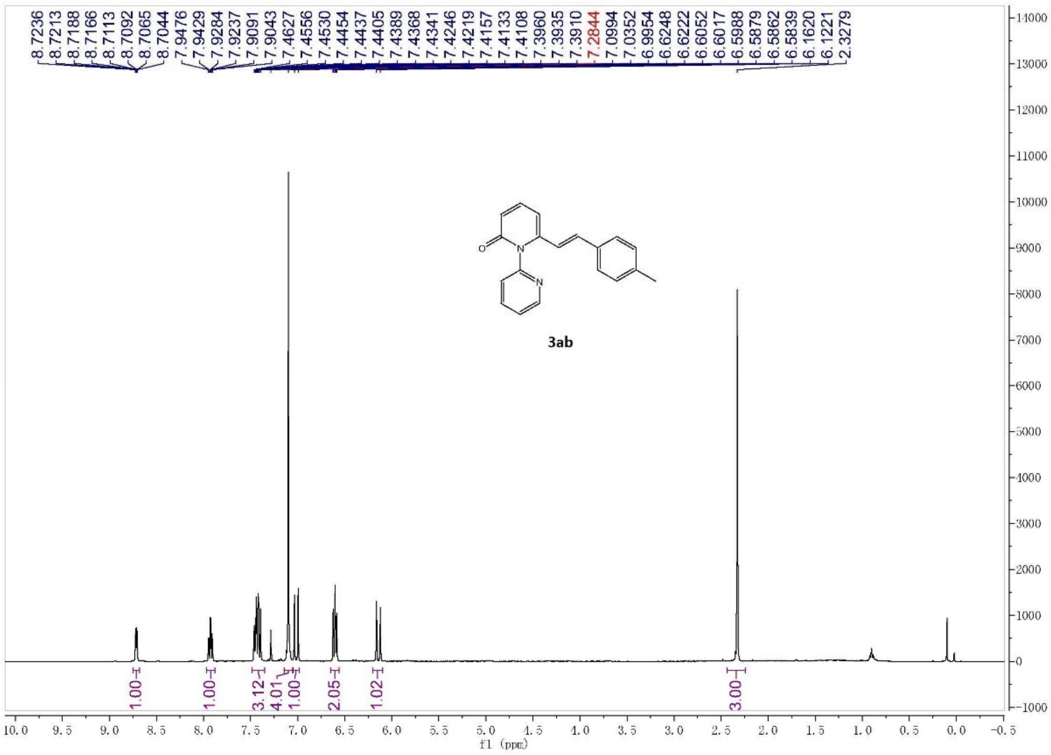

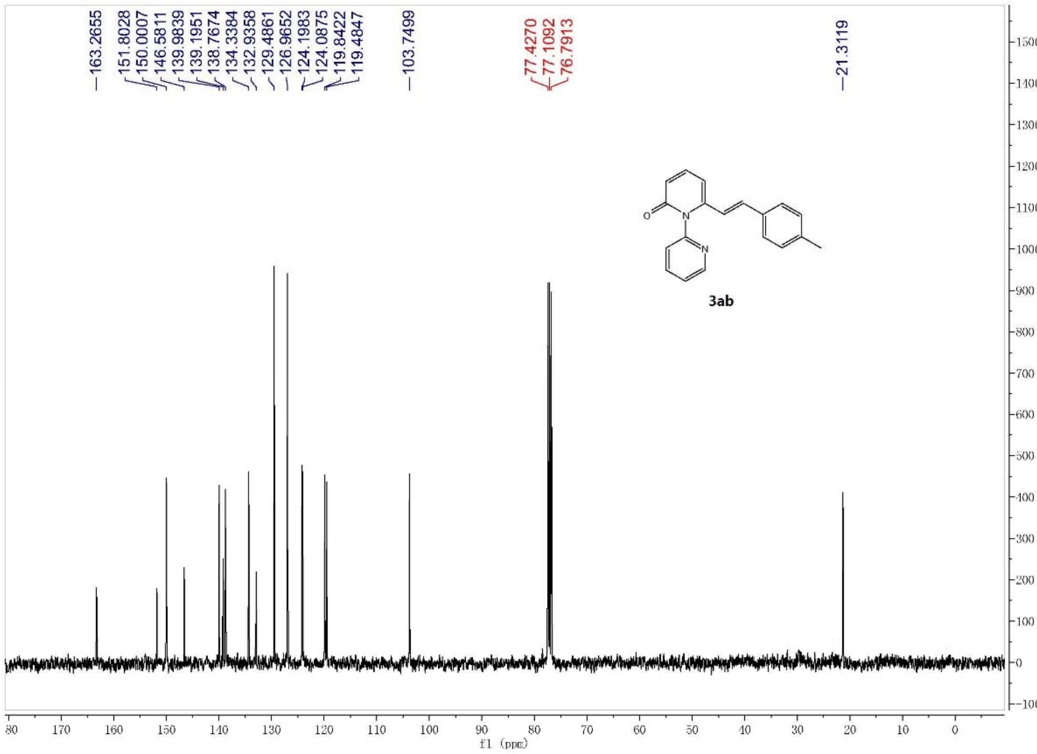

^{1}H and $^{13}C\{^{1}H\}$ NMR spectra of compound 3ac in $CDCl_3$

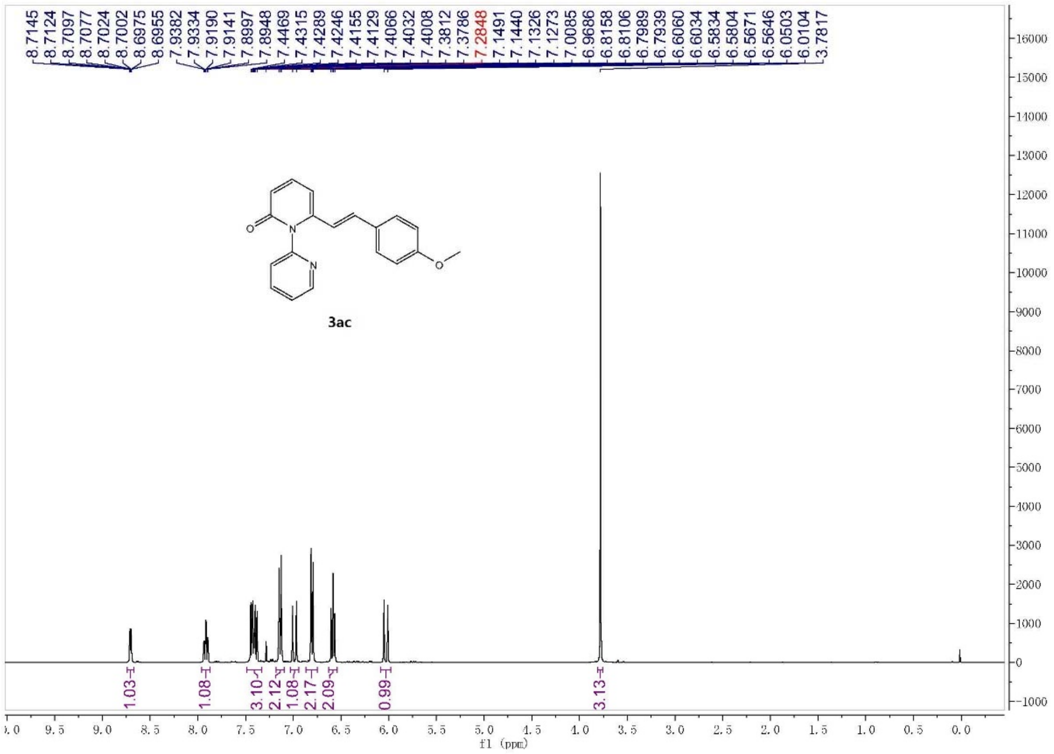

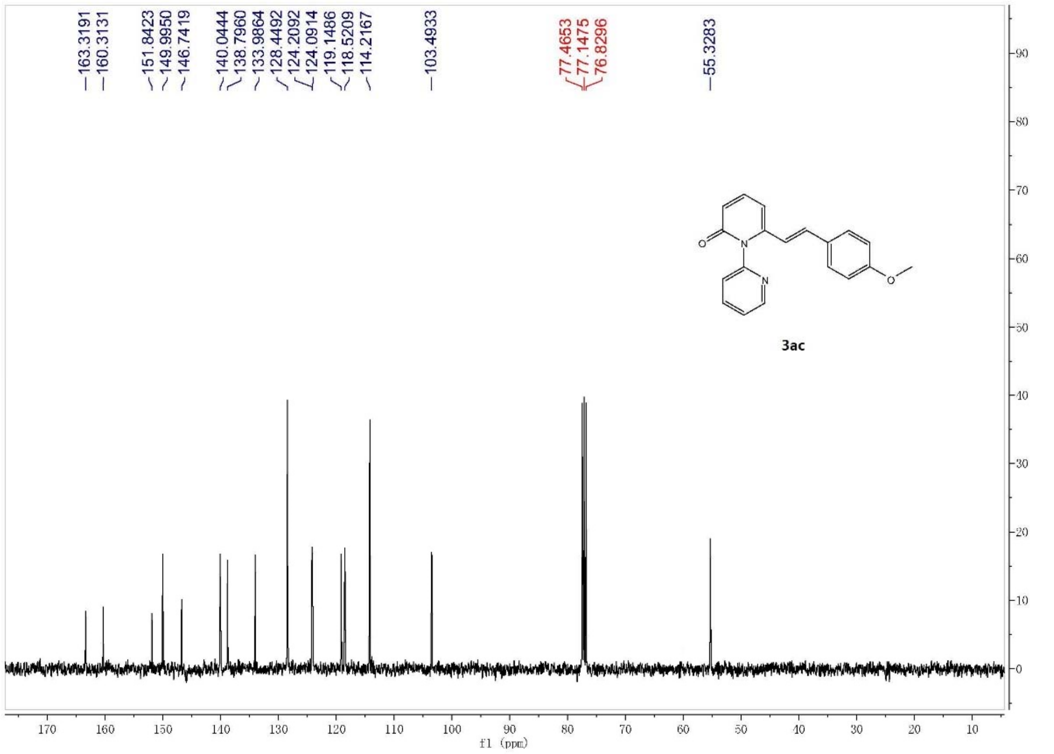

^{1}H and $^{13}C\{^{1}H\}$ NMR spectra of compound 3ad in $CDCl_3$

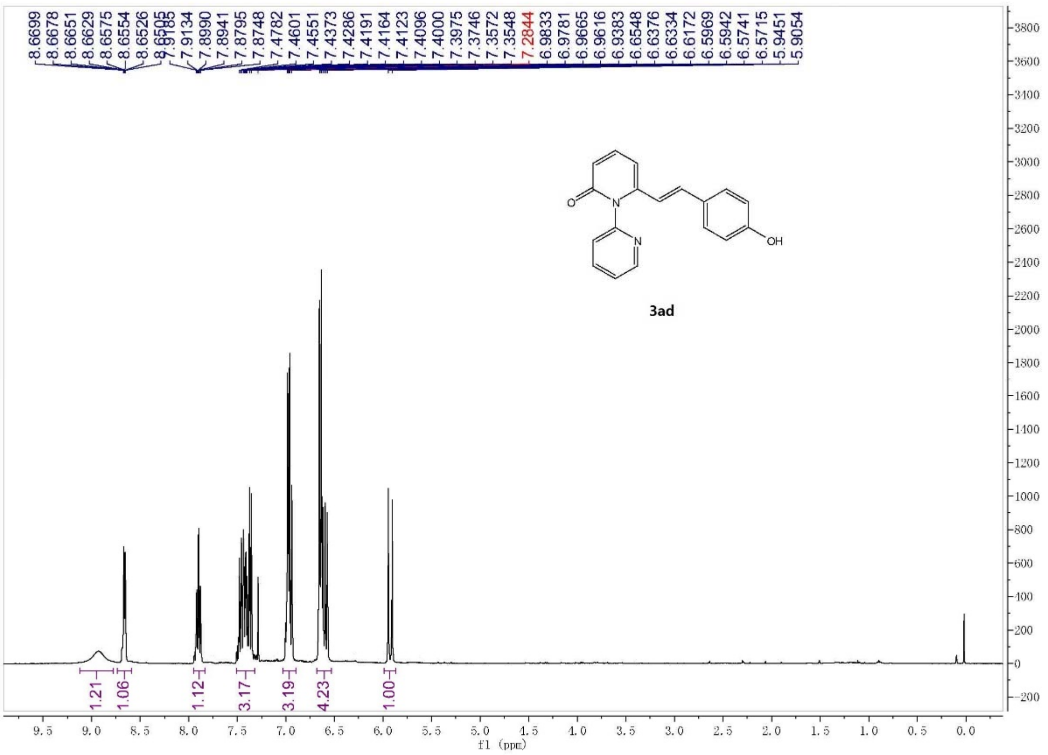

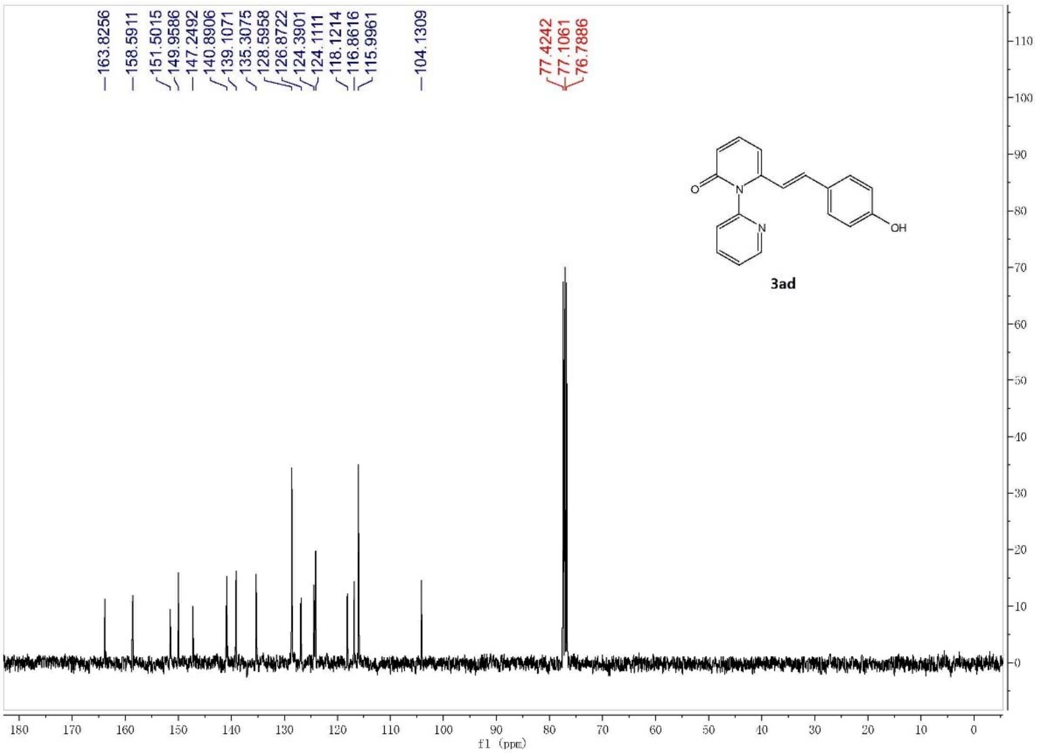

^{1}H and ^{13}C{^{1}H} NMR spectra of compound 3ae in $CDCl_3$

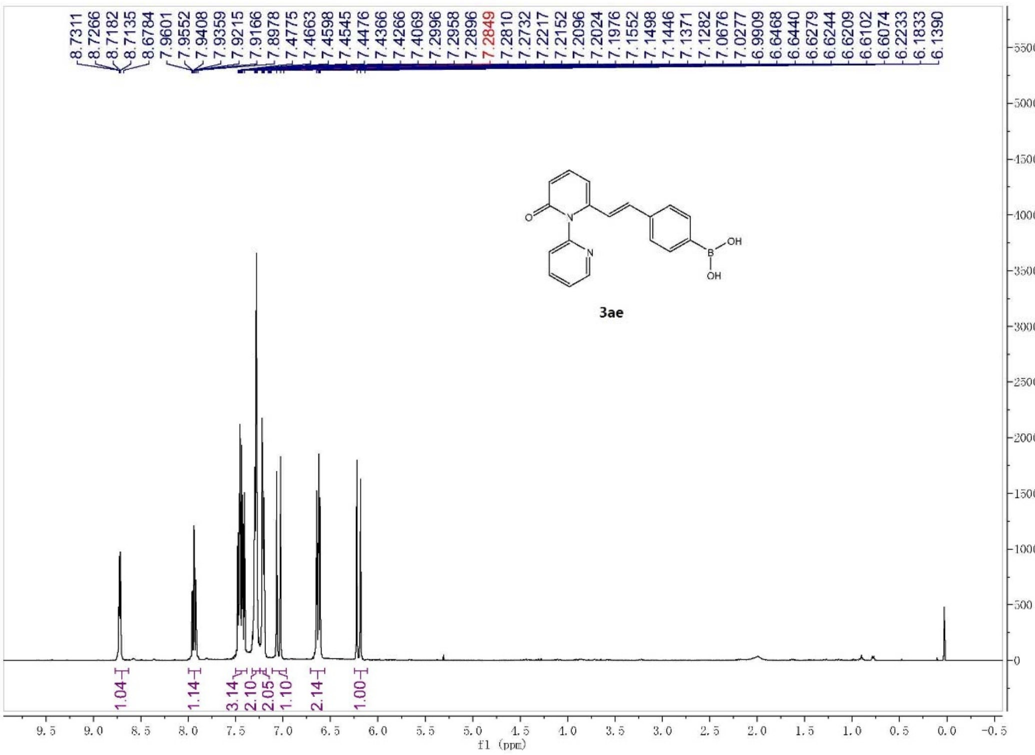

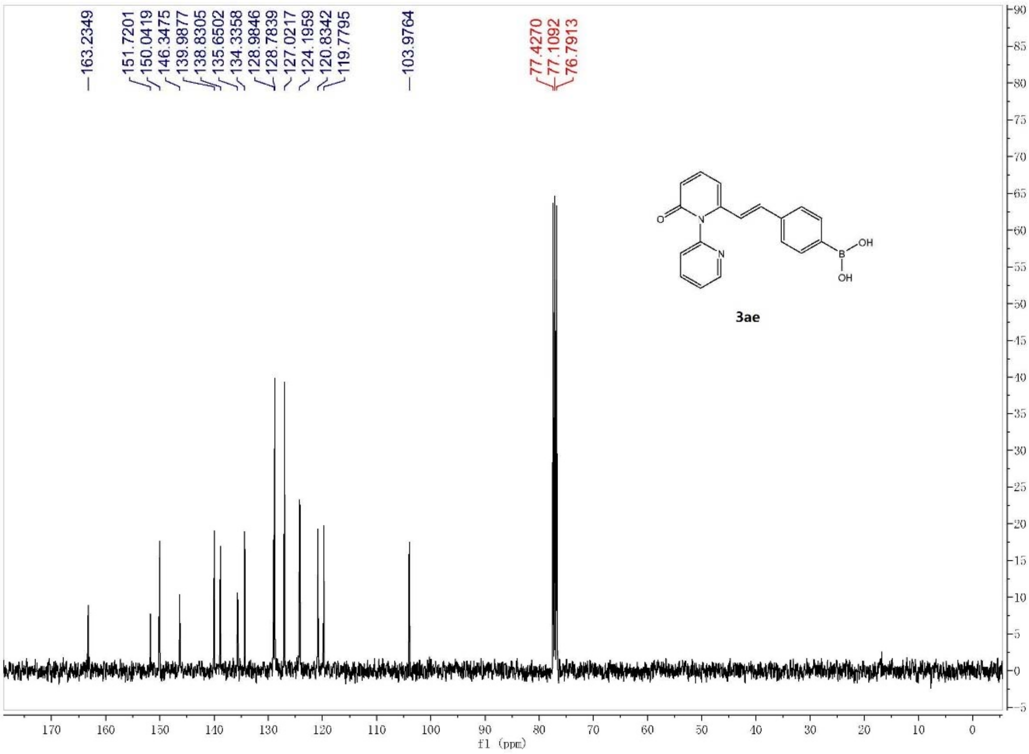

^{1}H and $^{13}C\{^{1}H\}$ NMR spectra of compound 3af in $CDCl_3$

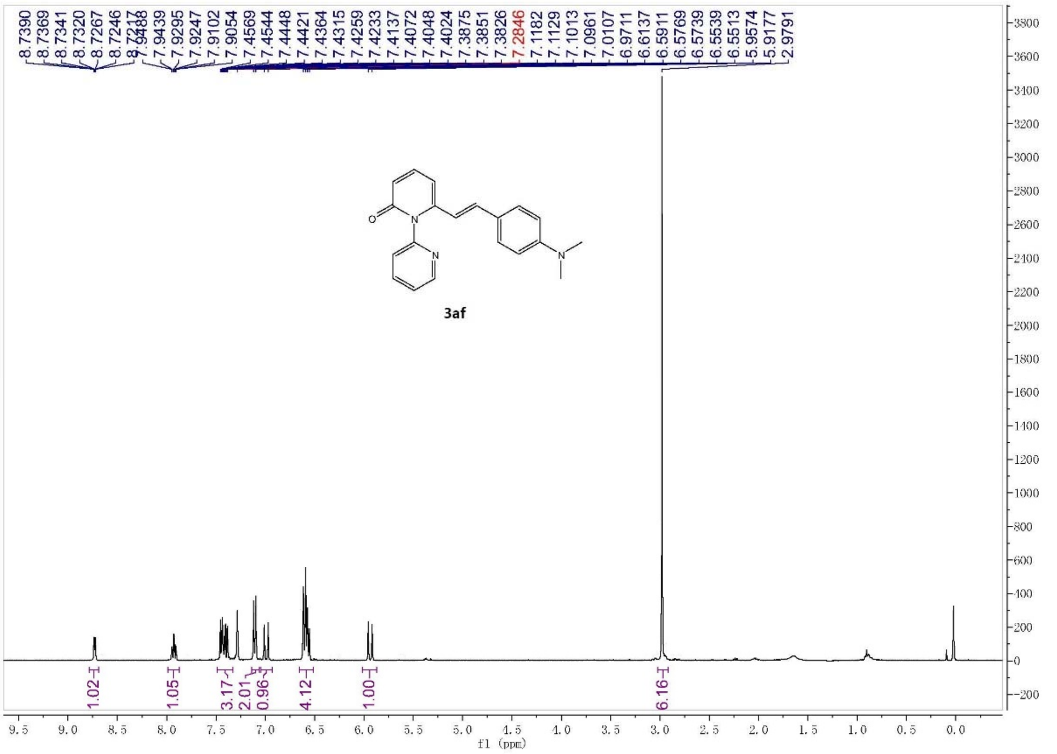

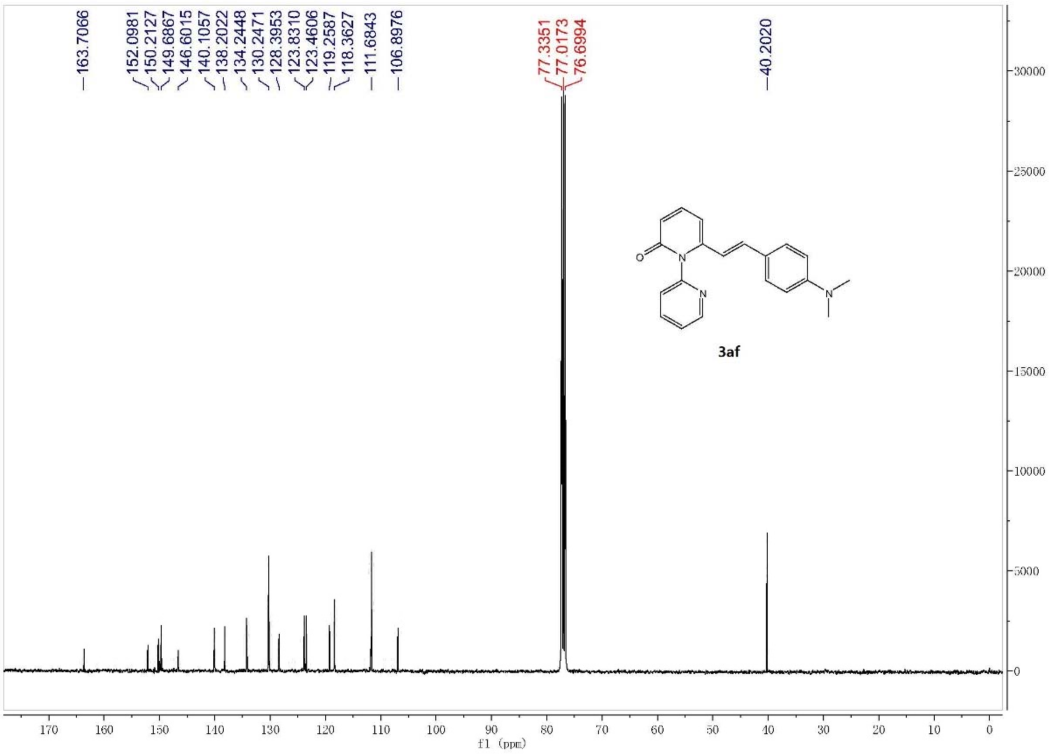

^1H and $^{13}C\{^1H\}$ NMR spectra of compound 3ag in $CDCl_3$

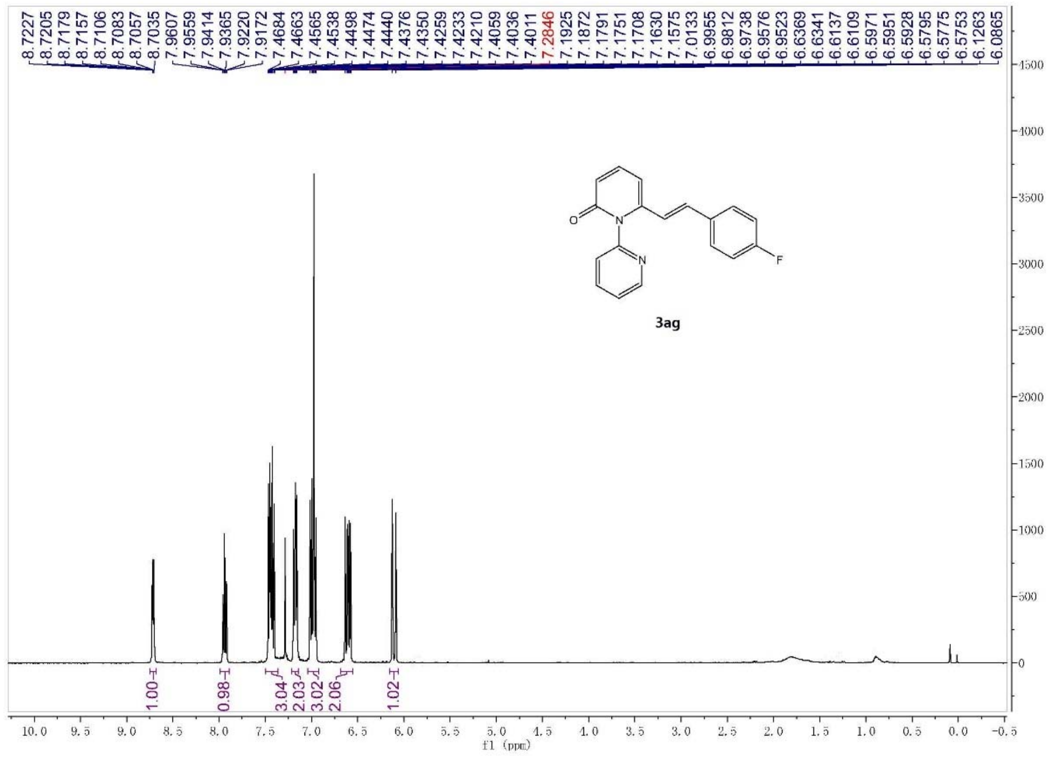

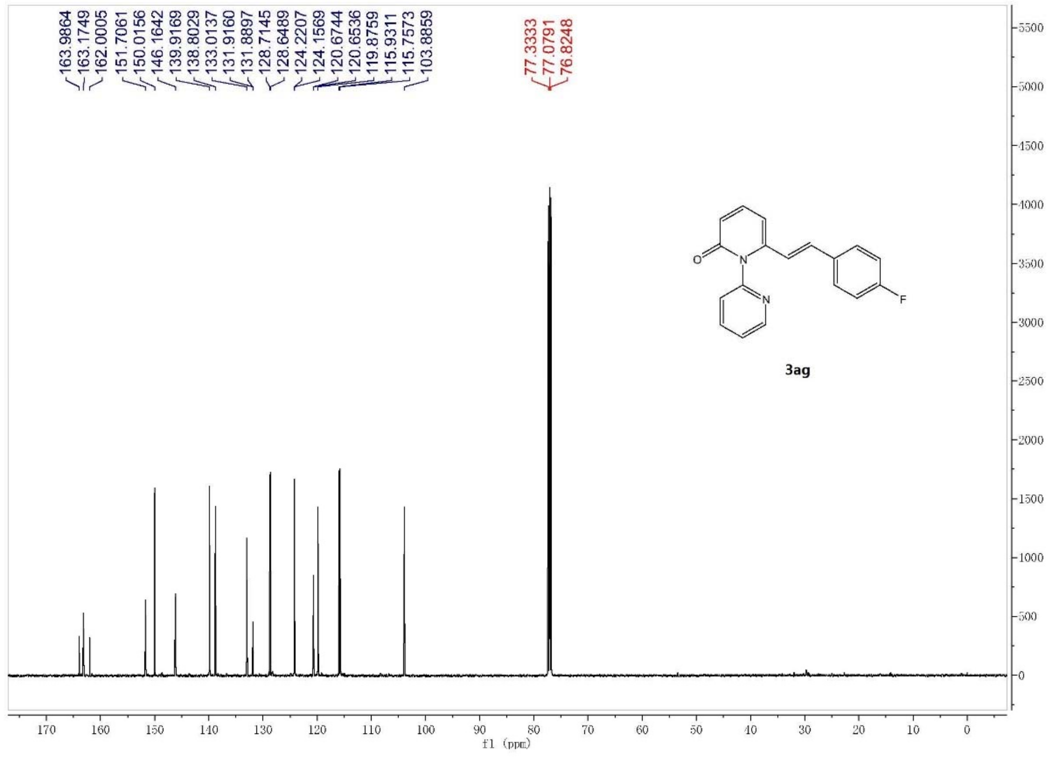

^{1}H and $^{13}C\{^{1}H\}$ NMR spectra of compound 3ah in $CDCl_3$

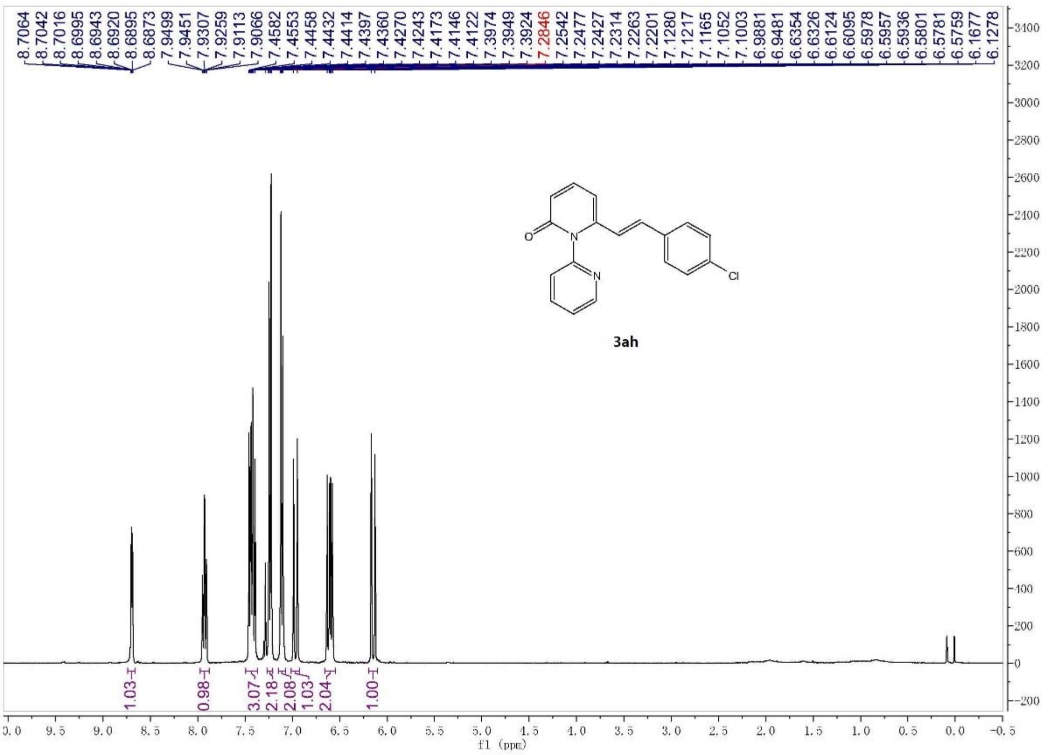

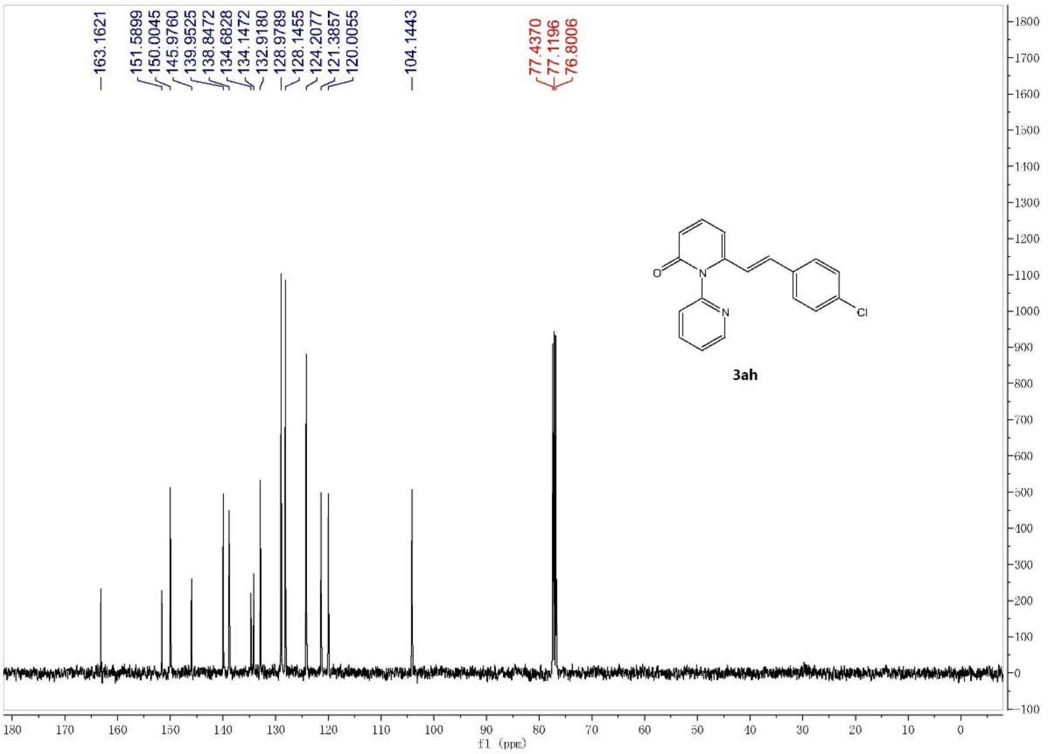

^{1}H and $^{13}C\{^{1}H\}$ NMR spectra of compound 3ai in $CDCl_3$

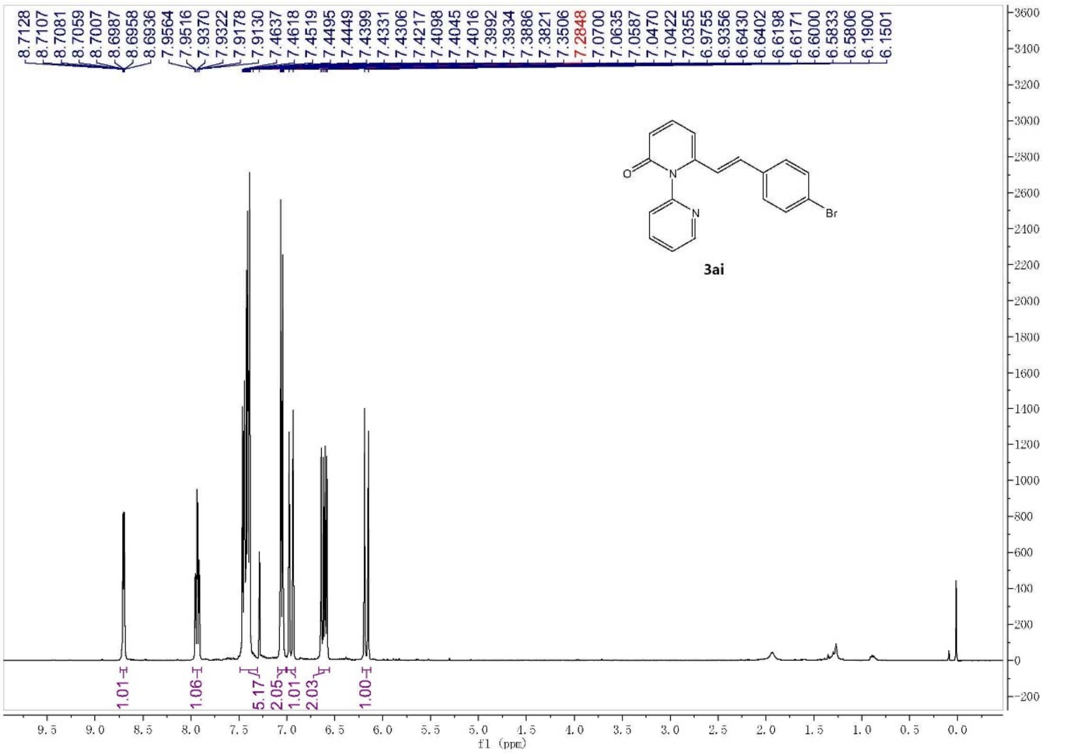

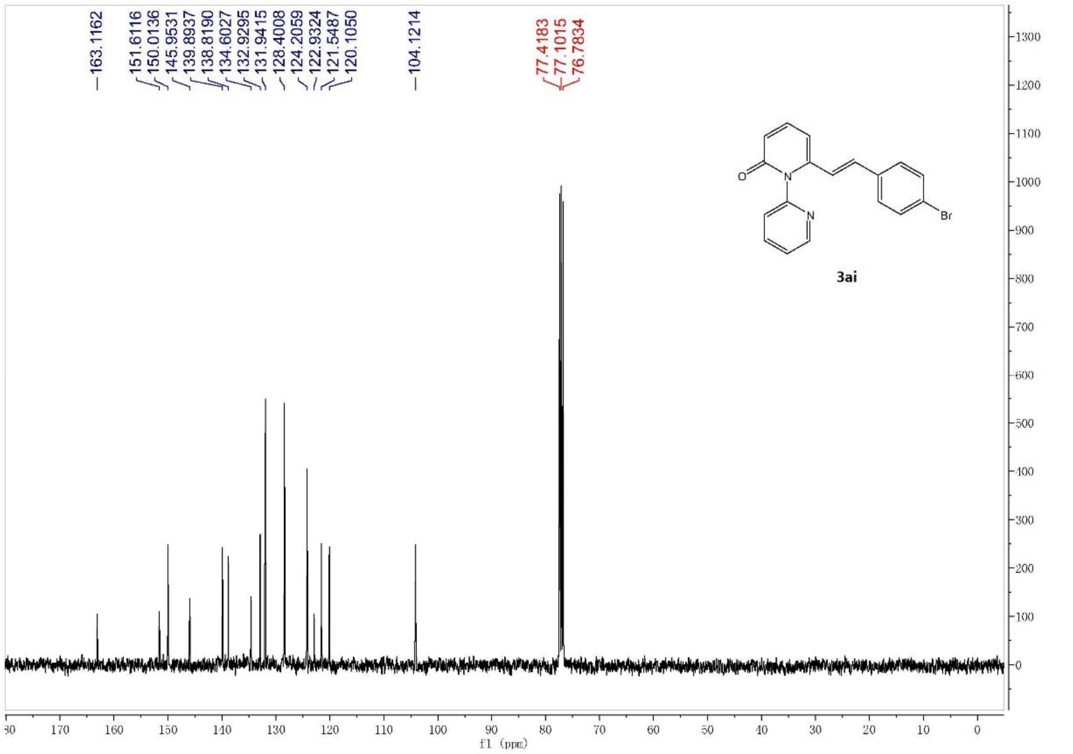

^{1}H and $^{13}C\{^{1}H\}$ NMR spectra of compound 3aj in $CDCl_3$

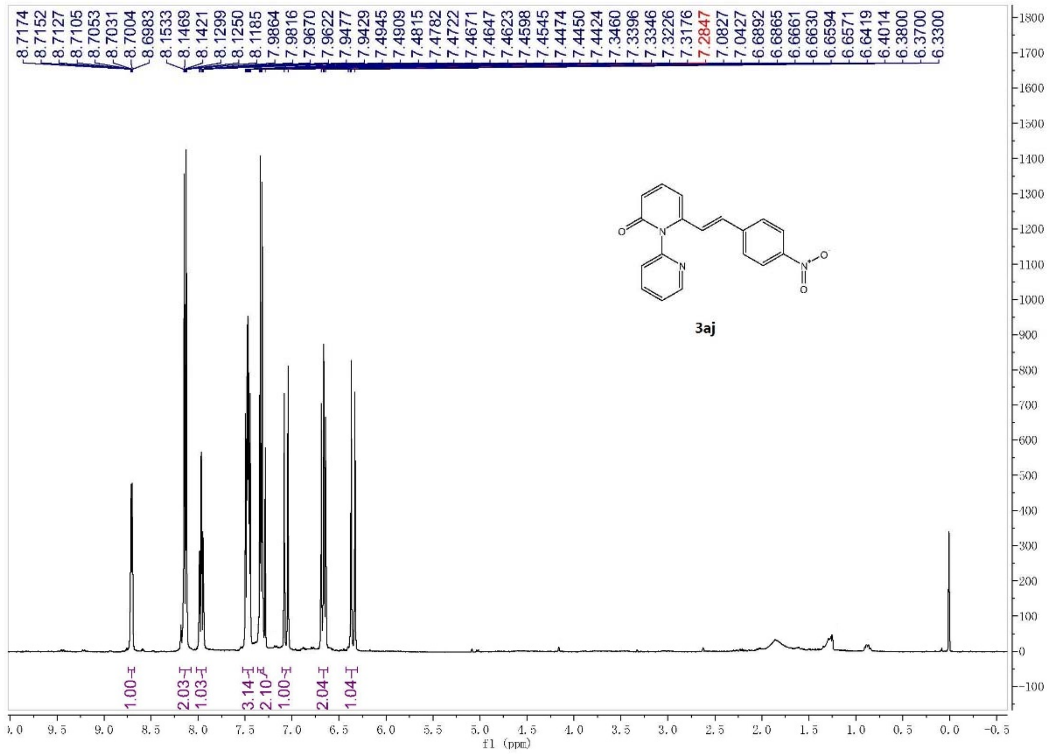

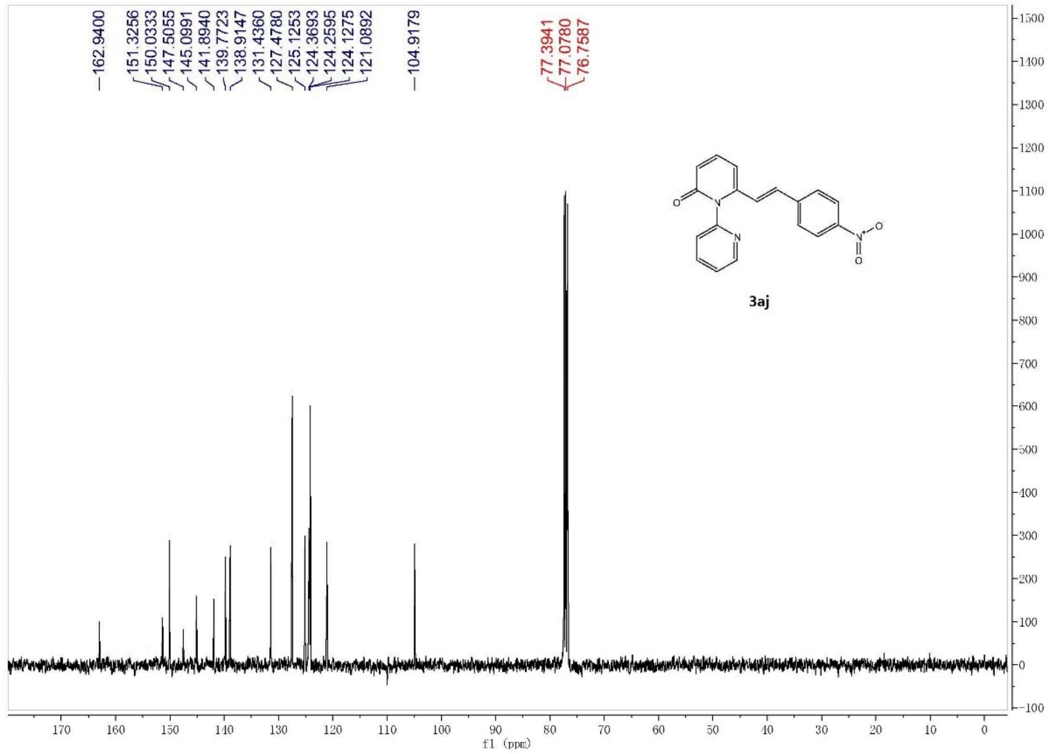

^1H and $^{13}C\{^1H\}$ NMR spectra of compound 3ak in $CDCl_3$

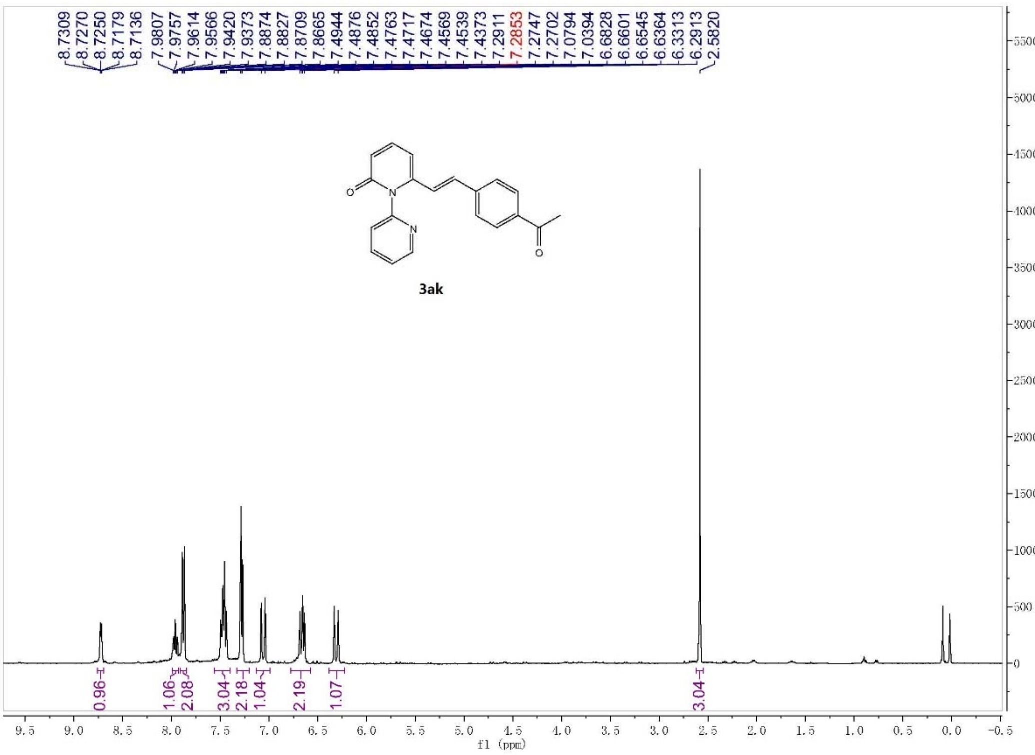

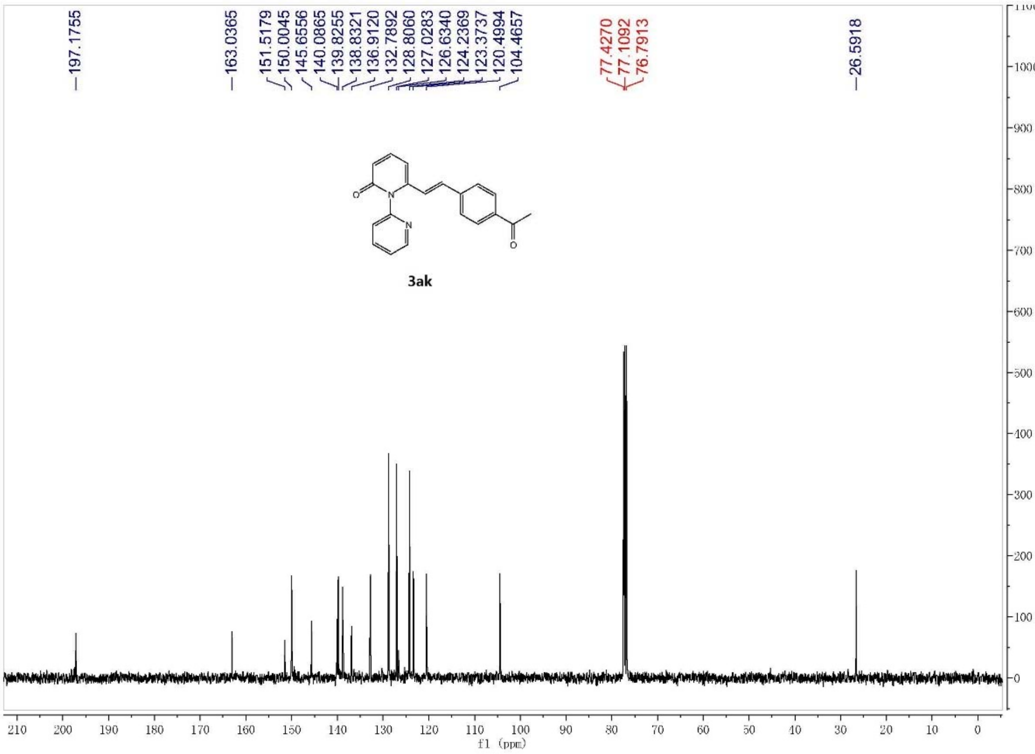

^{1}H and $^{13}C\{^{1}H\}$ NMR spectra of compound 3al in $CDCl_3$

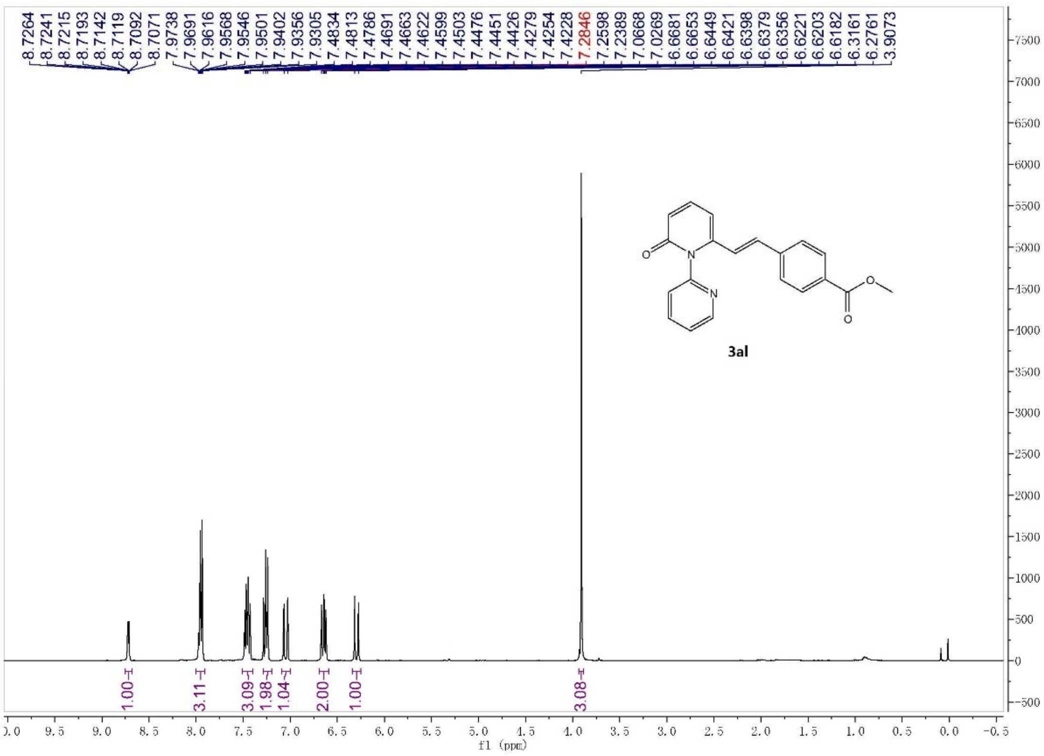

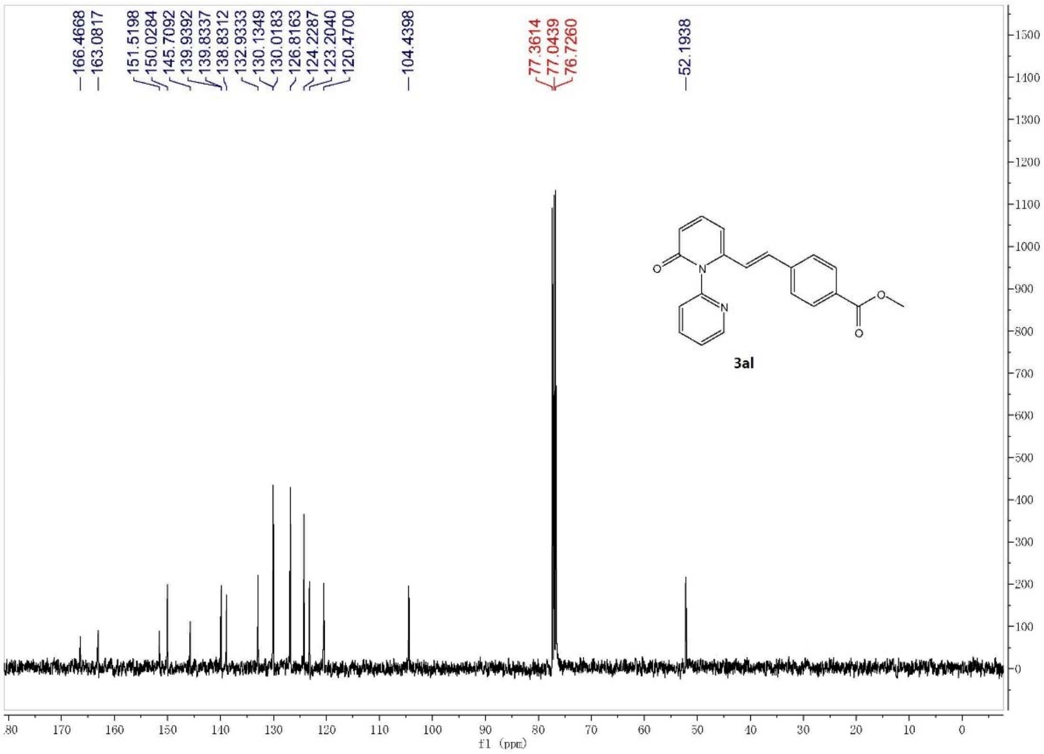

^{1}H and $^{13}C\{^{1}H\}$ NMR spectra of compound 3am in $CDCl_3$

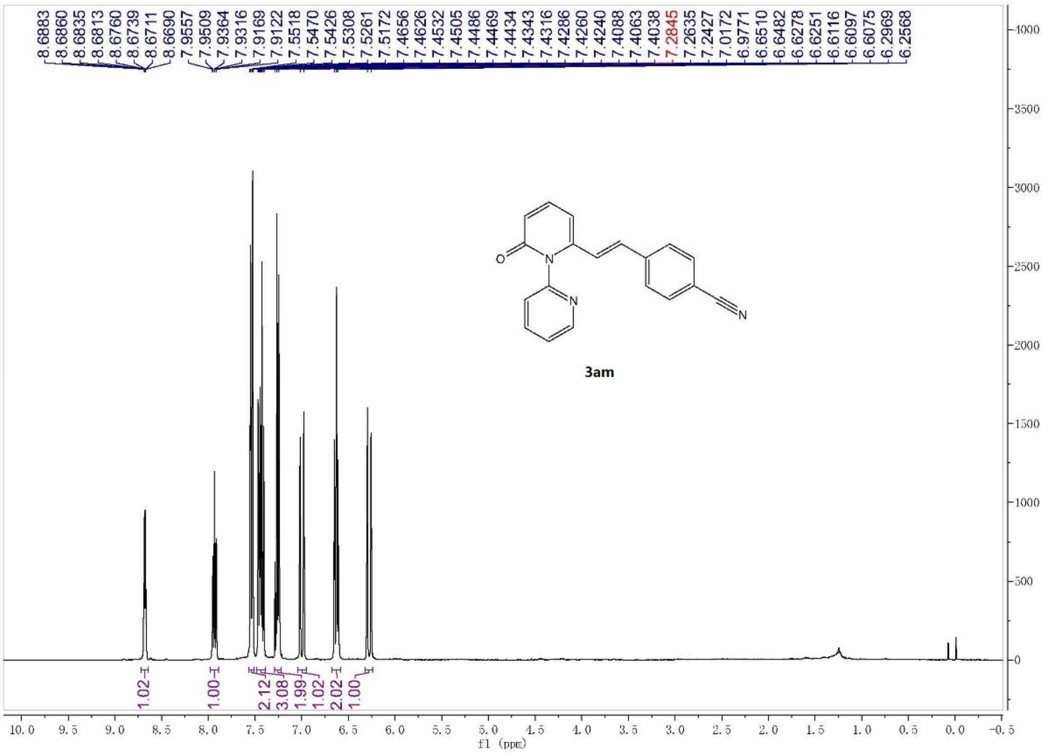

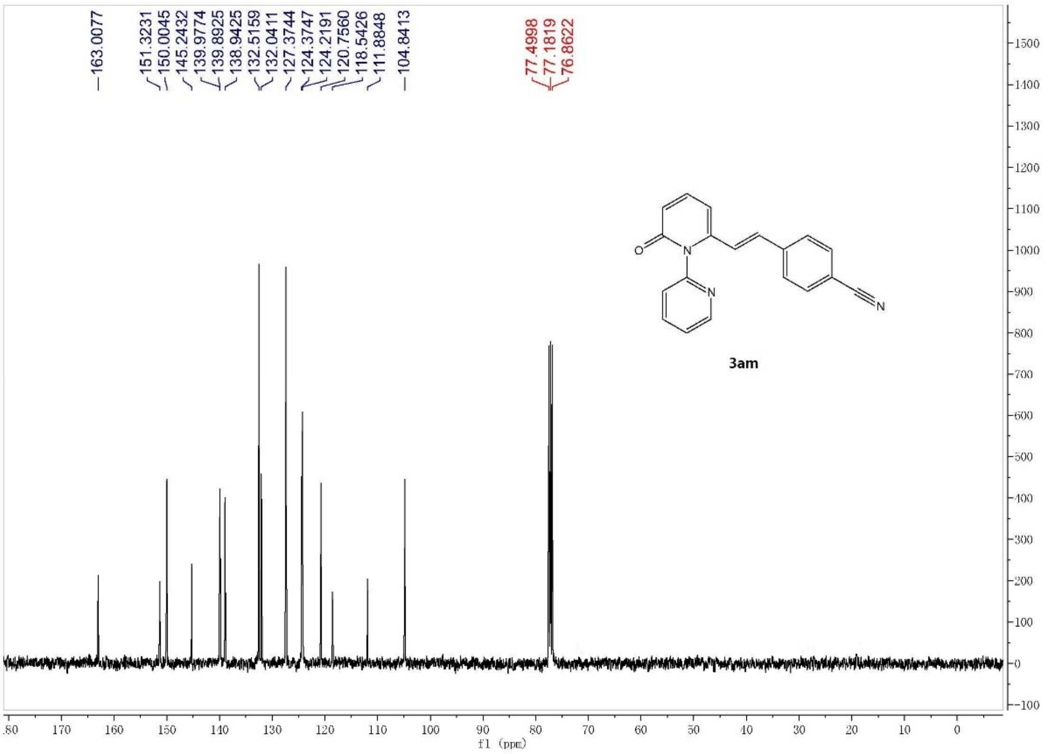

^{1}H and $^{13}C\{^{1}H\}$ NMR spectra of compound 3an in $CDCl_3$

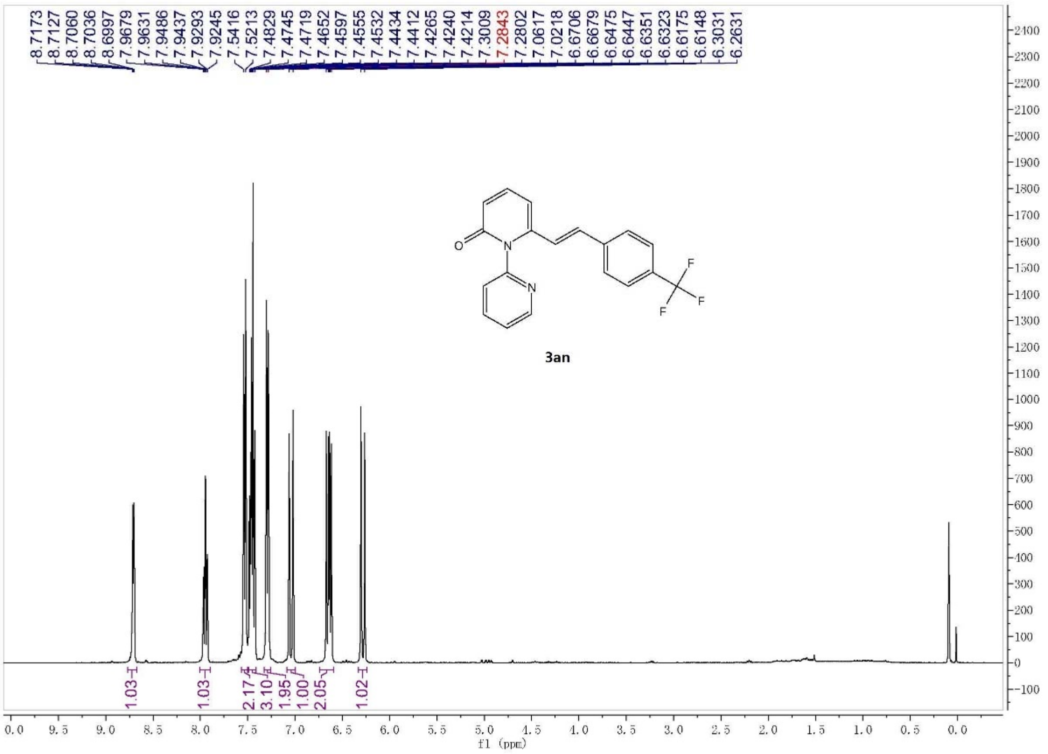

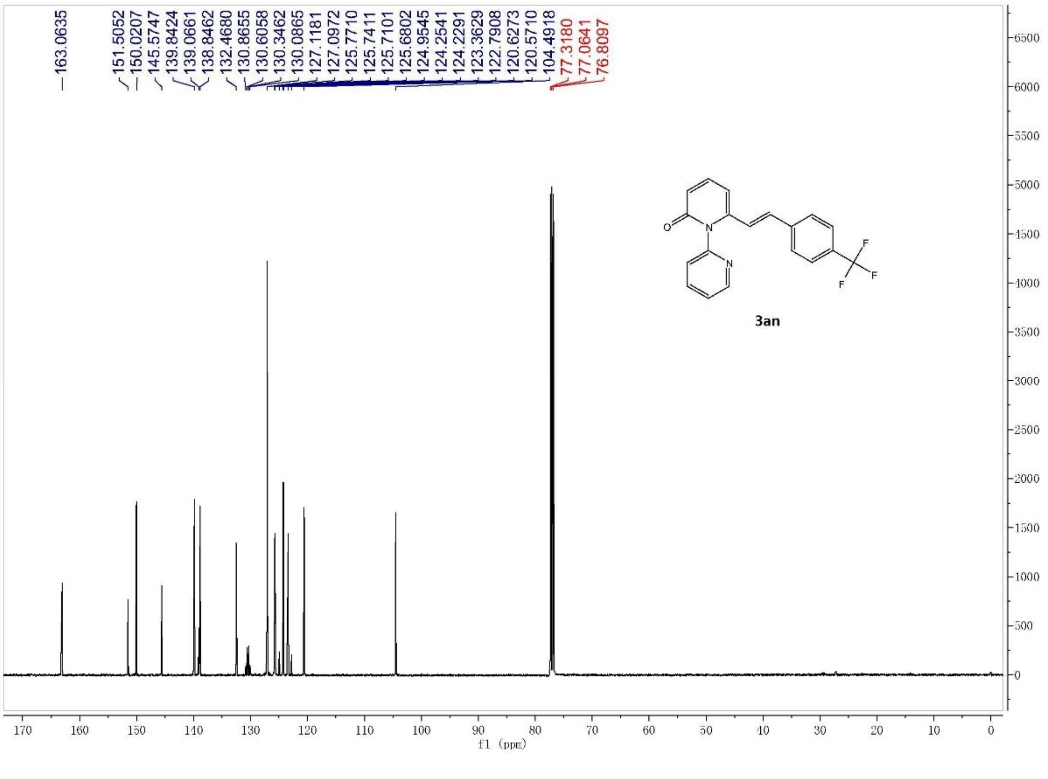

^{1}H and $^{13}C\{^{1}H\}$ NMR spectra of compound 3ao in $CDCl_3$

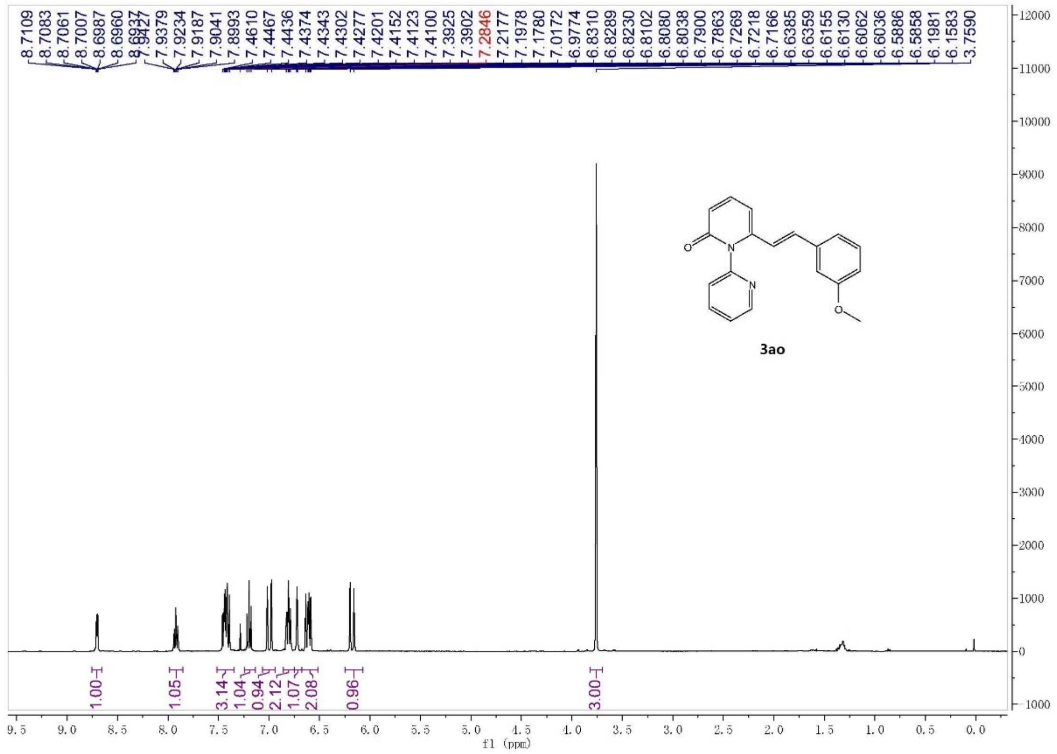

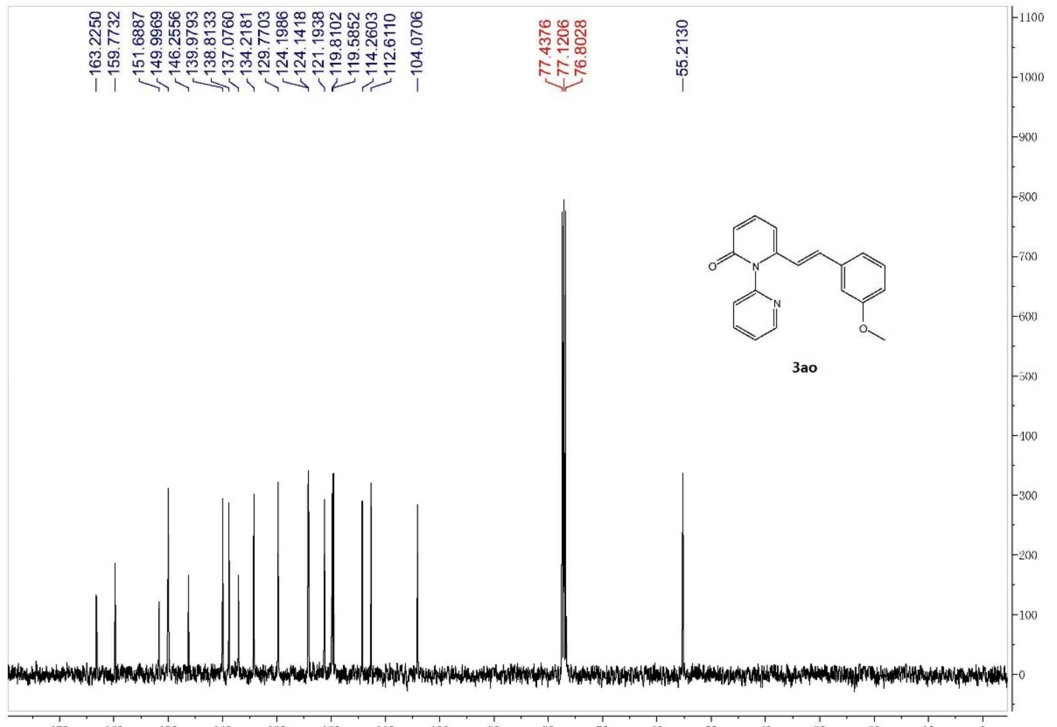

^1H and $^{13}C\{^1H\}$ NMR spectra of compound 3ap in $CDCl_3$

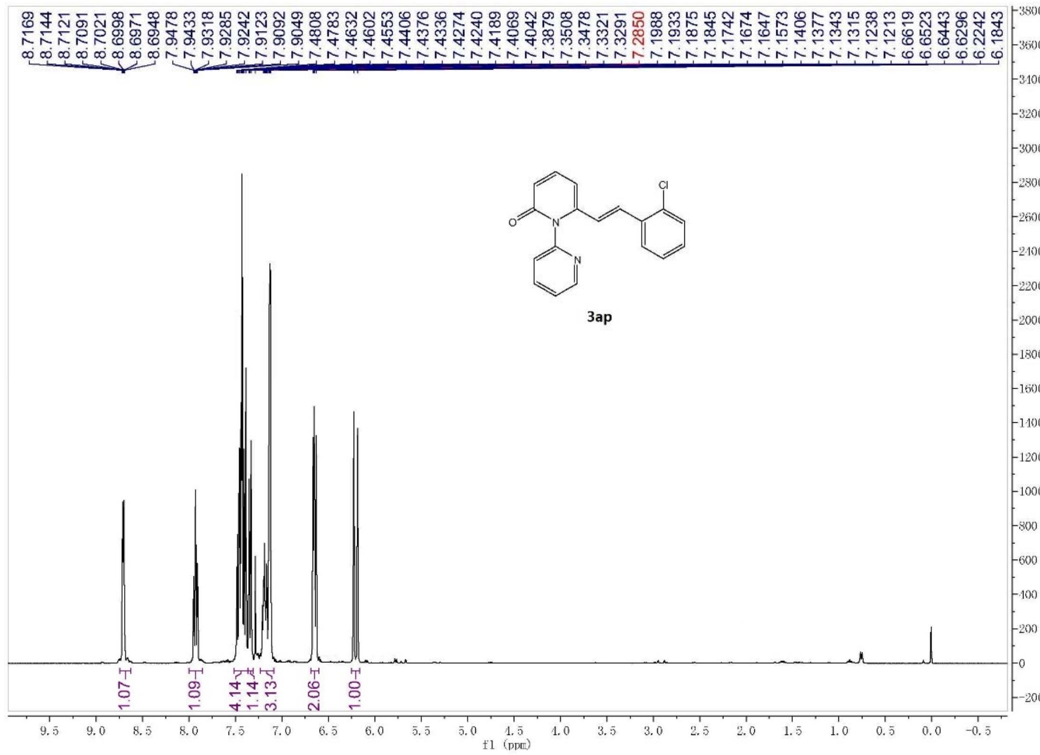

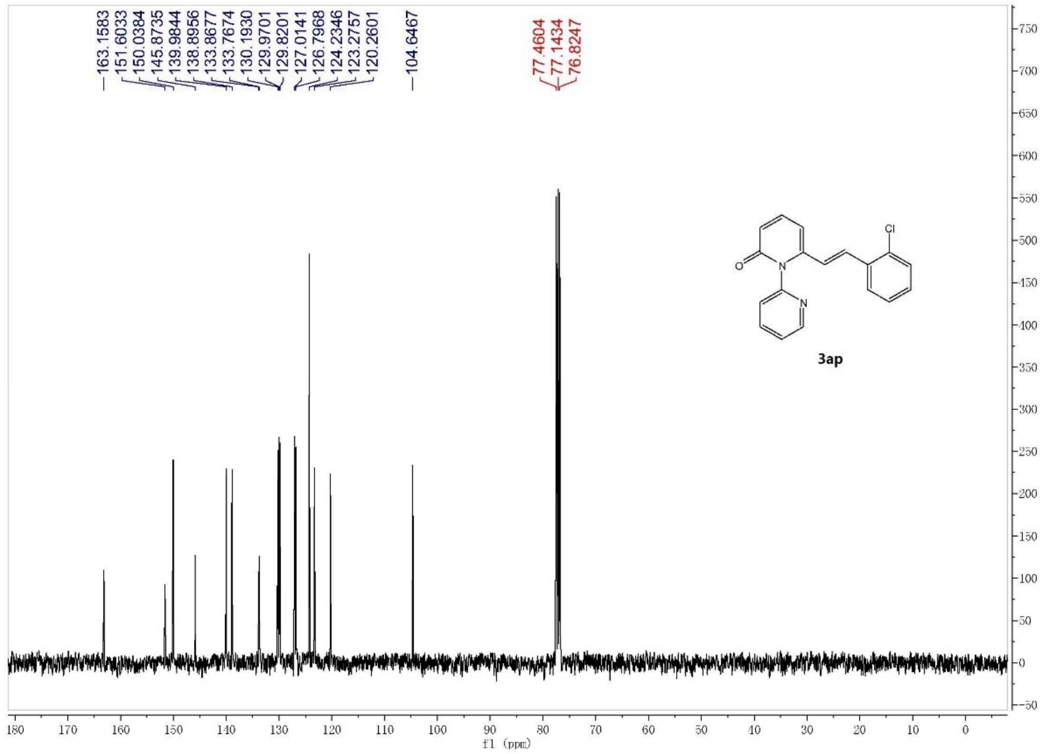

^{1}H and $^{13}C\{^{1}H\}$ NMR spectra of compound 3aq in $CDCl_3$

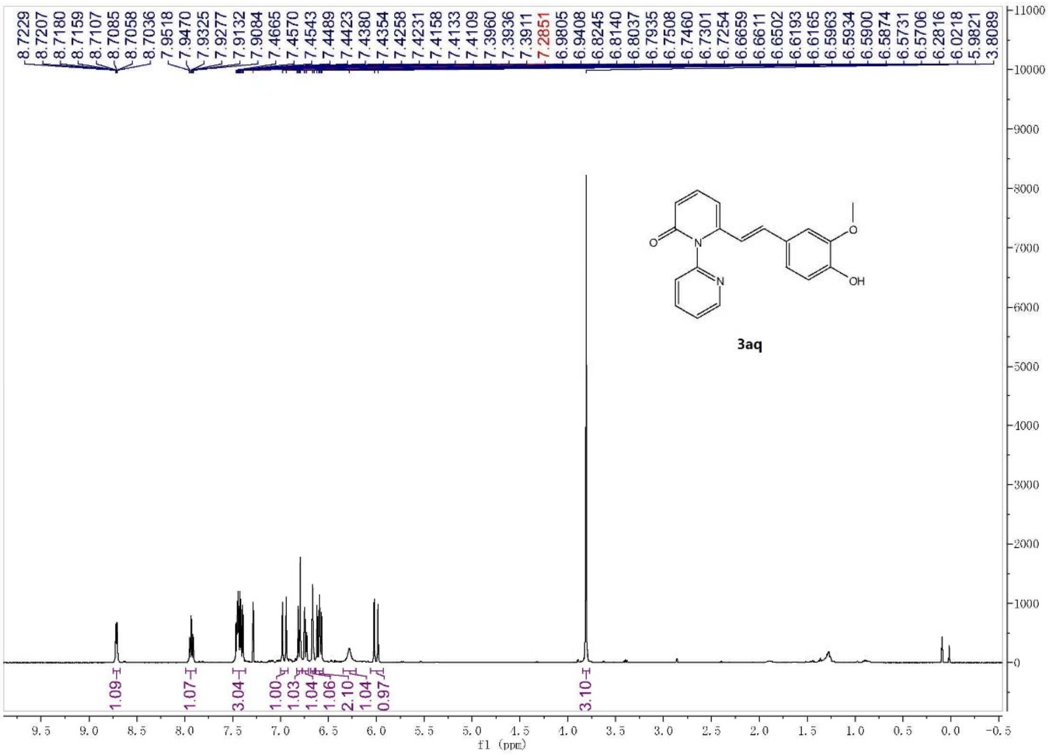

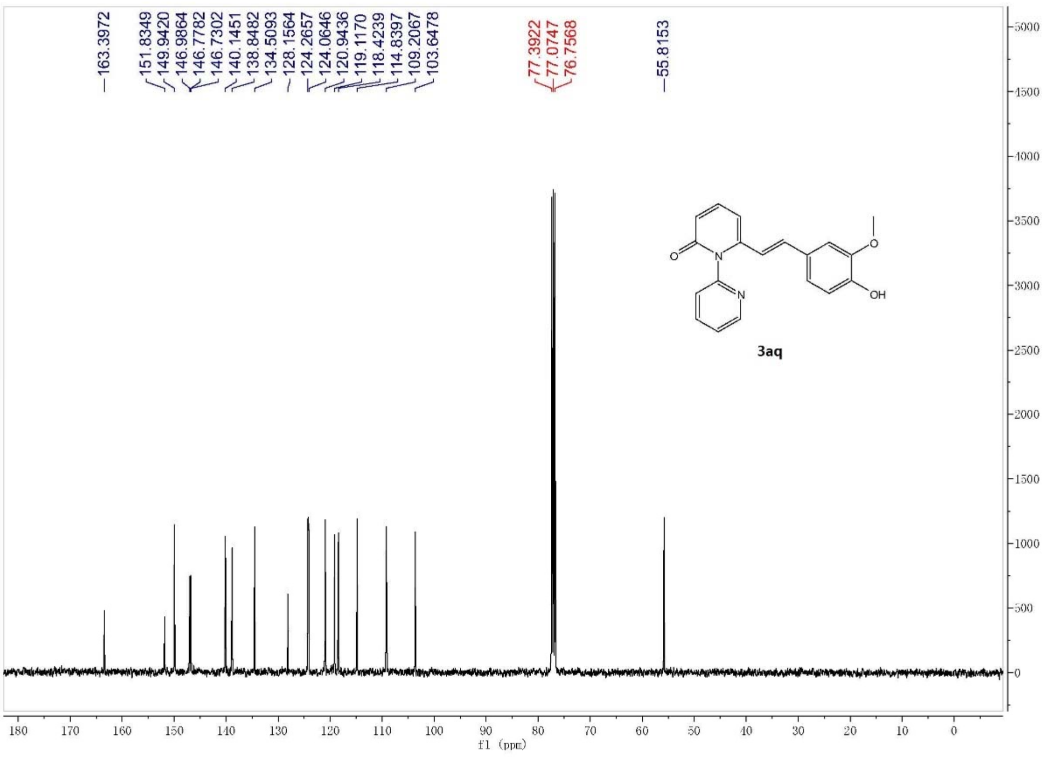

^1H and $^{13}C\{^1H\}$ NMR spectra of compound 3ar in $CDCl_3$

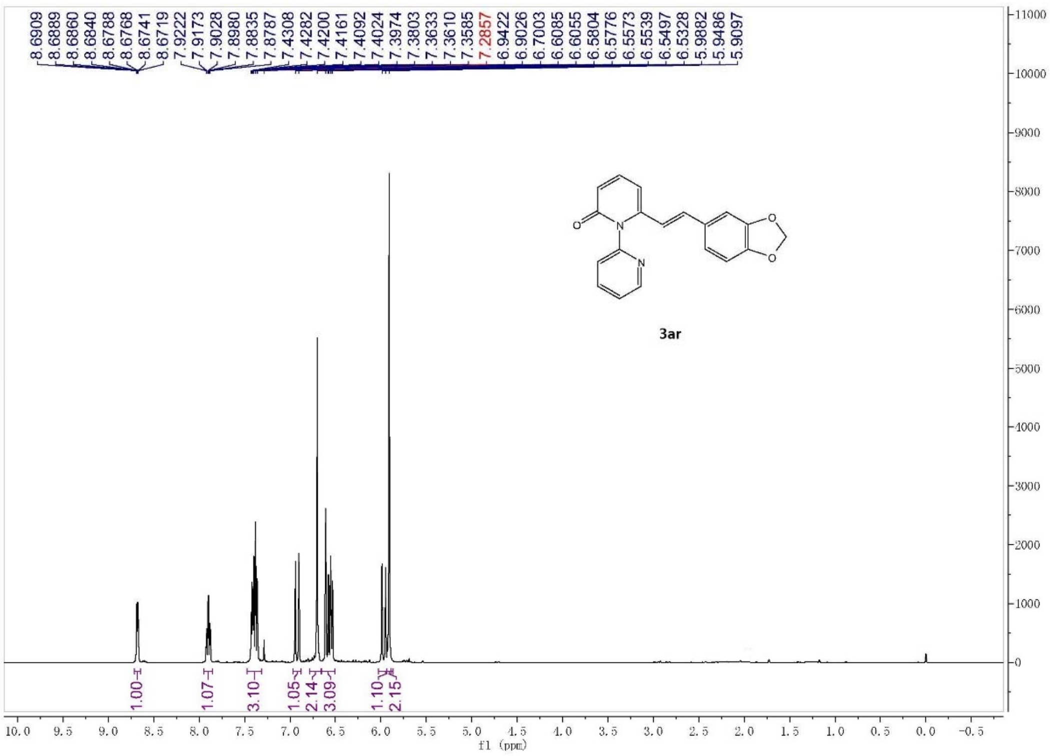

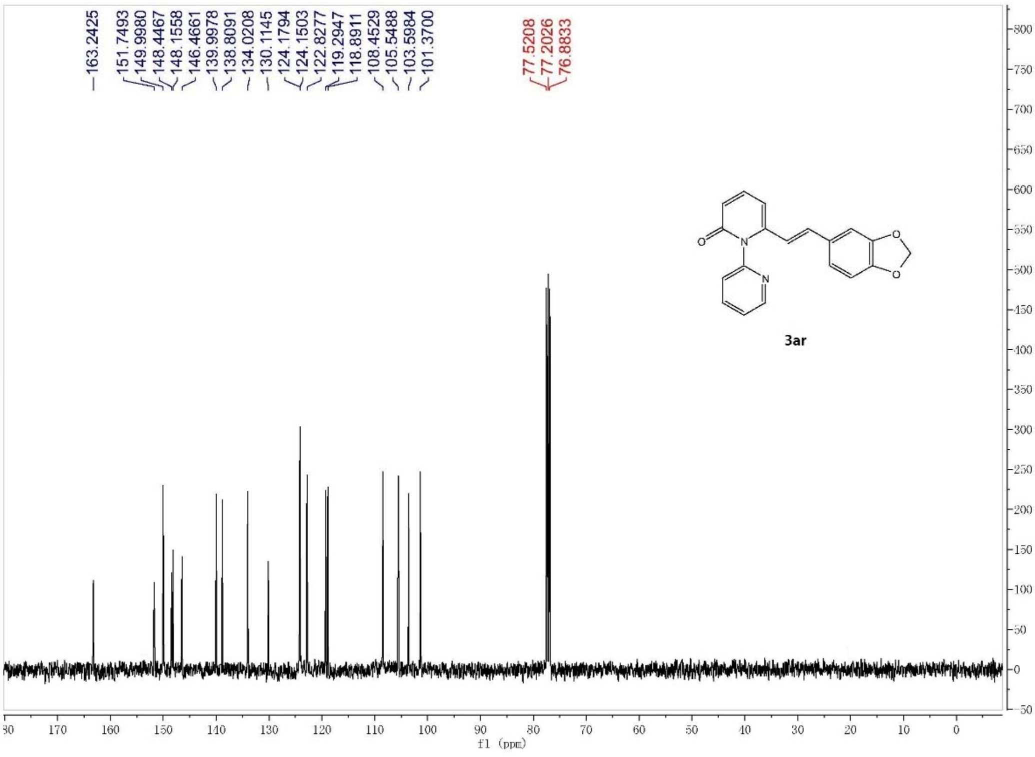

^{1}H and $^{13}C\{^{1}H\}$ NMR spectra of compound 3as in $CDCl_3$

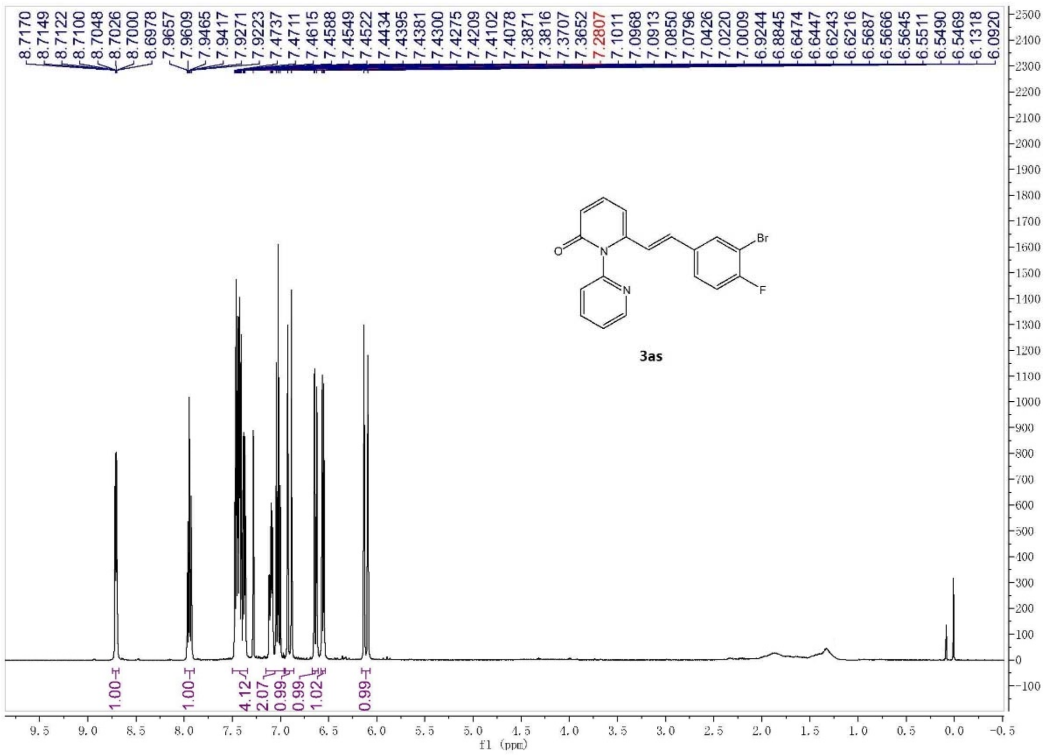

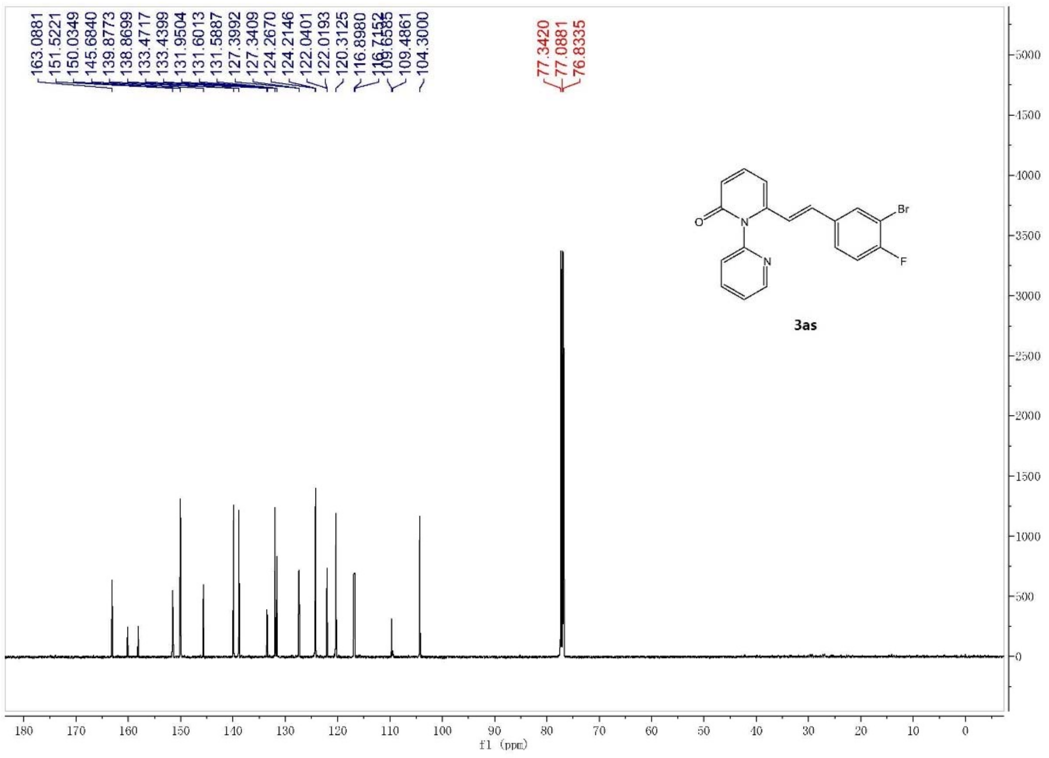

^{1}H and ^{13}C{^{1}H} NMR spectra of compound 3at in $CDCl_3$

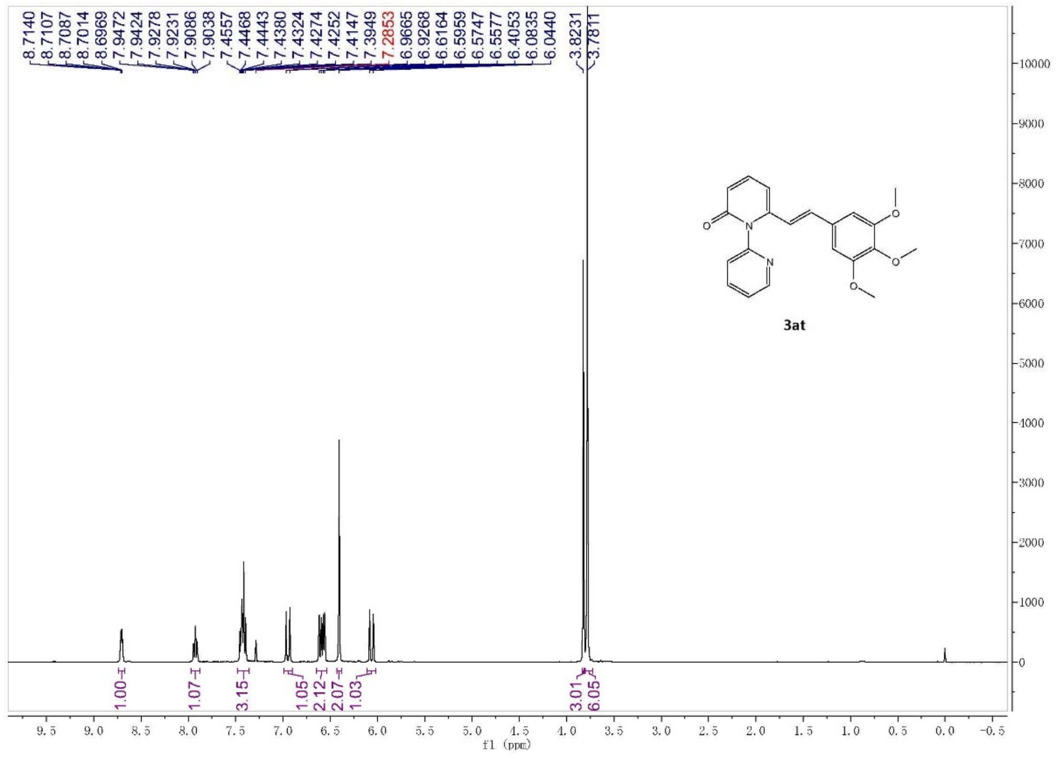

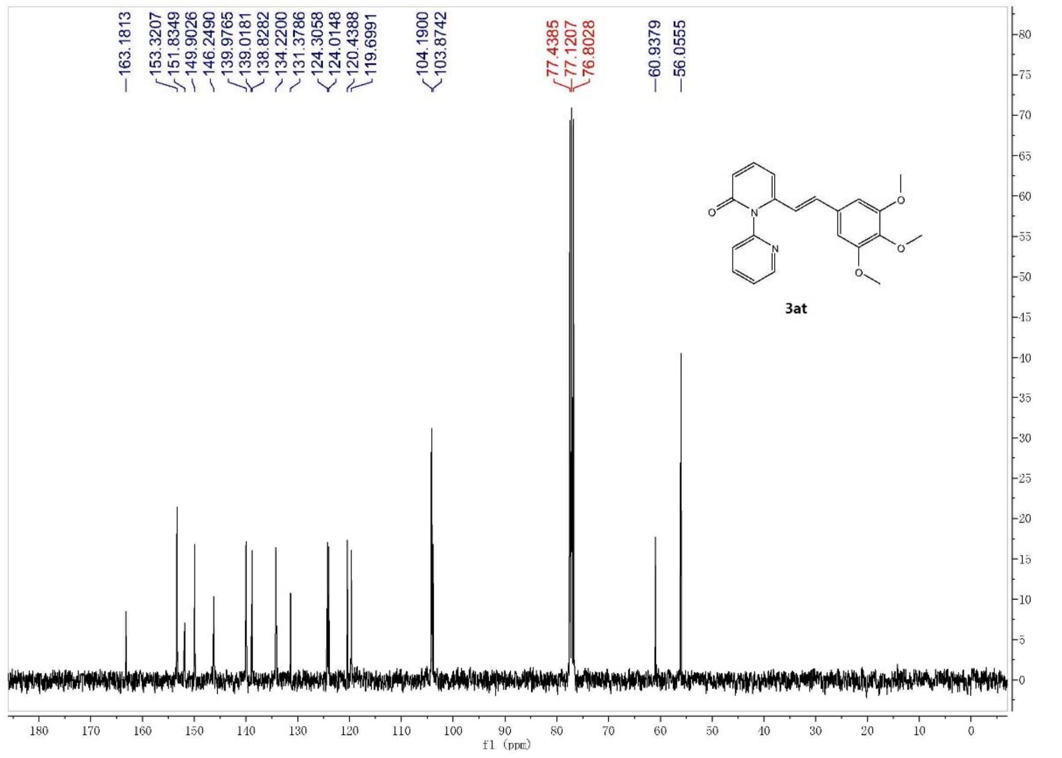

^{1}H and $^{13}C\{^{1}H\}$ NMR spectra of compound 3au in $CDCl_3$

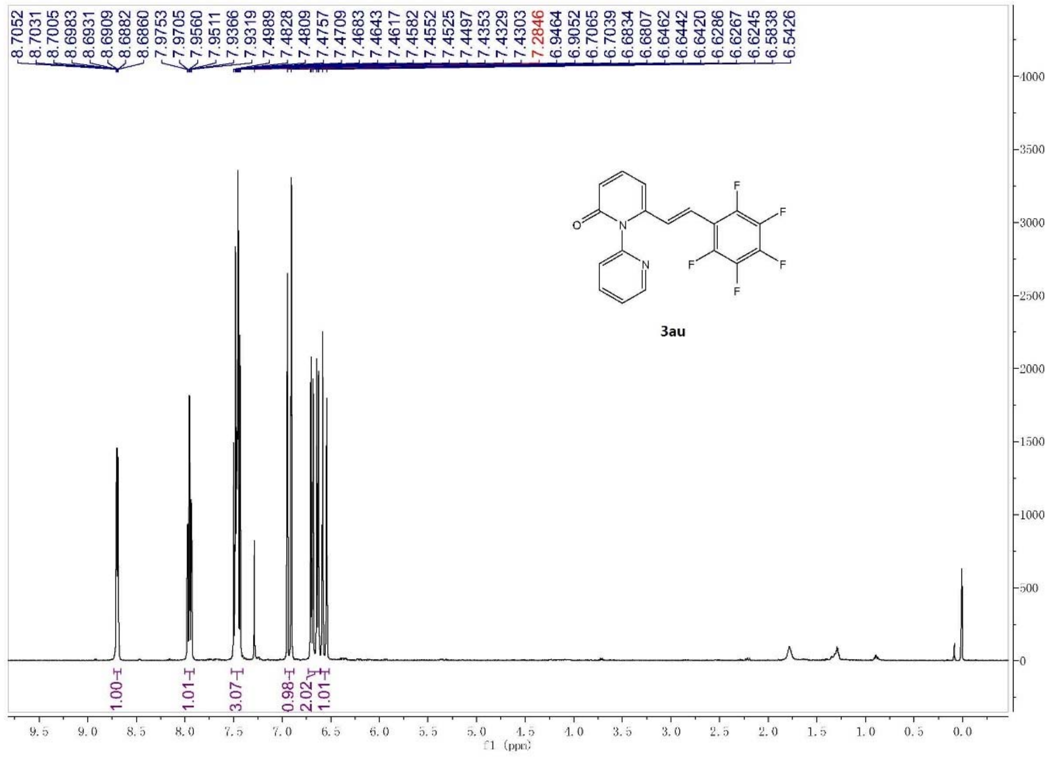

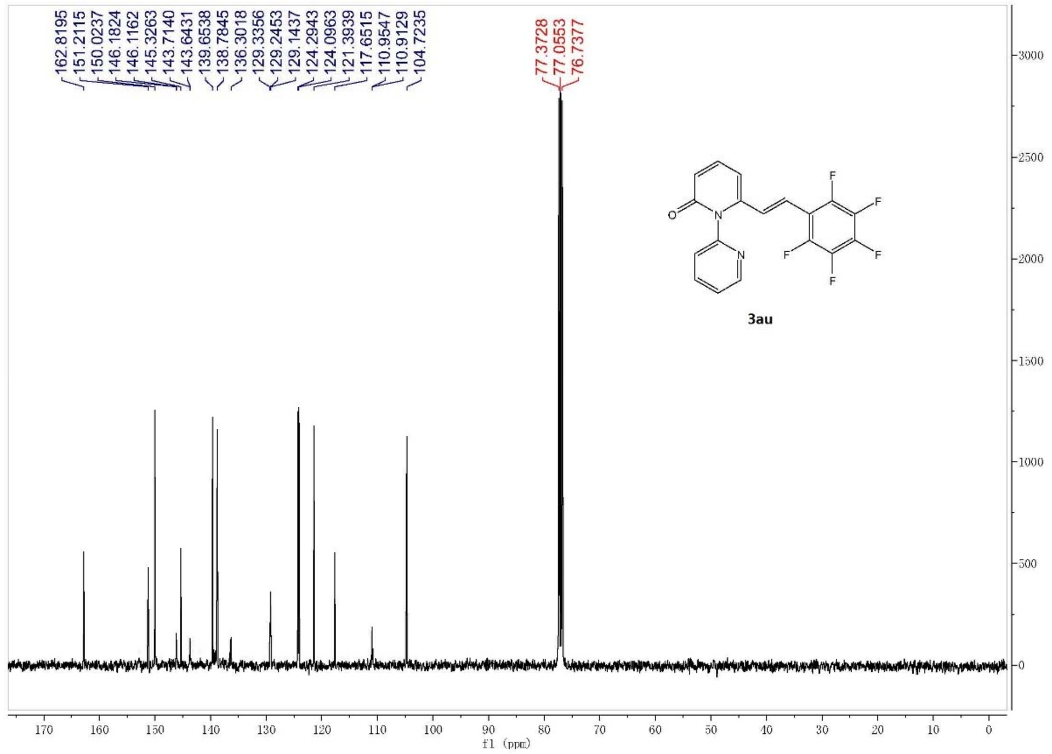

^{19}F NMR spectra of compound 3au in $CDCl_3$

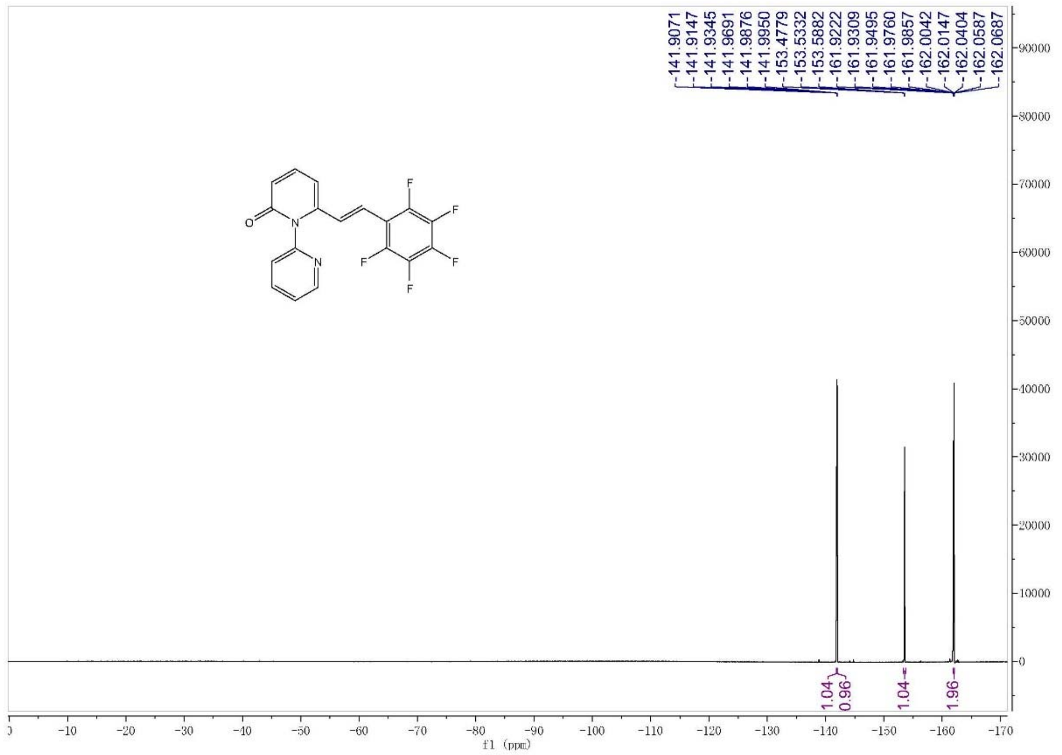

^{1}H and $^{13}C\{^{1}H\}$ NMR spectra of compound 3av in $CDCl_3$

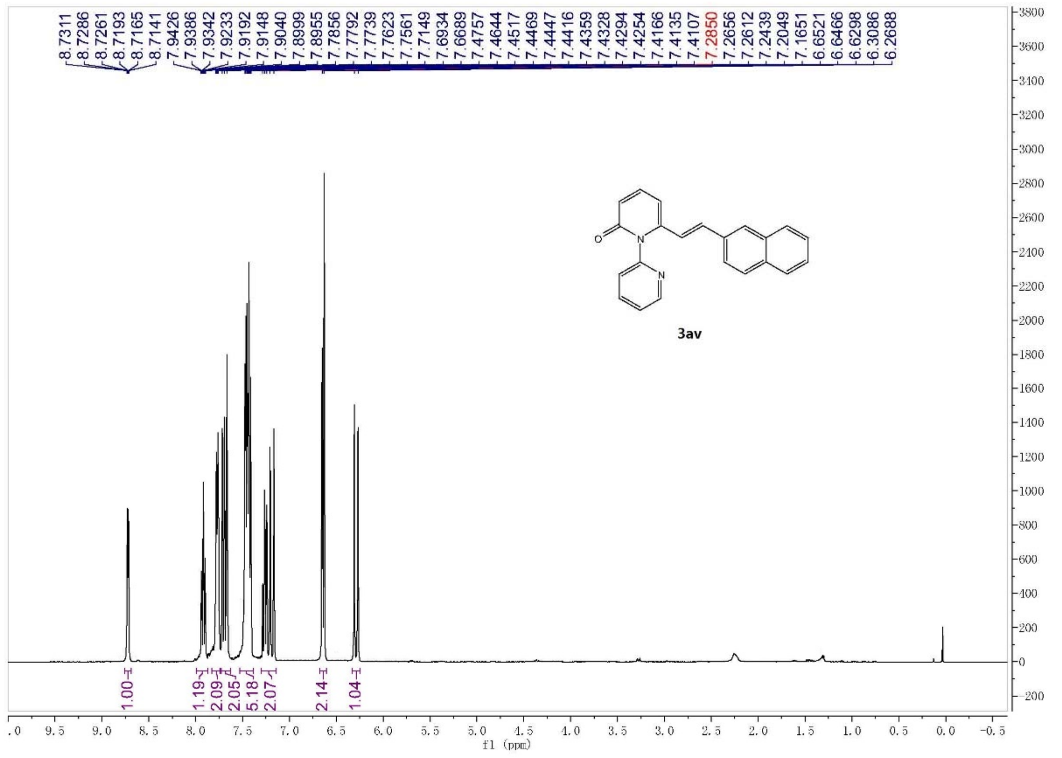

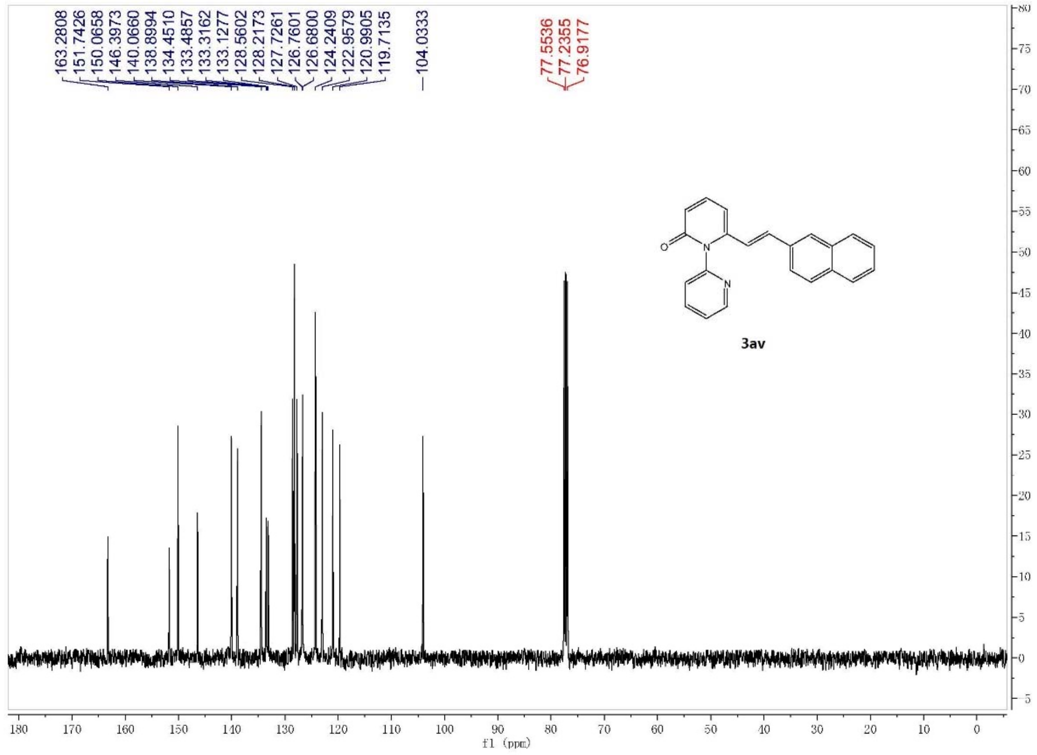

^{1}H and ^{13}C{^{1}H} NMR spectra of compound 3aw in $CDCl_3$

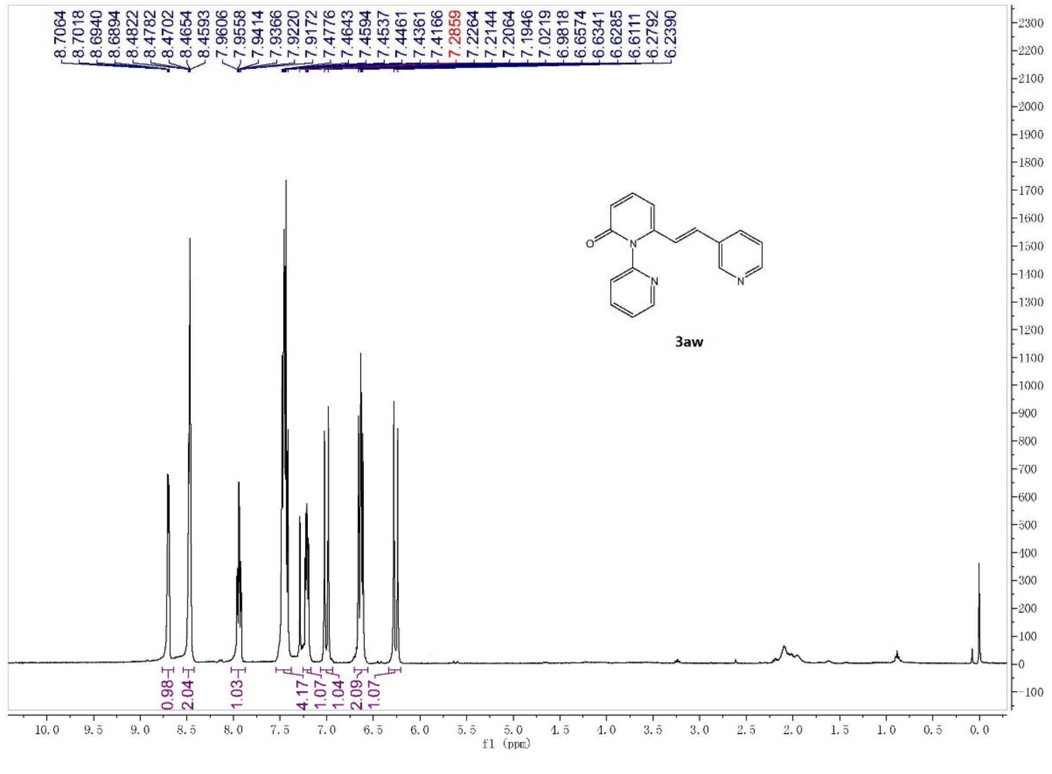

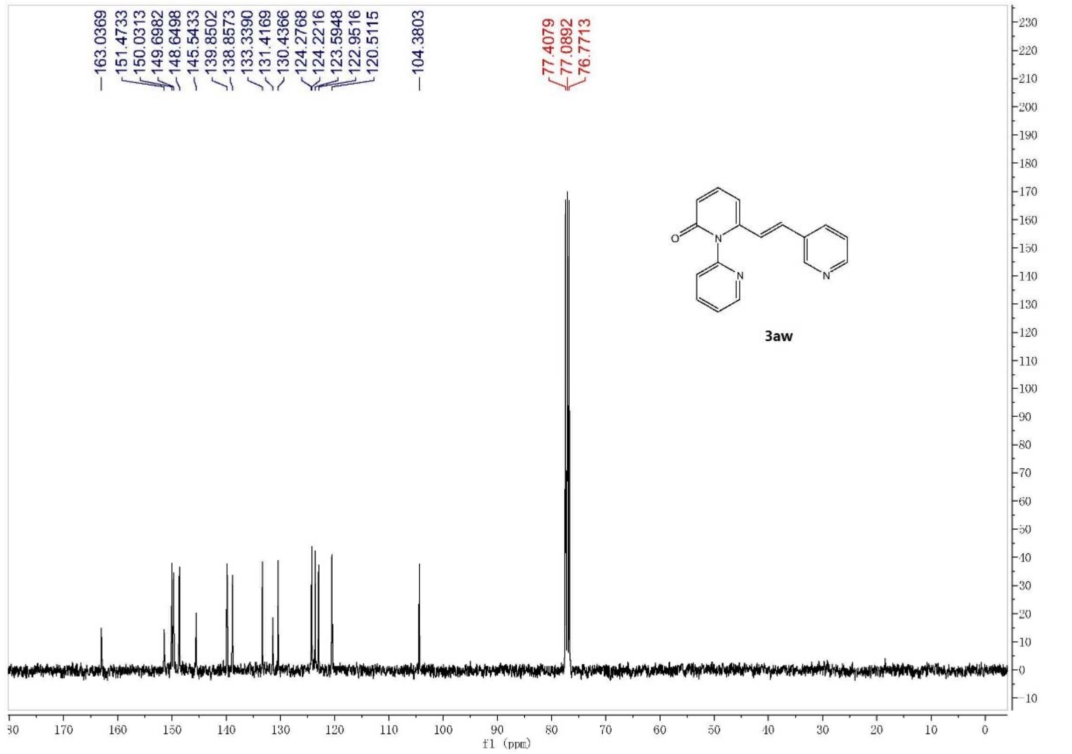

^{1}H and ^{13}C{^{1}H} NMR spectra of compound 3ax in $CDCl_3$

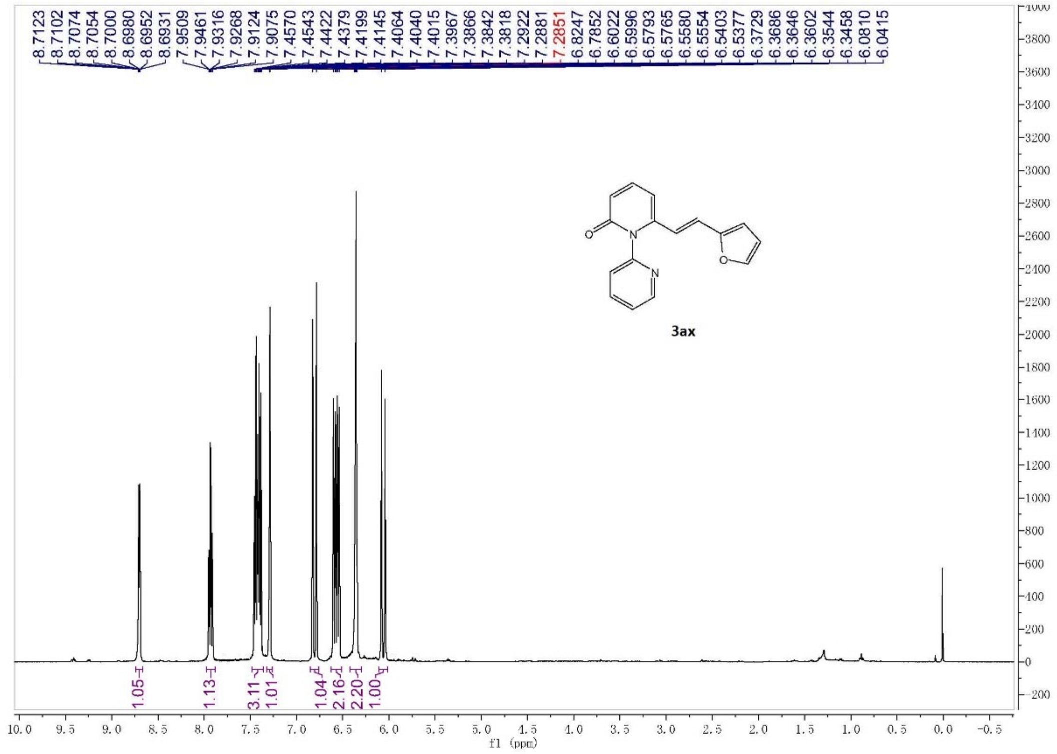

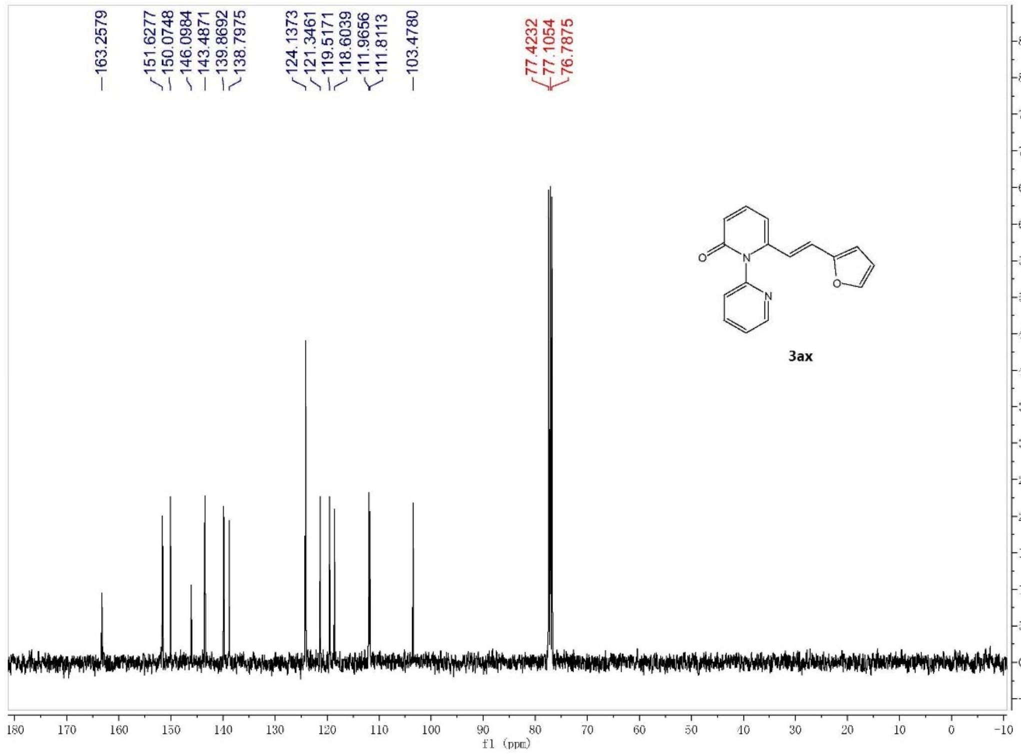

^{1}H and ^{13}C{^{1}H} NMR spectra of compound 3ay in $CDCl_3$

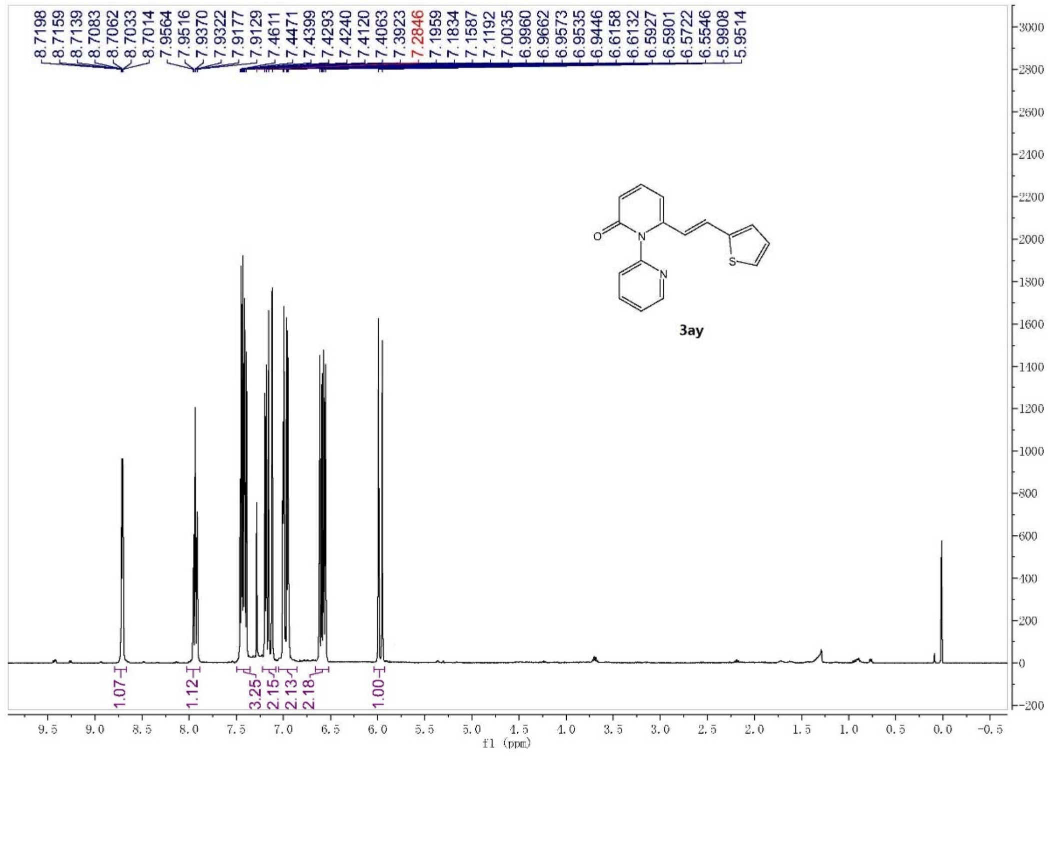

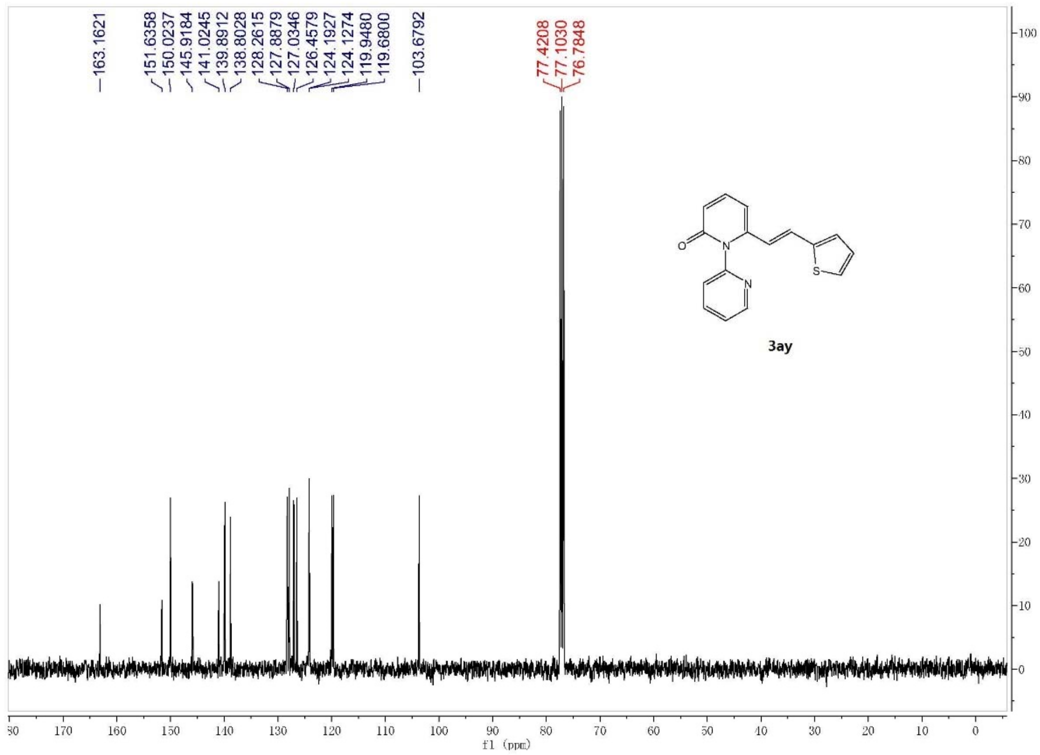

^{1}H and ^{13}C{^{1}H} NMR spectra of compound 3az in $CDCl_3$

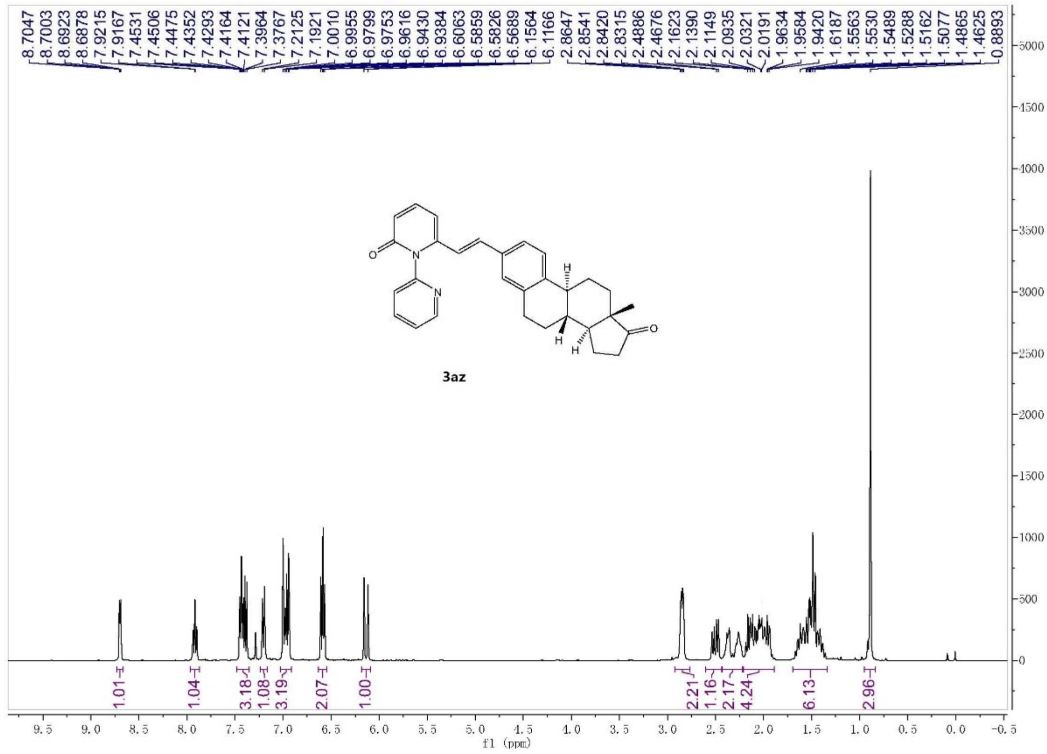

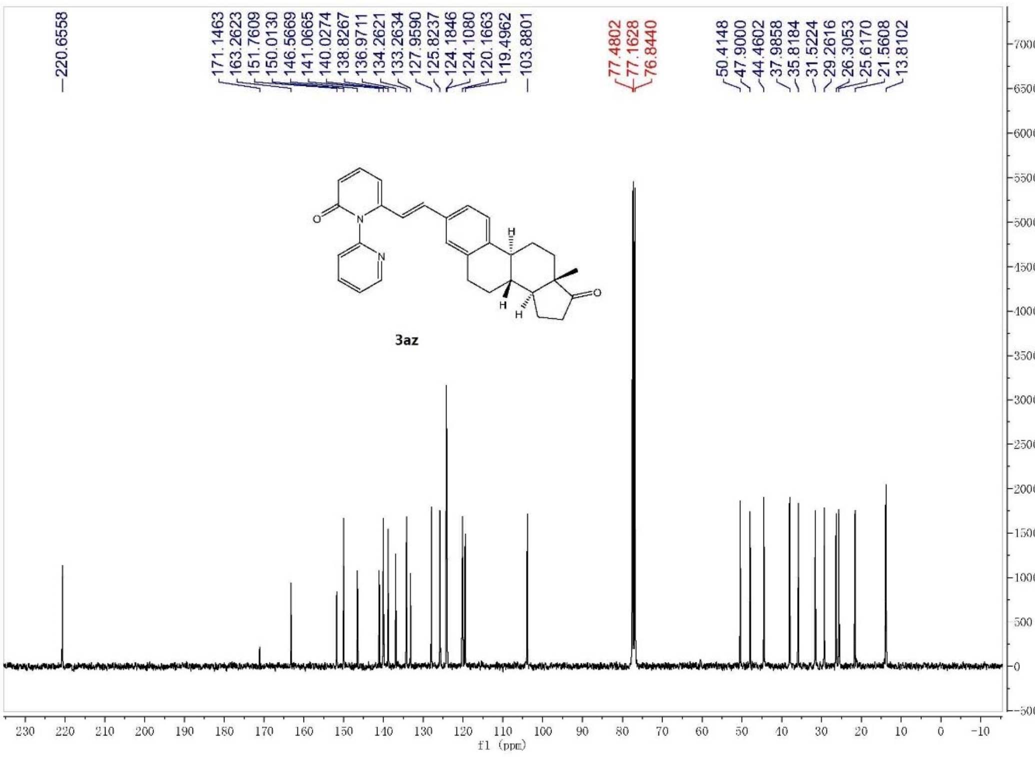

^{1}H and $^{13}C\{^{1}H\}$ NMR spectra of compound 3ba in $CDCl_3$

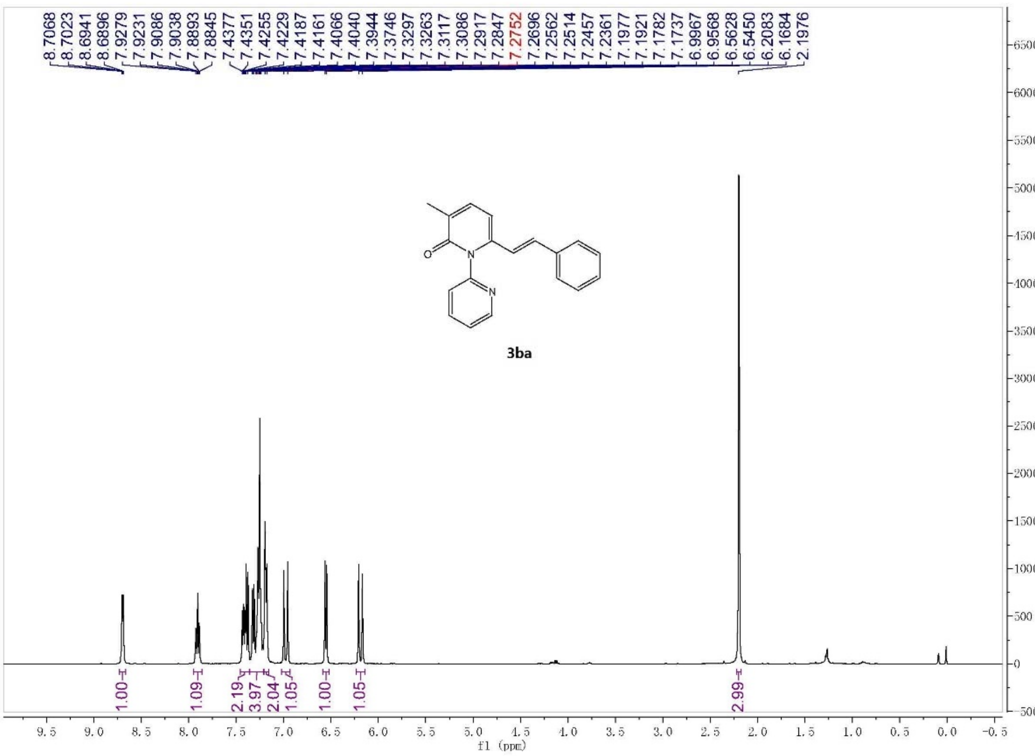

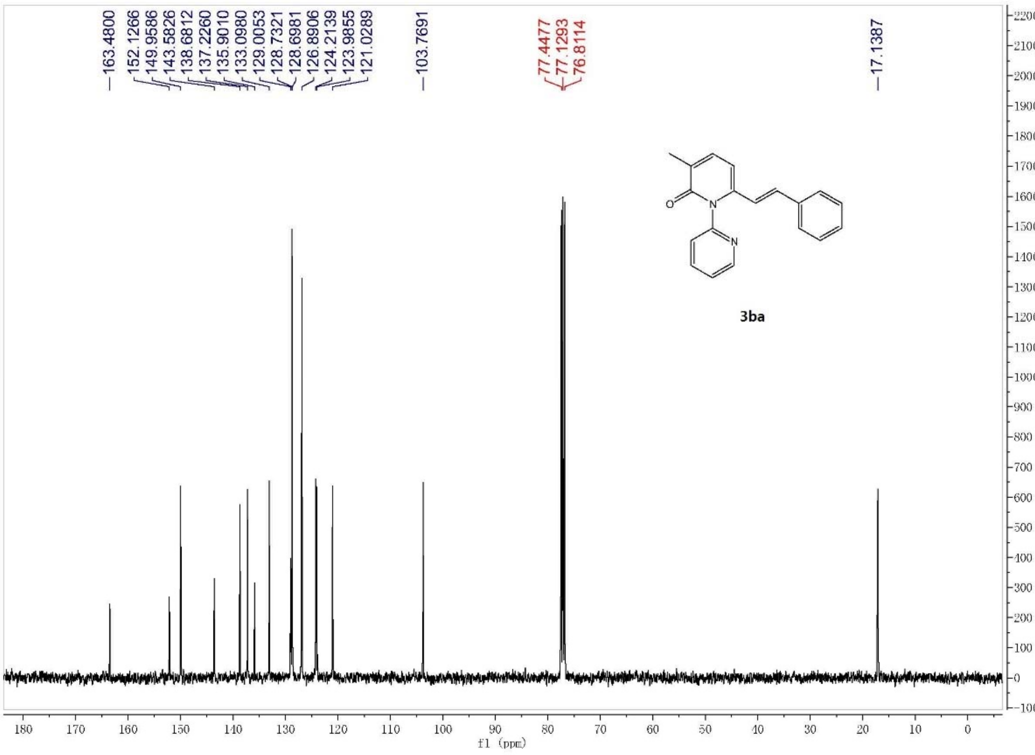

^{1}H and $^{13}C\{^{1}H\}$ NMR spectra of compound 3ca in $CDCl_3$

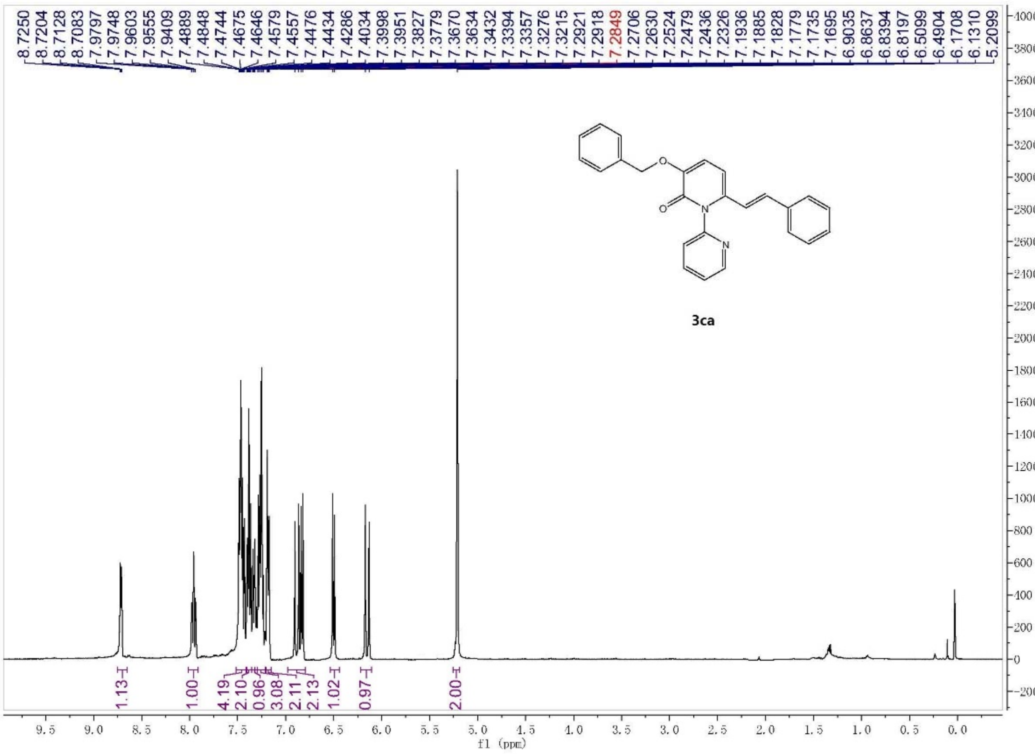

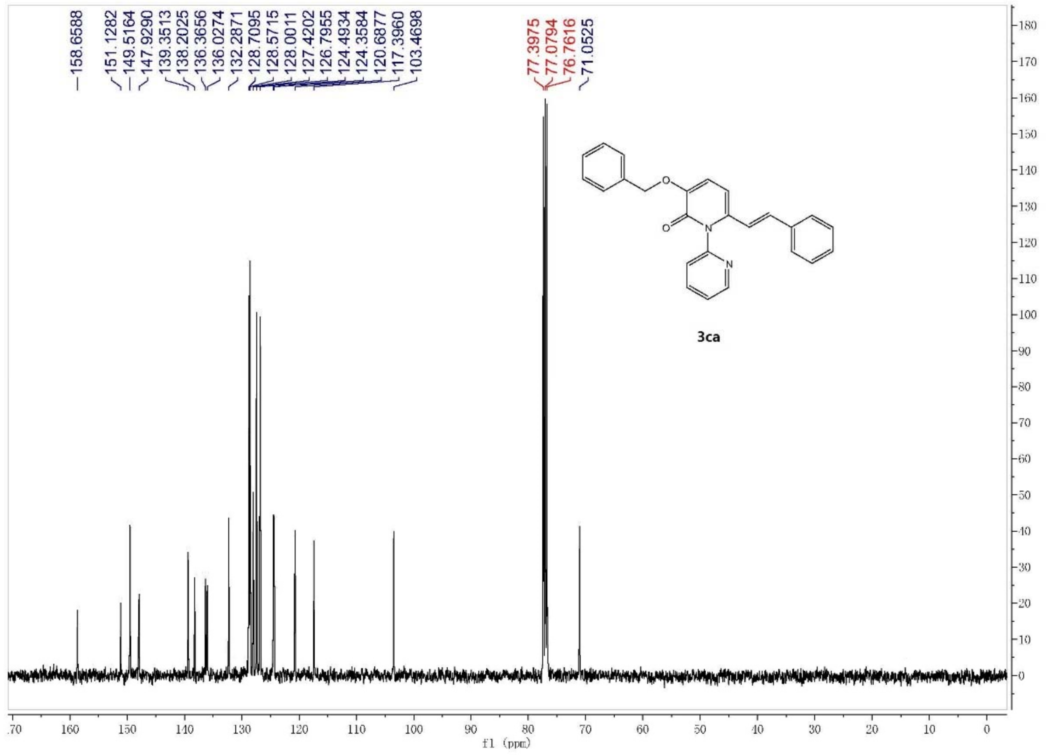

^{1}H and $^{13}C\{^{1}H\}$ NMR spectra of compound 3da in $CDCl_3$

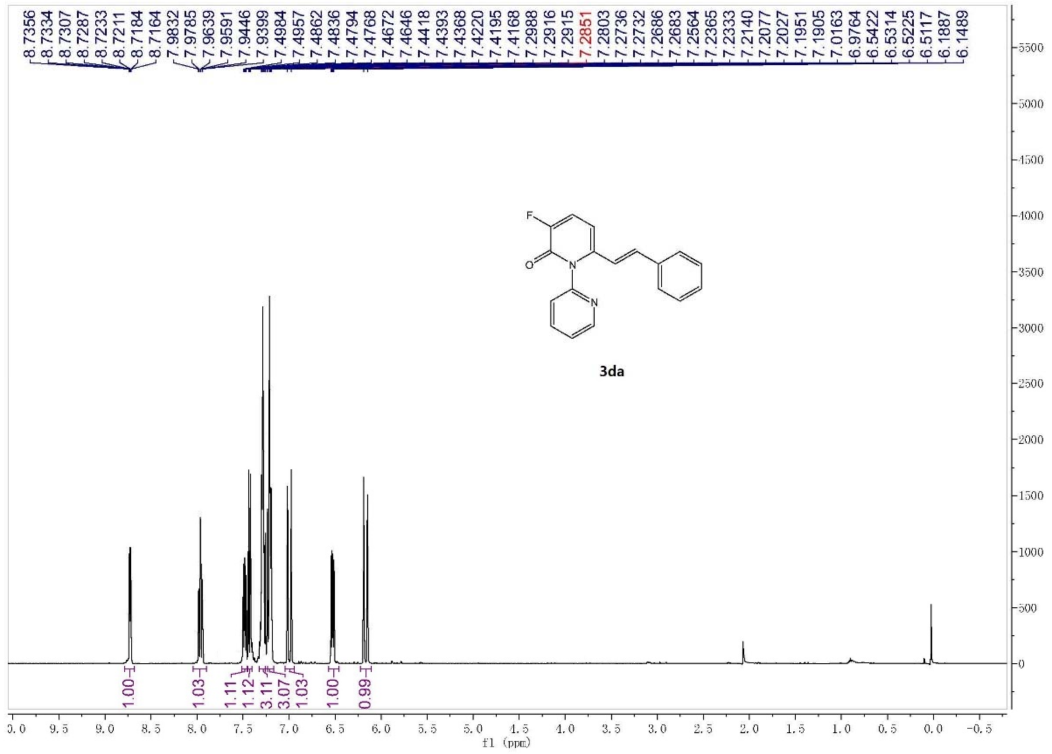

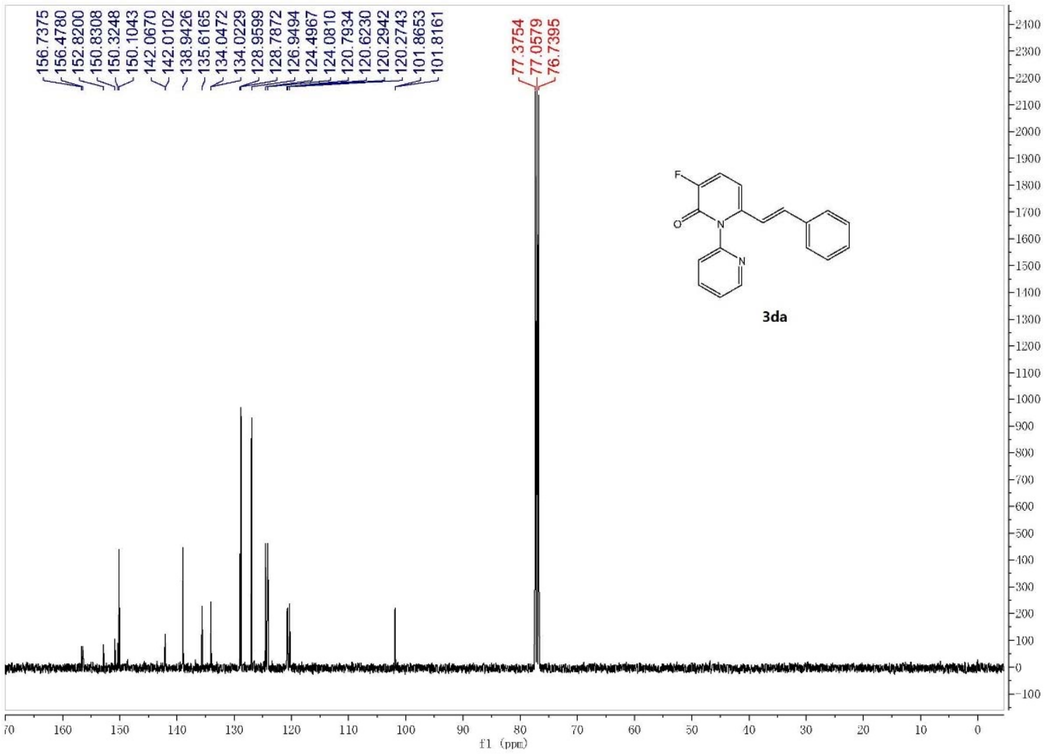

^1H and $^{13}C\{^1H\}$ NMR spectra of compound 3ea in $CDCl_3$

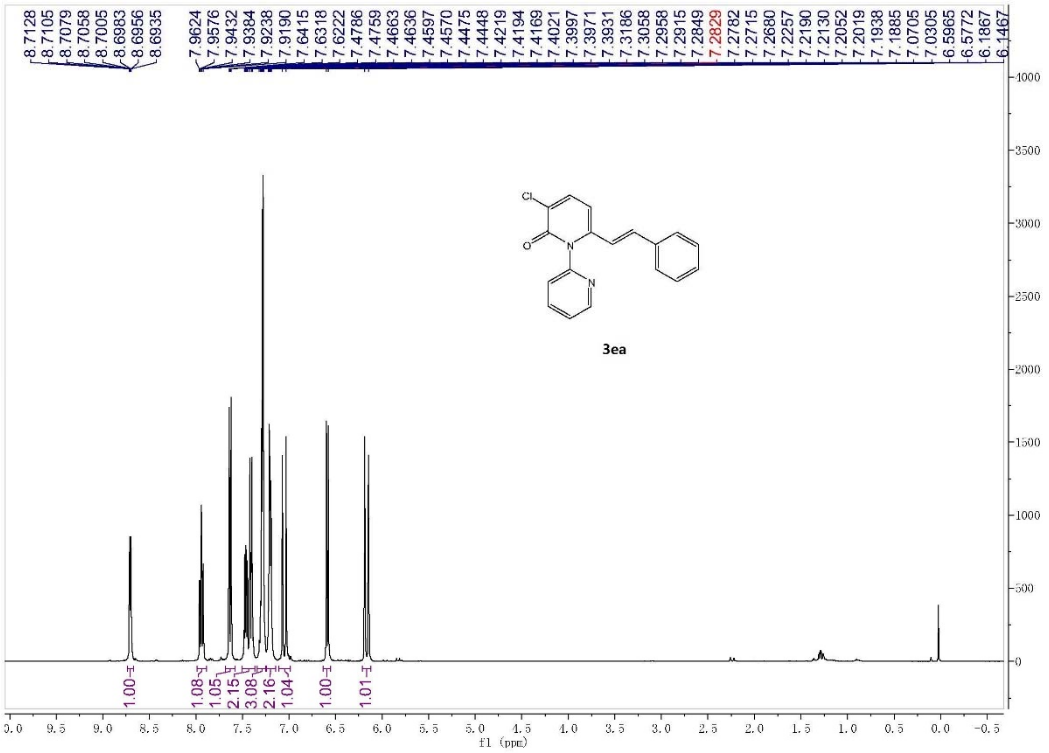

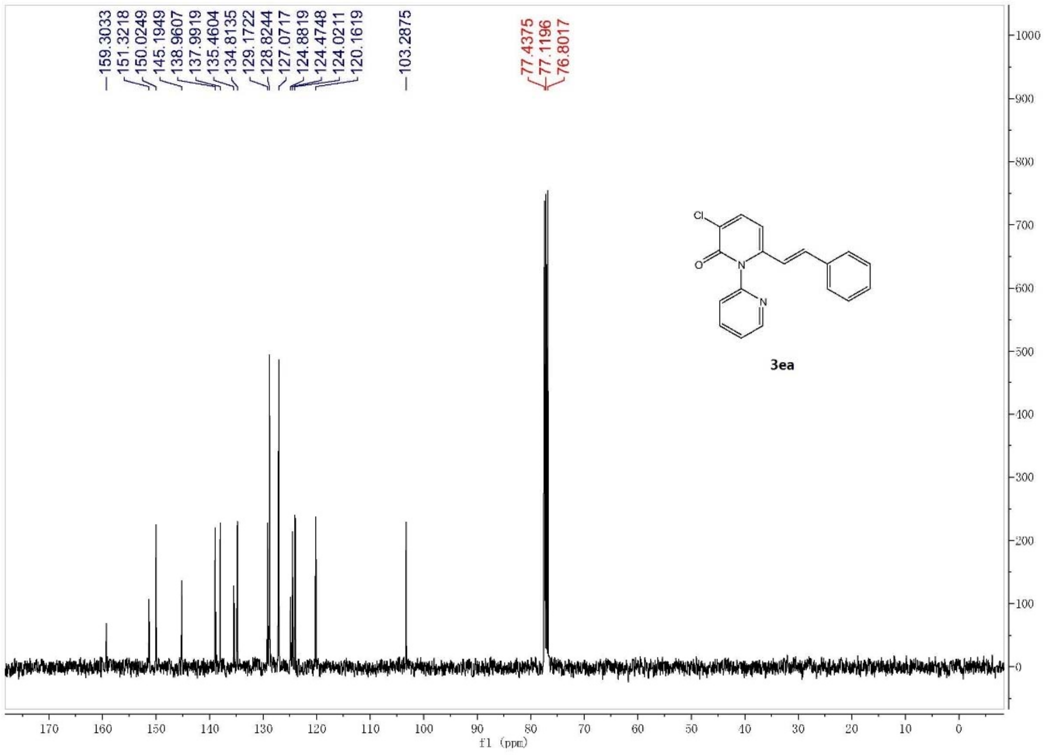

^1H and $^{13}C\{^1H\}$ NMR spectra of compound 3fa in $CDCl_3$

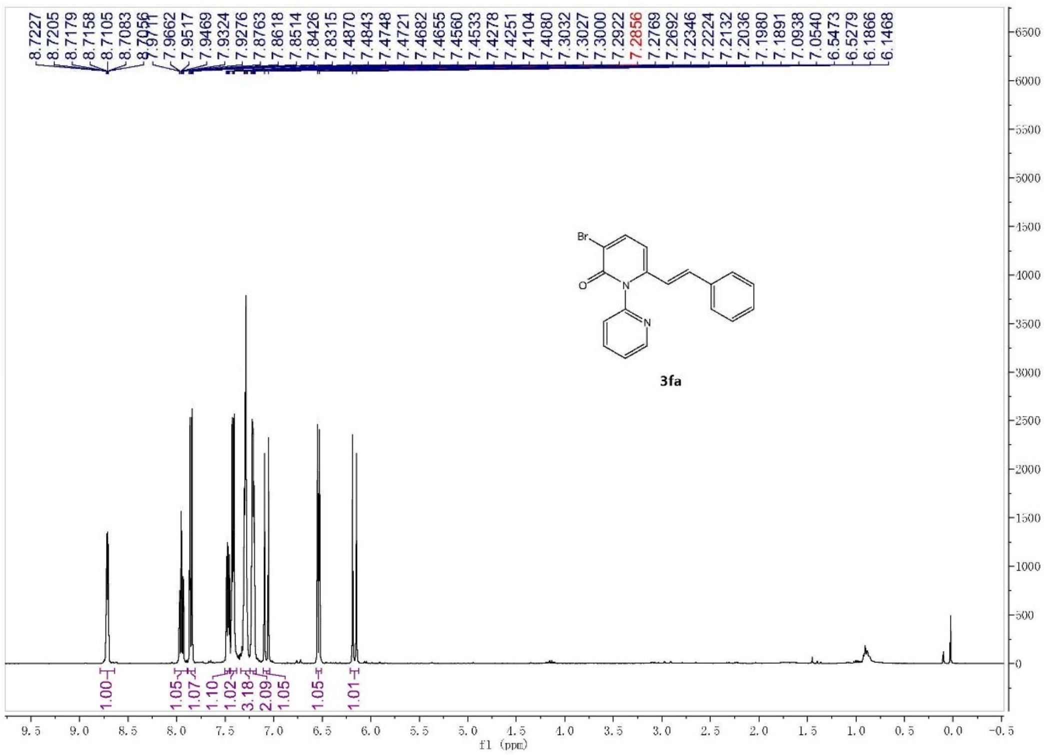

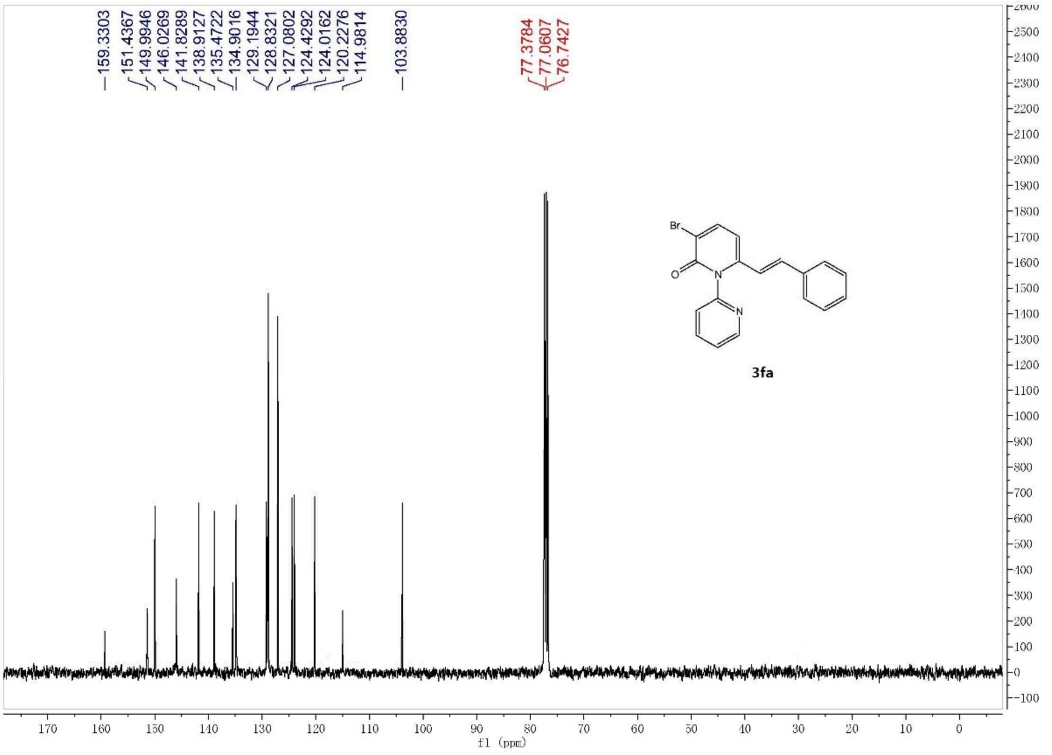

^{1}H and $^{13}C\{^{1}H\}$ NMR spectra of compound 3ga in $CDCl_3$

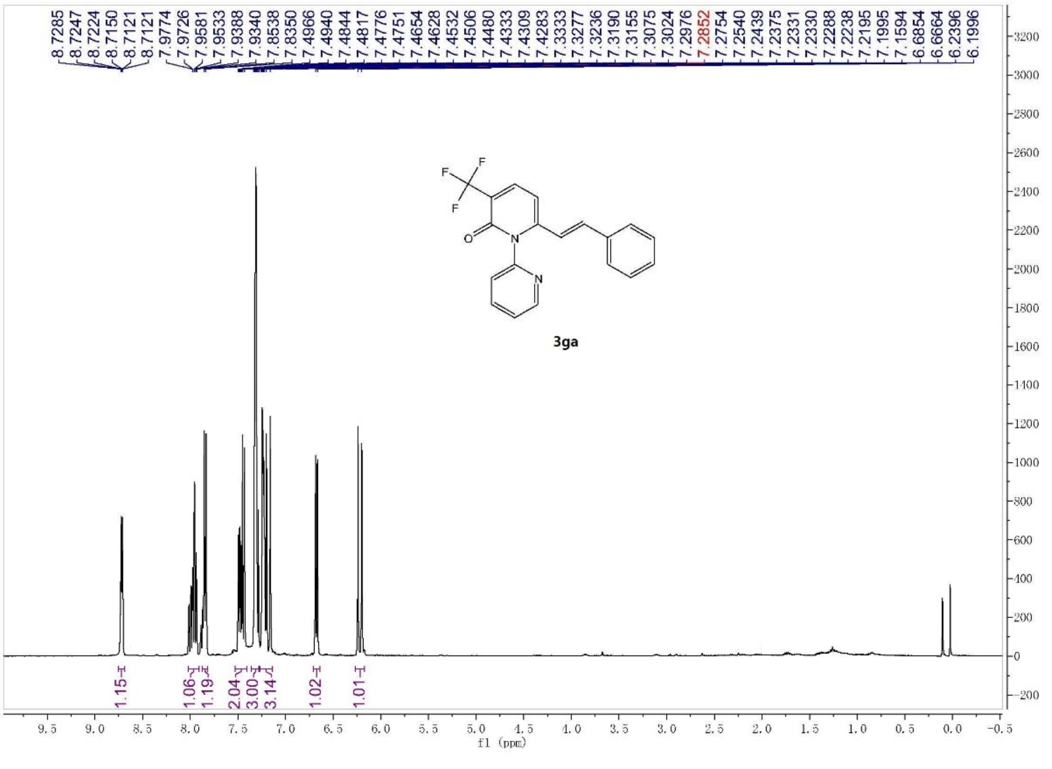

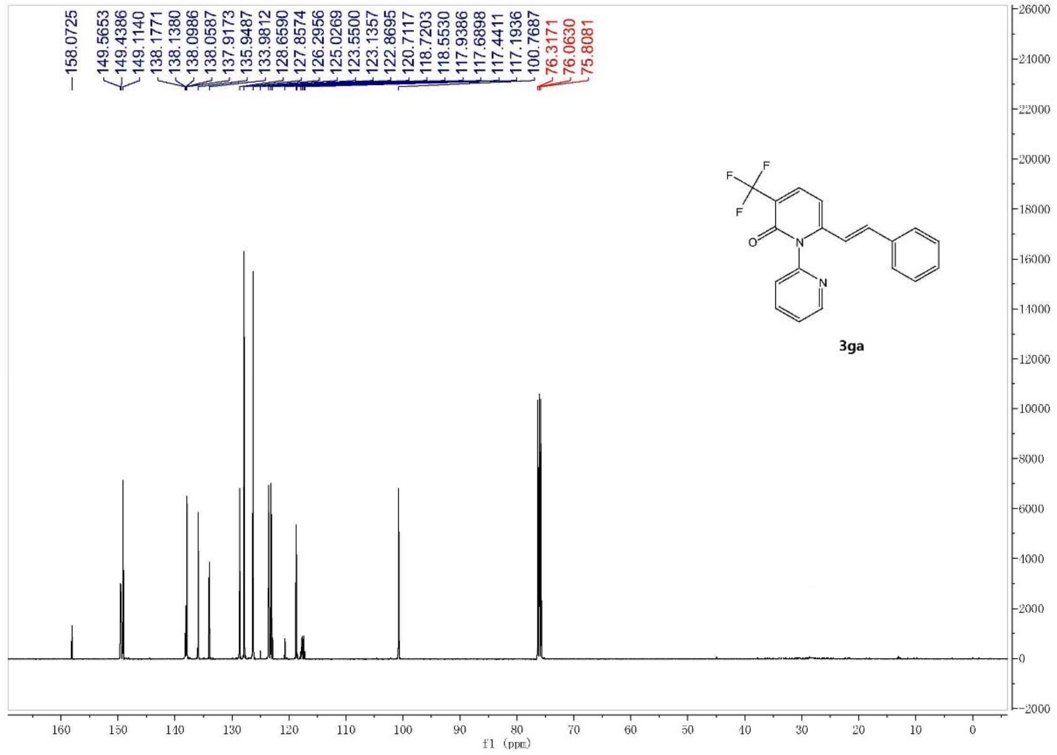

^1H and $^{13}C\{^1H\}$ NMR spectra of compound 3ha in $CDCl_3$

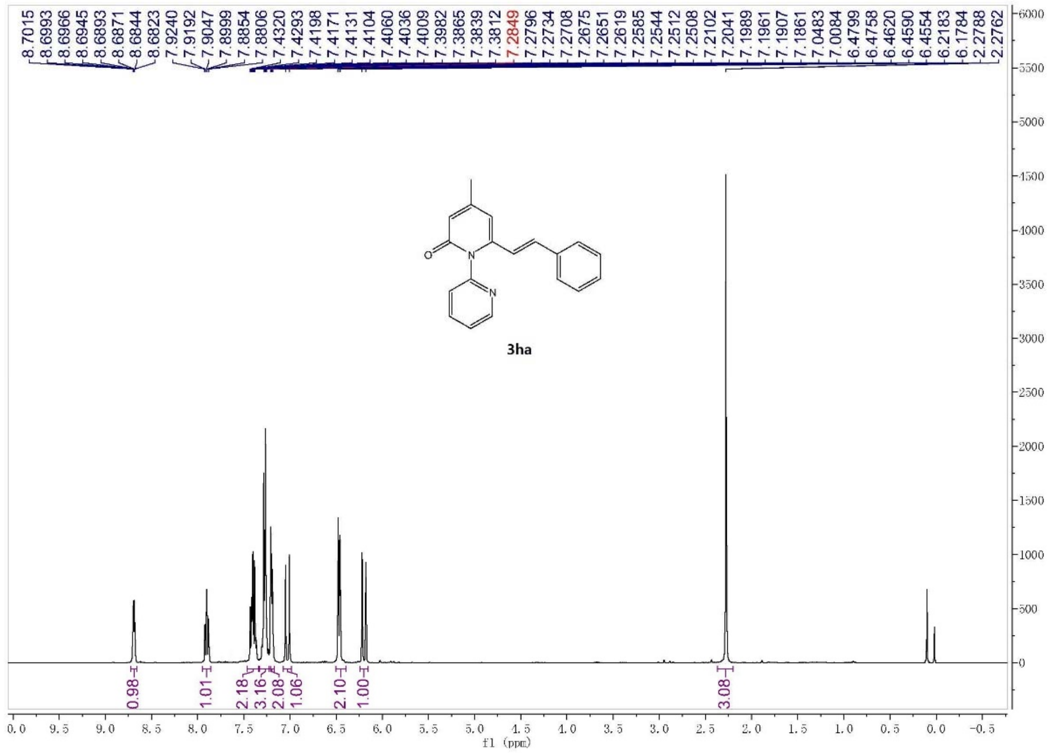

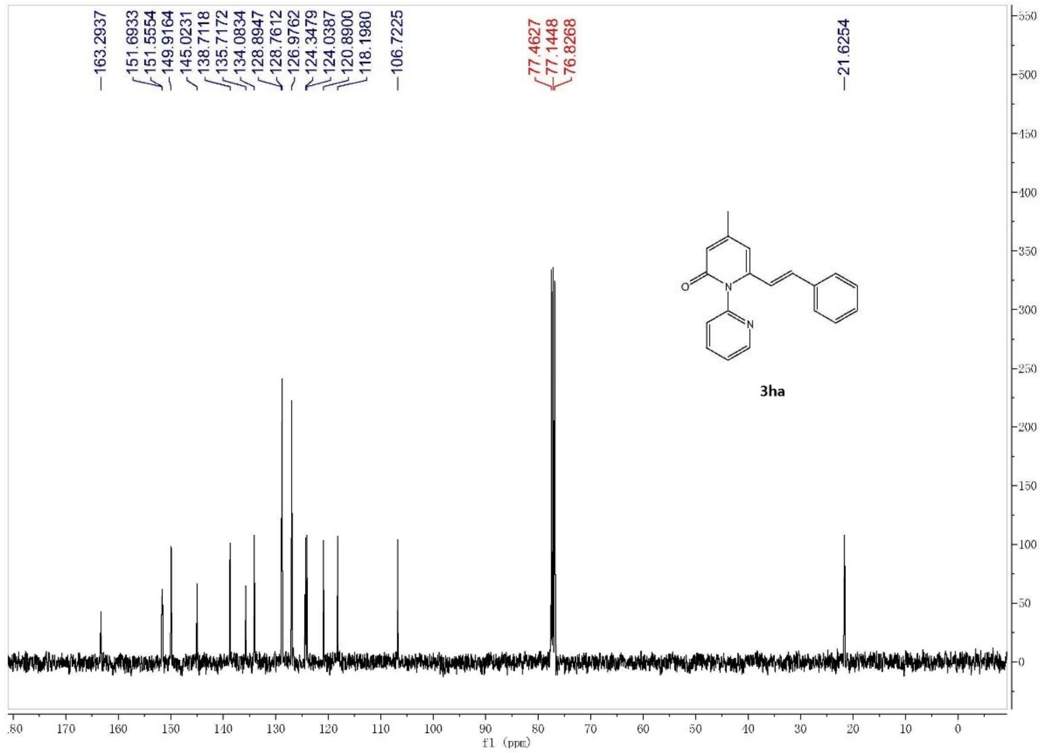

^1H and $^{13}C\{^1H\}$ NMR spectra of compound 3ia in $CDCl_3$

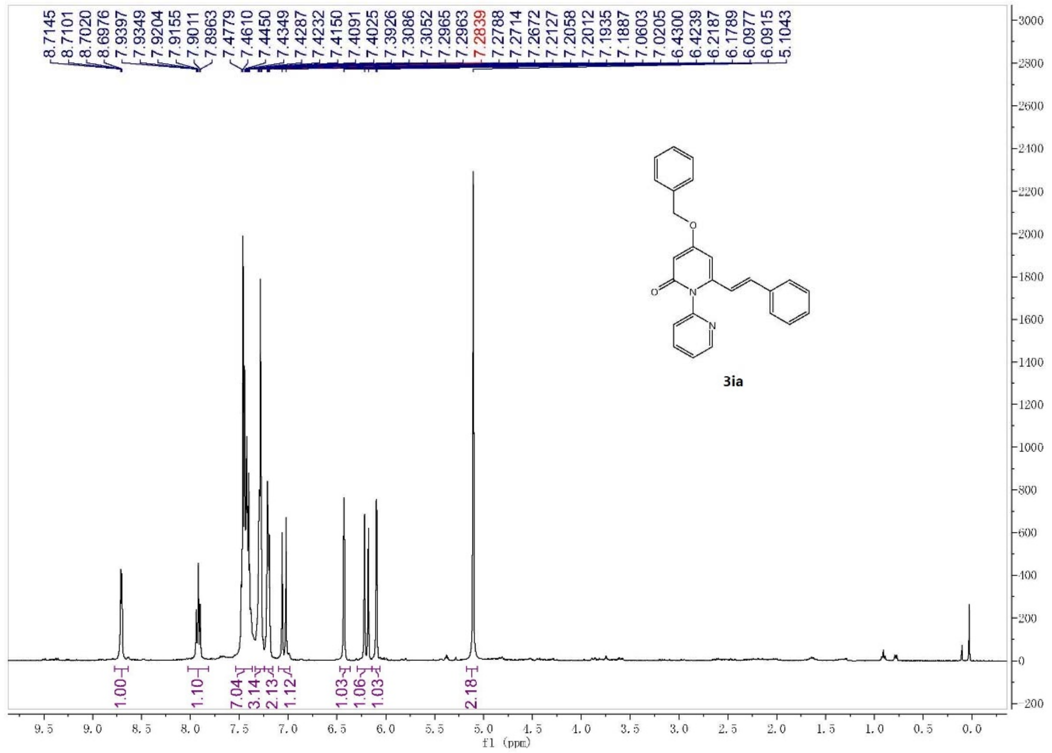

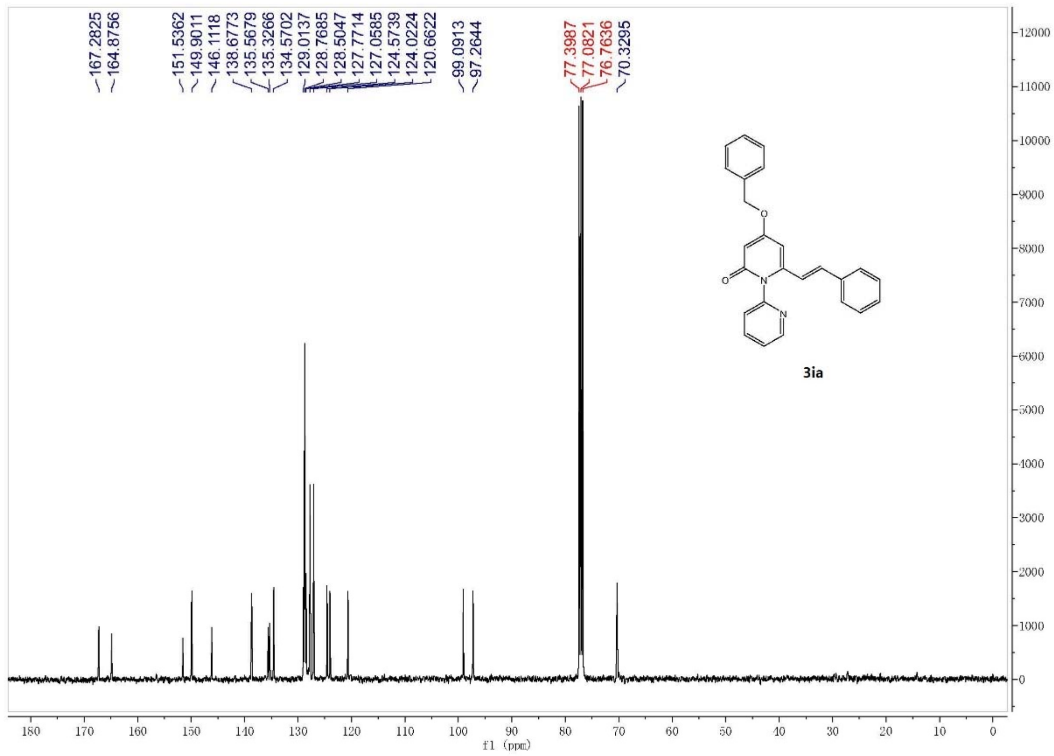

^{1}H and ^{13}C{^{1}H} NMR spectra of compound 3ja in $CDCl_3$

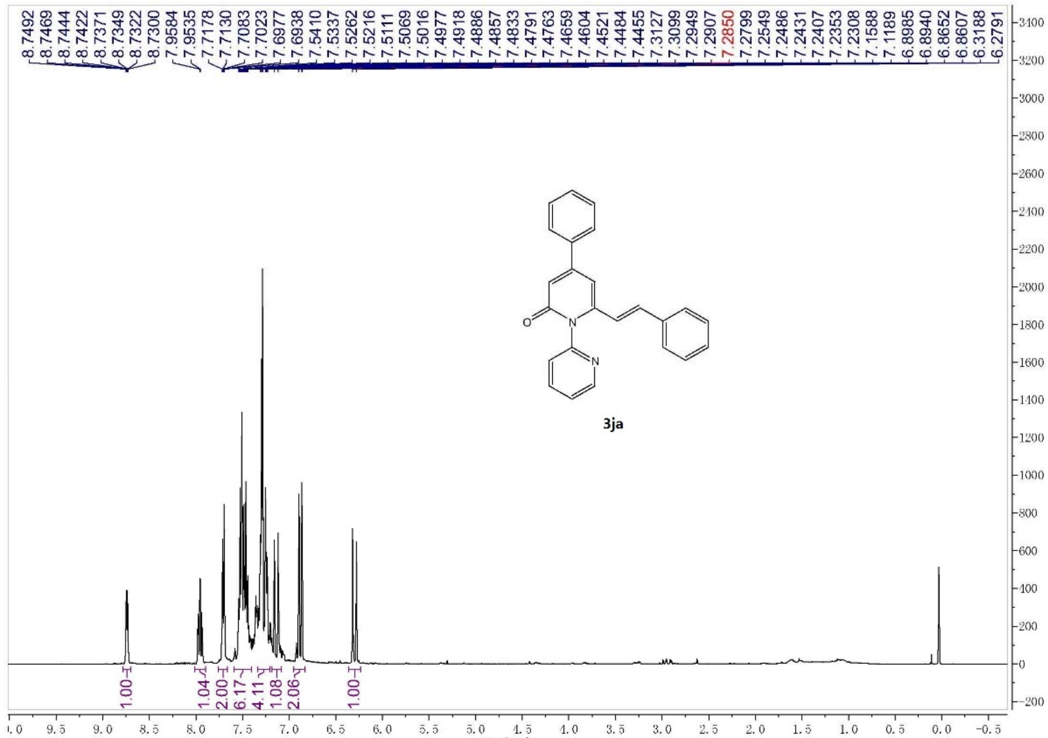

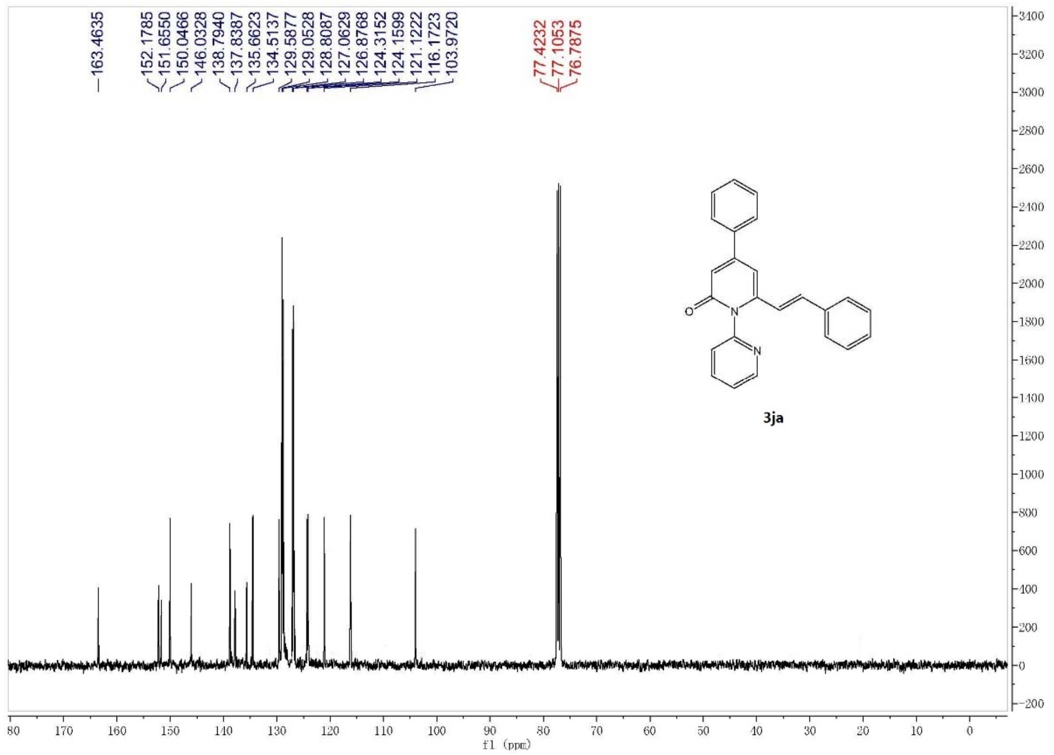

^{1}H and $^{13}C\{^{1}H\}$ NMR spectra of compound 3ka in $CDCl_3$

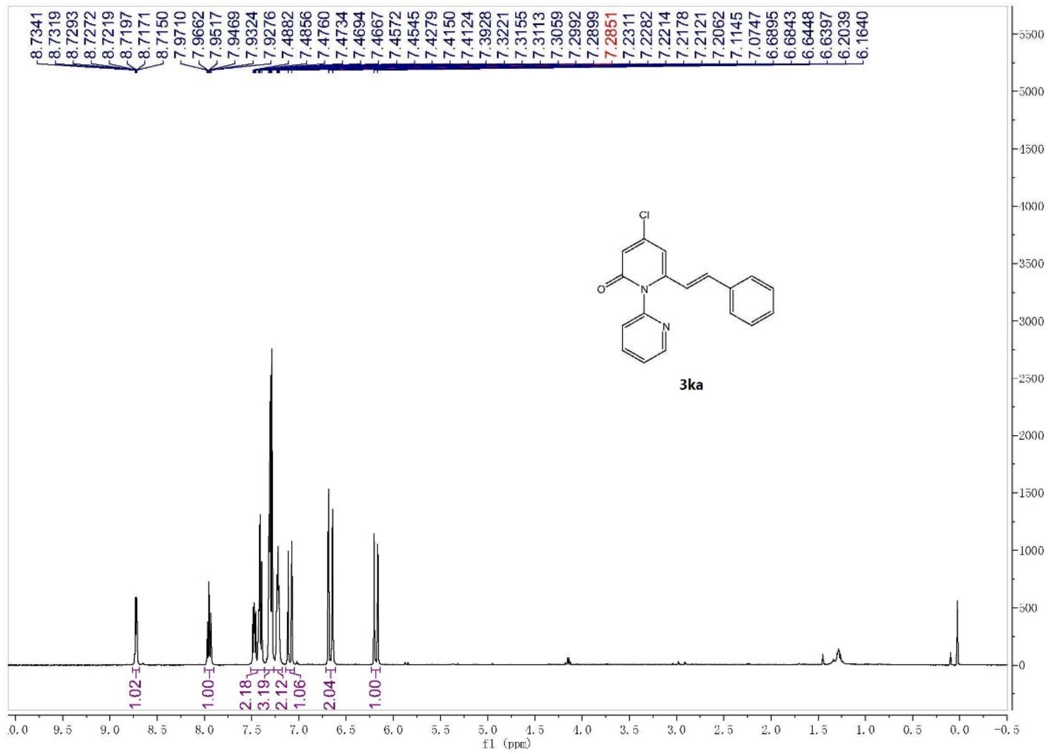

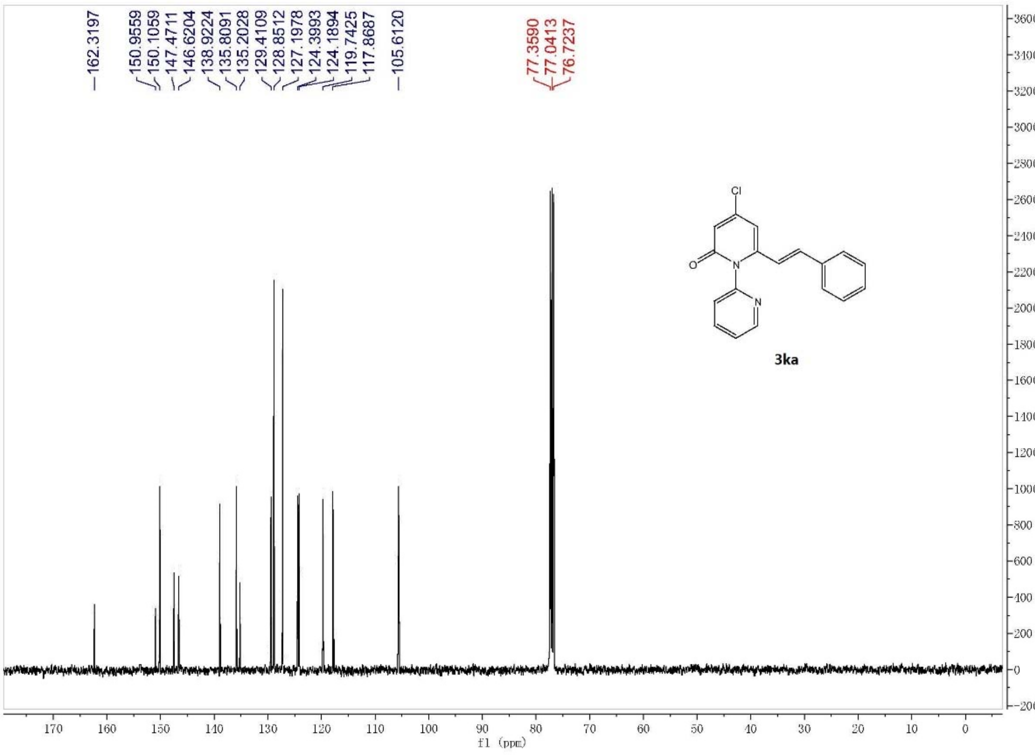

^{1}H and $^{13}C\{^{1}H\}$ NMR spectra of compound 3la in $CDCl_3$

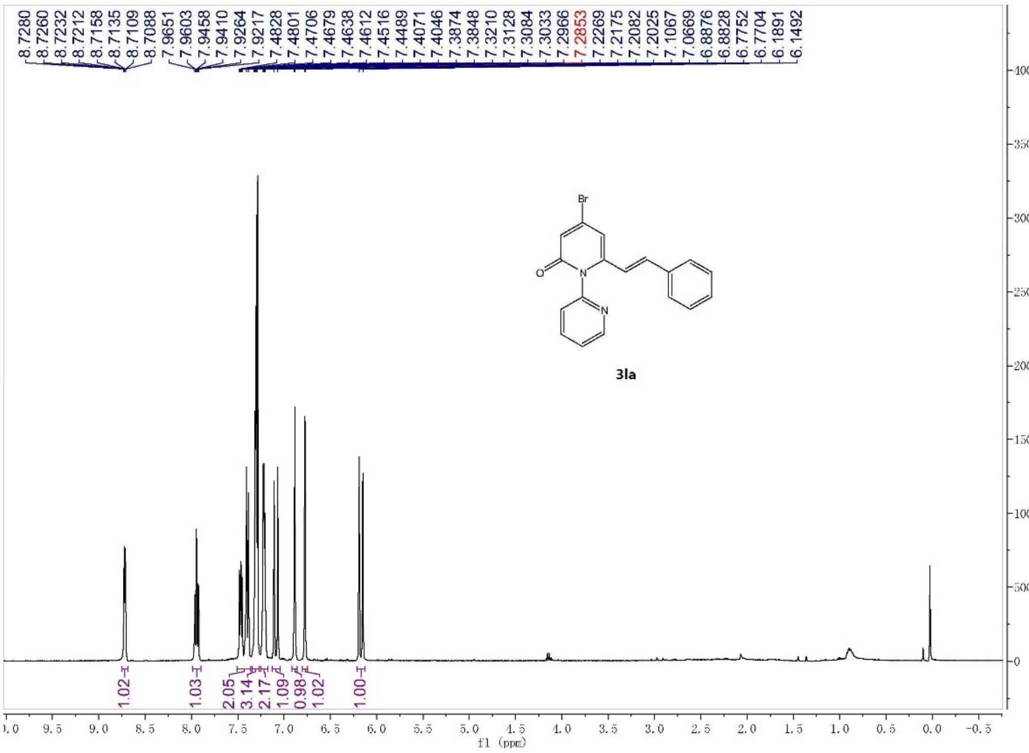

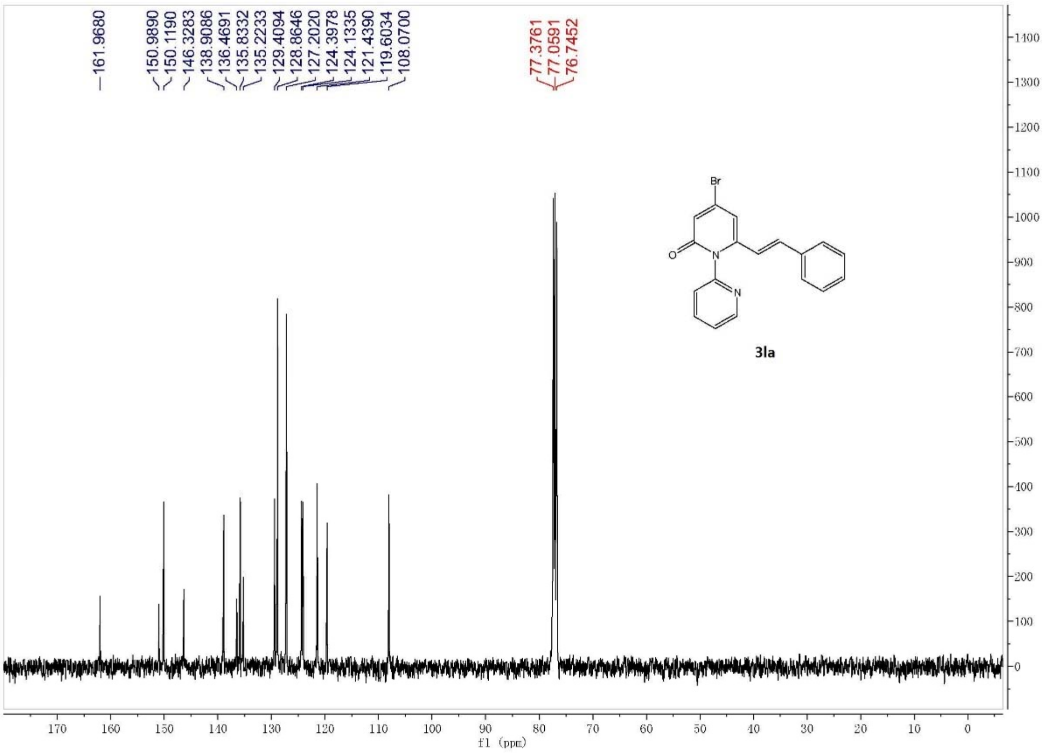

^1H and $^{13}C\{^1H\}$ NMR spectra of compound 3ma in $CDCl_3$

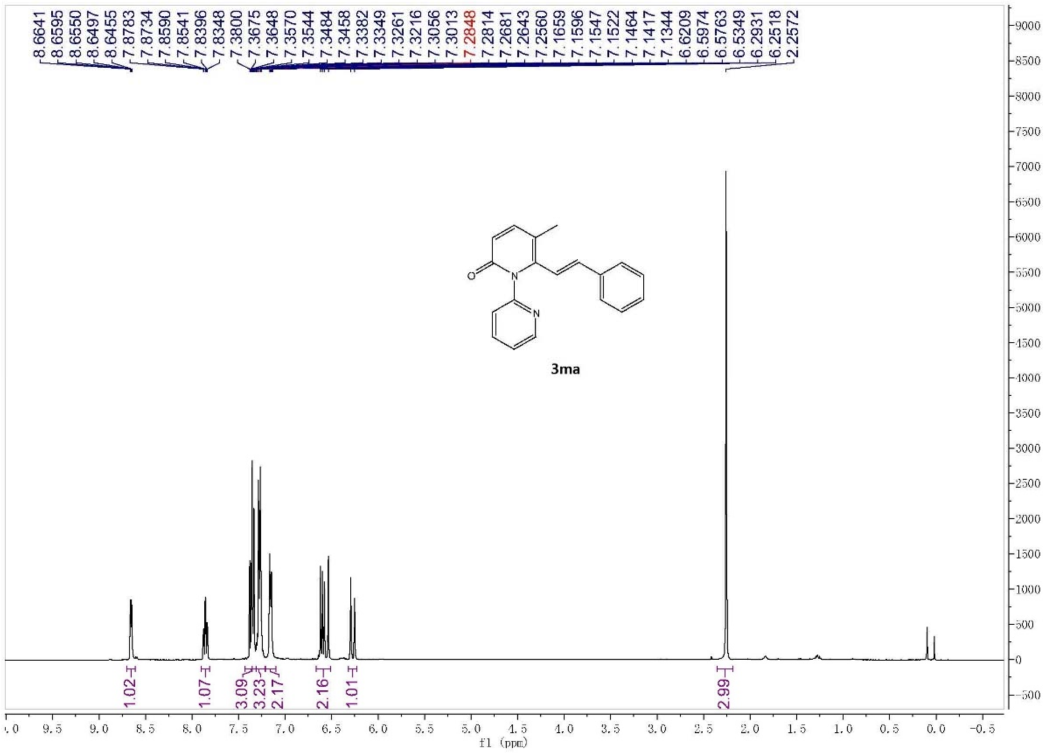

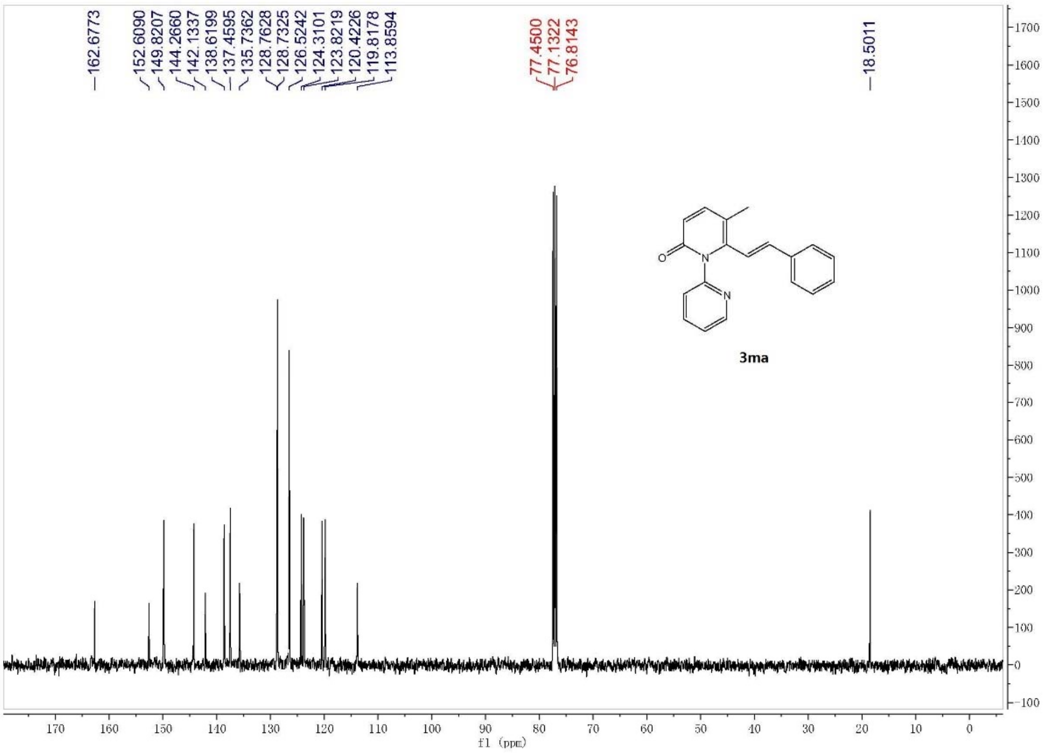

^1H and $^{13}C\{^1H\}$ NMR spectra of compound 3na in $CDCl_3$

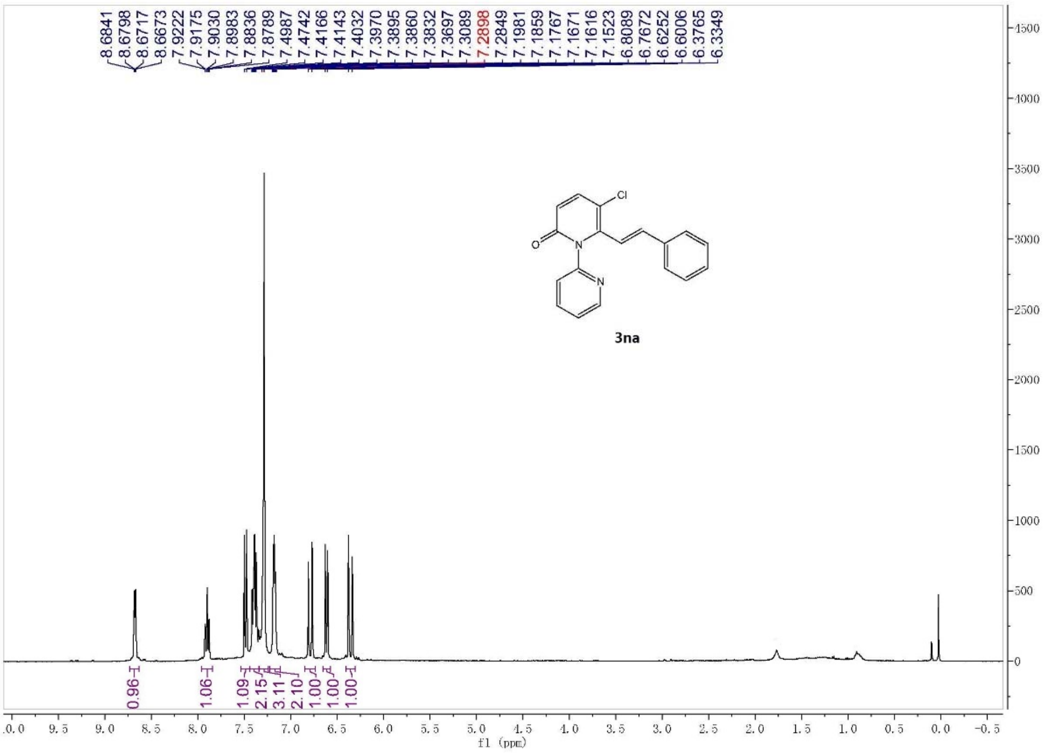

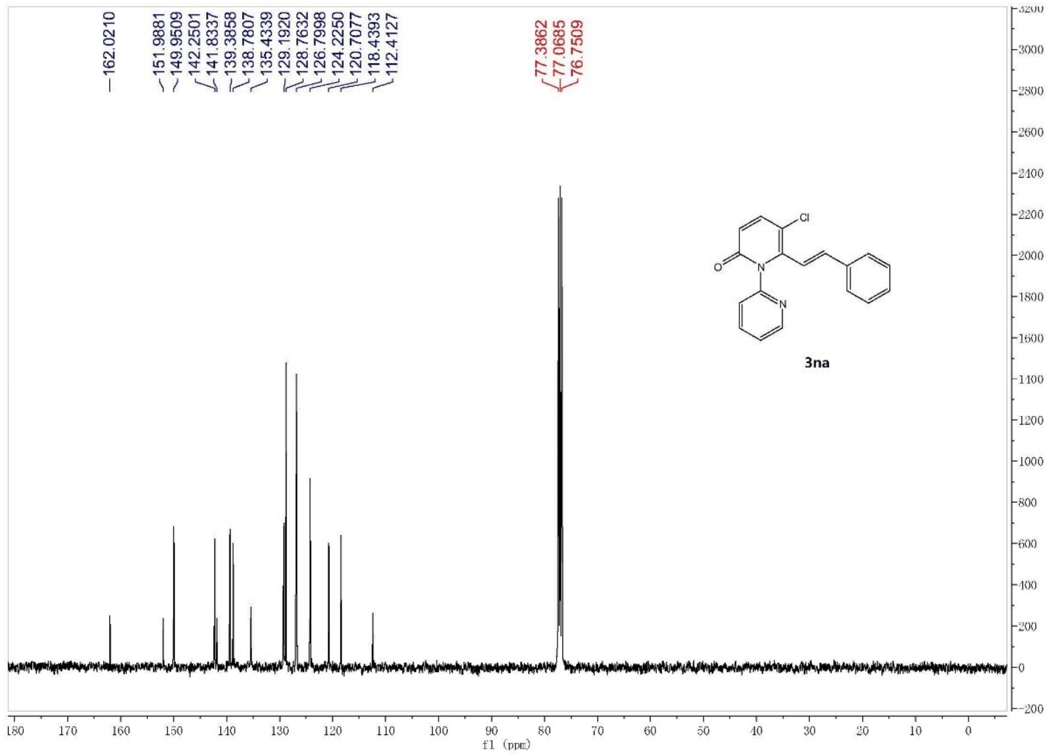

^{1}H and $^{13}C\{^{1}H\}$ NMR spectra of compound 3oa in $CDCl_3$

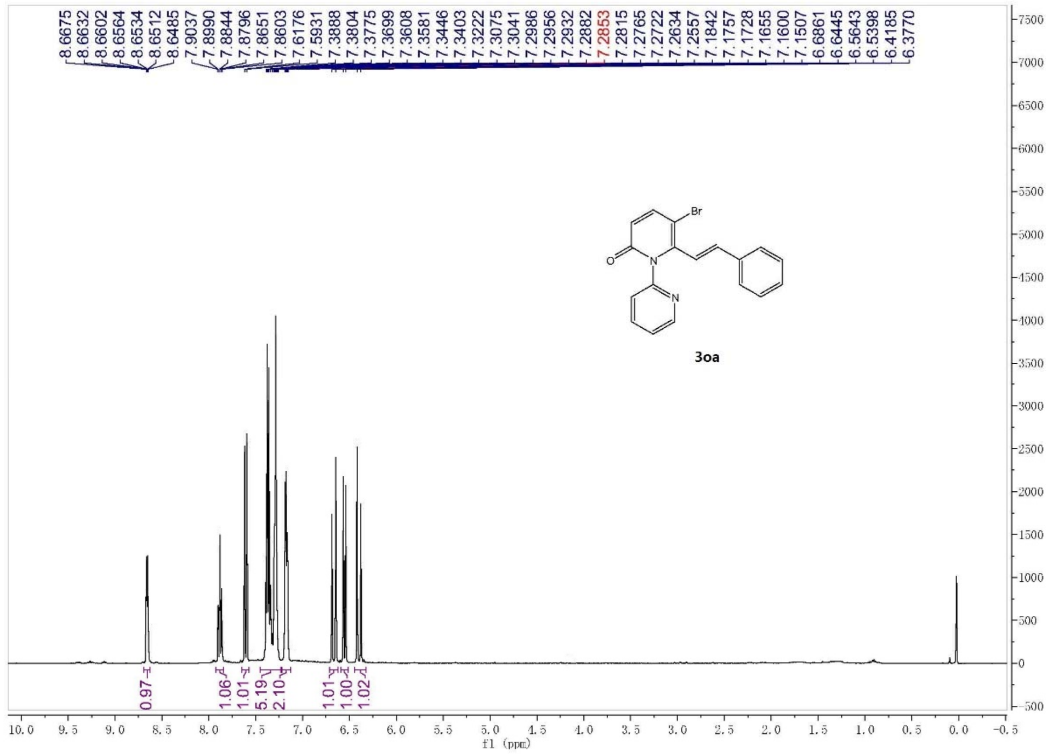

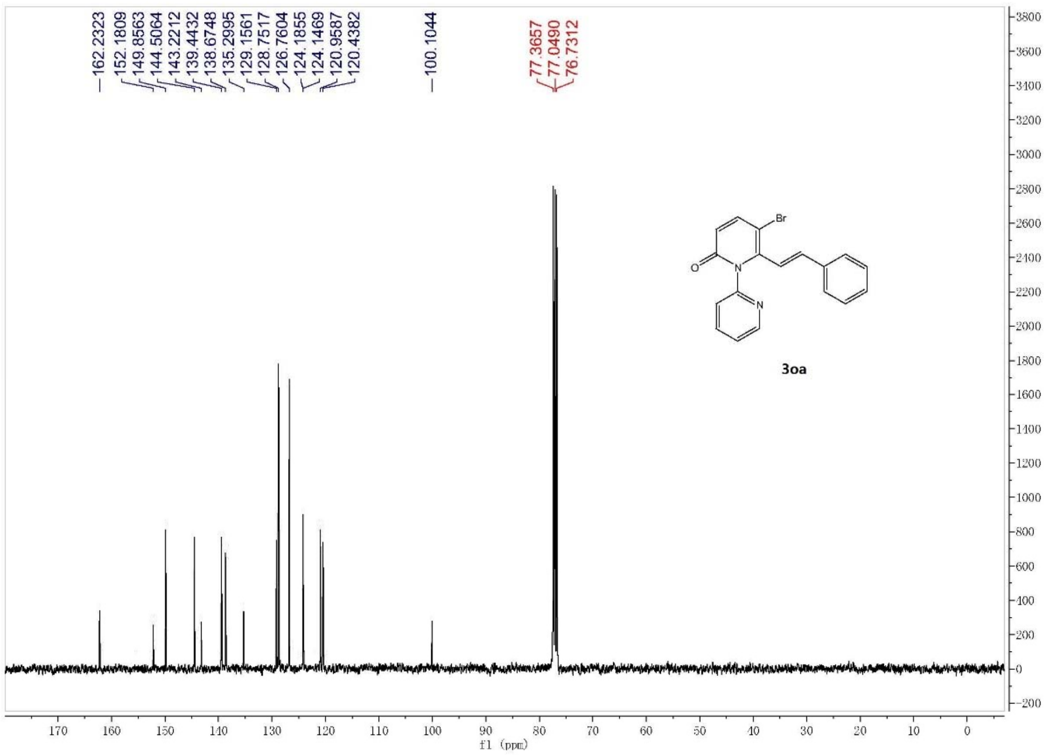

^{1}H and ^{13}C{^{1}H} NMR spectra of compound 3pa in $CDCl_3$

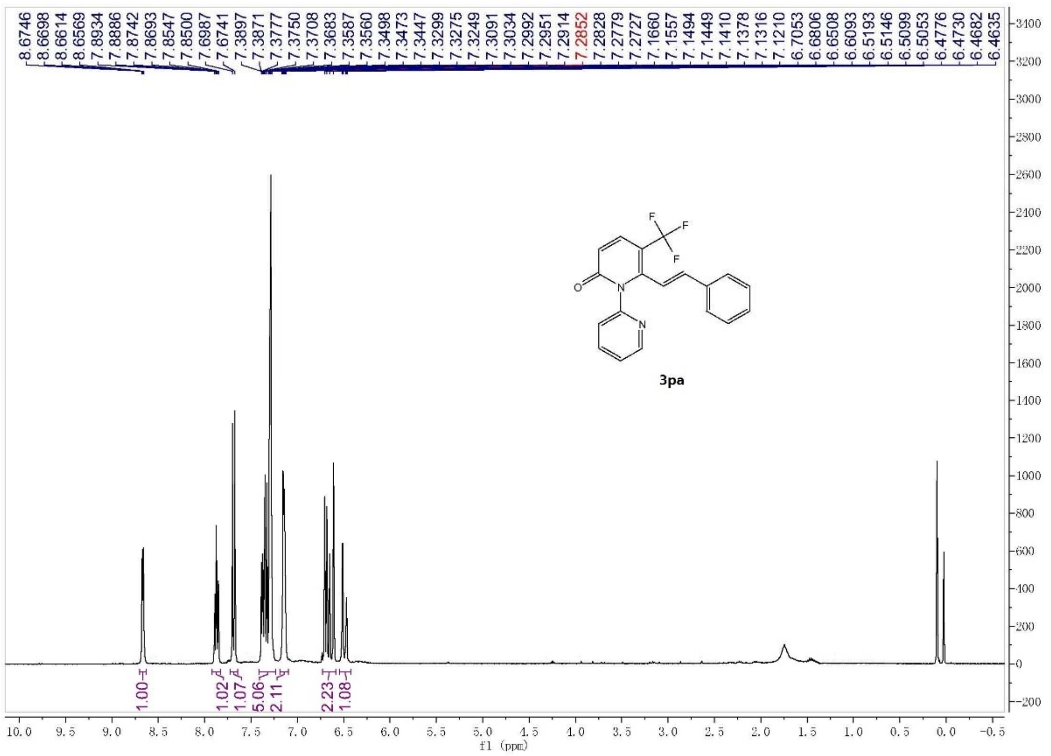

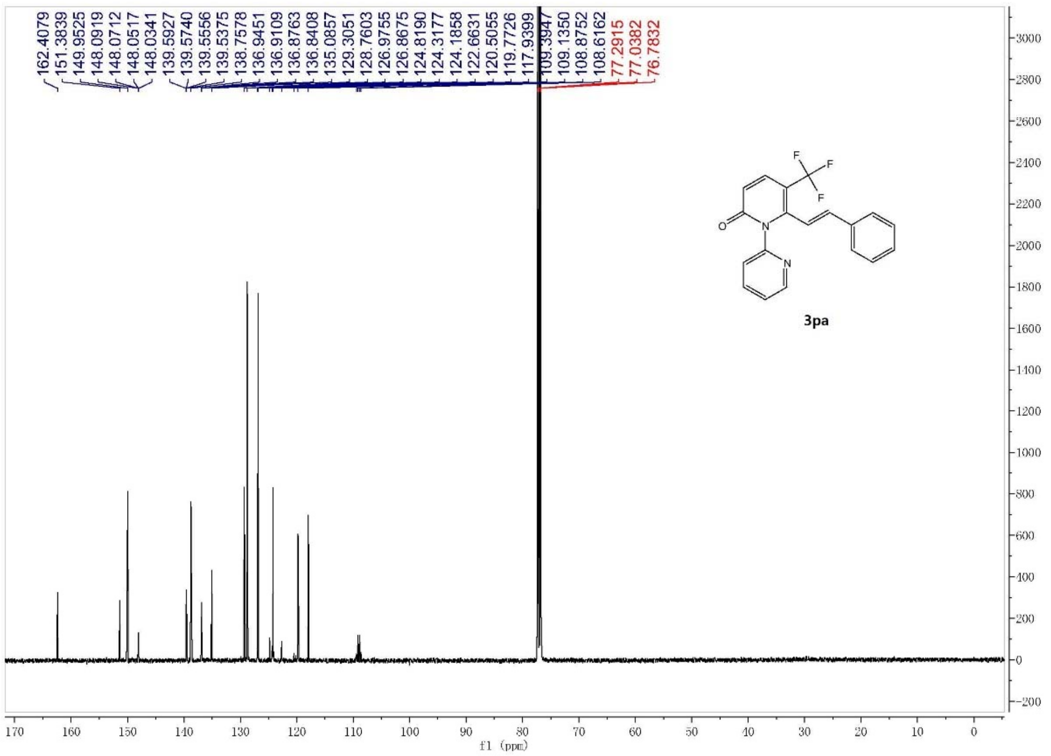

^{1}H and $^{13}C\{^{1}H\}$ NMR spectra of compound 3qa in $CDCl_3$

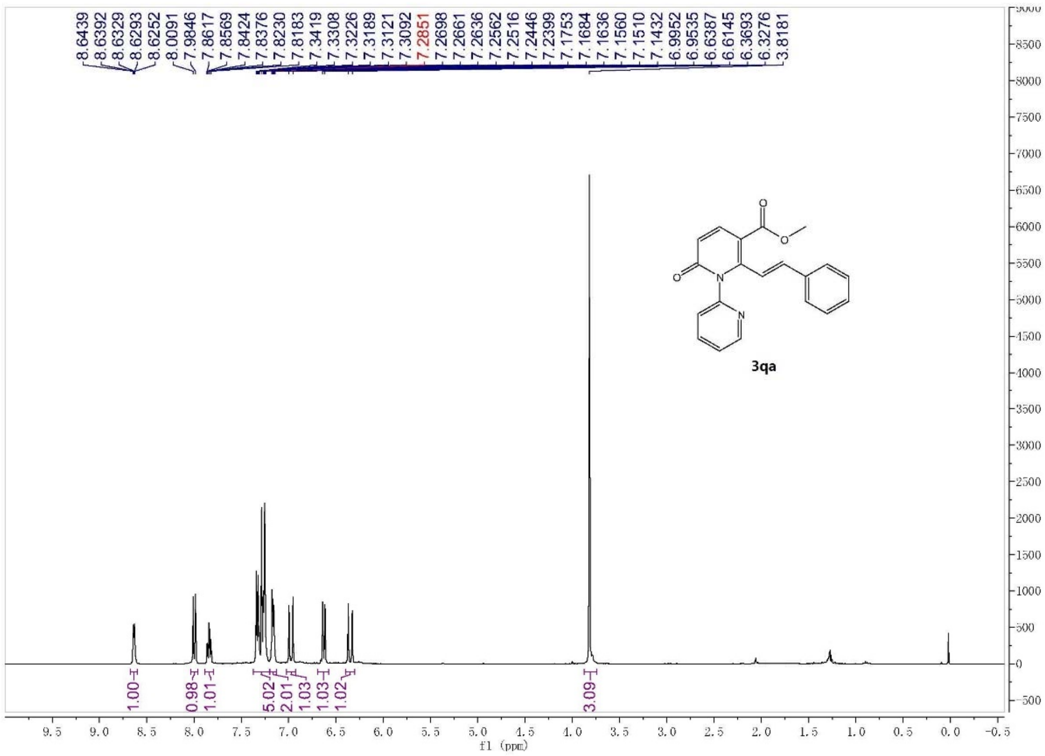

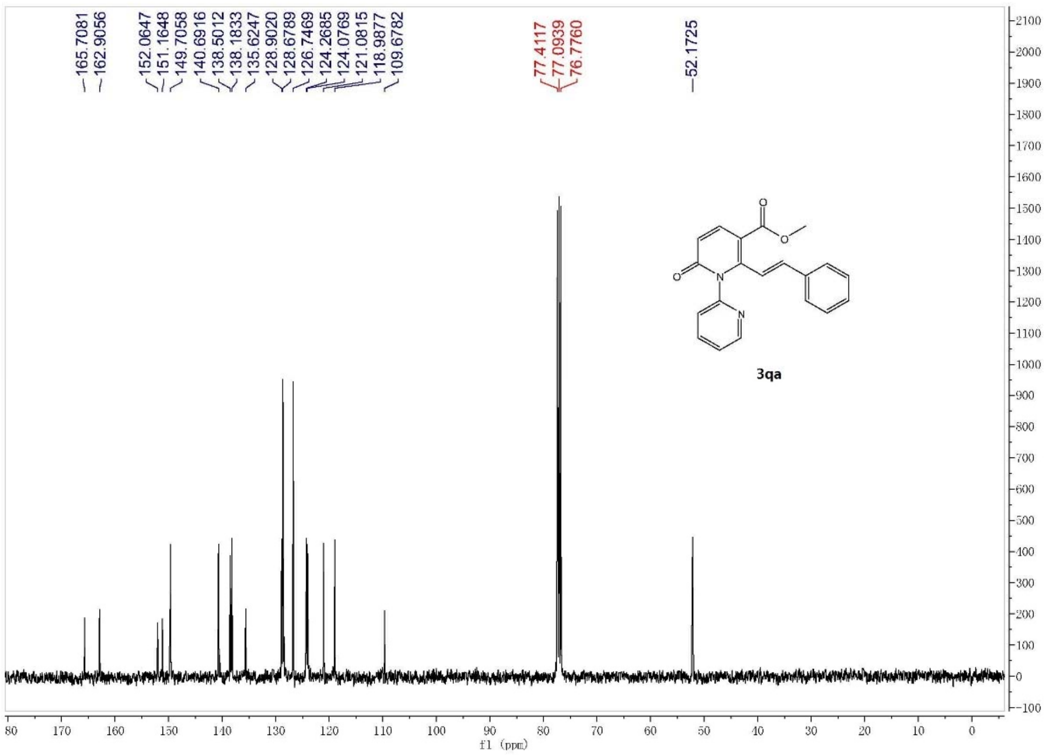

^{1}H and $^{13}C\{^{1}H\}$ NMR spectra of compound 3ra in $CDCl_3$

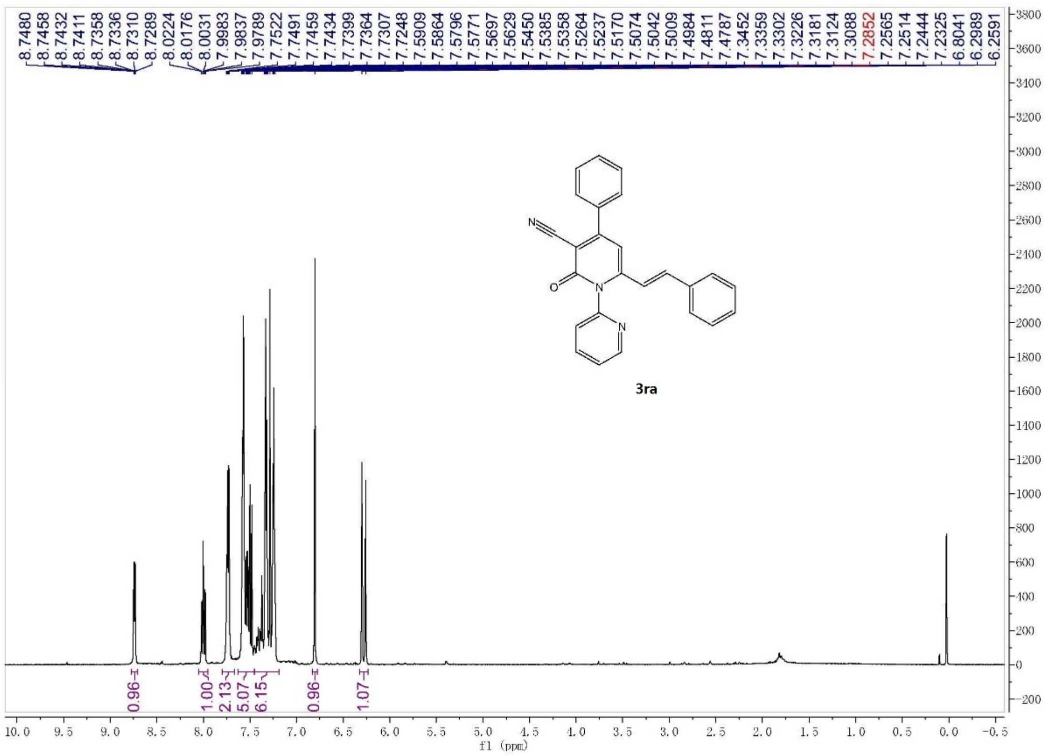

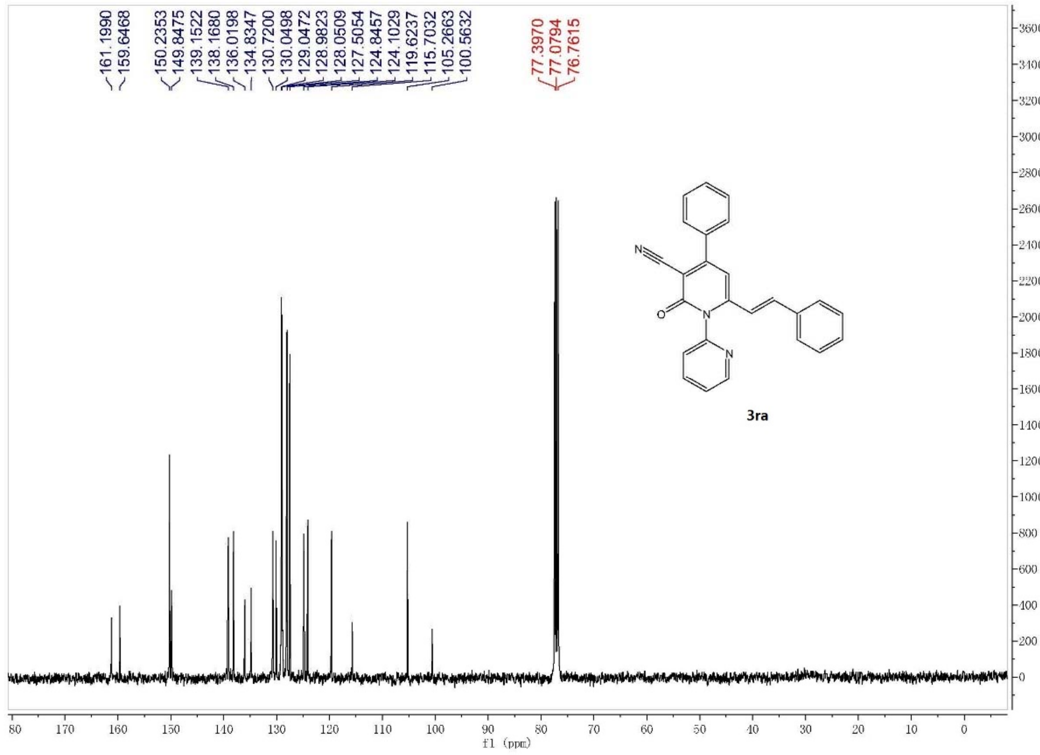

^{1}H and $^{13}C\{^{1}H\}$ NMR spectra of compound 3sa in $CDCl_3$

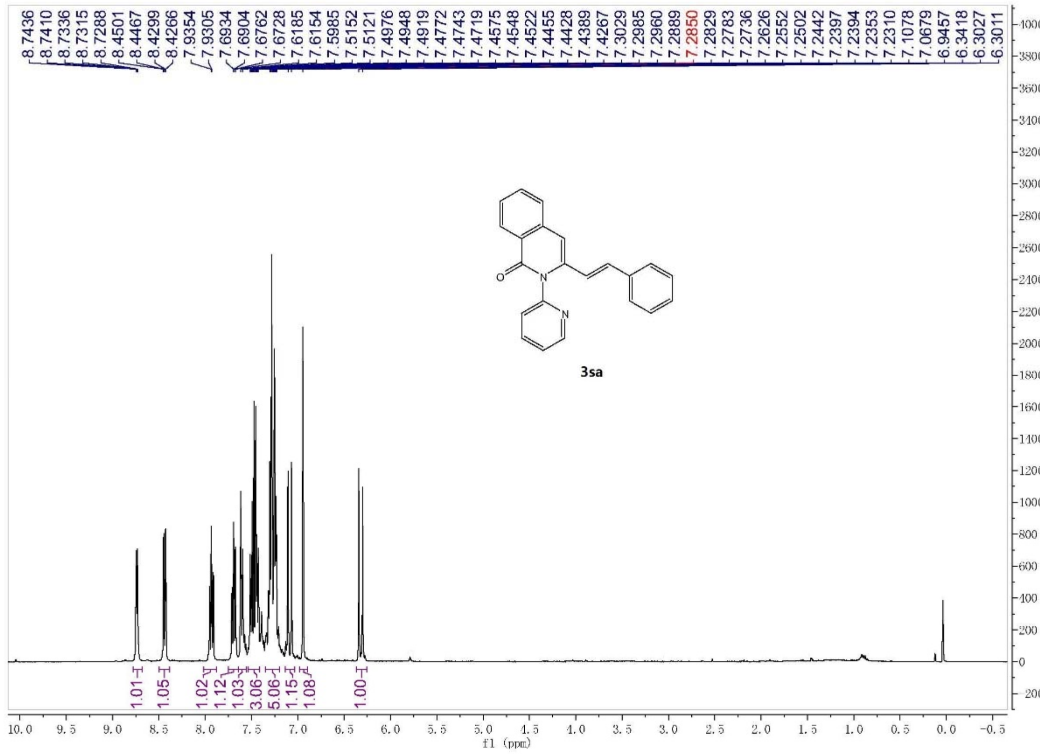

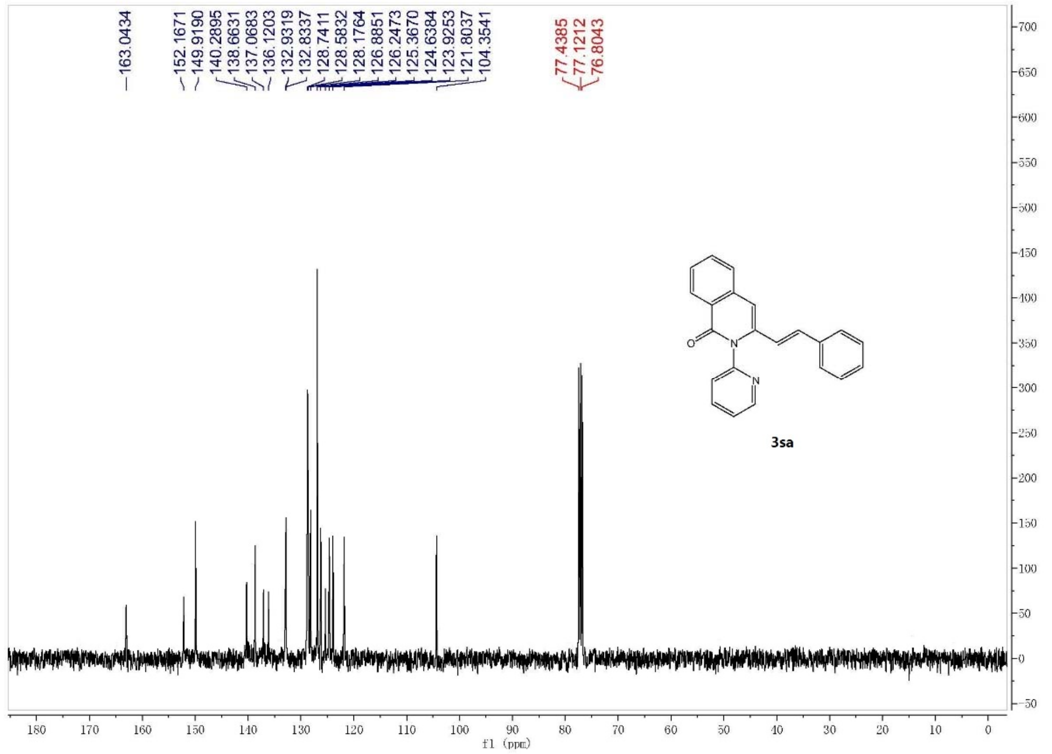

^{1}H and $^{13}C\{^{1}H\}$ NMR spectra of compound 3ta in $CDCl_3$

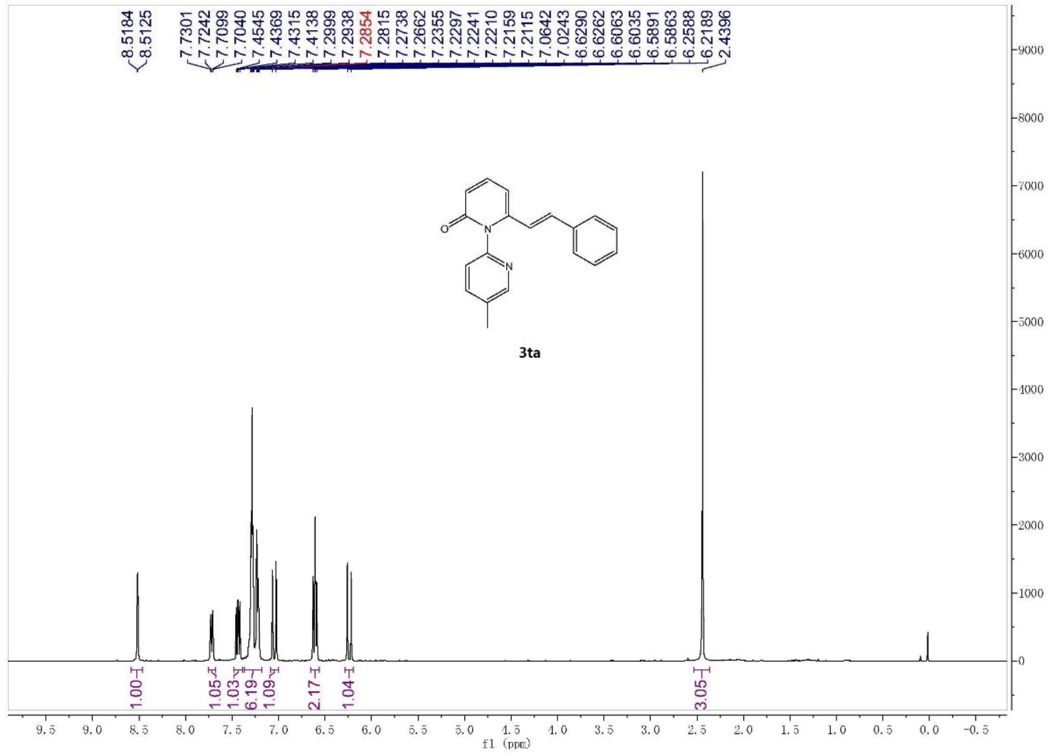

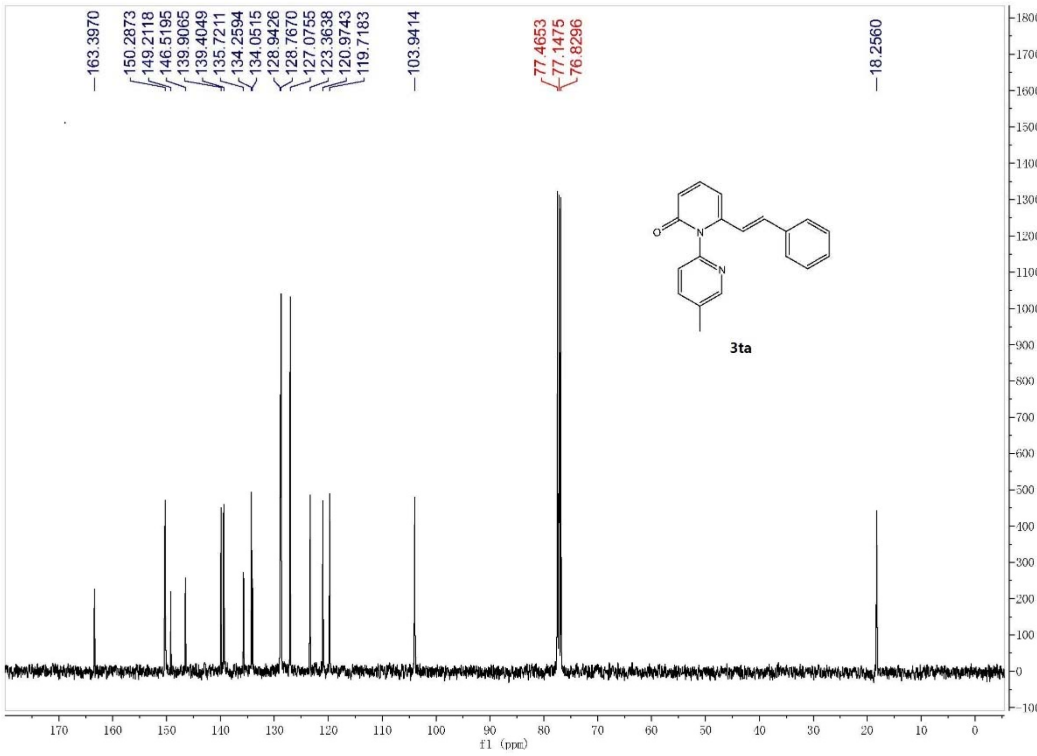

^{1}H and $^{13}C\{^{1}H\}$ NMR spectra of compound 3ua in $CDCl_3$

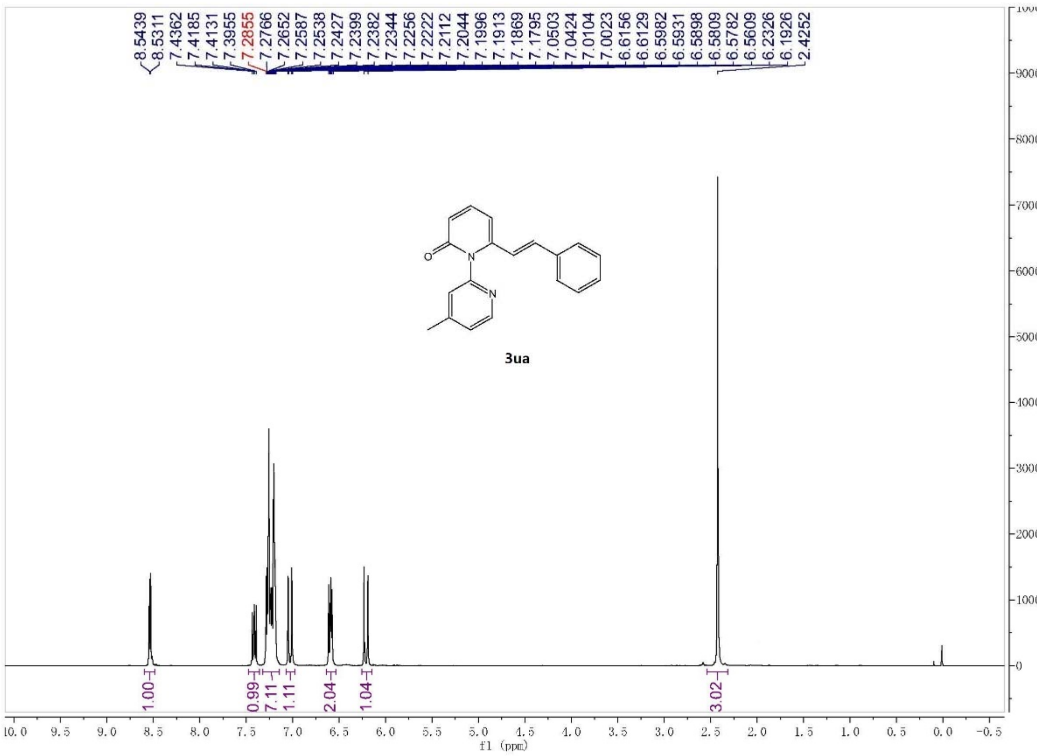

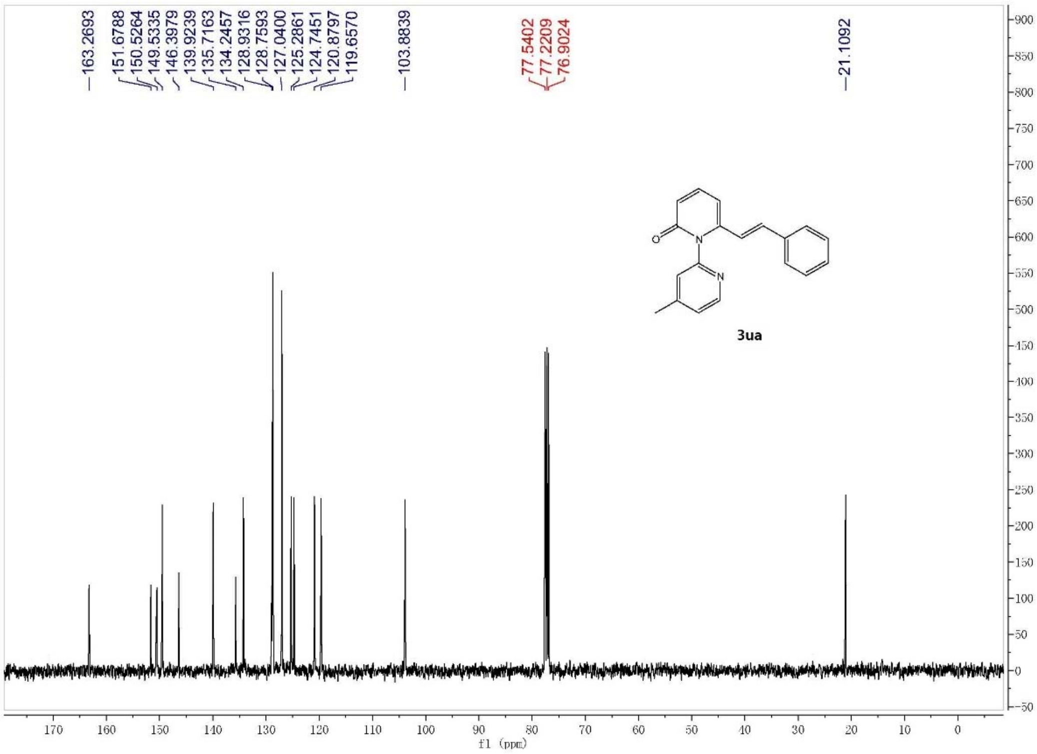

^1H and $^{13}C\{^1H\}$ NMR spectra of compound 3va in $CDCl_3$

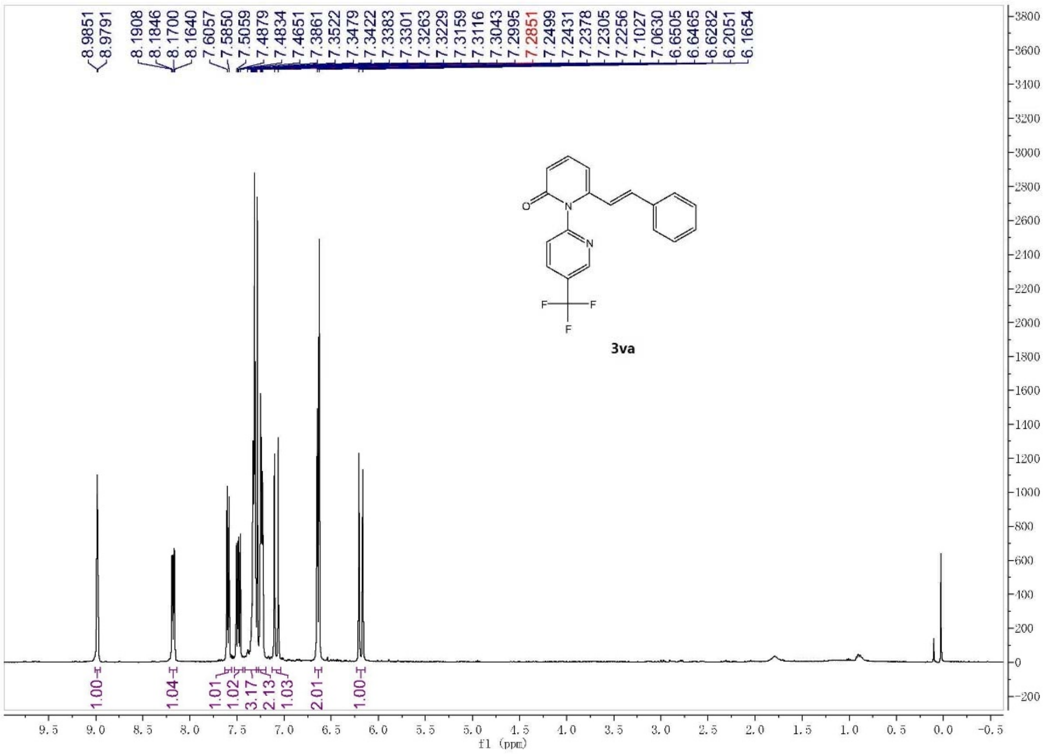

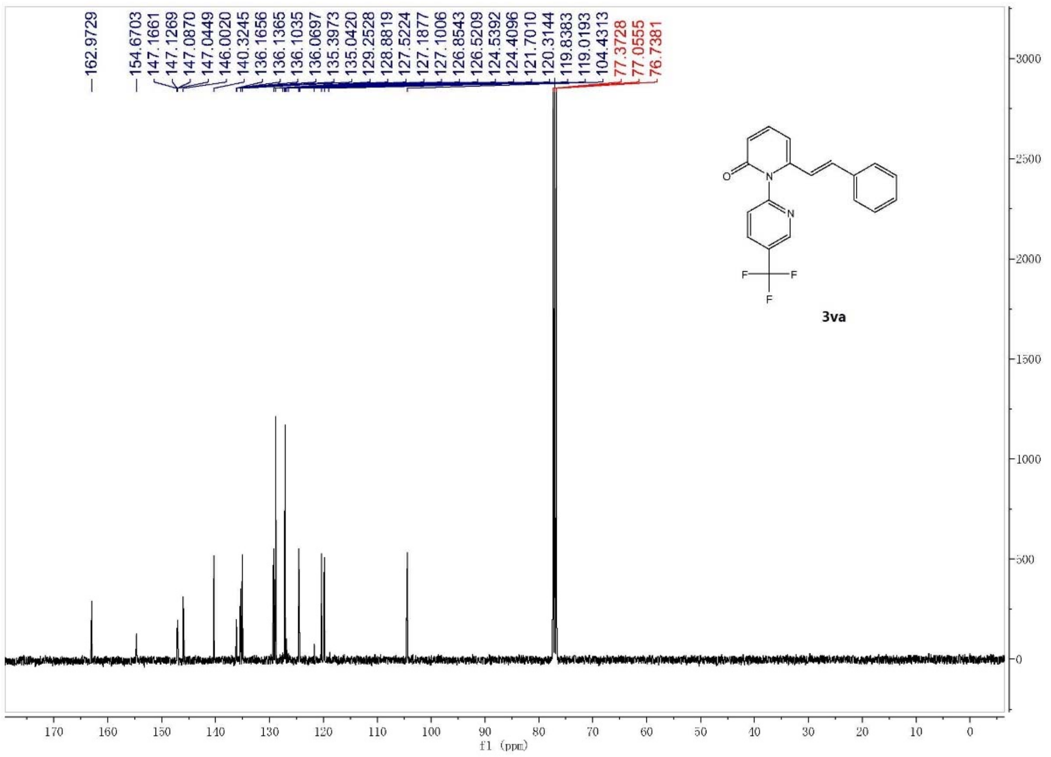

^1H and $^{13}C\{^1H\}$ NMR spectra of compound 3wa in $CDCl_3$

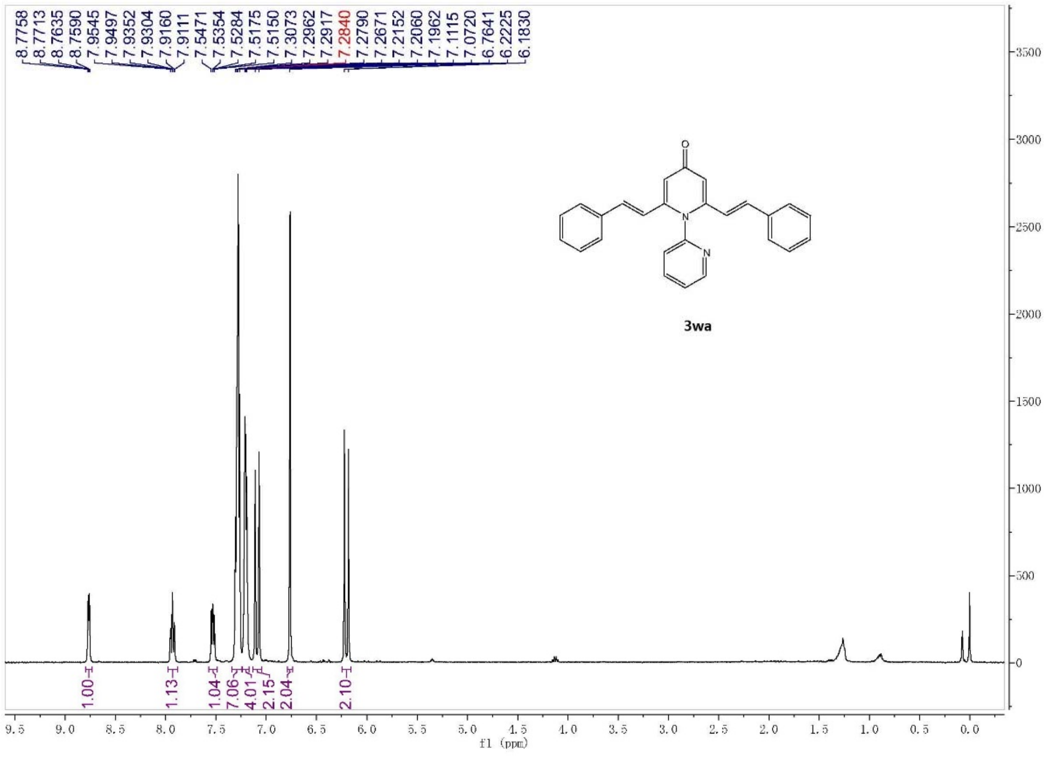

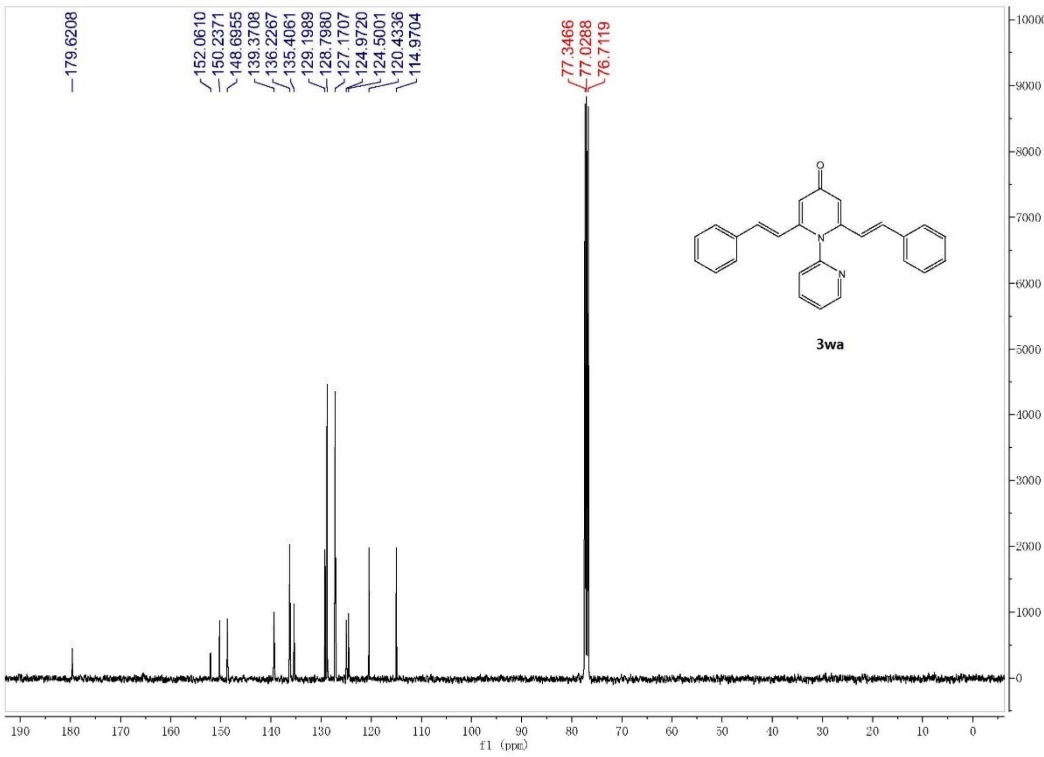

^1H and $^{13}C\{^1H\}$ NMR spectra of compound 3xa in $CDCl_3$

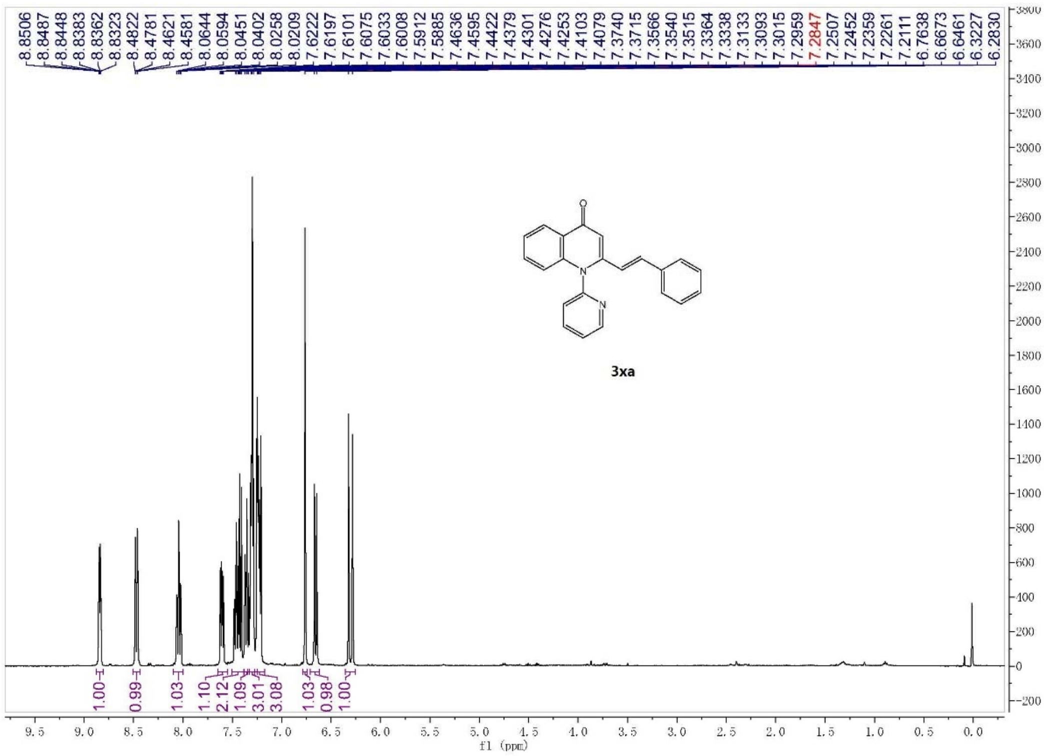

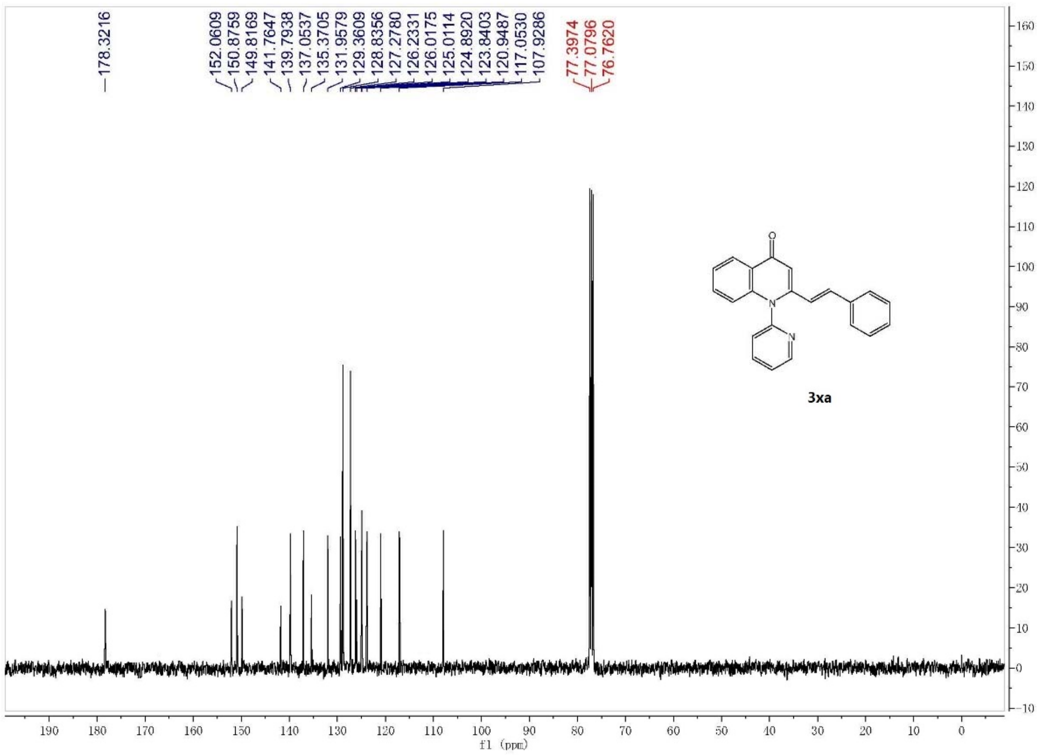

^{1}H and $^{13}C\{^{1}H\}$ NMR spectra of compound 5aa in $CDCl_3$

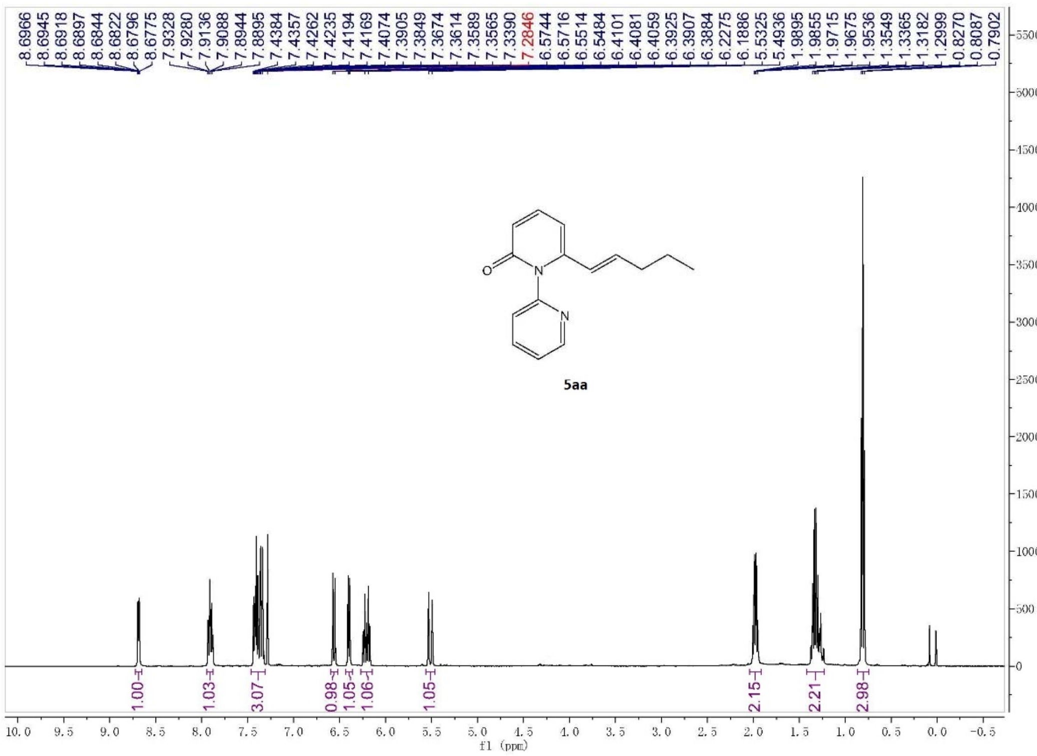

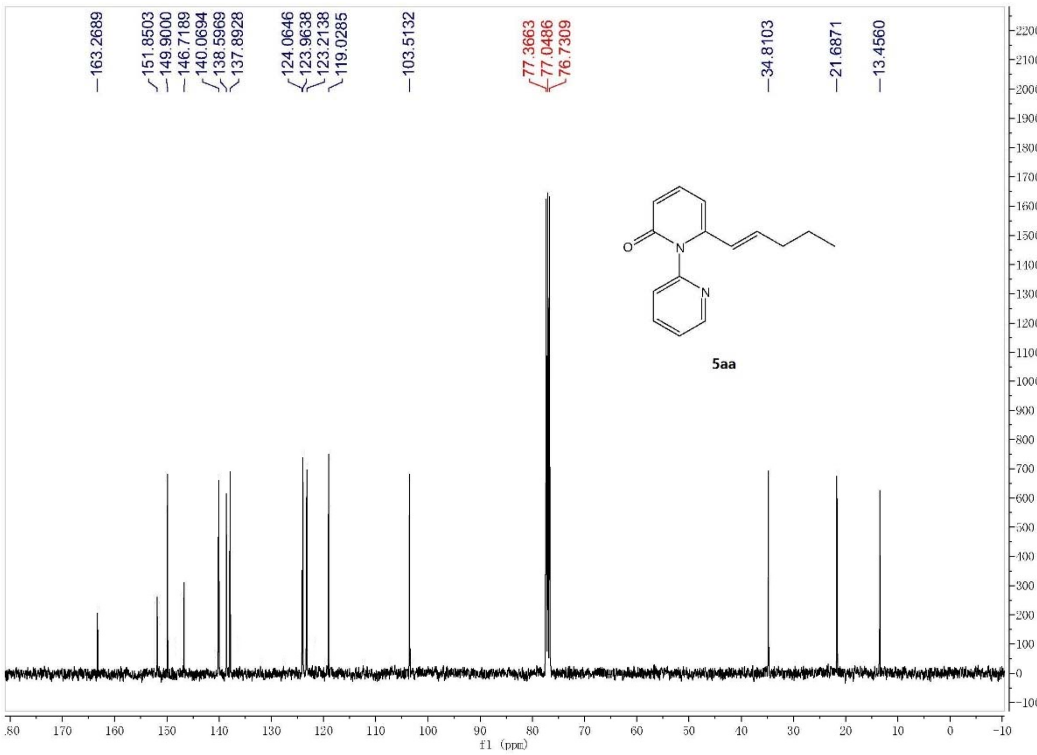

^{1}H and ^{13}C{^{1}H} NMR spectra of compound 5ab in $CDCl_3$

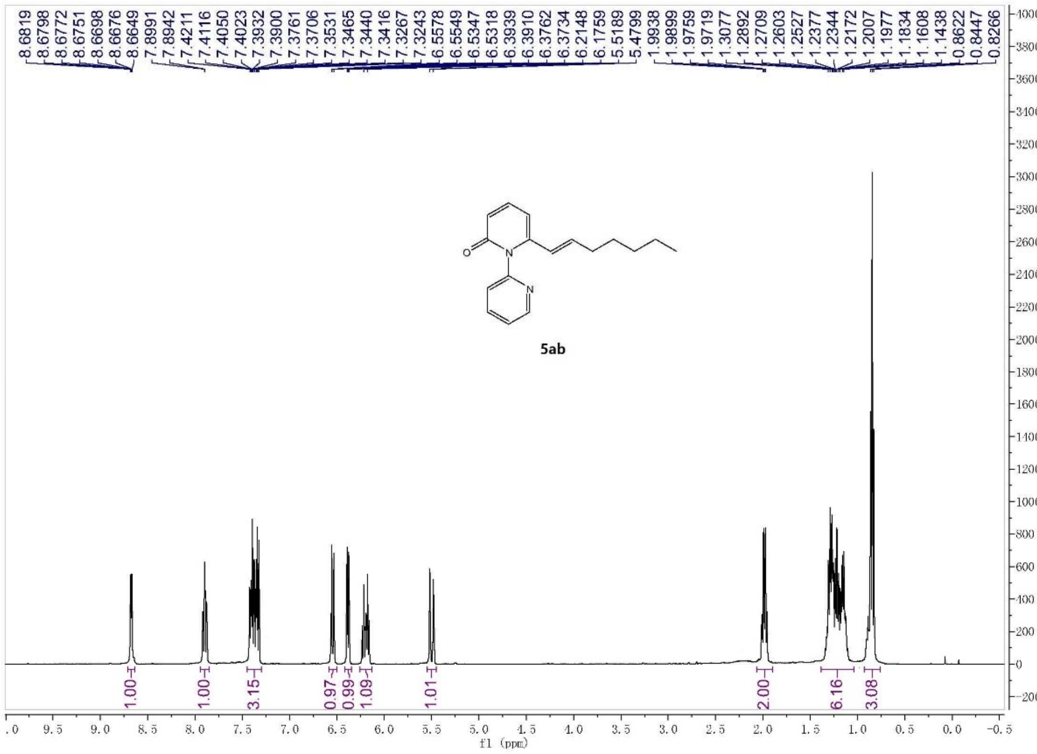

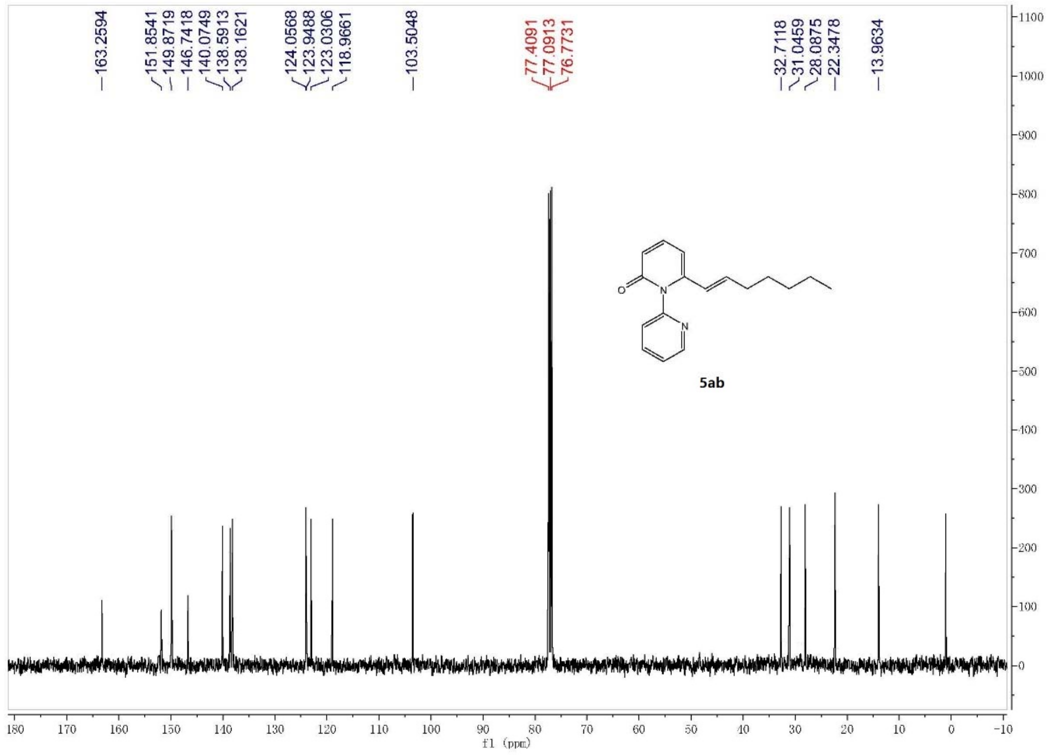

^{1}H and ^{13}C{^{1}H} NMR spectra of compound 5ac in $CDCl_3$

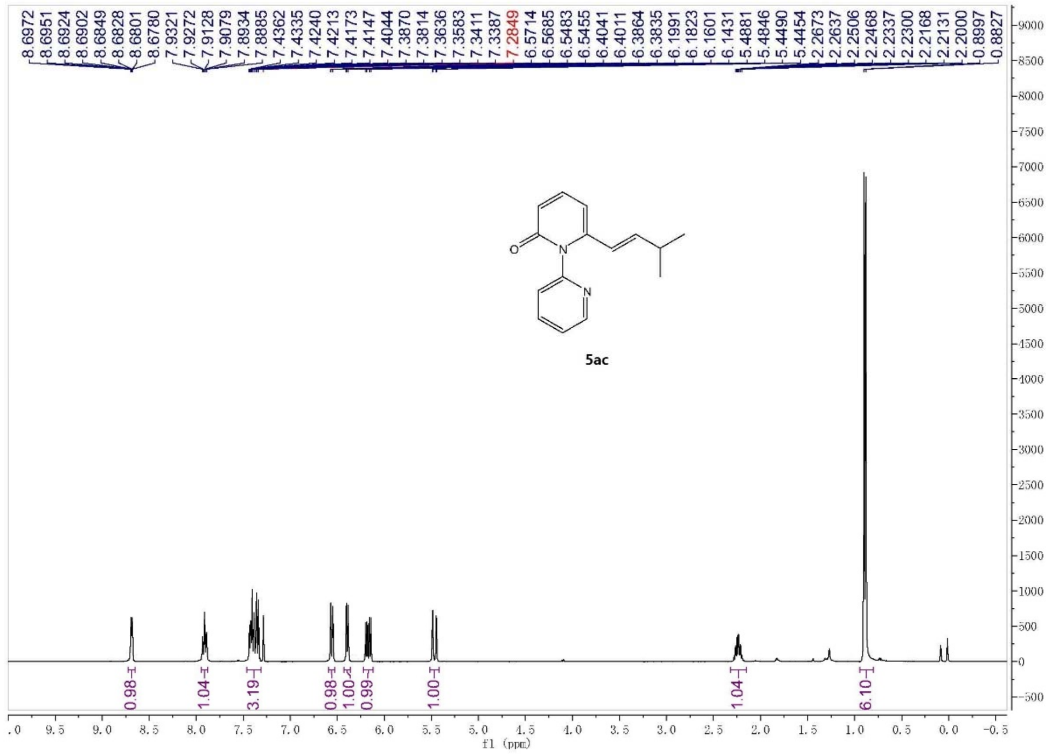

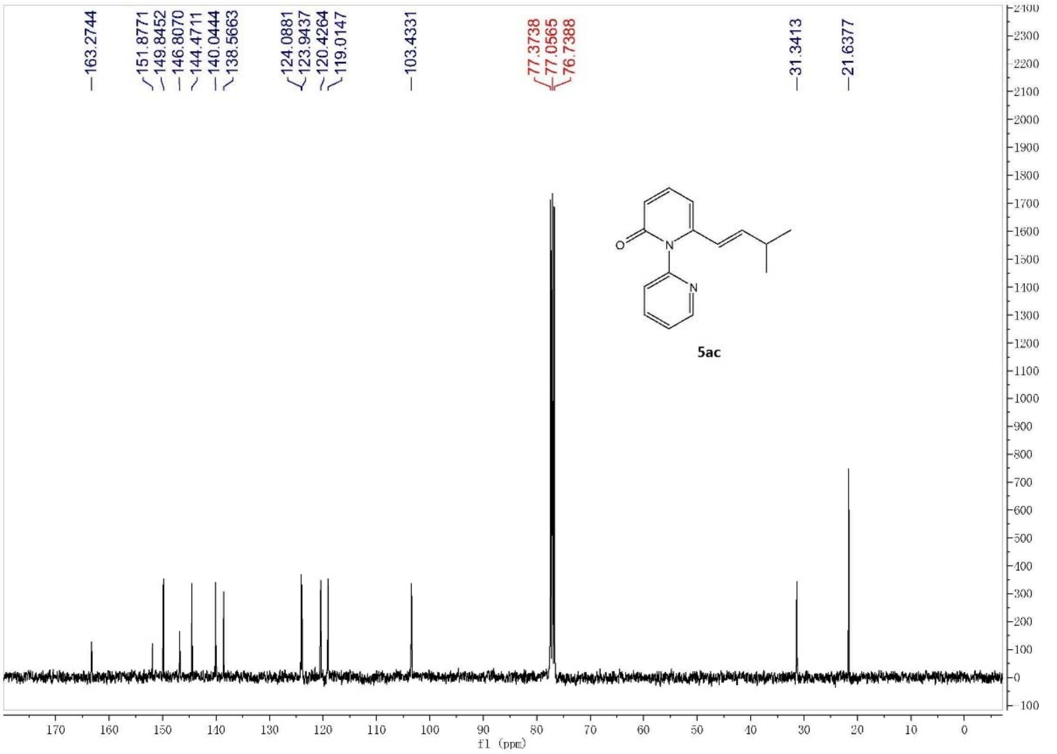

^{1}H and ^{13}C{^{1}H} NMR spectra of compound 5ad in $CDCl_3$

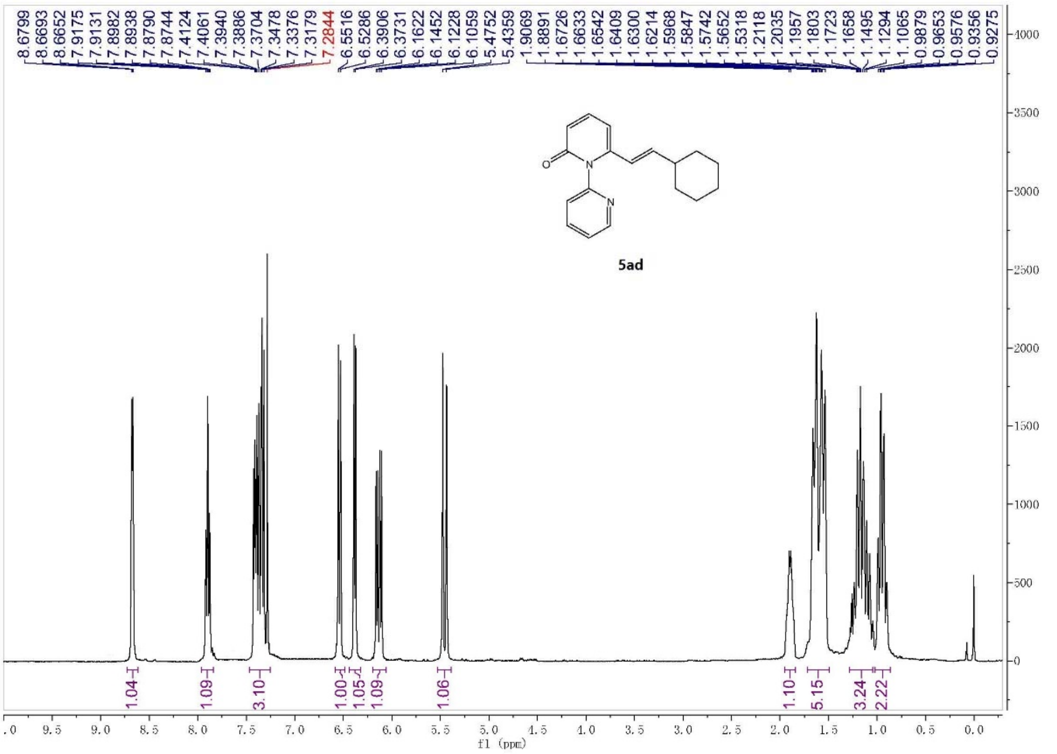

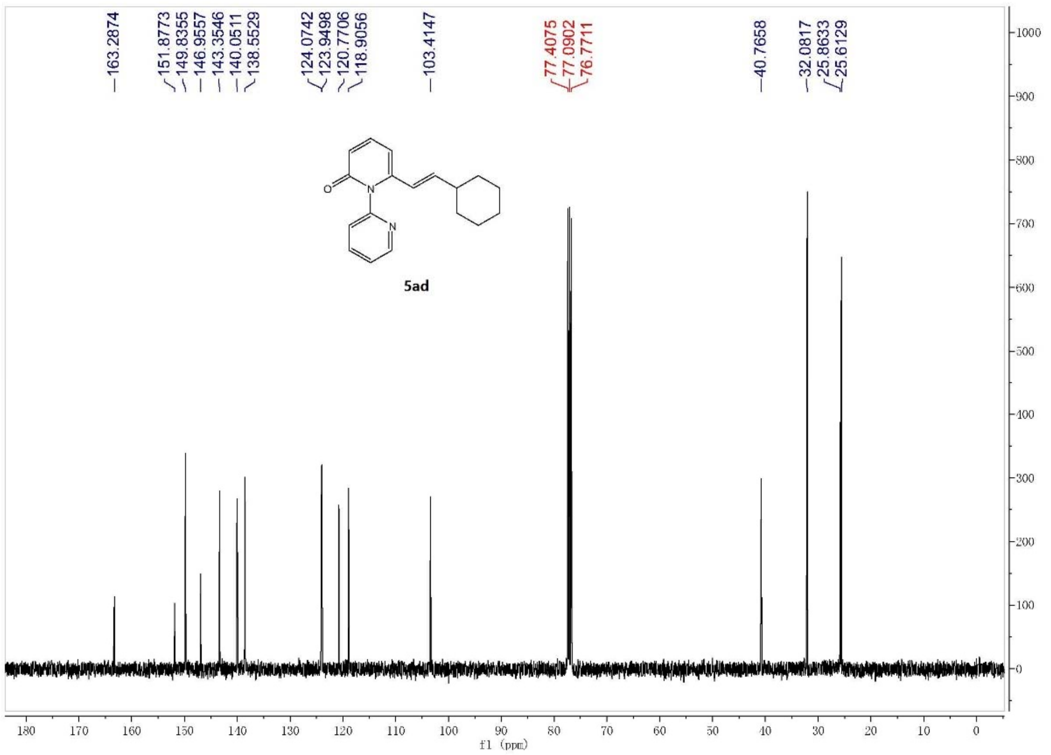

^{1}H and $^{13}C\{^{1}H\}$ NMR spectra of compound 5ae in $CDCl_3$

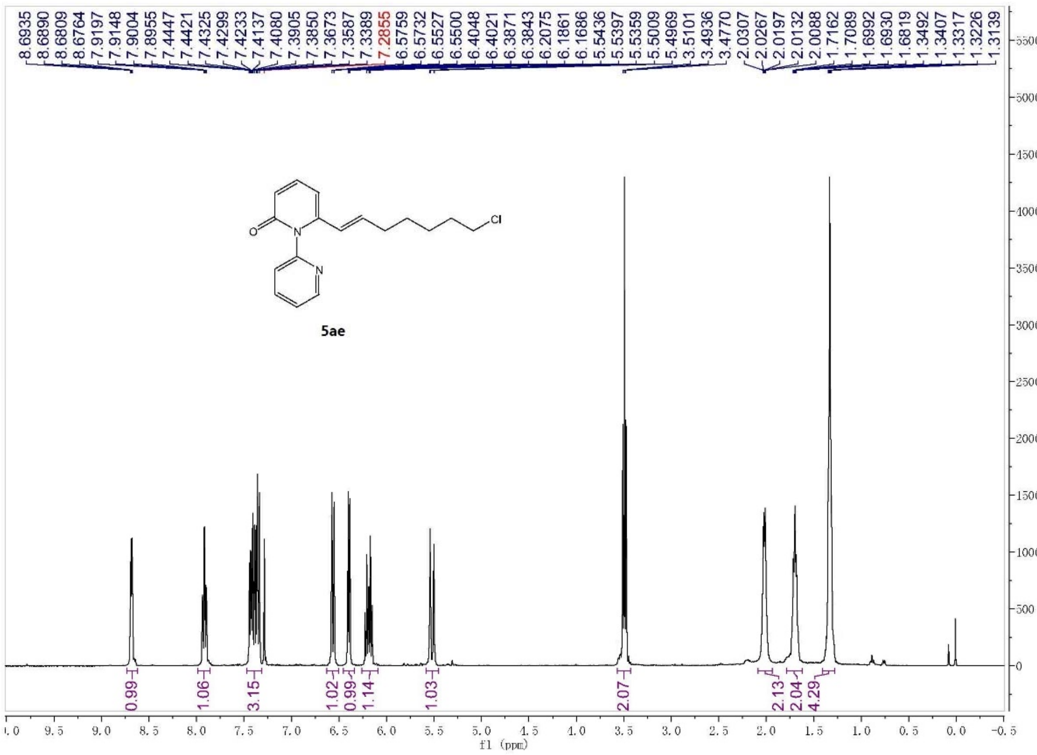

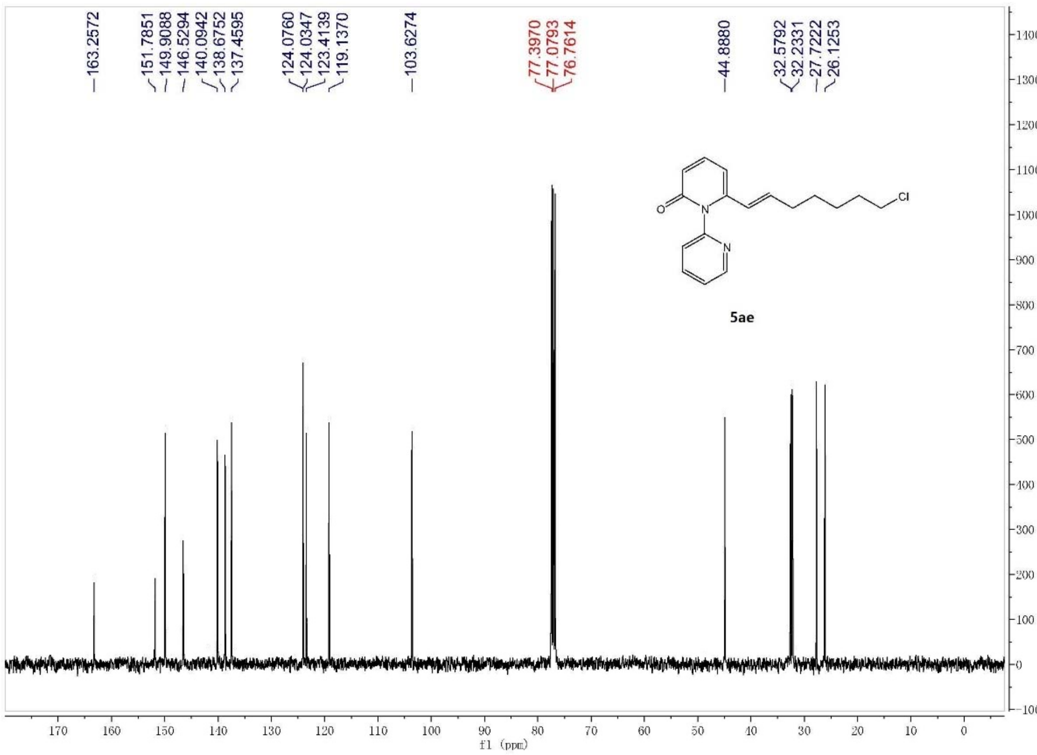

^{1}H and ^{13}C{^{1}H} NMR spectra of compound 5af in $CDCl_3$

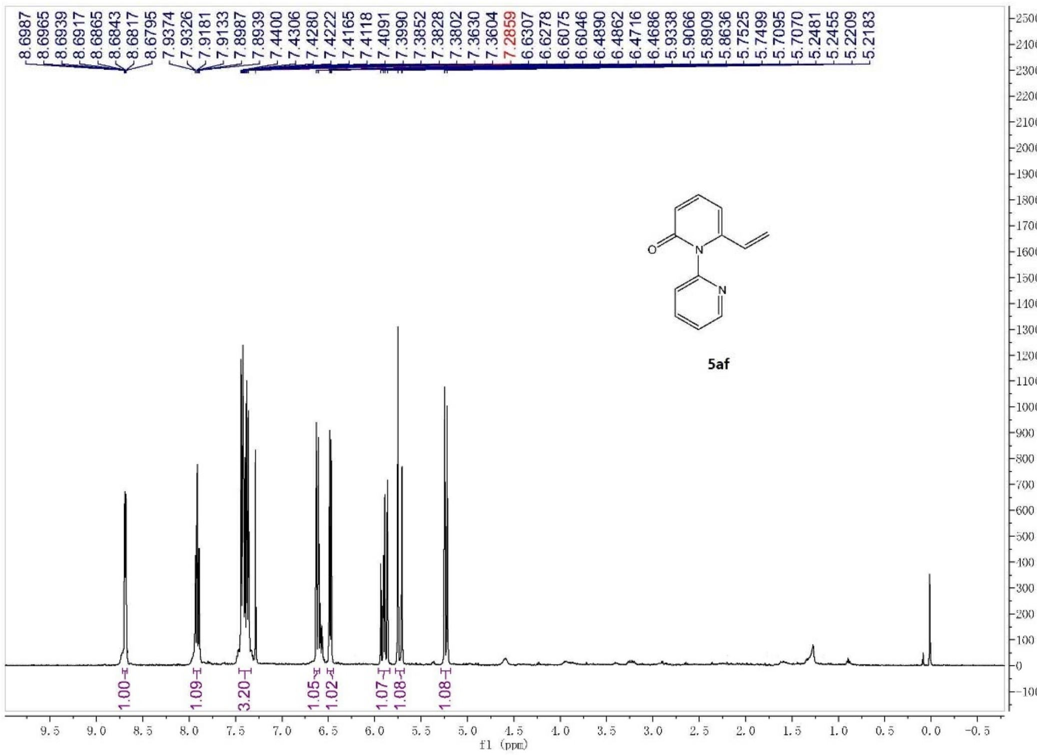

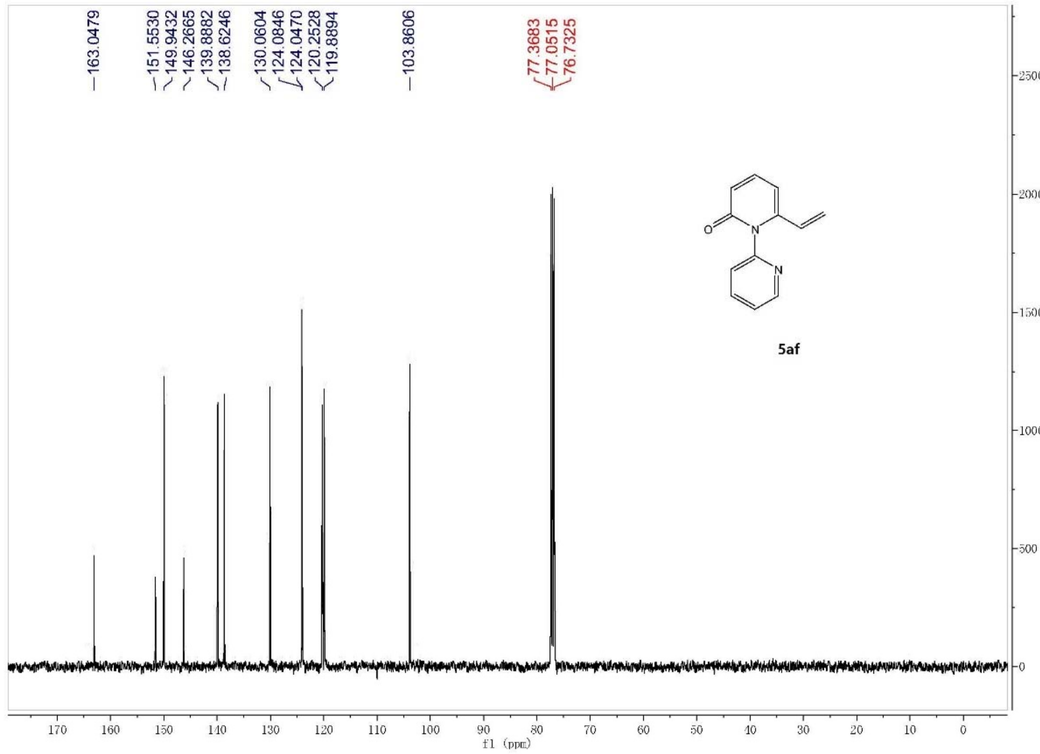

^1H and $^{13}C\{^1H\}$ NMR spectra of compound 5ag in $CDCl_3$

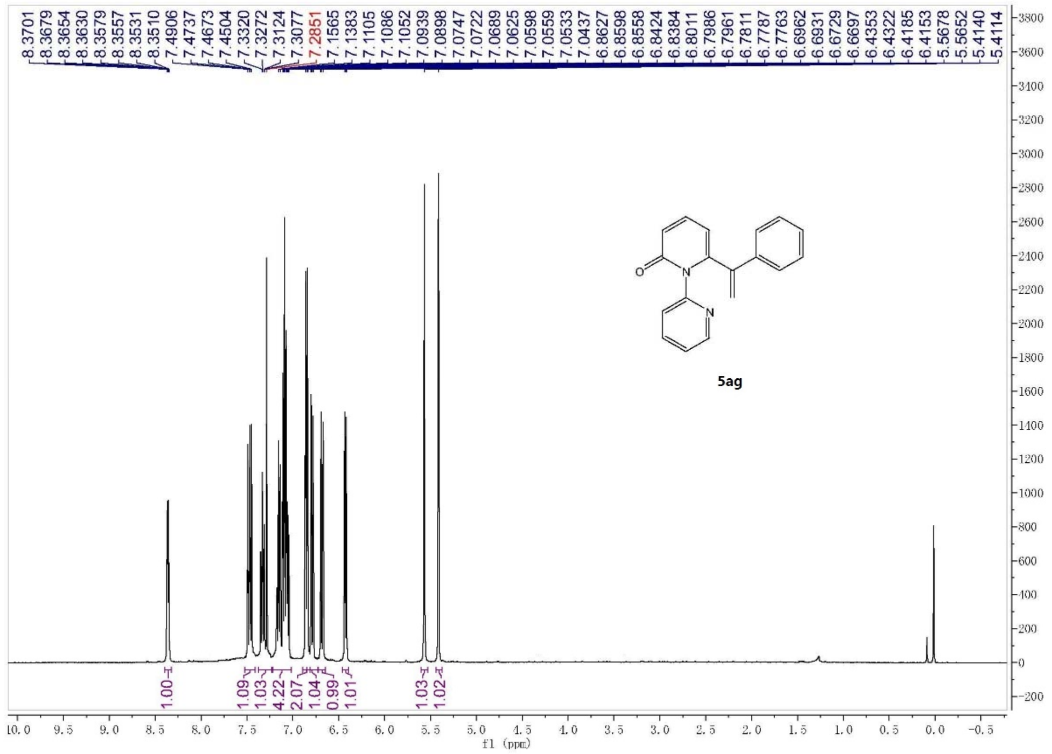

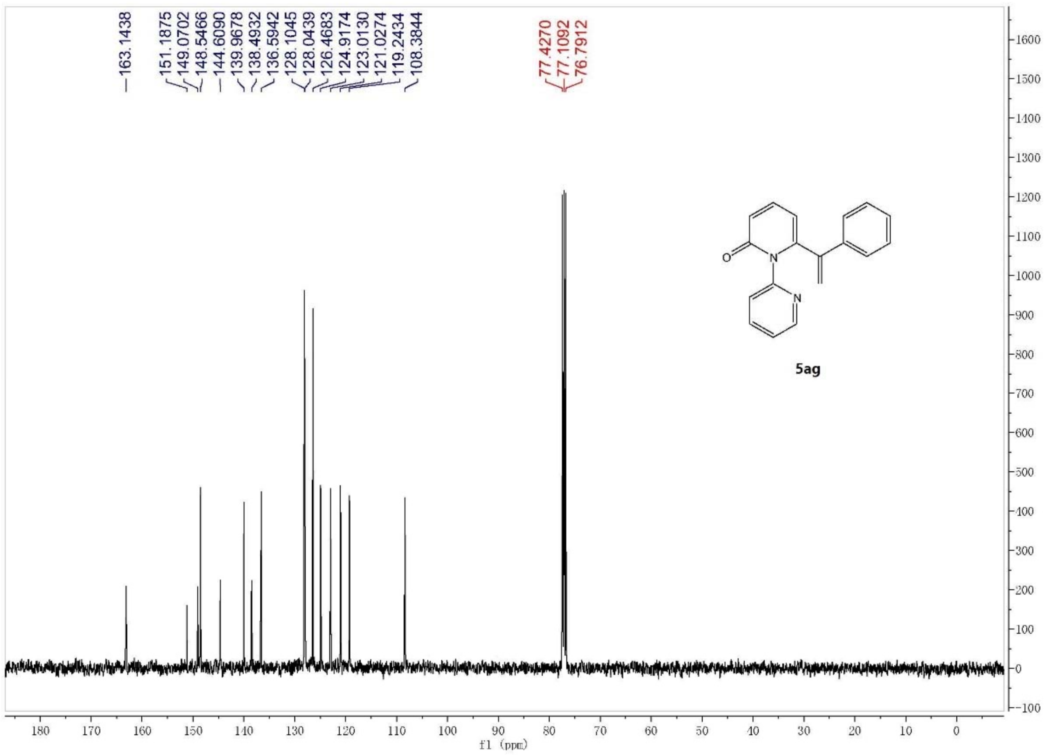

^{1}H and ^{13}C{^{1}H} NMR spectra of compound 5ah in $CDCl_3$

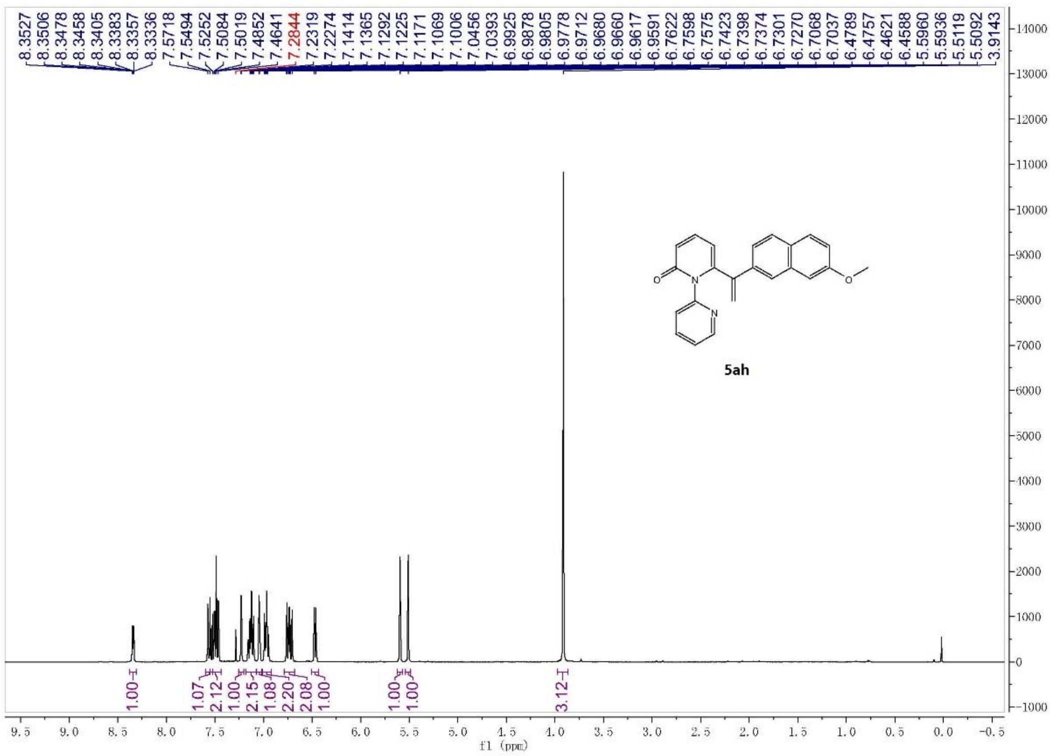

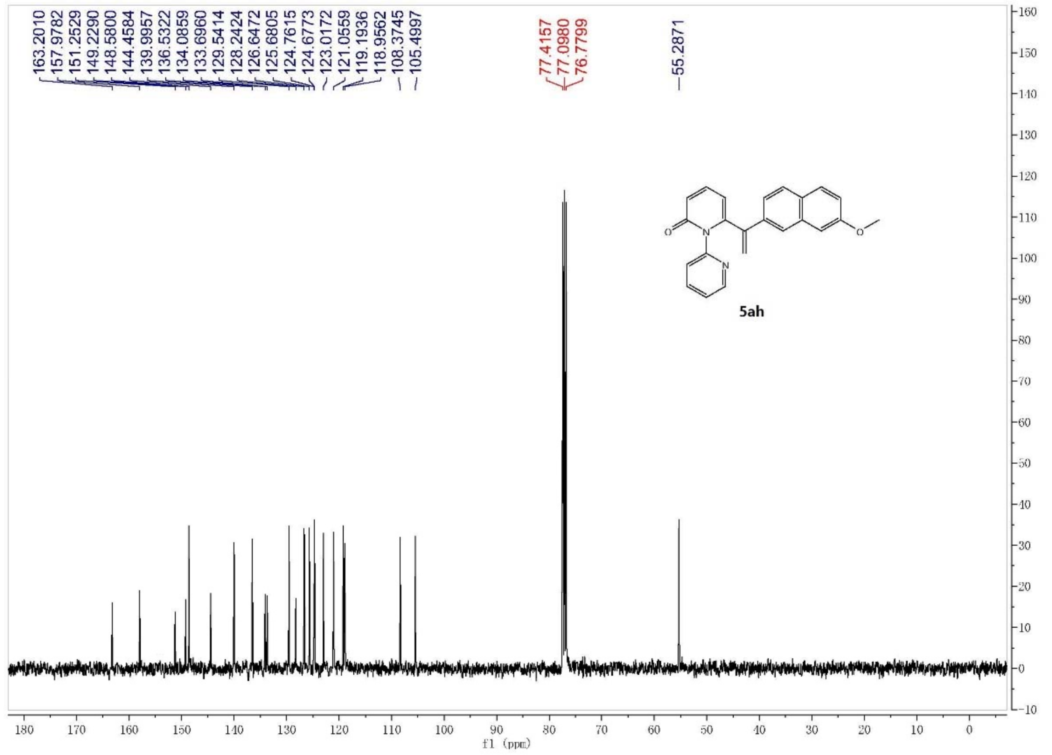

^1H and $^{13}C\{^1H\}$ NMR spectra of compound 5ai in $CDCl_3$

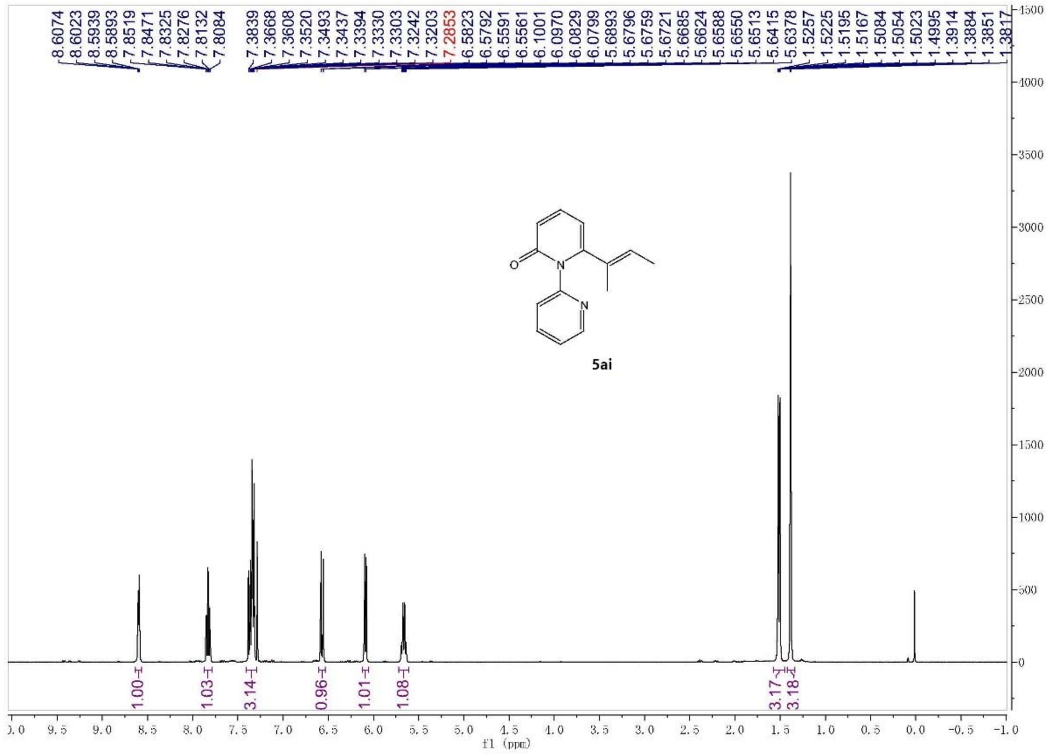

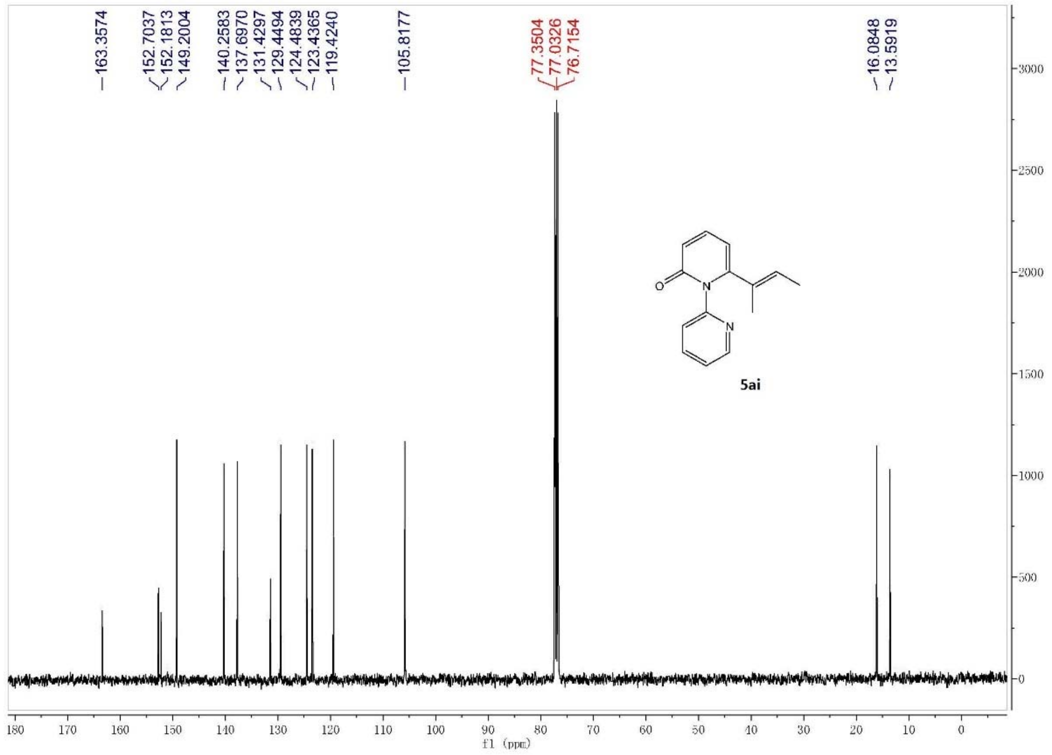

^{1}H and ^{13}C{^{1}H} NMR spectra of compound 5aj in $CDCl_3$

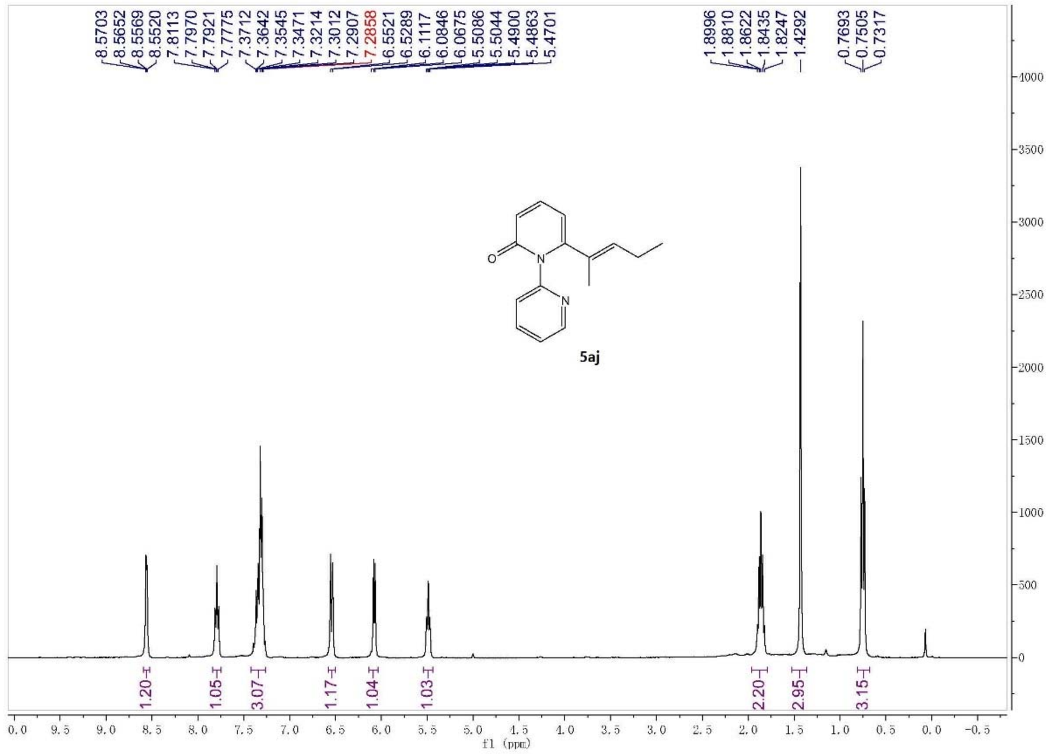

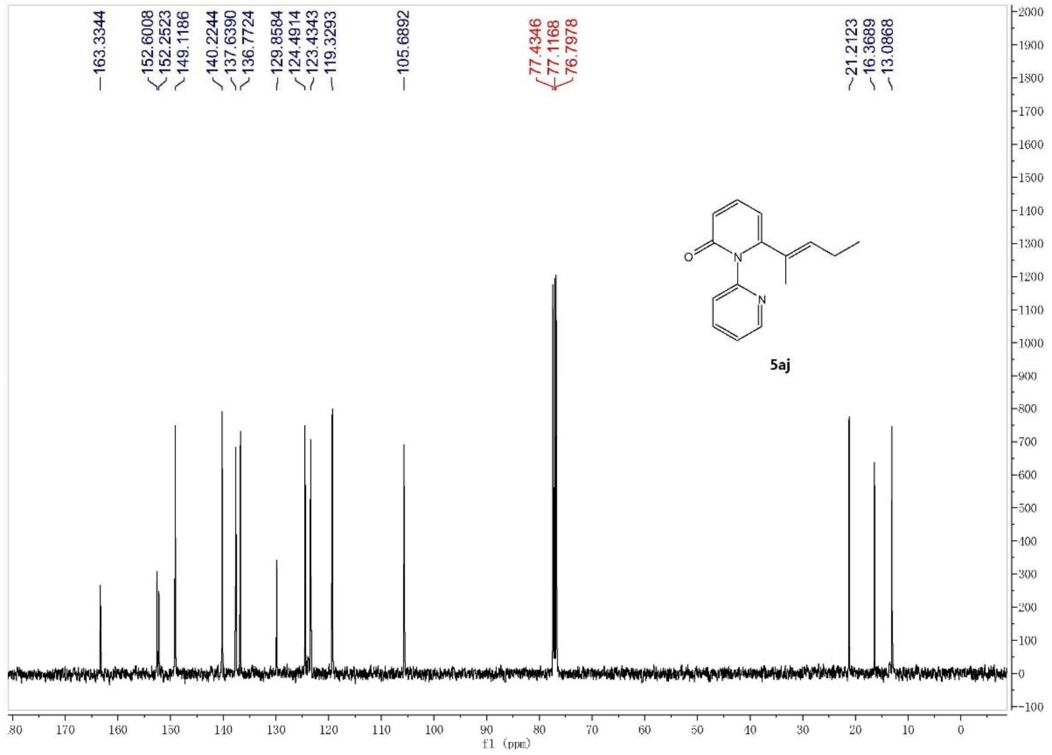

^{1}H and $^{13}C\{^{1}H\}$ NMR spectra of compound 5ak in $CDCl_3$

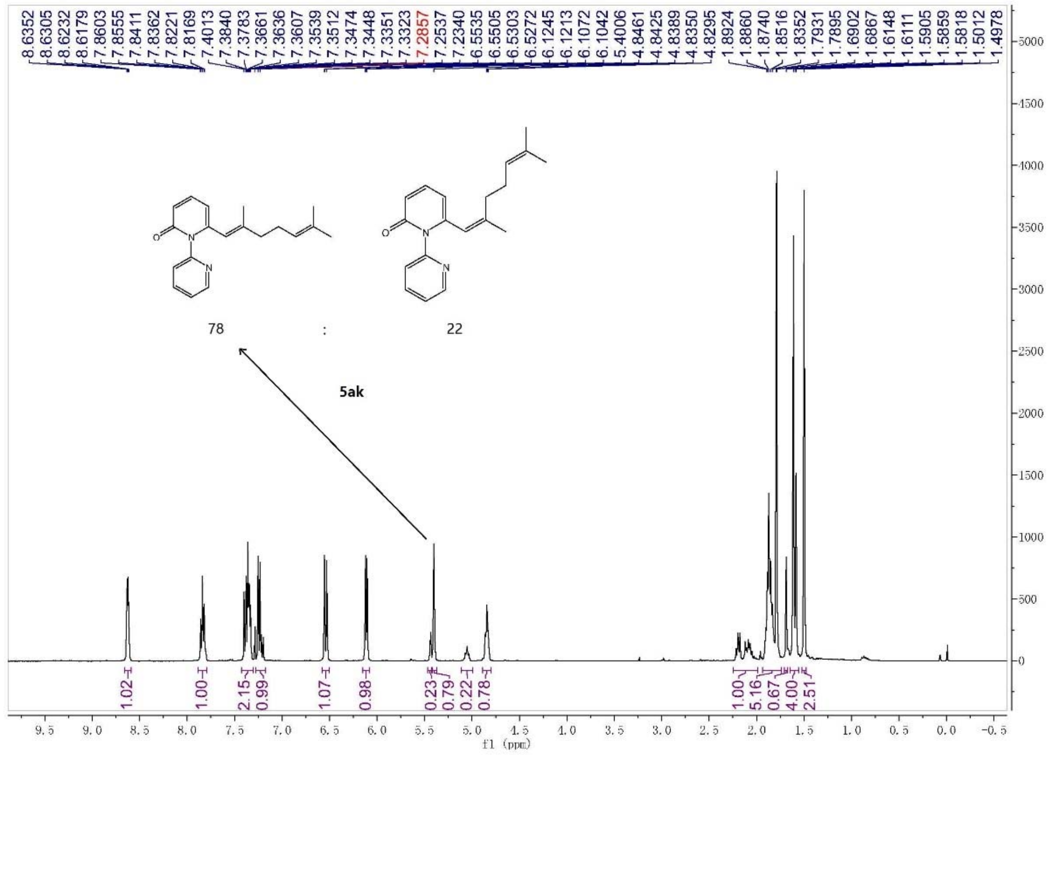

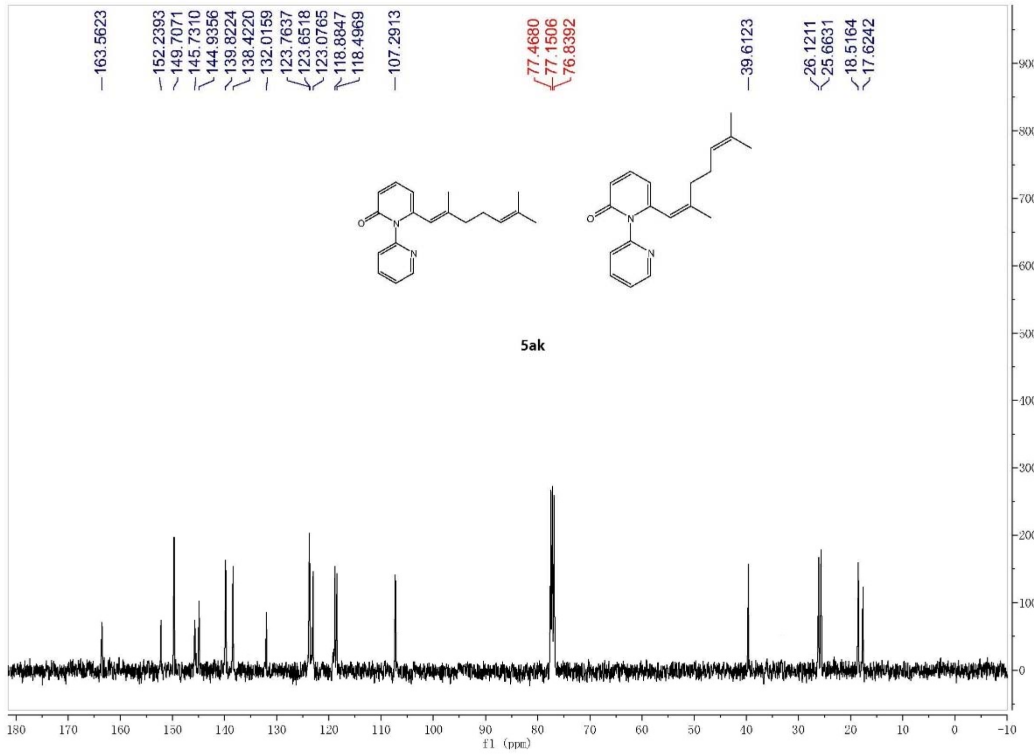

^{1}H and $^{13}C\{^{1}H\}$ NMR spectra of compound 5al in $CDCl_3$

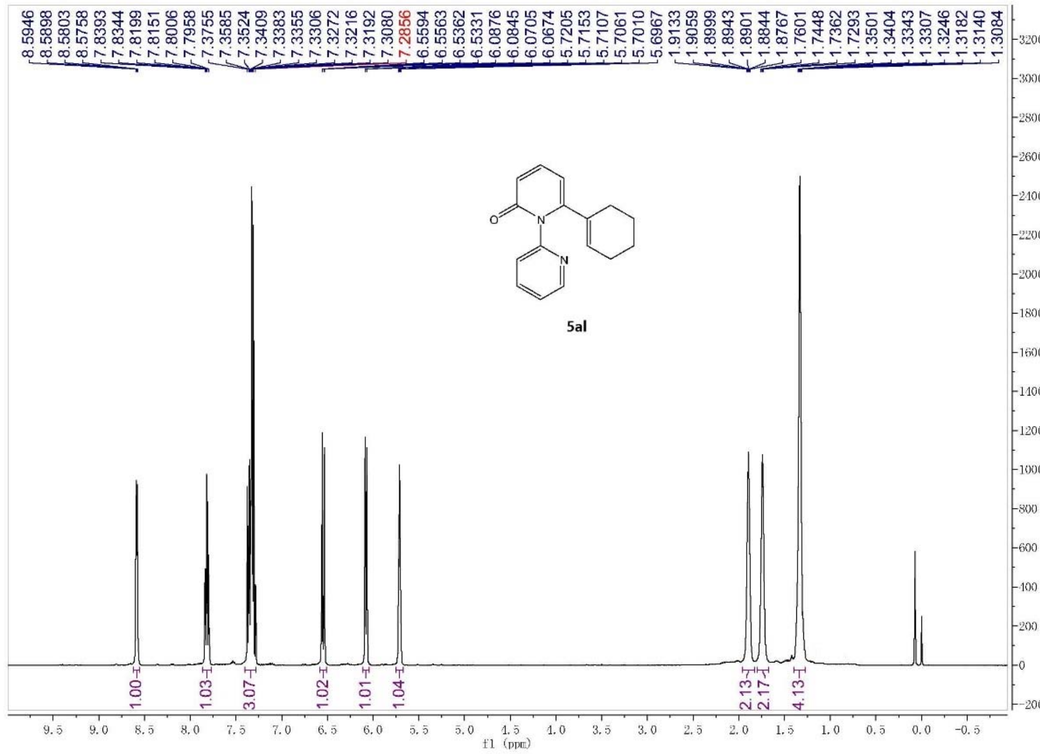

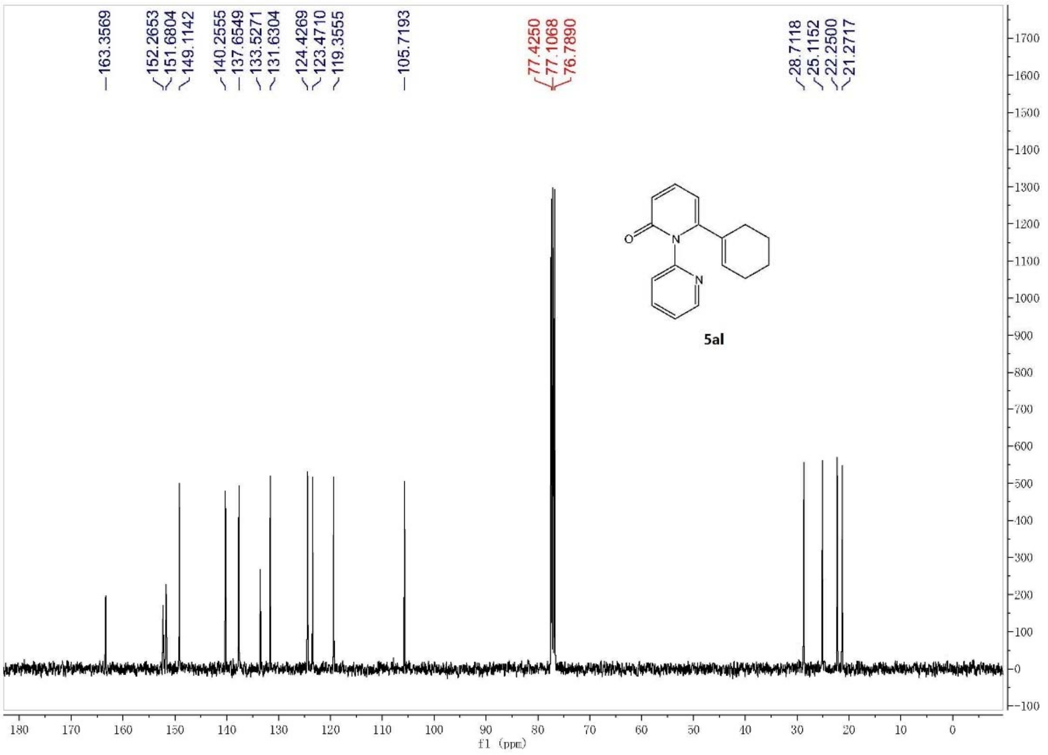

^1H and $^{13}C\{^1H\}$ NMR spectra of compound 5am in CD_3OD_3

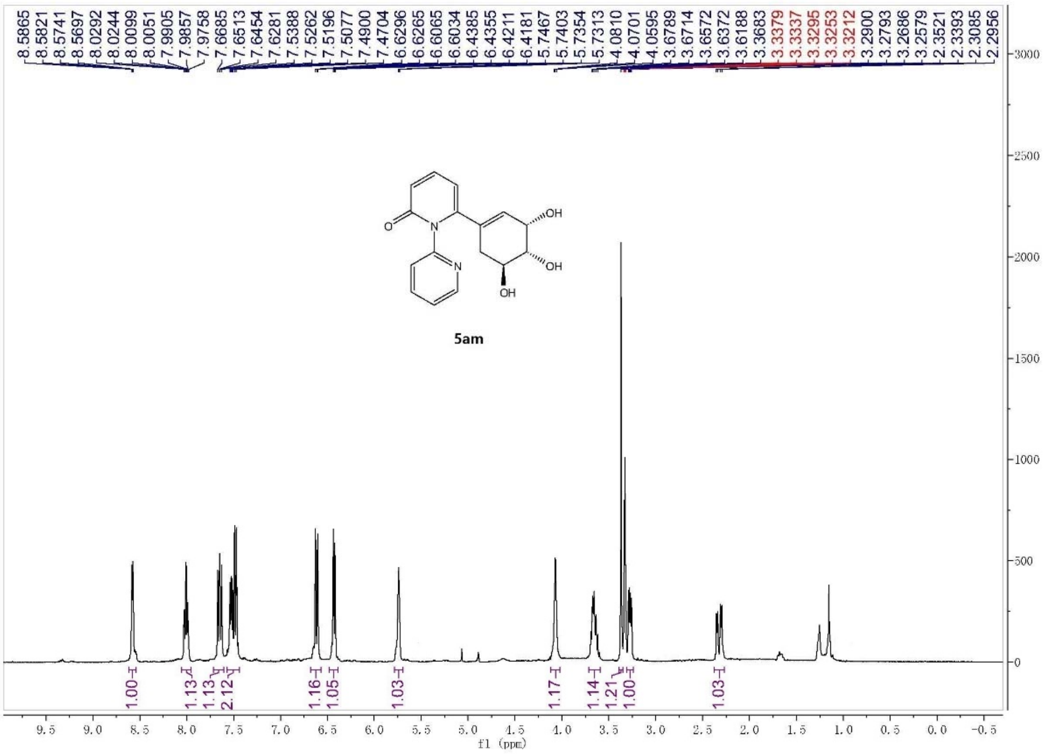

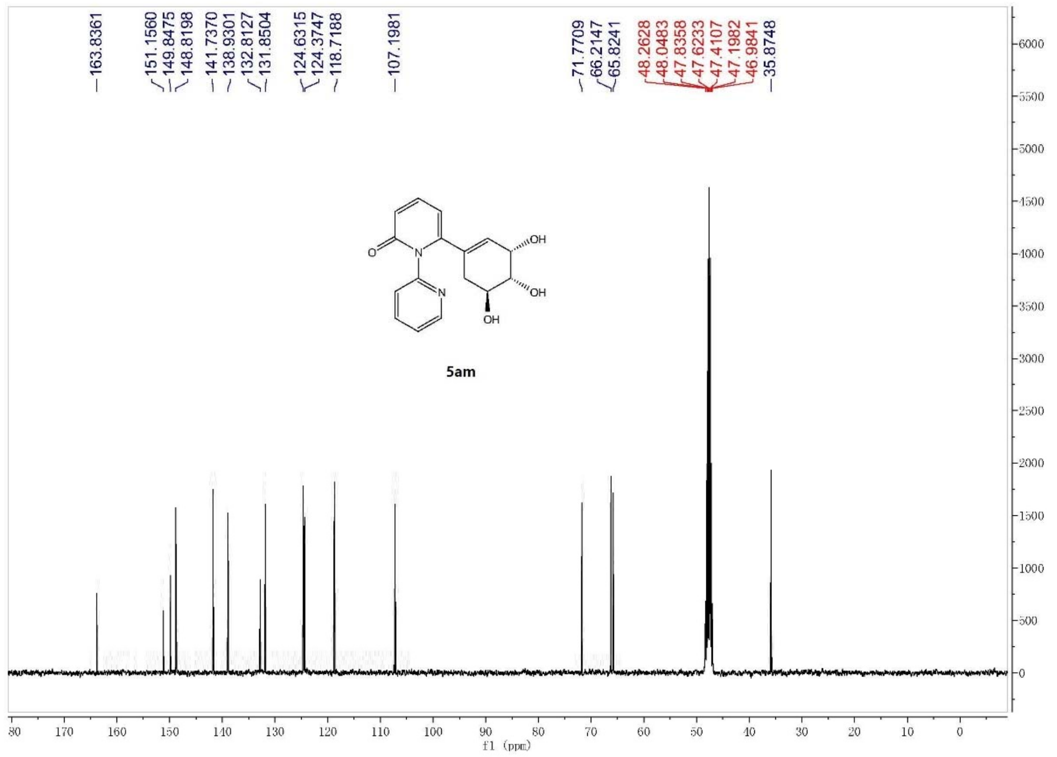

^1H and $^{13}C\{^1H\}$ NMR spectra of compound 5an in $CDCl_3$

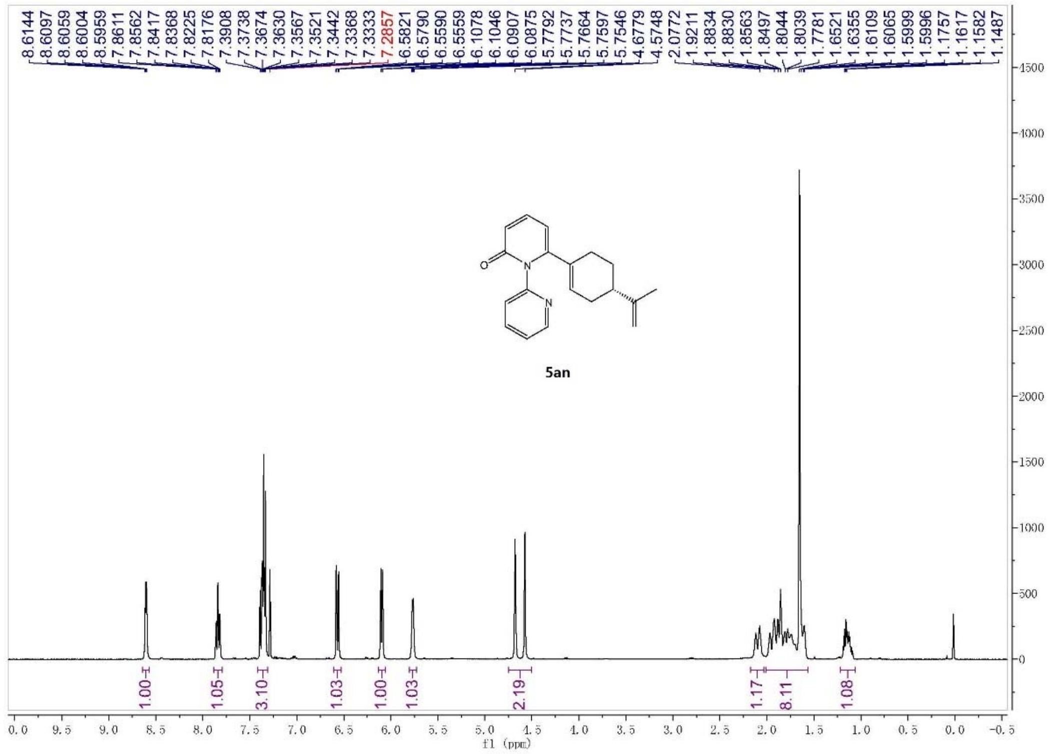

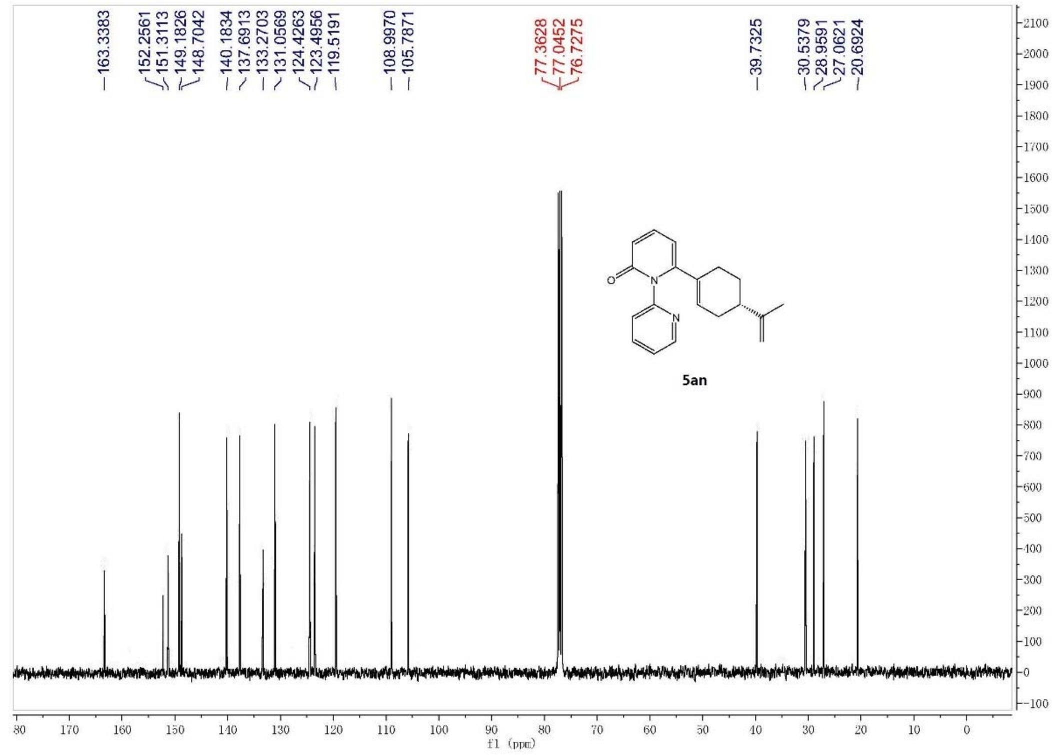

^{1}H and $^{13}C\{^{1}H\}$ NMR spectra of compound 5ao in $CDCl_3$

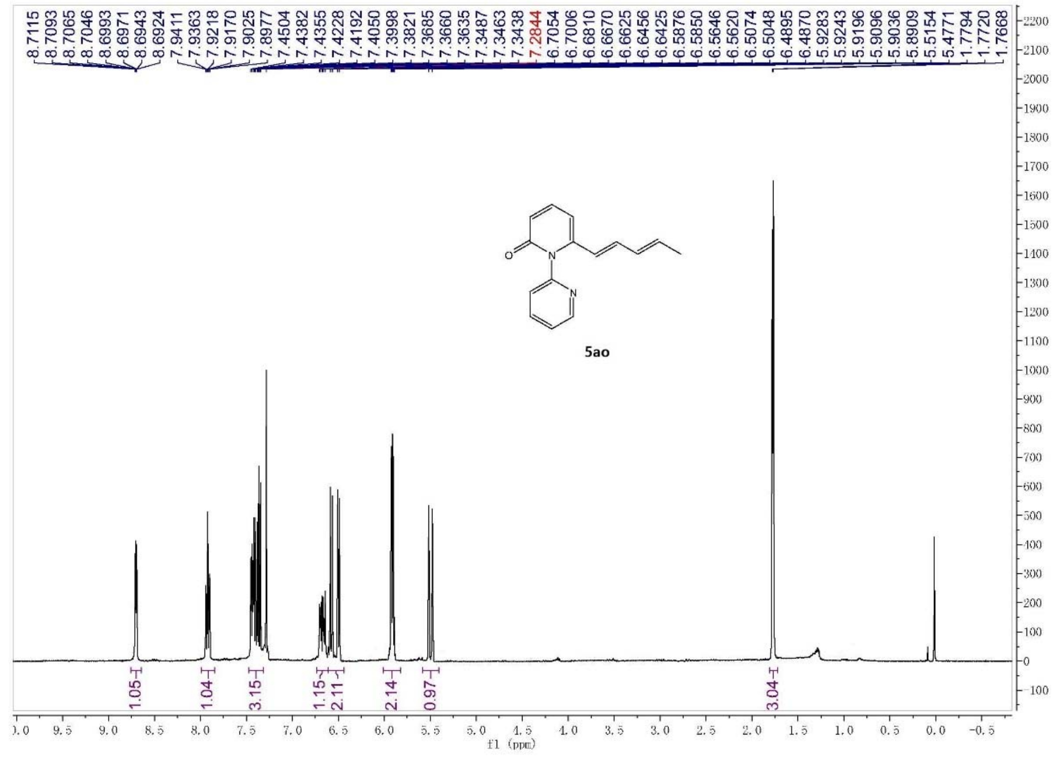

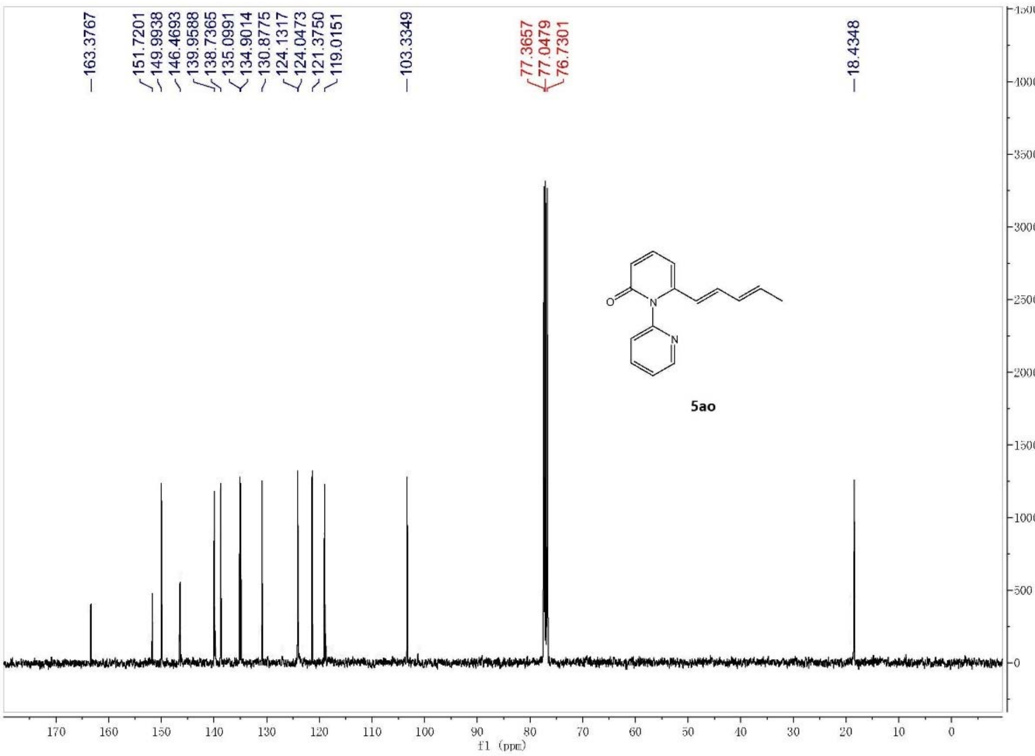

^{1}H and ^{13}C{^{1}H} NMR spectra of compound 5ap in $CDCl_3$

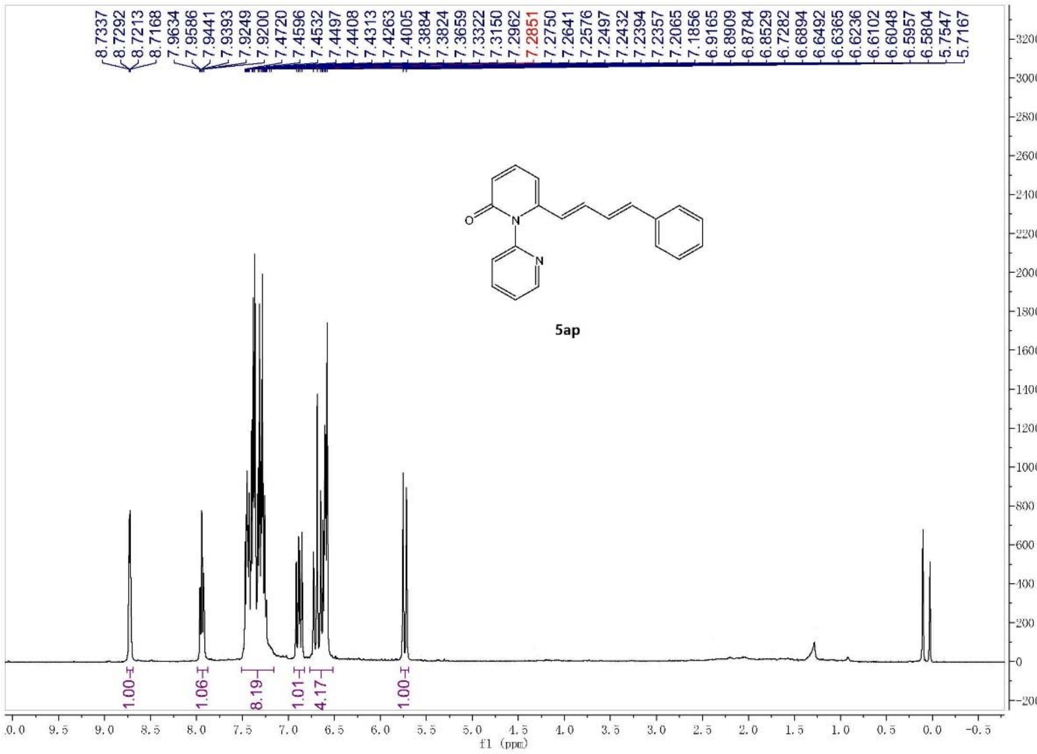

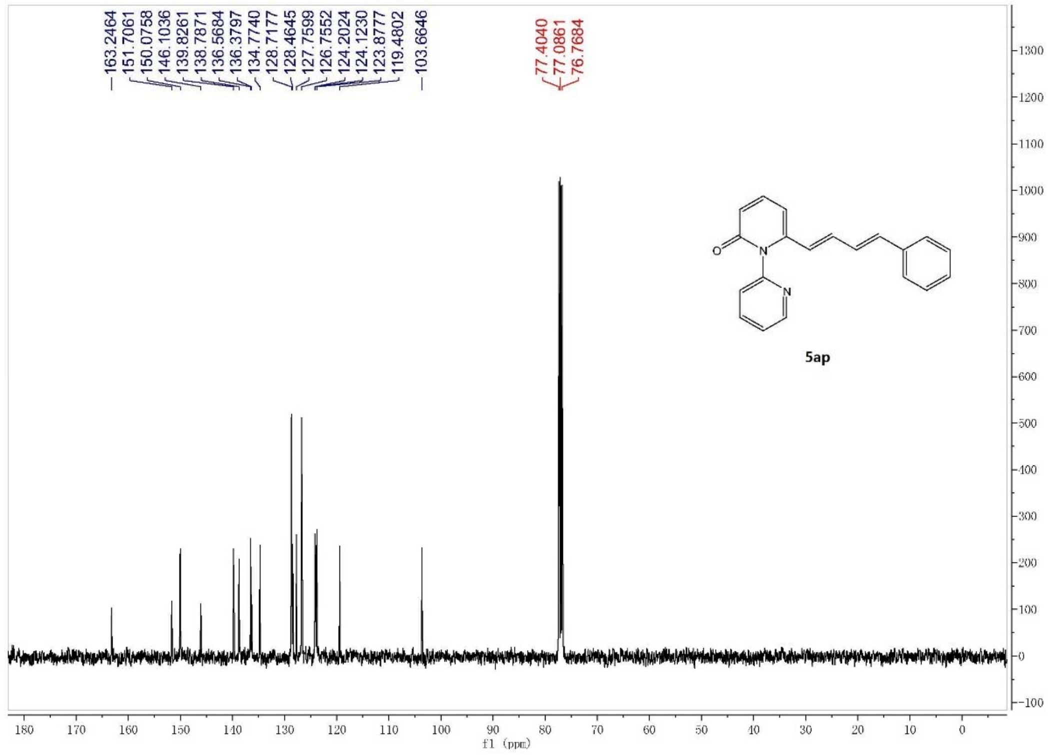

^{1}H and $^{13}C\{^{1}H\}$ NMR spectra of compound 5aq in $CDCl_3$

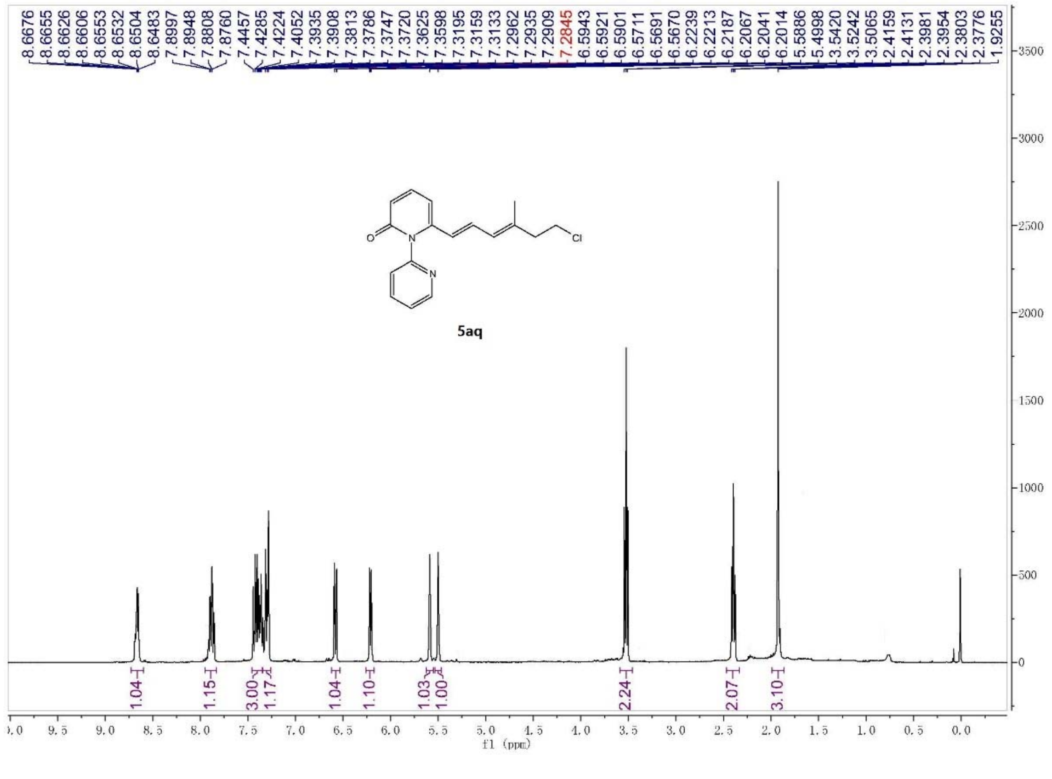

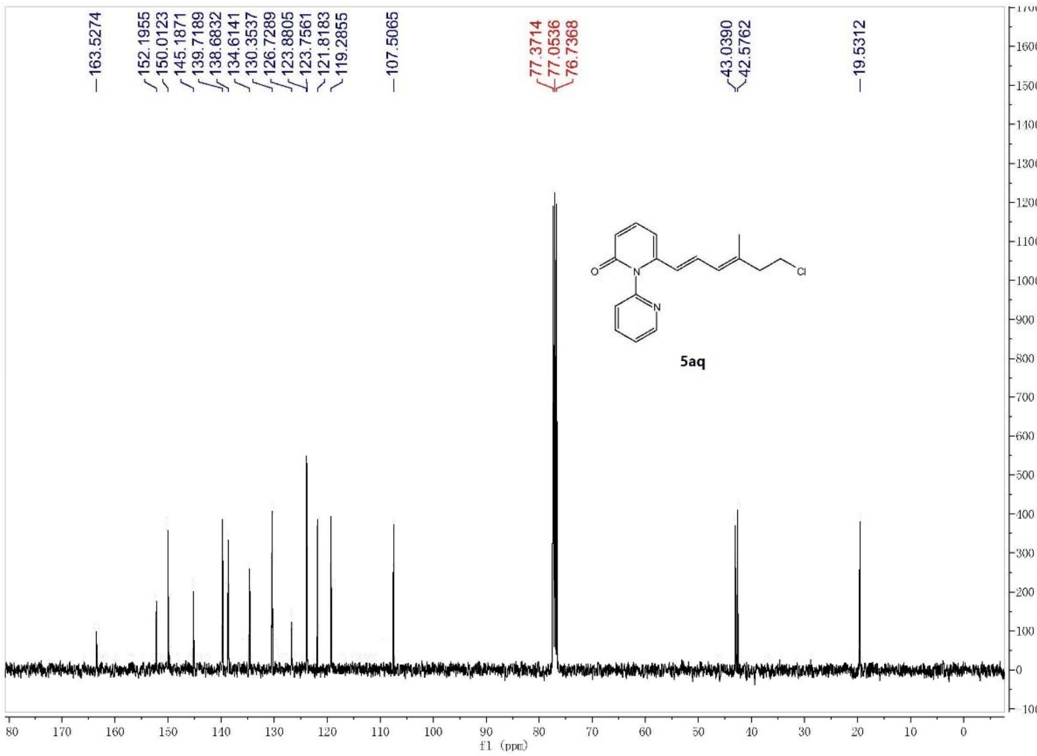

^{1}H and $^{13}C\{^{1}H\}$ NMR spectra of compound 5ar in $CDCl_3$

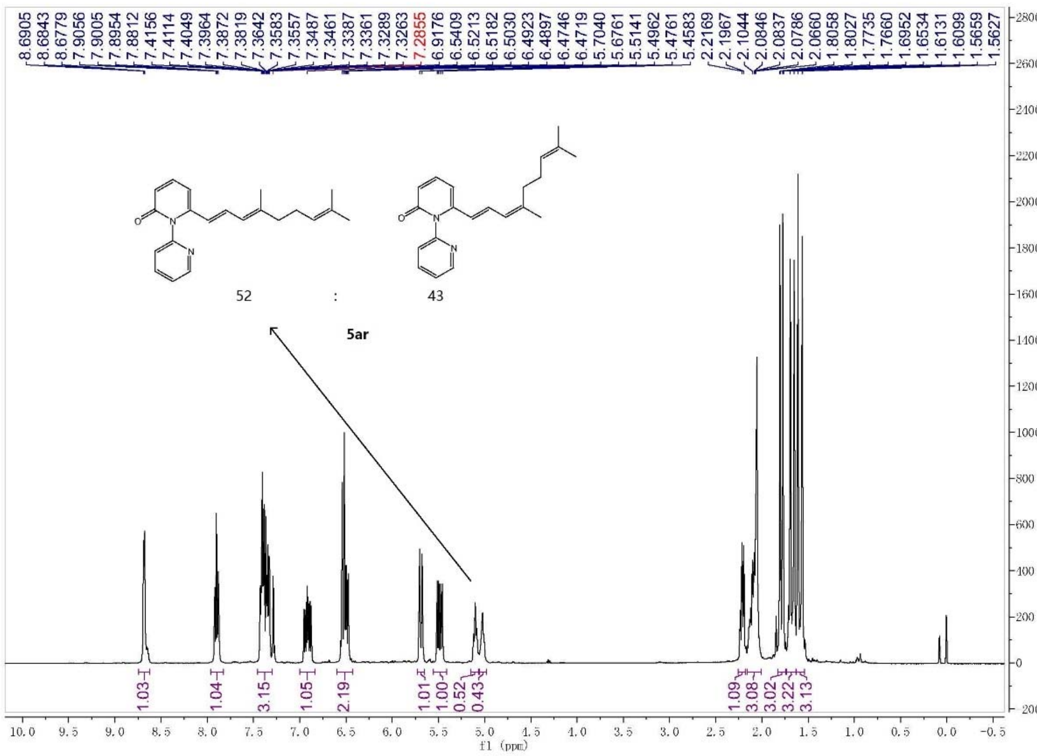

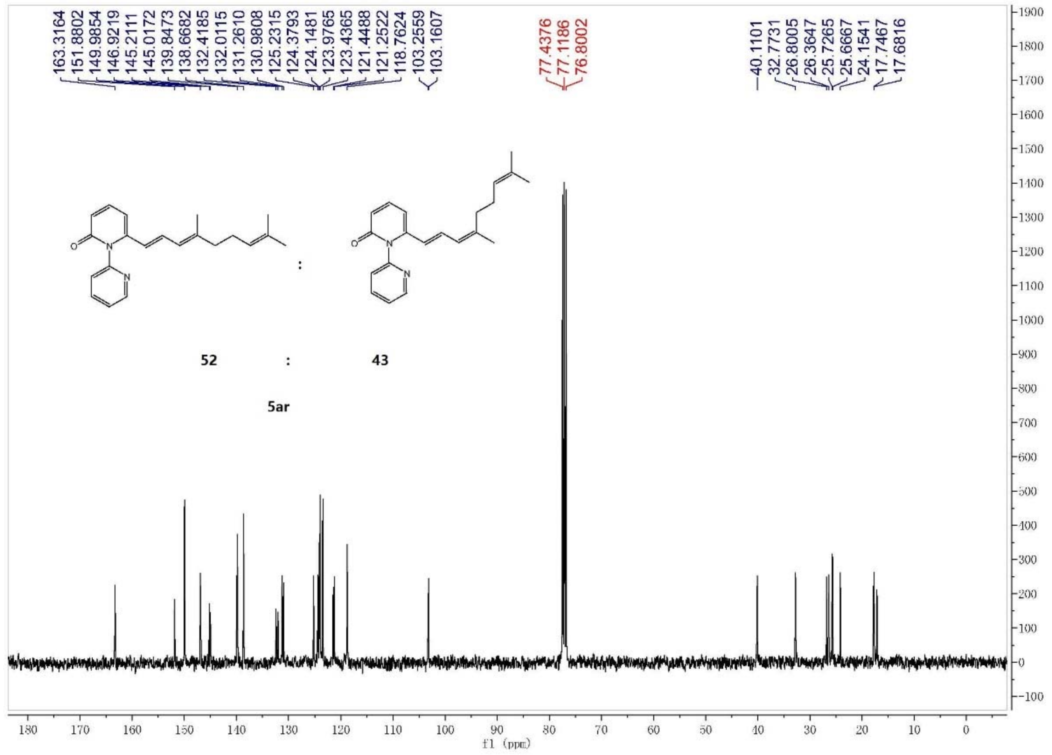

^{1}H and ^{13}C{^{1}H} NMR spectra of compound 5as in $CDCl_3$

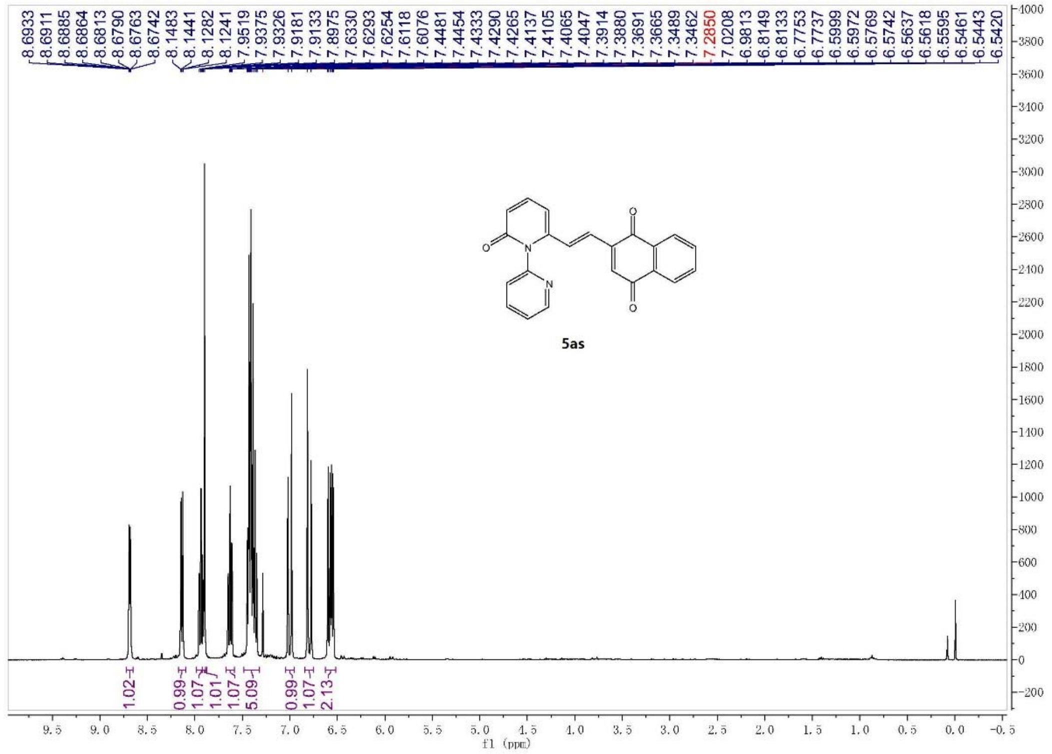

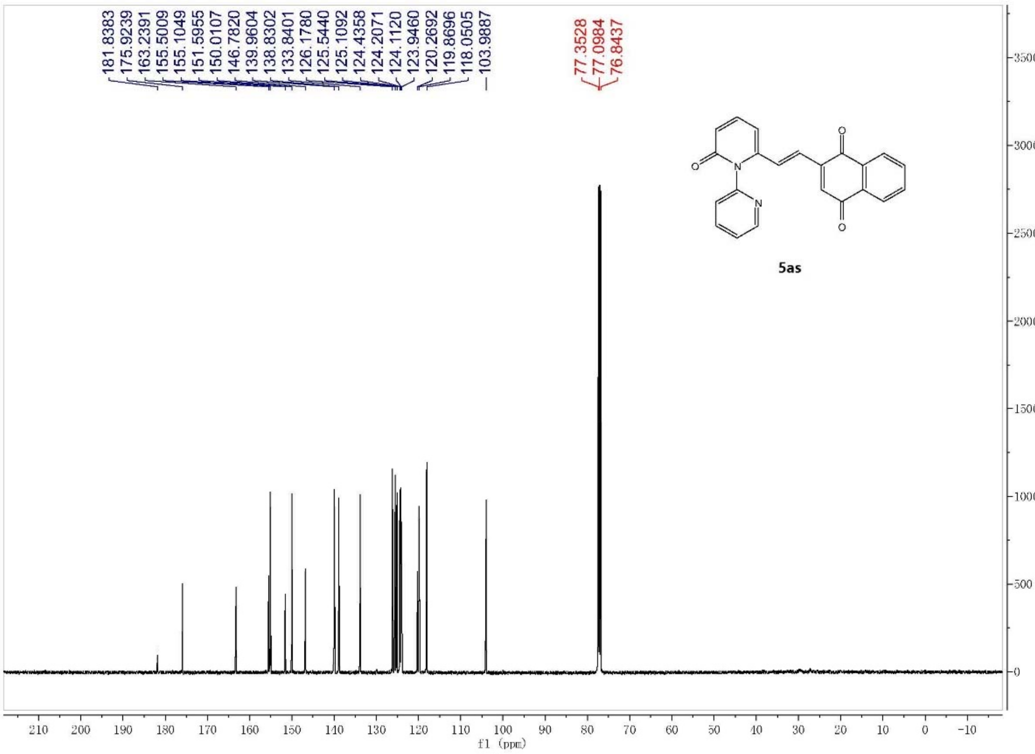

^{1}H and ^{13}C{^{1}H} NMR spectra of compound 5at in $CDCl_3$

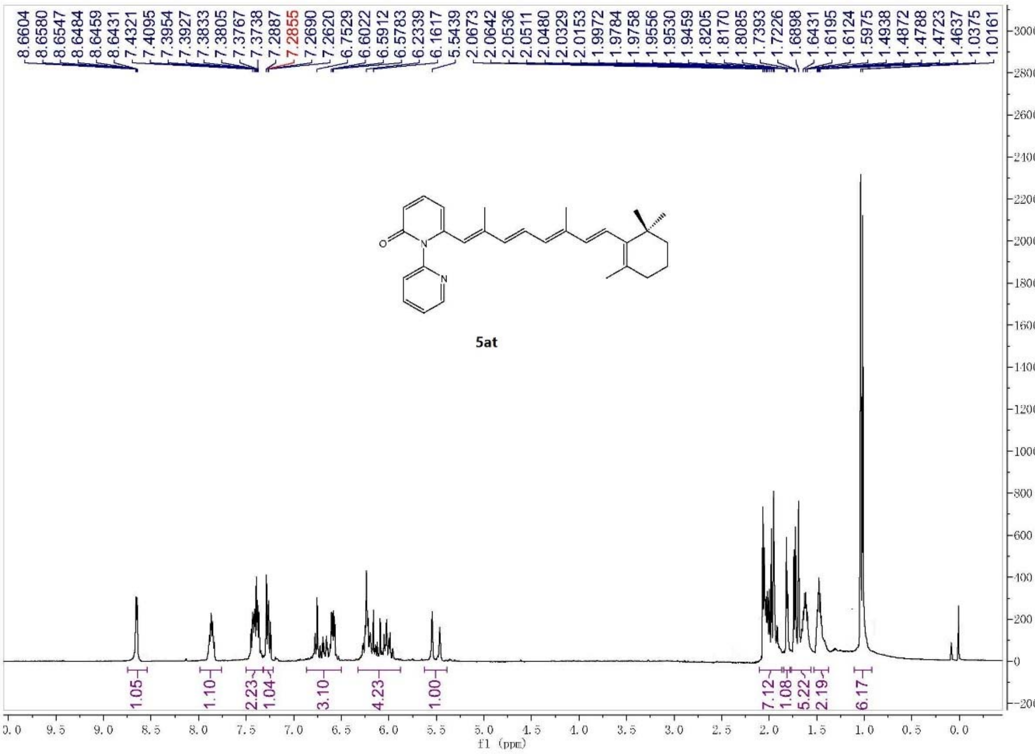

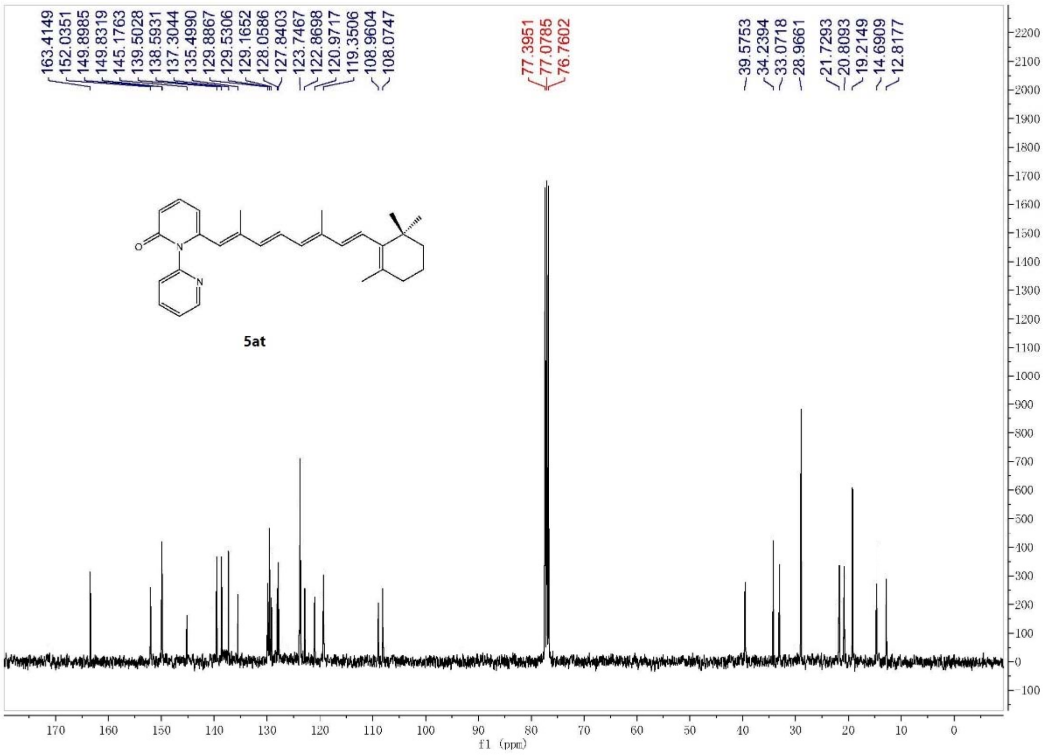

^{1}H and ^{13}C{^{1}H} NMR spectra of compound 5au in $CDCl_3$

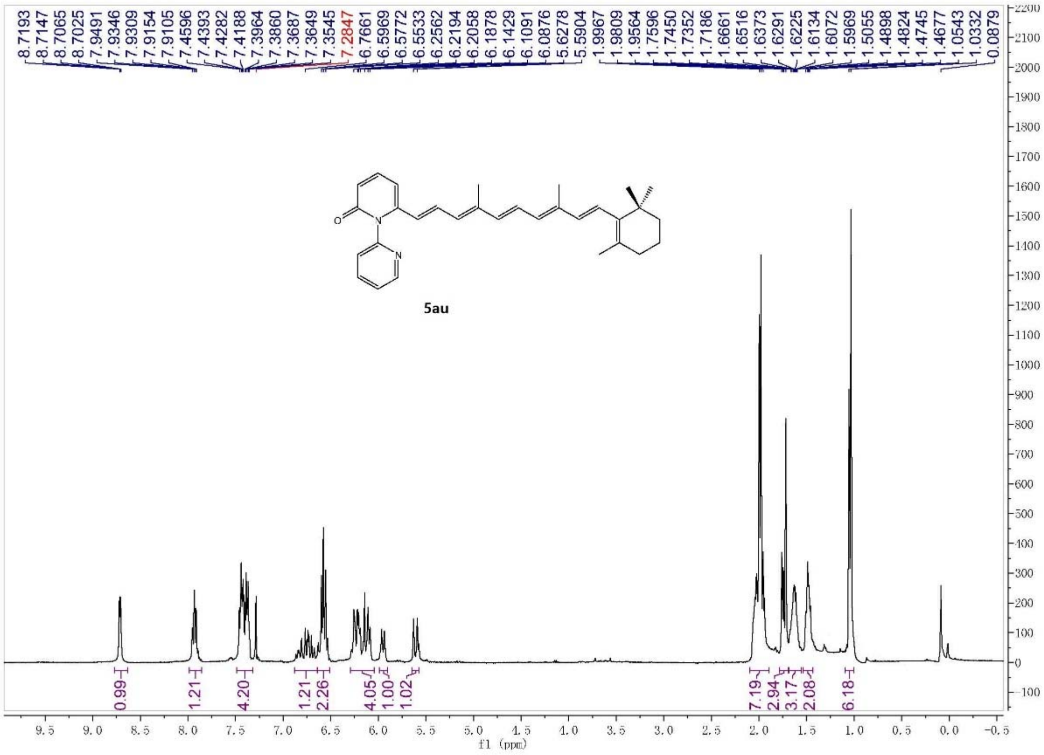

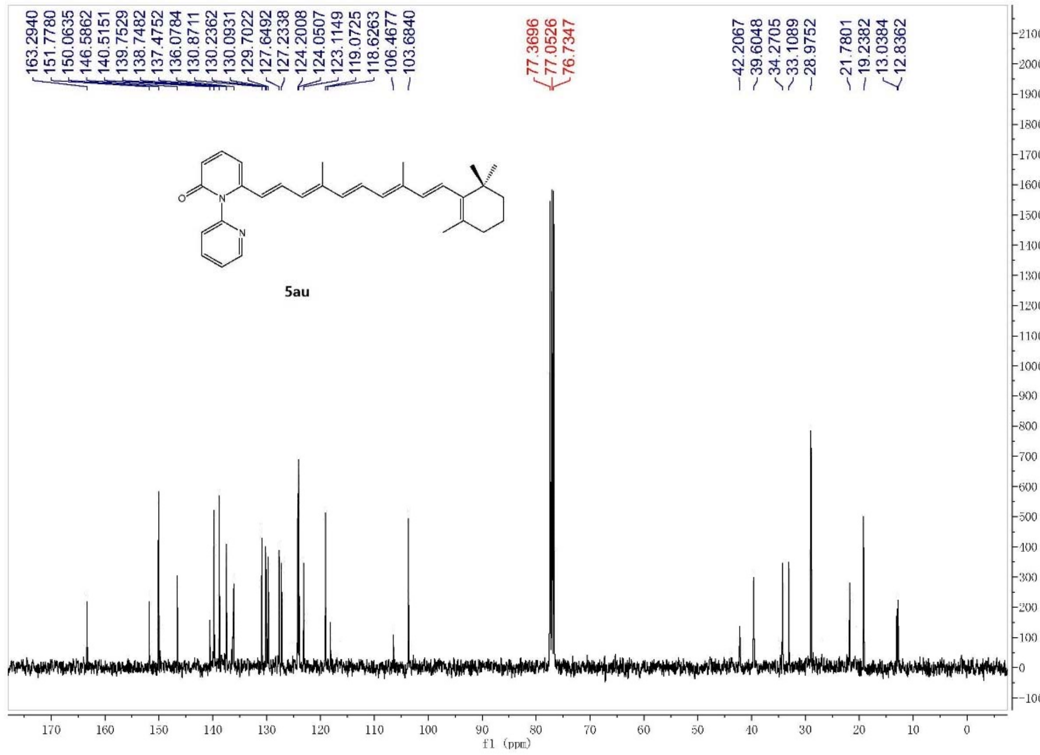

^1H and $^{13}C\{^1H\}$ NMR spectra of compound 5av in $CDCl_3$

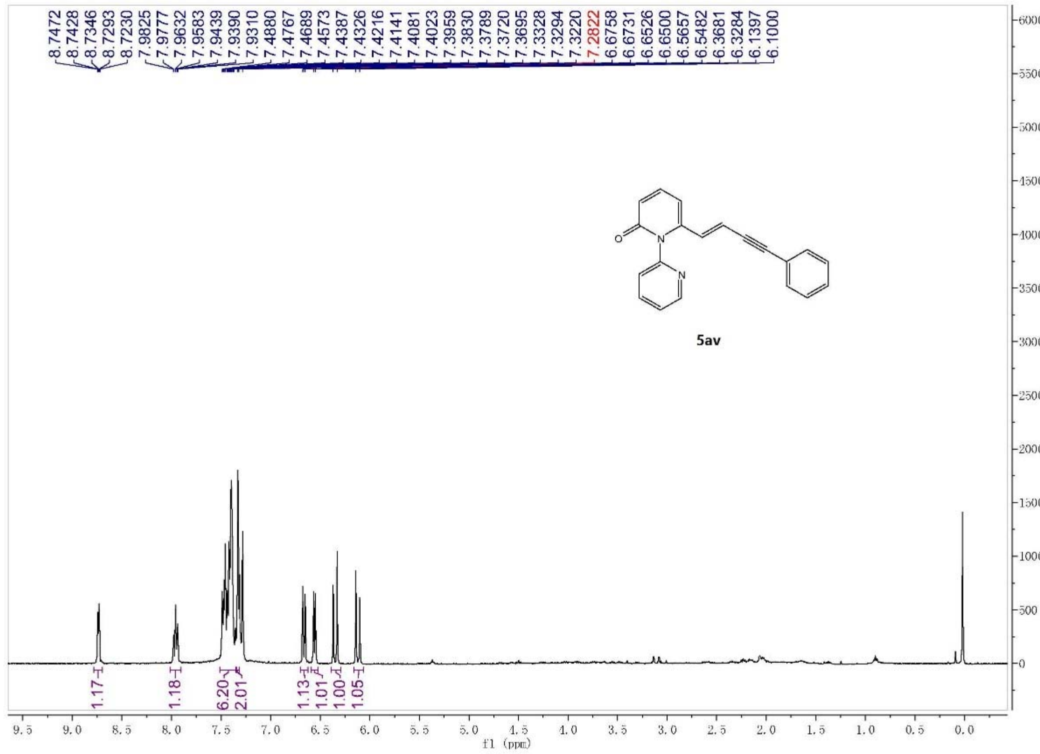

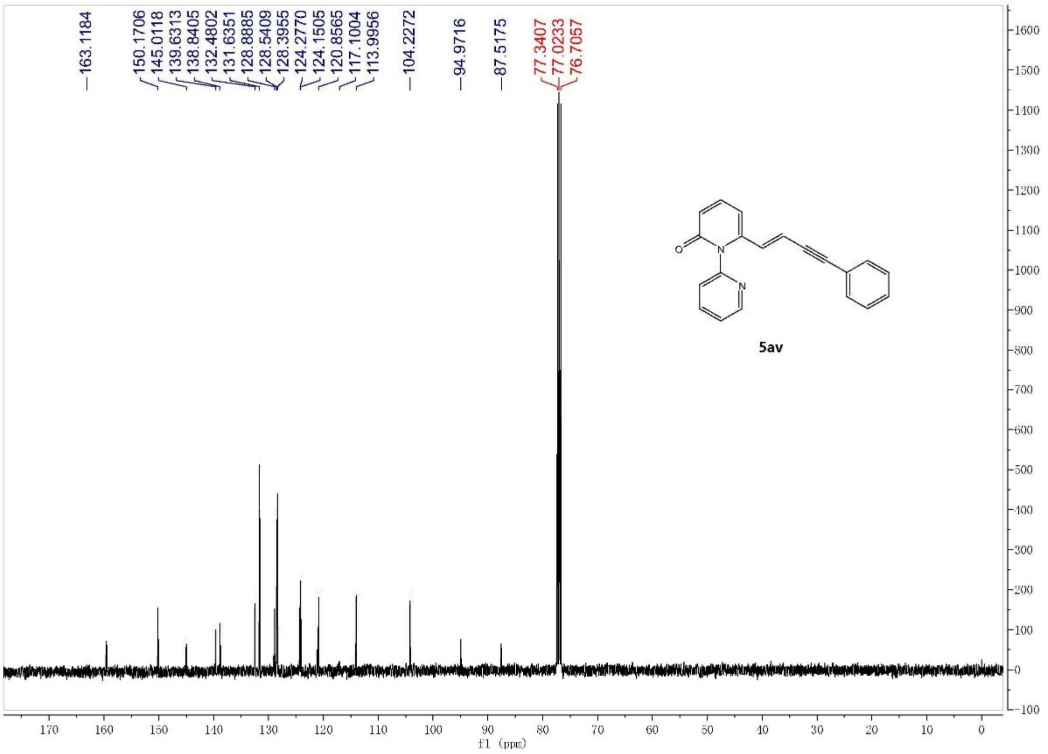

^{1}H and $^{13}C\{^{1}H\}$ NMR spectra of compound 6 in $CDCl_3$

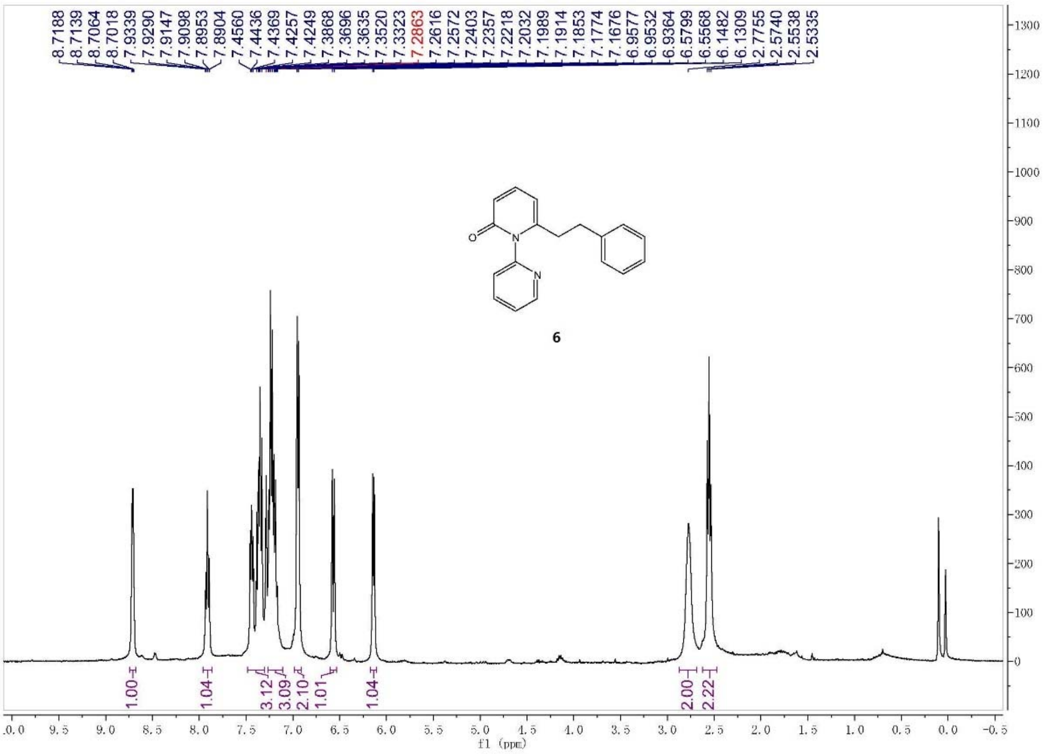

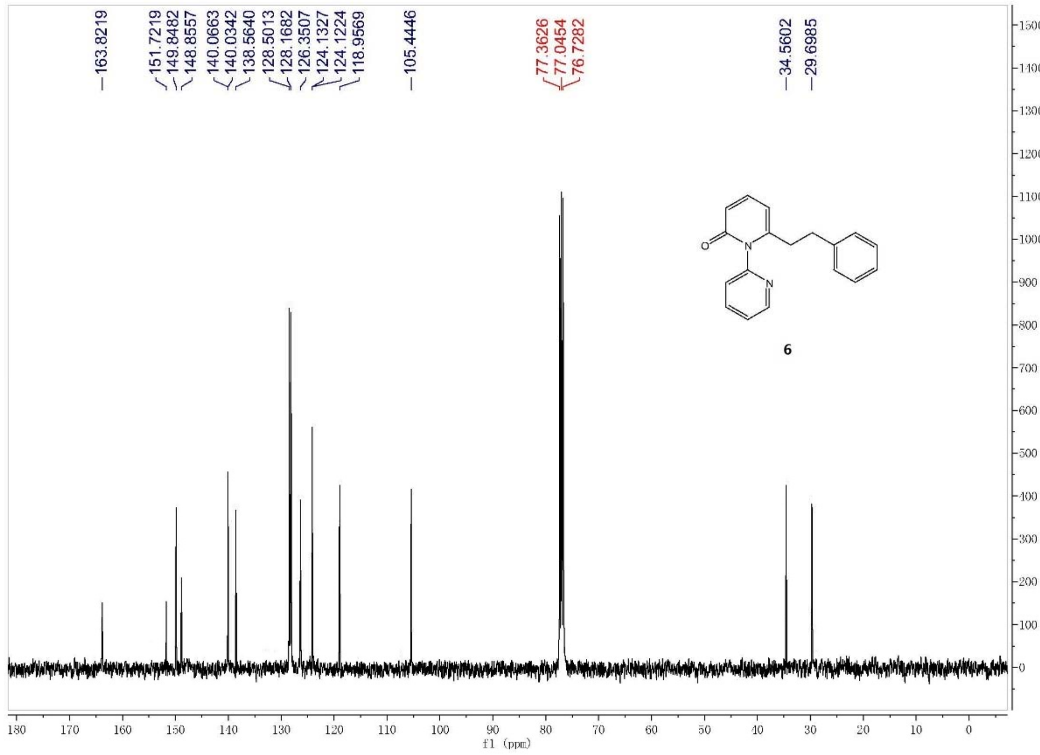

6

^{1}H and $^{13}C\{^{1}H\}$ NMR spectra of compound 7 in $CDCl_3$

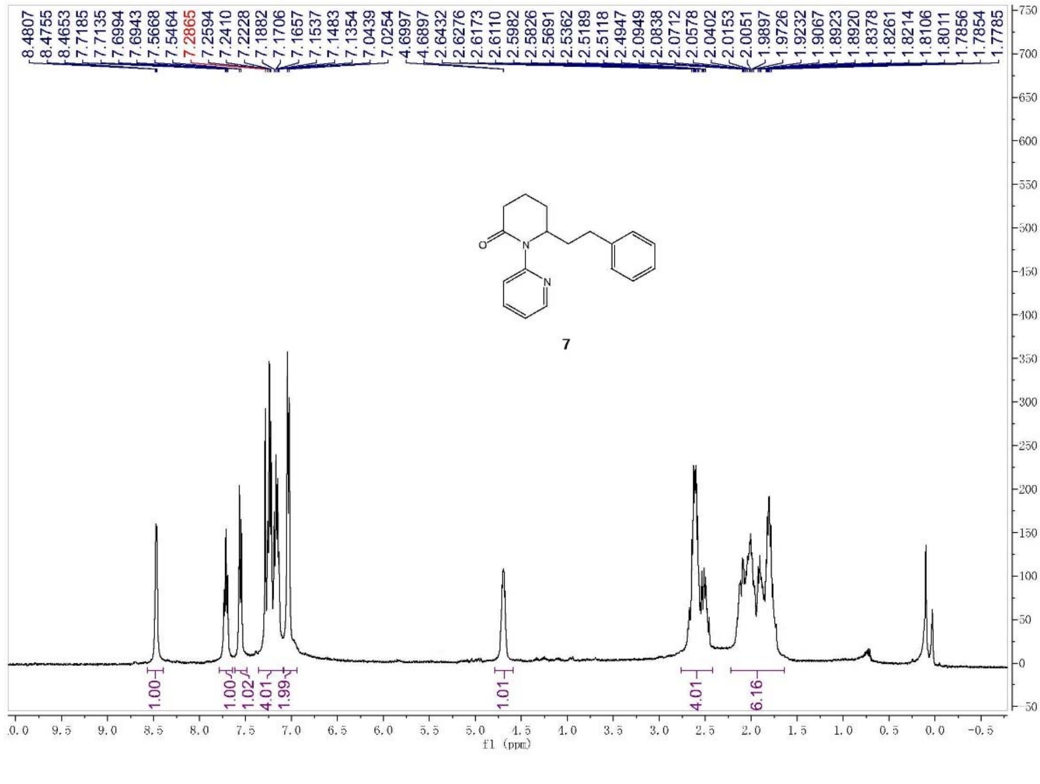

7

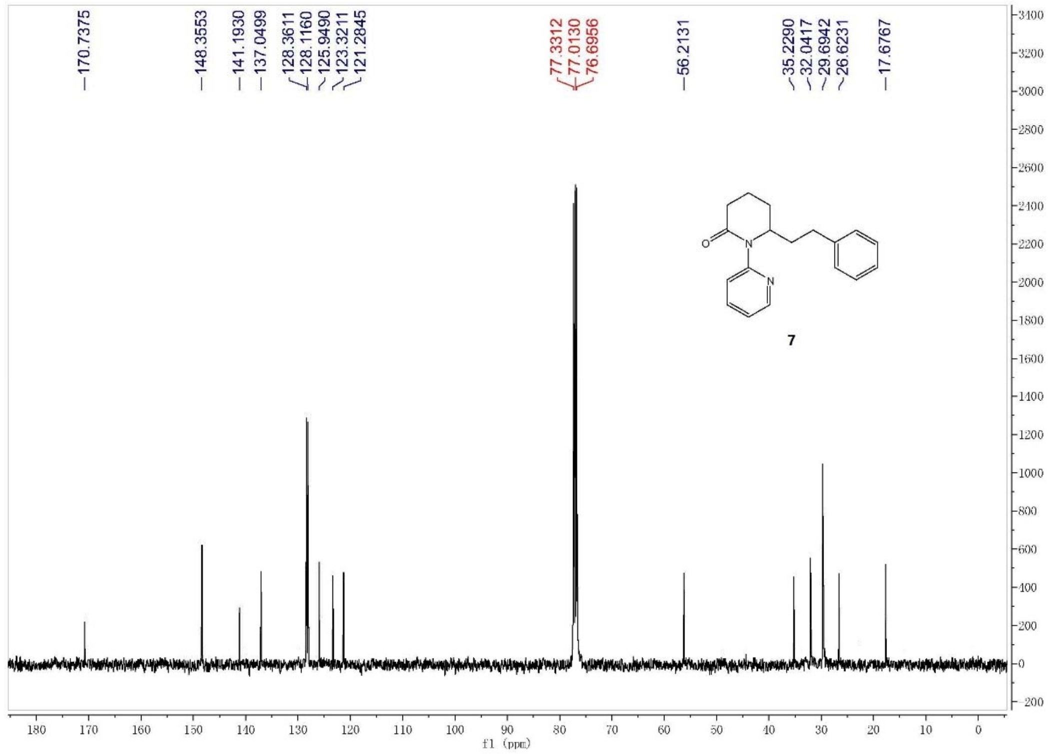

^{1}H and $^{13}C\{^{1}H\}$ NMR spectra of compound 8 in $CDCl_3$

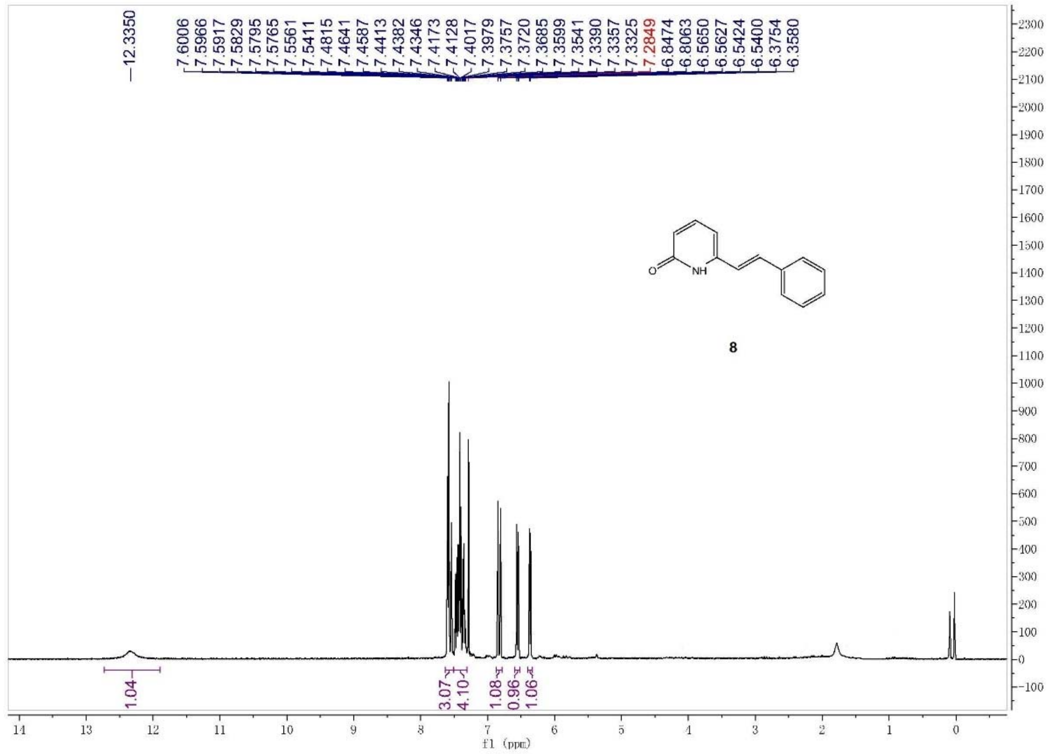

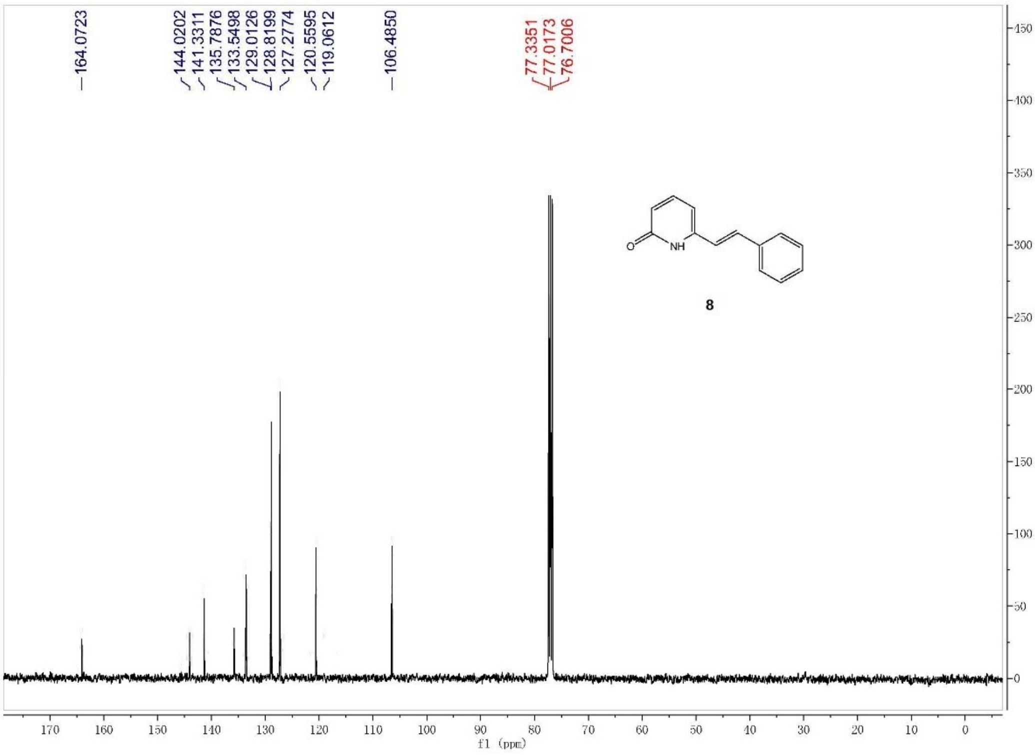

^1H and $^{13}C\{^1H\}$ NMR spectra of compound 9 in $CDCl_3$

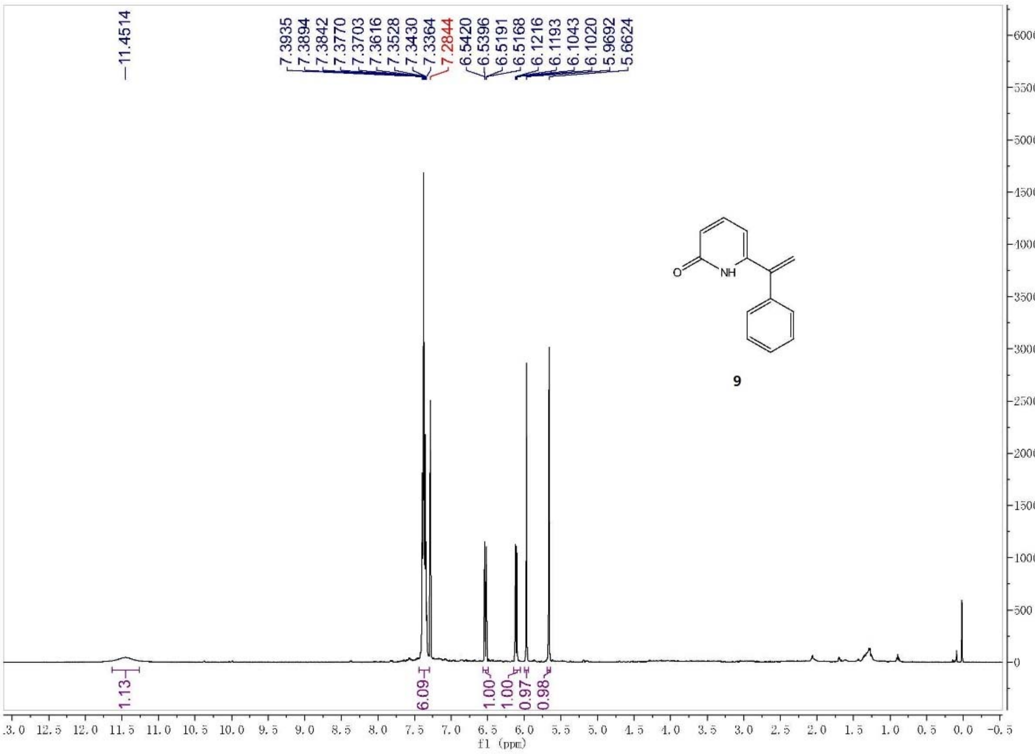

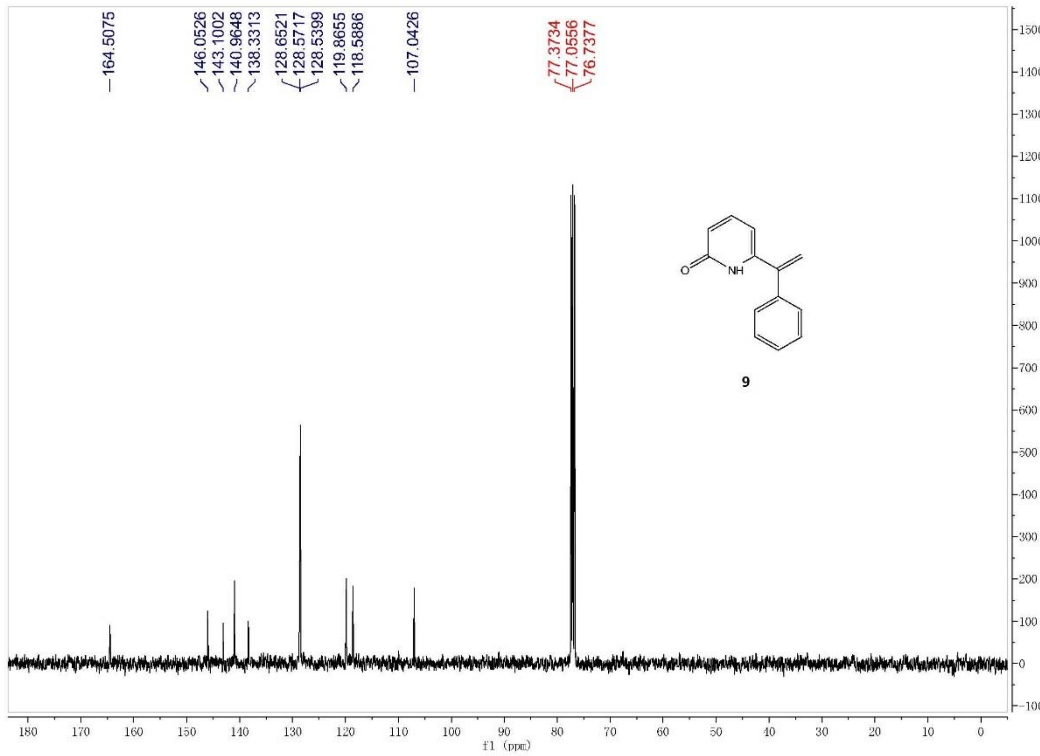

^{1}H and ^{13}C{^{1}H} NMR spectra of compound 10 in $CDCl_3$

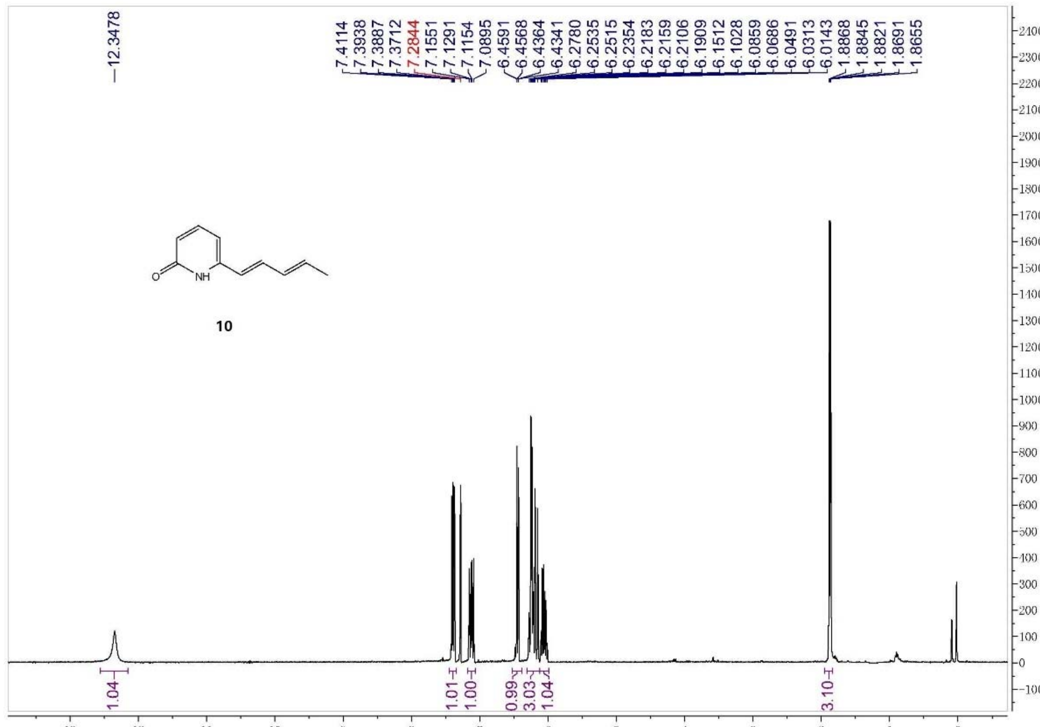

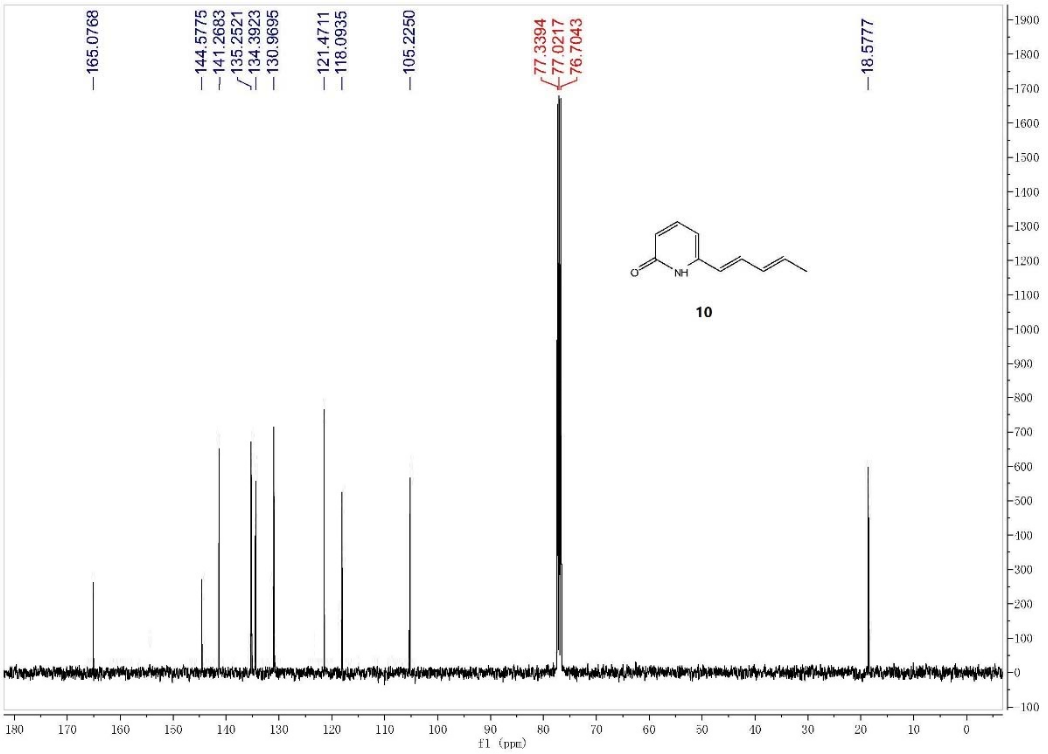

^{1}H and $^{13}C\{^{1}H\}$ NMR spectra of compound 11 in $CDCl_3$

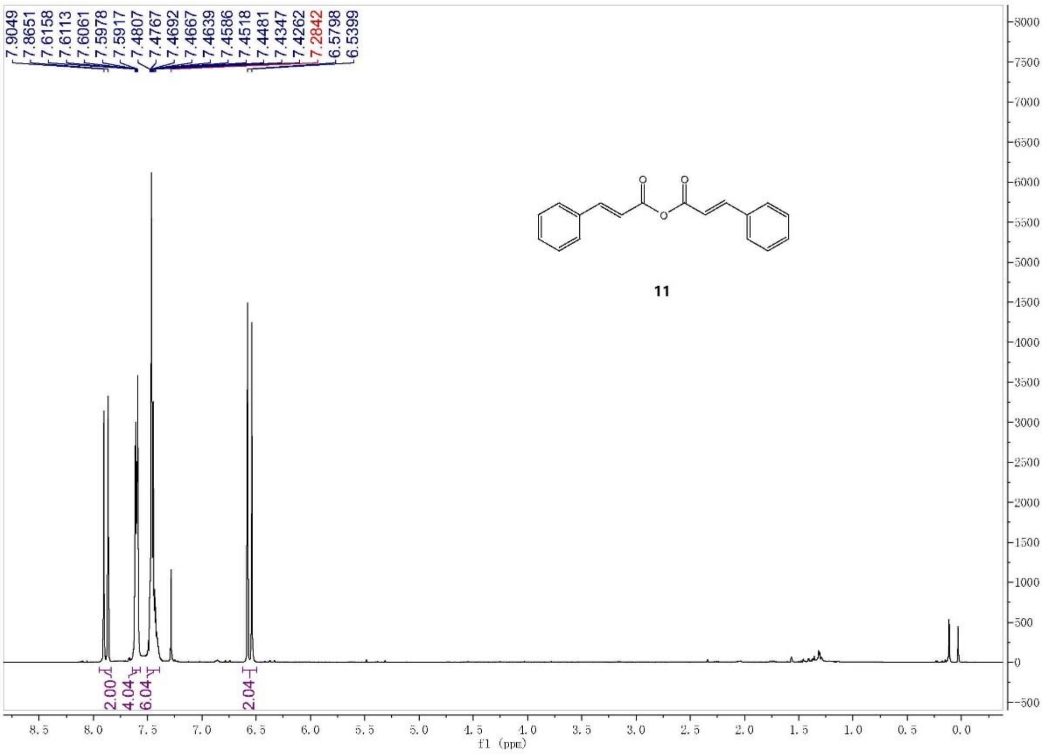

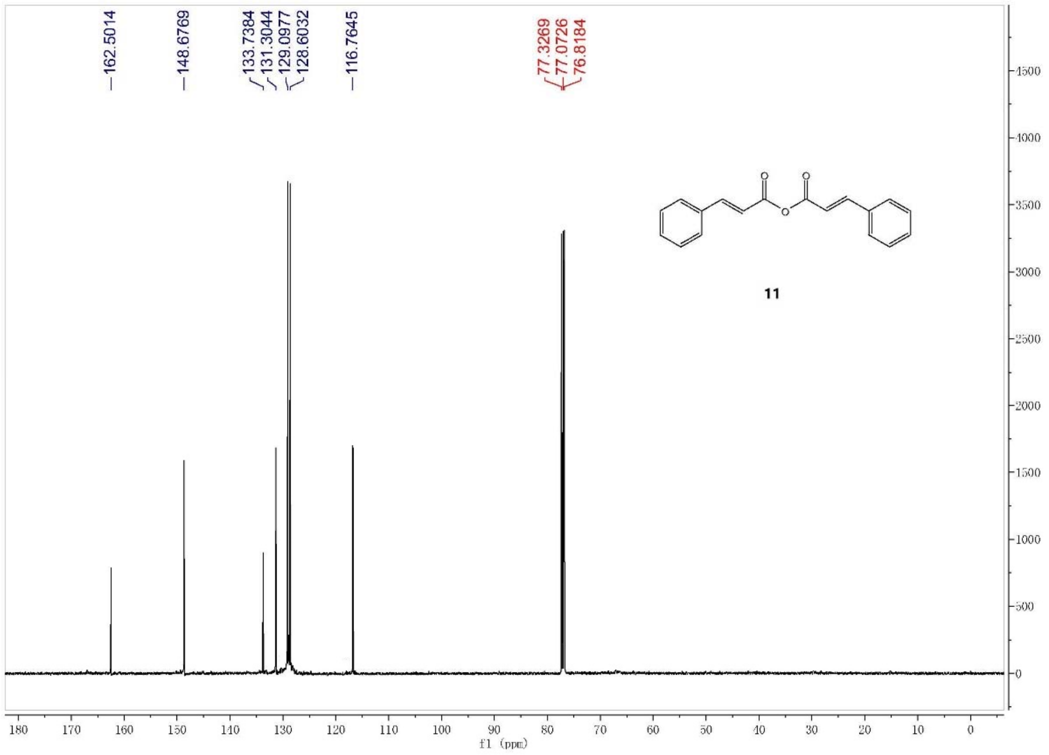
162.5014
148.6769
133.7384
131.3044
129.0977
128.6032
116.7645
77.3269
77.0726
76.8184
11
f1 (ppm)

10. X-ray crystal structure determination of 3ap

Light yellow block-like specimen of $C_{18}H_{13}ClN_2O$, was used for the X-ray crystallographic analysis. The X-ray intensity data were measured.

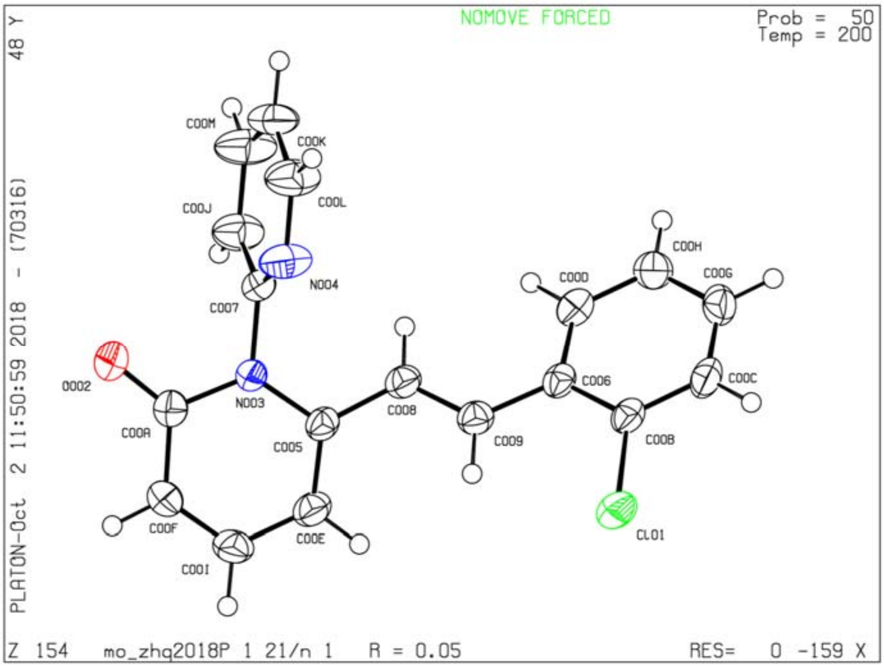

Table 1. Crystal data and structure refinement for **3ap**.

Identification code	mo_ZHQ2018100202_0ma
Empirical formula	$C_{18}H_{13}ClN_2O$
Formula weight	308.75
Temperature/K	199.99
Crystal system	monoclinic
Space group	$P2_1/n$
a/Å	5.6055(8)
b/Å	27.048(3)
c/Å	10.1964(14)
α/°	90.00
β/°	105.96
γ/°	90.00
Volume/Å^3	1486.4(3)
Z	4
ρ_{calc}g/cm^3	1.380
μ/mm^{-1}	0.260

F(000)	640.0
Crystal size/mm^3	0.28 × 0.2 × 0.18
Radiation	MoKα (λ = 0.71073)
2Θ range for data collection/°	5.14 to 55.1
Index ranges	-7 ≤ h ≤ 5, -35 ≤ k ≤ 35, -13 ≤ l ≤ 13
Reflections collected	10039
Independent reflections	3420 [R_{int} = 0.0651, R_{sigma} = 0.0785]
Data/restraints/parameters	3420/0/199
Goodness-of-fit on F^2	1.022
Final R indexes [I>=2σ (I)]	R_1 = 0.0535, wR_2 = 0.1086
Final R indexes [all data]	R_1 = 0.1059, wR_2 = 0.1274
Largest diff. peak/hole / e Å^{-3}	0.23/-0.32

Table 2 Fractional Atomic Coordinates (×10^4) and Equivalent Isotropic Displacement Parameters (Å^2×10^3) for **3ap**. U_{eq} is defined as 1/3 of of the trace of the orthogonalised U_{IJ} tensor.

**Atom**	***x***	***y***	***z***	**U(eq)**
Cl01	3232.8(13)	5304.8(2)	1054.2(6)	44.7(2)
O002	12805(3)	6535.2(6)	8148.5(19)	52.8(5)
N003	9237(3)	6282.0(6)	6639.0(17)	29.9(4)
N004	8878(5)	7013.3(7)	5384(2)	50.9(6)
C005	7703(4)	5917.4(8)	5892(2)	29.9(5)
C006	1975(4)	5945.8(8)	2824(2)	29.5(5)
C007	8511(4)	6797.1(7)	6465(2)	31.3(5)
C008	5345(4)	6063.9(8)	4975(2)	33.7(5)
C009	4310(4)	5824.9(8)	3815(2)	32.9(5)
C00A	11573(5)	6189.4(9)	7529(2)	36.3(6)
C00B	1339(4)	5744.0(8)	1505(2)	31.8(5)
C00C	-796(5)	5881.1(8)	536(2)	38.5(6)
C00D	282(4)	6277.1(8)	3118(2)	35.6(6)
C00E	8512(5)	5440.3(8)	6065(2)	38.8(6)
C00F	12338(5)	5682.5(8)	7620(2)	39.3(6)
C00G	-2402(5)	6215.8(9)	852(3)	41.1(6)
C00H	-1856(5)	6408.8(9)	2159(3)	39.5(6)
C00I	10843(5)	5326.0(8)	6924(2)	40.6(6)
C00J	7546(5)	7023.3(9)	7403(3)	49.8(7)
C00K	7257(6)	7751.7(9)	6075(3)	52.8(7)
C00L	8228(6)	7491.4(9)	5207(3)	58.0(8)
C00M	6907(6)	7514.5(10)	7197(3)	64.0(9)

Table 3 Anisotropic Displacement Parameters (Å^2×10^3) for **3ap**. The Anisotropic displacement factor exponent takes the form: $-2\pi^2[h^2a^{*2}U_{11}+2hka^*b^*U_{12}+\ldots]$.

**Atom**	**U_{11}**	**U_{22}**	**U_{33}**	**U_{23}**	**U_{13}**	**U_{12}**
Cl01	55.5(5)	37.4(3)	39.1(3)	-11.1(3)	9.3(3)	1.8(3)

O002	47.9(12)	38.2(9)	57.2(11)	-7.5(9)	-11.2(9)	-3.1(8)
N003	33.9(12)	25.5(9)	26.7(9)	-1.3(8)	2.4(9)	0.3(8)
N004	83.6(18)	32.0(11)	43.6(12)	2.1(9)	28.7(12)	9.1(11)
C005	37.0(14)	28.1(11)	24.2(11)	-1.8(9)	7.8(10)	-2.4(10)
C006	32.6(13)	25.6(10)	29.7(11)	0.6(9)	7.4(10)	-8.5(10)
C007	35.8(14)	25.3(10)	31.0(12)	-3.1(9)	6.0(11)	-0.6(10)
C008	39.7(15)	26.0(11)	33.8(12)	-1.4(9)	7.5(11)	-2.9(10)
C009	38.0(15)	28.3(11)	32.5(12)	0.4(10)	9.7(11)	-0.8(10)
C00A	38.0(15)	35.0(13)	32.5(12)	-1.3(10)	3.7(11)	0.1(11)
C00B	36.1(14)	27.8(11)	30.5(12)	-2.5(9)	7.8(11)	-7.8(10)
C00C	42.2(16)	36.5(13)	31.5(12)	-4.8(10)	1.0(12)	-11.9(12)
C00D	38.9(15)	33.9(12)	35.1(12)	-4.6(10)	11.9(12)	-7.1(11)
C00E	52.1(17)	26.5(11)	34.6(13)	-1.2(10)	6.7(12)	-4.6(11)
C00F	42.1(16)	34.7(12)	35.7(13)	3.9(10)	1.6(12)	5.7(11)
C00G	34.1(15)	38.5(13)	43.5(14)	3.9(11)	-1.5(12)	-7.2(11)
C00H	33.1(15)	36.4(12)	49.1(15)	-0.7(11)	11.3(12)	-1.3(11)
C00I	54.3(17)	28.9(11)	36.1(13)	5.9(10)	7.9(12)	8.1(12)
C00J	71(2)	39.3(13)	47.0(15)	3.4(12)	28.5(15)	10.5(13)
C00K	63(2)	26.6(12)	70.2(19)	1.7(13)	20.4(16)	9.6(12)
C00L	93(2)	35.2(14)	51.5(16)	9.7(12)	28.7(17)	10.5(15)
C00M	90(2)	41.3(15)	75(2)	-0.6(14)	45.9(19)	19.0(16)

Table 4 Bond Lengths for **3ap**.

**Atom**	**Atom**	**Length/Å**	**Atom**	**Atom**	**Length/Å**
Cl01	C00B	1.738(2)	C007	C00J	1.366(3)
O002	C00A	1.230(3)	C008	C009	1.333(3)
N003	C005	1.390(3)	C00A	C00F	1.432(3)
N003	C007	1.449(3)	C00B	C00C	1.377(3)
N003	C00A	1.395(3)	C00C	C00G	1.376(3)
N004	C007	1.313(3)	C00D	C00H	1.369(3)
N004	C00L	1.342(3)	C00E	C00I	1.393(3)
C005	C008	1.449(3)	C00F	C00I	1.345(3)
C005	C00E	1.363(3)	C00G	C00H	1.385(3)
C006	C009	1.454(3)	C00J	C00M	1.377(4)
C006	C00B	1.403(3)	C00K	C00L	1.356(4)
C006	C00D	1.397(3)	C00K	C00M	1.373(4)

Table 5 Bond Angles for **3ap**.

**Atom**	**Atom**	**Atom**	**Angle/°**	**Atom**	**Atom**	**Atom**	**Angle/°**
C005	N003	C007	120.32(18)	O002	C00A	C00F	125.4(2)
C005	N003	C00A	123.87(18)	N003	C00A	C00F	115.1(2)
C00A	N003	C007	115.73(17)	C006	C00B	Cl01	120.44(18)
C007	N004	C00L	116.2(2)	C00C	C00B	Cl01	117.88(17)
N003	C005	C008	118.48(18)	C00C	C00B	C006	121.7(2)

C00E	C005	N003	117.9(2)	C00G	C00C	C00B	120.3(2)
C00E	C005	C008	123.7(2)	C00H	C00D	C006	121.8(2)
C00B	C006	C009	121.5(2)	C005	C00E	C00I	120.8(2)
C00D	C006	C009	122.0(2)	C00I	C00F	C00A	121.4(2)
C00D	C006	C00B	116.5(2)	C00C	C00G	C00H	119.2(2)
N004	C007	N003	115.30(19)	C00D	C00H	C00G	120.5(2)
N004	C007	C00J	124.9(2)	C00F	C00I	C00E	120.9(2)
C00J	C007	N003	119.8(2)	C007	C00J	C00M	117.5(2)
C009	C008	C005	122.5(2)	C00L	C00K	C00M	118.5(2)
C008	C009	C006	126.6(2)	N004	C00L	C00K	123.7(3)
O002	C00A	N003	119.5(2)	C00K	C00M	C00J	119.1(3)

Table 6 Hydrogen Atom Coordinates (Å×10^4) and Isotropic Displacement Parameters (Å^2×10^3) for **3ap**.

**Atom**	***x***	***y***	***z***	**U(eq)**
H008	4504	6341	5209	40
H009	5188	5547	3615	39
H00C	-1161	5744	-355	46
H00D	622	6415	4007	43
H00E	7473	5182	5593	47
H00F	13934	5596	8183	47
H00G	-3867	6313	182	49
H00H	-2973	6634	2392	47
H00I	11384	4992	7020	49
H00J	7325	6848	8169	60
H00K	6830	8090	5910	63
H00L	8461	7657	4430	70
H00M	6231	7687	7824	77
